# Sensitivity and Specificity of a Urine Circulating Anodic Antigen Test for the Diagnosis of *Schistosoma haematobium* in Low Endemic Settings

**DOI:** 10.1371/journal.pntd.0003752

**Published:** 2015-05-14

**Authors:** Stefanie Knopp, Paul L. A. M. Corstjens, Artemis Koukounari, Colin I. Cercamondi, Shaali M. Ame, Said M. Ali, Claudia J. de Dood, Khalfan A. Mohammed, Jürg Utzinger, David Rollinson, Govert J. van Dam

**Affiliations:** 1 Wolfson Wellcome Biomedical Laboratories, Department of Life Sciences, Natural History Museum, London, United Kingdom; 2 Department of Epidemiology and Public Health, Swiss Tropical and Public Health Institute, Basel, Switzerland; 3 University of Basel, Basel, Switzerland; 4 Department of Molecular Cell Biology, Leiden University Medical Center, Leiden, The Netherlands; 5 Biostatistics Department, Institute of Psychiatry, Psychology and Neuroscience, Kings College London, London, United Kingdom; 6 Laboratory of Human Nutrition, Institute of Food, Nutrition, and Health, Eidgenössische Technische Hochschule (ETH) Zurich, Zurich, Switzerland; 7 Public Health Laboratory-Ivo de Carneri, Chake Chake, Pemba, United Republic of Tanzania; 8 Helminth Control Laboratory Unguja, Ministry of Health, Zanzibar Town, Unguja, United Republic of Tanzania; 9 Department of Parasitology, Leiden University Medical Center, Leiden, The Netherlands; Hitit University, TURKEY

## Abstract

**Background:**

Elimination of schistosomiasis as a public health problem and interruption of transmission in selected areas are key goals of the World Health Organization for 2025. Conventional parasitological methods are insensitive for the detection of light-intensity infections. Techniques with high sensitivity and specificity are required for an accurate diagnosis in low-transmission settings and verification of elimination. We determined the accuracy of a urine-based up-converting phosphor-lateral flow circulating anodic antigen (UCP-LF CAA) assay for *Schistosoma haematobium* diagnosis in low-prevalence settings in Zanzibar, Tanzania.

**Methodology:**

A total of 1,740 urine samples were collected in 2013 from children on Pemba Island, from schools where the *S*. *haematobium* prevalence was <2%, 2–5%, and 5–10%, based on a single urine filtration. On the day of collection, all samples were tested for microhematuria with reagent strips and for the presence of *S*. *haematobium* eggs with microscopy. Eight months later, 1.5 ml of urine from each of 1,200 samples stored at -20°C were analyzed by UCP-LF CAA assay, while urine filtration slides were subjected to quality control (QCUF). In the absence of a true ‘gold’ standard, the diagnostic performance was calculated using latent class analyses (LCA).

**Principal Findings:**

The ‘empirical’ *S*. *haematobium* prevalence revealed by UCP-LF CAA, QCUF, and reagent strips was 14%, 5%, and 4%, respectively. LCA revealed a sensitivity of the UCP-LF CAA, QCUF, and reagent strips of 97% (95% confidence interval (CI): 91–100%), 86% (95% CI: 72–99%), and 67% (95% CI: 52–81%), respectively. Test specificities were consistently above 90%.

**Conclusions/Significance:**

The UCP-LF CAA assay shows high sensitivity for the diagnosis of *S*. *haematobium* in low-endemicity settings. Empirically, it detects a considerably higher number of infections than microscopy. Hence, the UCP-LF CAA employed in combination with QCUF, is a promising tool for monitoring and surveillance of urogenital schistosomiasis in low-transmission settings targeted for elimination.

## Author Summary

The World Health Organization aspires to eliminate snail fever (schistosomiasis) as a public health problem and to interrupt the transmission of this disease in selected areas by 2025. Efforts to achieve these goals are currently being intensified. As a result, the prevalence and intensity of infection will decline in many parts of the world. To detect light-intensity infections, diagnostic tools with a high sensitivity and specificity are needed. We assessed the accuracy of a method that is able to diagnose schistosomiasis via the detection of circulating anodic antigen (CAA) in urine. We examined 1,200 urine samples from children living on Pemba Island, Tanzania, a low-endemic area targeted for schistosomiasis elimination. We found that the CAA-test had a considerably higher sensitivity than conventional urine filtration microscopy and reagent strips that are widely used in schistosomiasis control programs. The empirical prevalence of infection with the parasite *Schistosoma haematobium* determined by the CAA-test was up to 10 times higher than that obtained by urine filtration. Our results suggest that the CAA-test—in combination with urine filtration—is a promising approach for the diagnosis of *S*. *haematobium* in low-transmission settings that are targeted for elimination.

## Introduction

After many years of neglect, schistosomiasis and other parasitic worm infections are given considerable attention by the research community, non-governmental organizations, funding bodies, international organizations, policy makers, and disease control managers [1,2]. Indeed, drug donations, in conjunction with political and financial commitment for scaling up control interventions, have markedly expanded since the London Declaration on Neglected Tropical Diseases was launched in early 2012 [3,4]. For example, the World Health Organization (WHO) strategic plan 2012–2020 for schistosomiasis aims at morbidity control by 2020, and elimination of schistosomiasis as a public health problem and interruption of transmission in selected areas by 2025 [5]. The mainstay of morbidity control is preventive chemotherapy that is the large-scale administration of praziquantel to at-risk groups (e.g., school-aged children). To achieve elimination of schistosomiasis as a public health problem (i.e., the reduction of heavy infection intensities in the at-risk population to below 1%), additional public health measures are recommended, alongside intensified interventions in pockets of high transmission [5,6]. For interruption of transmission (i.e., reducing the incidence of infection to zero), it is essential to increase the frequency of preventive chemotherapy and to supplement it with additional control measures, such as improved access to clean water and adequate sanitation, and controlling intermediate host snails [5–9]. Monitoring the progress of control programs and rigorous surveillance to identify remaining or reemerging transmission hotspots and individuals with high infection levels (so-called superspreaders), will be relevant for tailoring an adequate response in close-to-elimination settings [5,6,10].

An accurate diagnosis is essential and should be adapted to the specific stage of a schistosomiasis control program [11,12]. Parasitological methods to detect *Schistosoma* eggs in stool (e.g., Kato-Katz thick smear [13]) or urine (e.g., urine filtration [14]), or reagent strips to detect microhematuria in urine [15] are widely used in control programs. However, while these methods are reasonably accurate in diagnosing moderate and heavy infection intensities, they show low sensitivity for detecting light-intensity infections [16,17]. For appropriate monitoring and surveillance in areas approaching schistosomiasis elimination, other, highly sensitive tools are needed [4,11,18–22]. Moreover, as the prevalence of infection decreases over the course of a control program, specificity becomes more important and will be an absolute requirement for certification of elimination [11,19].

A promising diagnostic approach that might be suitable for highly sensitive and specific diagnosis of very light *Schistosoma* infections is the detection of *Schistosoma* adult worm circulating anodic antigen (CAA) in serum and urine using an up-converting phosphor-lateral flow (UCP-LF) assay [20,23–25]. Concentration systems allowing the recognition of very low worm numbers have been developed, and currently four urine-based assays are available in a robust dry-reagent format [26,27]. These UCP-LF tests can detect 30 pg/ml, 3 pg/ml, 0.3 pg/ml, and 0.1 pg/ml CAA using 10 μl (UCAA10), 250 μl (UCAA250), 2 ml (UCAA2000), and 7.5 ml urine (UCAA7500), respectively [27].

The UCAA2000 (and perhaps the UCAA250) is currently considered to show the best trade-off between a high sensitivity and convenient field applicability [28]. However, the performance of different UCP-LF CAA assays as sensitive and specific diagnostic tools for non-invasive monitoring and surveillance remain to be determined in laboratories in *Schistosoma* low-endemic settings. Recently, the UCAA2000, as well as a variant of the test conducted with 500 μl serum, have been successfully applied for the diagnosis of *S*. *japonicum* infections in low transmission settings in the People’s Republic of China [29]. First validation attempts have also been made with the UCAA250 for *S*. *japonicum* and *S*. *mekongi* detection in banked urine samples from the Philippines and Cambodia, respectively [30].

Here, we assess the accuracy of the UCAA2000 for the diagnosis of *S*. *haematobium* in three low-prevalence settings (<2%, 2–5%, and 5–10%), as determined with a single urine filtration. In the absence of a true ‘gold’ standard, sensitivity and specificity were determined empirically and by means of latent class analysis (LCA). Urine samples were collected from children attending primary schools on Pemba Island, United Republic of Tanzania, where prevalences ranged between 1% and 10% according to a single urine filtration in a recent parasitological survey conducted in the frame of the Zanzibar Elimination of Schistosomiasis Transmission (ZEST) project in early 2013. The Zanzibar archipelago is deemed a setting where elimination of urogenital schistosomiasis is feasible and where a series of control measures, including biannual mass drug administration (MDA), snail control, and behavioral change interventions, are currently being implemented to achieve this goal and to learn about which intervention works best [6,31–33].

## Methods

### Ethics Statement

The study was approved by the Zanzibar Medical Research Ethics Committee (ZAMREC) in Zanzibar, United Republic of Tanzania (reference no. ZAMREC 0003/Sept/011), the ethics committee of Basel, Switzerland (reference no. EKBB 236/11), and the Institutional Review Board of the University of Georgia in the United States of America (project no. 2012-10138-0) [31]. The study is registered with the International Standard Randomized Controlled Trial Number register (identifier: ISRCTN48837681).

The purpose of collecting urine samples and potentially storing them for examination with newly developed and more sensitive diagnostic techniques was outlined in the participant information sheet that was explained in lay terms to the children in school and distributed to the parents for their information when asked to provide written informed consent on behalf of children’s participation in the study. All school-aged children were offered praziquantel (40 mg/kg) against schistosomiasis and albendazole (400 mg) against soil-transmitted helminthiasis free of charge in the frame of the island-wide MDA campaigns conducted in June 2013 and November 2013 as part of the elimination interventions.

### Sample Size Calculation

The required number of individuals per prevalence setting to detect significant differences in the diagnostic outcome was calculated with an equation given by Fleiss [34]. Based on preliminary laboratory findings, we estimated the ‘empirical’ prevalence outcomes with the UCAA2000 to be three times higher than with a single urine filtration in prevalence settings below 5% and at least two times higher in prevalence settings of at least 5%. Using a significance level of 5% and a power of 80%, the minimum required number of individuals that had to be examined per prevalence setting was 867 for settings <2%, 235 for settings 2–5%, and 303 for settings 5–10%, according to a single urine filtration. Hence, at least 1,405 urine samples were required from individuals stemming from the three respective prevalence settings.

### Study Area

The Zanzibar archipelago consist of two main islands, Unguja and Pemba, which are located in the Indian Ocean, approximately 70 km East and 180 km North-East, respectively, from Dar es Salaam, the economic capital of the United Republic of Tanzania located on the mainland’s coast. According to the 2012 population and housing census, Unguja consists of 210 and Pemba of 121 administrative areas (shehias) with an approximate combined population of 1.3 million inhabitants [35]. Achieving elimination of urogenital schistosomiasis as public health problem on Pemba and interruption of transmission on Unguja are the goals of the Zanzibar president, the Ministry of Health, and an alliance of institutions, including the Schistosomiasis Consortium for Operational Research and Evaluation (SCORE), WHO, the Schistosomiasis Control Initiative (SCI), the Natural History Museum (NHM), and the Swiss Tropical and Public Health Institute (Swiss TPH) [31,33]. Since early 2012, biannual MDA on the whole islands and additionally snail control and behavior change interventions in selected communities have been implemented to achieve the primary goals and to learn lessons about which intervention combination works best for elimination.

In the frame of a three-arm multi-year intervention trial funded by SCORE, annual parasitological surveys are carried out in 45 randomly selected shehias on both Unguja and Pemba to assess the extent of *S*. *haematobium* infections in school children and adults [31]. The urine samples used for the diagnostic investigations presented here were collected from children aged 9–12 years visiting primary schools in 16 shehias on Pemba Island between March and May 2013, more than 3 months after the last round of MDA that had been carried out in early November 2012. All urine samples were examined in the laboratories of the Public Health Laboratory-Ivo de Carneri (PHL-IdC) in Chake Chake, Pemba.

### Field Procedures

In each primary school, the headmaster and teachers were informed about the aims of the study. Classes of standards 3 and 4 were visited by the field team of the PHL-IdC and the purpose of the study was explained in lay terms to the children. All children aged 9–12 years were asked to line up, stratified by boys and girls, and every third child was selected to participate in the study until 130 children were reached. The name, age, sex, and additional demographic information of these children were recorded and they received an information sheet and a consent form to bring to their parents. If the parents agreed that their child participated, the children were asked to return the signed consent form the following day. After collection of the signed consent forms by the field team, children received a urine collection container (120 ml) and were asked to fill it with their own urine (urine collection occurred between 10 a.m. and 12 a.m.) and to give the filled containers to the field team.

### Laboratory Procedures

At the day of collection, between March and May 2013, all urine samples of sufficient amount (at least 10 ml) were examined by trained laboratory technicians for microhematuria using reagent strips (Hemastix; Siemens Healthcare Diagnostics GmbH, Eschborn, Germany), and for the presence and number of eggs detected under a microscope using the urine filtration method with polycarbonate filters (Sterlitech, Kent, WA, United States of America). All urine filters were covered with hydrophilic cellophane soaked in glycerol solution and the slides were stored for a potential second reading for quality control.

At the day of collection, if a sufficiently large amount of urine was submitted, 1.8 ml of urine was frozen and stored at -20°C from children with IDs 1–100 from each shehia for future examinations, before subjecting to reagent strip testing and urine filtration. The frozen samples from children from the 16 shehias selected for this study were examined with the UCAA2000 or UCAA250 assays in November 2013 at PHL-IdC. Four laboratory technicians received an in-depth training for preparation of samples and how to conduct the UCAA2000 and UCAA250 tests by two of the authors (PLAMC and GJvD) at PHL-IdC. Supervised by, and in collaboration with a trained post-doctoral fellow (CIC), the technicians examined the samples as described elsewhere [26,27] blinded to the reagent strip and initial urine filtration reading results.

In brief, for the UCAA2000 procedure, 1.5 ml of urine was mixed with 1.5 ml of 4% tri-chloro-acetic acid (TCA) in a 10-ml tube and centrifuged at 4,000 revolutions per min (rpm) using a table centrifuge (Hettich Universal 320 centrifuge; Tuttlingen, Germany) for 15 min. From each tube, 2.8 ml of the supernatant was added to an Amicon Ultra-4 Centrifugal Filter Device (Merck Millipore) and centrifuged at 4,000 rpm for 30–45 min. From the concentrate, 20 μl were added to a tube placed in a 96-wells rack, which contained dried UCP-conjugate that was resuspended in 100 μl of assay buffer. After incubation at 37°C at 900 rpm for 1 h on a microtiter-plate thermo-shaker (L079; Kisker Biotech GmbH & Co; Steinfort, Germany), one lateral flow strip was placed into the solution. After the solution’s volume had been soaked up and run through the strip, it was left in the tube to dry for at least 2 h (usually overnight). The following day, the strips were read in a portable strip reader (UCP-Quant: QIAGEN Lake Constance GmbH; Hilden, Germany) [27]. Scans were analyzed with Lateral Flow Studio version 3.03.05 (QIAGEN Lake Constance GmbH; Hilden, Germany). A similar procedure was followed for the UCAA250, except that the Amicon Ultra-0.5 Centrifugal Filter Devices were loaded two times, allowing a sample volume of 1 ml supernatant (representing 500 μl urine) to be tested. The devices were centrifuged in a benchtop microcentrifuge (Eppendorf Mini Spin; Hamburg, Germany) at 14,000 rpm for 2 x 15 min.

The stored urine filtration slides from all individuals, whose urines were examined with a UCP-LF CAA test, were retrospectively re-read between November 2013 and January 2014 by a post-doctoral fellow (CIC) blinded to the reagent strip, initial urine filtration, and UCP-LF CAA results. This second reading is indicated as quality control urine filtration (QCUF).

### Data Handling and Statistical Analysis

The results from the reagent strip testing for microhematuria, urine filtration, and QCUF results were recorded on paper laboratory forms and subsequently double entered into a Microsoft Excel 2010 electronic database (Microsoft Corporation 2010) and cleaned. Discrepant results in the double entry were traced back in the original paper record forms and corrected. Results of the UCAA2000 and UCAA250 tests were directly transferred from the UCP-Quant reader into an electronic format. Data were analyzed using STATA version 12 (StataCorp.; College Station, TX, United States of America) and Mplus V7 [36].

Microhematuria was graded into negative, trace, 1+, 2+, and 3+ according to the color chart provided by the manufacturer. *S*. *haematobium* egg numbers were recorded per 10 ml of urine. The concentration of CAA in urine was calculated using standard curves derived from daily freshly prepared concentration series of partly purified antigen and expressed as pg/ml. High and low specificity cut-offs were determined as described elsewhere [24,27]. A sample was considered positive at CAA values of >0.4 pg/ml, as indecisive at 0.2–0.4 pg/ml, and as negative at <0.2 pg/ml for the UCAA2000 assay. Samples tested with the UCAA250 were considered as positive at CAA levels of >1.4 pg/ml, indecisive at 0.7–1.4 pg/ml, and as negative at <0.7 pg/ml. Of note, applied cut-off values are slightly different from those described by Corstjens *et al*. (2014) [27], and directly related to the (slightly smaller) sample volume input and the concentration factor obtained with the Amicon concentration devices.

The selection of schools with *S*. *haematobium* prevalences of <2%, 2–5%, and 5–10% for inclusion into the present study was based on results of the initial urine filtration examination performed on the day of sample collection and including all children with written informed consent, microhematuria, and urine filtration results. For assessing diagnostic accuracy, however, we only included data from individuals with complete diagnostic results on (i) reagent strip testing; (ii) urine filtration reading; (iii) UCAA2000 testing (considering indecisive results either as positive (UCAA2000+) or as negative (UCAA2000-) or as missing, depending on the approach applied to calculate diagnostic accuracy described below); and (iv) QCUF reading into the final analysis. While urine samples stored for UCP-LF CAA examination were not selected fully at random (i.e., only urine samples of sufficient amount of the first 100 among 130 collected samples per school were stored), we nevertheless considered this approach as valid and assumed complete randomness of missing samples (and that missing values are unrelated to the status of *S*. *haematobium* infection), since the overall percentage of positive individuals detected by the initial urine filtration did not differ between the initially sampled group (3.3%; Table 1) and the group included into the final analysis (3.4%; Table 2).

**Table 1:** *S*. *haematobium* prevalence results according to single urine filtration readings.

						Initial urine filtration reading	
Prevalence level	School	Number of children examined	Females	Males	Median (range) age (years)	*S*. *haematobium* positive	%
<2%	Ng'ombeni	105	53	52	10 (9–11)	1	1.0
	Kengeja	91	52	39	10 (9–12)	1	1.1
	Kangani	125	71	54	11 (10–12)	2	1.6
	Wesha	116	65	51	11 (9–13)	2	1.7
	Chanjamjawiri	112	60	52	11 (9–12)	2	1.8
	Kwale	109	64	45	11 (9–12)	2	1.8
	Mbuzini	109	50	59	11 (8–12)	2	1.8
	Mtambile	109	70	39	10 (9–12)	2	1.8
2–5%	Wawi	103	52	51	10 (9–12)	3	2.9
	Ole	122	68	54	11 (8–12)	4	3.3
	Makangale	90	57	33	11 (9–12)	3	3.3
	Mkanyageni	123	74	49	11 (9–12)	6	4.9
5–10%	Konde A	118	66	52	11 (9–12)	6	5.1
	Ngwachani	93	48	45	11 (9–12)	6	6.5
	Kinowe	121	60	61	11 (9–12)	8	6.6
	Shungi	94	42	52	11 (9–12)	8	8.5
	**Total**	**1,740**	**952**	**788**	**11**	**58**	**3.3**

Primary schools from Pemba selected to be included in the study according to the initial *S*. *haematobium* prevalence results as determined with a single urine filtration reading at the day of sample collection between March and May 2013.

**Table 2:** *S*. *haematobium* prevalences in children visiting 16 primary schools on Pemba in 2013, stratified by diagnostic approach.

Prevalence level	School	Number of children examined	Micro-hematuria		UF		QCUF		UCAA2000-		UCAA2000+		UCAA250-		UCAA250+	
			**No. pos**	**%**	**No. pos**	**%**	**No. pos**	**%**	**No. pos**	**%**	**No. pos**	**%**	**No. pos**	**%**	**No. pos**	**%**
<2%	Ng'ombeni	75	2	2.7	1	1.3	1	1.3	1	1.3	2	2.7				
	Kengeja	68	2	2.9	1	1.5	2	2.9	no observation		no observation		10	14.7	21	30.9
	Kangani	95	2	2.1	2	2.1	2	2.1	(10/79)	12.7	(16/79)	20.3	(0/16)	0.00	(0/16)	0.00
	Wesha	69	1	1.5	2	2.9	4	5.8	5	7.3	5	7.3	-	-	-	-
	Chanjamjawiri	80	0	0.0	1	1.3	4	5.0	14	17.5	24	30.0	-	-	-	-
	Kwale	86	1	1.2	0	0.0	0	0.0	5	5.8	6	7.0	-	-	-	-
	Mbuzini	76	1	1.3	1	1.3	1	1.3	5	6.6	8	10.5	-	-	-	-
	Mtambile	81	3	3.7	1	1.2	2	2.5	15	18.5	20	24.7	-	-	-	-
	**Total setting** *	**546**	**10**	**1.8**	**8**	**1.5**	**14**	**2.6**	**55**	**10.1**	**81**	**14.8**	**-**	**-**	**-**	**-**
2–5%	Wawi	76	6	7.9	2	2.6	4	5.3	5	6.6	7	9.2	-	-	-	-
	Ole	92	5	5.4	4	4.4	5	5.4	21	22.8	24	26.1	-	-	-	-
	Makangale	71	2	2.8	1	1.4	1	1.4	3	4.2	11	15.5	-	-	-	-
	Mkanyageni	87	6	6.9	4	4.6	5	5.8	10	11.5	14	16.1	-	-	-	-
	**Total setting** *	**326**	**19**	**5.8**	**11**	**3.4**	**15**	**4.6**	**39**	**12.0**	**56**	**17.2**	**-**	**-**	**-**	**-**
5–10%	Konde A	88	0	0.0	4	4.6	5	5.7	6	6.8	8	9.1	-	-	-	-
	Ngwachani	70	5	7.1	5	7.1	5	7.1	26	37.1	28	40.0	-	-	-	-
	Kinowe	90	5	5.6	5	5.6	10	11.1	21	23.3	30	33.3	-	-	-	-
	Shungi	80	10	12.5	8	10.0	8	10.0	12	15.0	15	18.8	-	-	-	-
	**Total setting** *	**328**	**20**	**6.1**	**22**	**6.7**	**28**	**8.5**	**65**	**19.8**	**81**	**24.7**	**-**	**-**	**-**	**-**
	**Total** *	**1200**	**49**	**4.1**	**41**	**3.4**	**57**	**4.8**	**159**	**13.3**	**218**	**18.2**	**-**	**-**	**-**	**-**

UF: initial urine filtration at the day of sample collection between March and May 2013; QCUF: second reading of the urine filtration slide for quality control purposes between November 2013 and January 2014; UCP-LF CAA: up-converting phosphor-lateral flow assay detecting circulating anodic antigen in urine; UCAA2000+: UCP-LF CAA prepared with 1.5 ml of urine, indecisive results were considered as positive; UCAA2000-: UCP-LF CAA prepared with 1.5 ml of urine, indecisive results were considered as negative; UCAA250+: UCP-LF CAA prepared with 250 μl of urine, indecisive results were considered as positive; UCAA250-: UCP-LF CAA prepared with 250 μl of urine, indecisive results were considered as negative.

*Only participants with a UCAA2000 were included in analysis.

From this subsample, we calculated ‘empirical’ prevalences obtained by each diagnostic method, assuming 100% test specificity. Diagnostic accuracy parameters, including 95% confidence intervals (CIs), were assessed using two different approaches. In the first approach, we considered the combined results of QCUF and UCAA2000+ as imperfect ‘gold’ standard and an individual was regarded as true-positive when the QCUF and/or UCAA2000+ indicated a *S*. *haematobium* infection. A test specificity of 100% was assumed for each method. The sensitivity of each individual test was determined by calculating the proportion of positives that were correctly identified by the test when compared to the imperfect ‘gold’ standard. The sensitivity of all diagnostic tests was calculated for (i) combined data from all individuals included into the final analysis and (ii) stratified data according to the originally selected different prevalence levels (<2%, 2–5%, and 5–10%). To assess a correlation between CAA pg/ml levels and the number of eggs detected in 10 ml urines or microhematuria grading identified with reagent strips, we applied the non-parametric Spearman’s rank correlation test.

In the second approach, in the absence of a true ‘gold’ standard, we used LCA to estimate the sensitivity, specificity, and model estimated prevalences for reagent strip, QCUF, and UCAA2000 [37–39]. Four LCA models were applied and validated. The exact procedure is presented in a supplementary file (S1 Models) and model details have been described by Ibironke and colleagues (2012) [38]. The four LCA models were fitted using MPlus V7 [36] with full information maximum likelihood estimation and assuming that data were missing at random. We included the indecisive results of the UCAA2000 in all LCA models by considering them as ‘missing’ and not forcing them in a positive or negative category [40]. The four LCA models were evaluated according to the lowest Bayesian information criterion (BIC) and Akaike information criterion (AIC) as indications of the best model fit and parsimony in combination with different biological plausible scenarios and tests of assumptions. In the results section, we present results from LCA model 1 (Table S1, Model 1 in S1 Models).

## Results

### Operational Results

**Fig 1:**
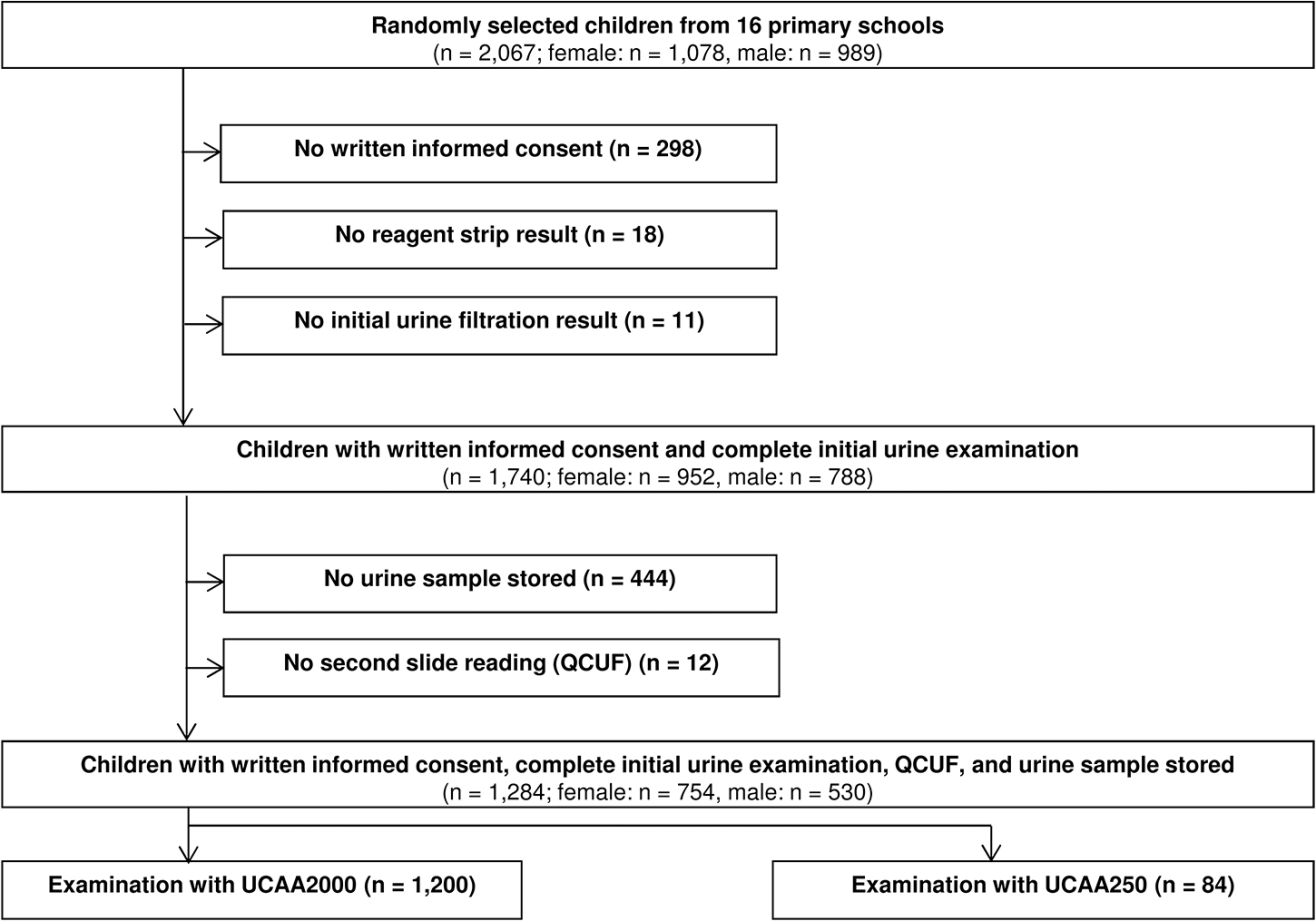
Flowchart detailing study participation and urine sampling procedures. Flowchart indicating the inclusion and exclusion of data for determining the accuracy of different methods for the diagnosis of *Schistosoma haematobium* in children from Pemba, United Republic of Tanzania, in 2013. UCP-LF CAA: up-converting phosphor-lateral flow assay detecting circulating anodic antigen in urine; UCAA2000: UCP-LF CAA prepared with 1.5 ml of urine; UCAA250: UCP-LF CAA prepared with 250 μl of urine.

To meet the prevalence thresholds and sample size for the study, we selected eight primary schools with a prevalence of *S*. *haematobium* <2%, four schools with a prevalence of 2–5%, and four schools with a prevalence of 5–10% based on single urine filtration readings per child (Table 1). From the 16 selected schools, 2,067 children were randomly selected to participate in the annual parasitological survey in 2013. Among them, 298 did not provide written informed consent from their parents and were therefore not asked to submit a urine sample (Fig 1). An additional 29 children did not submit a urine sample of a sufficiently large amount to perform reagent strip and urine filtration examinations. Hence, the initial *S*. *haematobium* prevalence at the unit of the school was calculated from single urine filtration results of 1,740 children. Among the 1,740 children examined with the initial urine filtration and reagent strip, 444 had no urine sample stored for future analysis, and 12 urine filtration slides were not available for reexamination by the quality control reader. Finally, UCP-LF CAA and QCUF readings were available from 1,284 children. The UCAA2000 and UCAA250 were applied on 1,200 and 84 urine samples, respectively.

The sample sizes of the UCAA2000 tests for prevalence settings <2%, 2–5%, and 5–10% were 546, 326, and 328, respectively. While the numbers for the latter two prevalence settings are in line with our initial sample size calculation, there were fewer UCAA2000 tests performed for the lowest prevalence settings. This is due to interim analyses, which revealed that the prevalence outcomes obtained with the UCAA2000 in these very low prevalence settings were seven times higher than those based on a single urine filtration, and hence the sample size could be lowered to around 500 UCAA2000 tests. The UCAA250 was performed to gather new information on a different approach, which is potentially less complicated to perform in resource-constrained settings.

### Empirical *S*. *haematobium* Prevalences According to Diagnostic Approach

**Fig 2:**
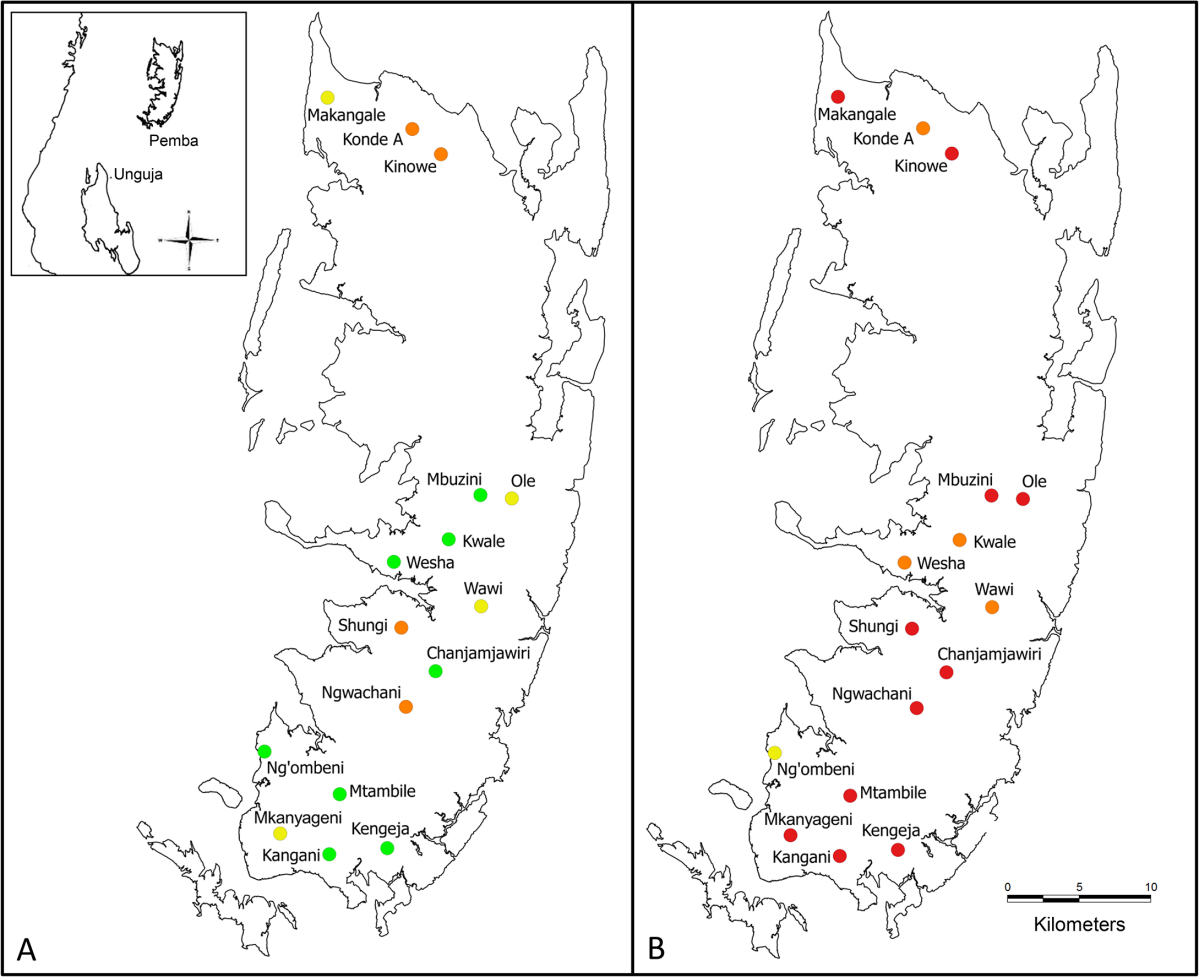
Maps indicating *Schistosoma haematobium* prevalence levels according to different diagnostic tests. The two maps indicate different *S*. *haematobium* prevalence levels as identified with a single urine filtration method (A) and a urine-based up-converting phosphor-lateral flow circulating anodic antigen (UCAA2000) assay (B) in 16 schools on Pemba island, United Republic of Tanzania, in 2013. UCAA2000: up-converting phosphor-lateral flow assay detecting circulating anodic antigen in urine and prepared with 1.5 ml of urine; green spot: school with a prevalence of <2%; yellow spot: school with a prevalence of 2–5%; orange spot: school with a prevalence of 5-<10%; red spot: school with a prevalence of ≥10%.

Fig 2 shows that there were considerable differences in the empirical *S*. *haematobium* prevalences at the unit of the school and according to endemicity level, depending on the diagnostic approach applied. The thresholds of <2%, 2–5%, and 5–10% were only met with the initial urine filtration reading, indicating an average prevalence of 1.5%, 3.4%, and 6.7% for the schools stratified to each endemicity level (Table 2). The QCUF reading revealed slightly higher average prevalences of 2.6%, 4.6% and 8.5%, respectively, and reagent strips of 1.8%, 5.8%, and 6.1%, respectively. Considerably higher prevalences for individual schools and average prevalences according to endemicity level were revealed by the UCAA2000. Considering indecisive results as negative, the UCAA2000- indicated average prevalences of 10.1%, 12.0% and 19.8%, respectively, for the three endemicity levels. Considering indecisive results as positive, the UCAA2000+ indicated average prevalences of 14.8%, 17.2% and 24.7%, respectively.

### Sensitivity Estimates of Diagnostic Methods Using an Imperfect ‘Gold’ Standard

**Fig 3:**
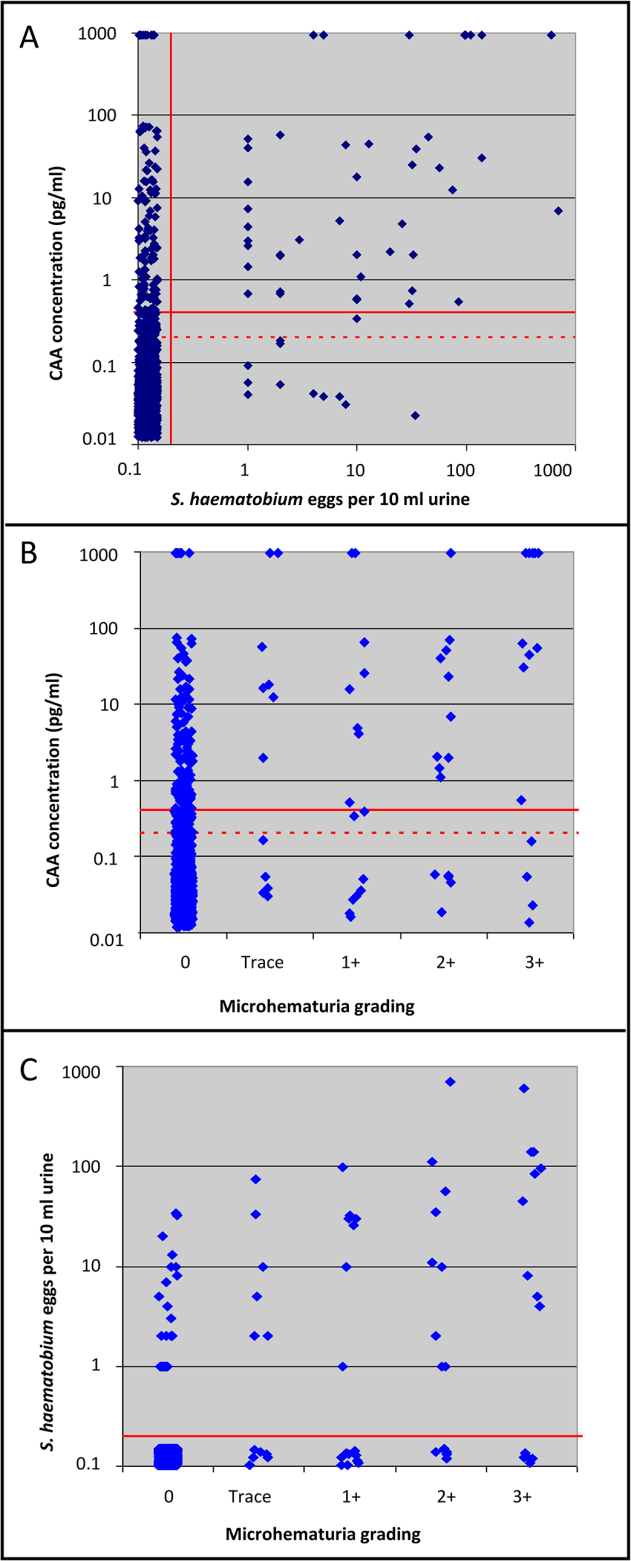
Correlation of circulating anodic antigen (CAA) levels and *S*. *haematobium* egg counts or microhematuria grading. (A) Correlation of CAA levels (pg/ml) in 1.5 ml of urine and the number of *S*. *haematobium* eggs detected in 10 ml of urine (Spearman’s rho = 0.24; P <0.001); (B) correlation of CAA levels (pg/ml) and the microhematuria grading (Spearman’s rho = 0.23; P <0.001); and (C) correlation of *S*. *haematobium* eggs detected and microhematuria grading (Spearman’s rho = 0.57; p<0.001), in urine samples from children from Pemba, United Republic of Tanzania, collected in 2013. The horizontal continuous red line indicates the cut-off value of >0.4 pg/ml for samples clearly indicated as *S*. *haematobium*-positive by the UCAA2000 (A and B). The horizontal dotted red line indicates the cut-off value of <0.2 pg/ml for samples clearly indicated as *S*. *haematobium*-negative by the UCAA2000 (A and B). Values right from the vertical continuous red line (A) and above the horizontal continuous red line (C) indicate egg-positive urine filtration tests

The numbers of positive, negative, and indecisive results for each diagnostic method combination are shown in 6-cell matrixes in Table 3. Since QCUF revealed more *S*. *haematobium*-infected individuals than the initial urine filtration microscopy, only QCUF results were considered for the calculation of sensitivity and specificity. Applying a combination of the QCUF and UCAA2000+ as imperfect diagnostic ‘gold’ standard, the UCAA2000+ had the highest overall sensitivity of 95.2%, followed by the UCAA2000- with a sensitivity of 69.4% (Table 4). The QCUF and reagent strips showed very low sensitivities (24.9% and 16.6%, respectively). While the UCAA2000+ showed stable sensitivity across the three *S*. *haematobium* prevalence settings (<2%, 2–5%, and 5–10%), a decreasing trend in sensitivity with lower prevalence was observed for the UCAA2000- and particularly for the QCUF. A considerable drop in the sensitivity of reagent strip results only occurred in the <2% prevalence setting. Changes in sensitivity were, however, not statistically significant. Noteworthy, the geometric mean egg count decreased significantly from highest to lowest prevalence settings from 0.22 eggs/10 ml urine to 0.05 eggs/10 ml urine. As shown in Fig 3, we found a significant relationship between CAA pg/ml levels and *S*. *haematobium* egg counts (Spearman’s rho = 0.24; p<0.001), between CAA pg/ml levels and microhematuria grading (Spearman’s rho = 0.23; p<0.001), and between egg counts and microhematuria grading (Spearman’s rho = 0.57; p<0.001).

**Table 3:** Agreement between the different diagnostic approaches.

	QCUF		
**UF**	Positive	Negative	Total
Positive	40	1	41
Negative	17	1,142	1,159
Total	57	1,143	1,200
	**QCUF**		
**Reagent strip**	Positive	Negative	Total
Positive	31	18	49
Negative	26	1,125	1,151
Total	57	1,143	1,200
	**QCUF**		
**UCAA2000**	Positive	Negative	Total
Positive	45	114	159
Indecisive	1	58	59
Negative	11	971	982
Total	57	1,143	1,200
	**Reagent strip**		
**UCAA2000**	Positive	Negative	Total
Positive	35	124	159
Indecisive	2	57	59
Negative	12	970	982
Total	49	1,151	1,200

Four-cell and six-cell-matrixes showing the agreement of the number of positive, negative, and indecisive results of the initial urine filtration (UF), the quality control slide reading (QCUF), the reagent strips, and the up-converting phosphor-lateral flow assay detecting circulating anodic antigen in urine (UCAA2000) methods for the diagnosis of *S*. *haematobium* in urine samples from children from 16 primary schools in Pemba, United Republic of Tanzania in 2013.

**Table 4:** Diagnostic accuracy of the tests used to detect *S*. *haematobium* infections stratified by prevalence setting.

‘Gold’ standard	Prevalence	n	GM eggs/10 ml	Test	Sensitivity % [95% CI]
Combination of QCUF and UCAA2000+ as ‘gold’ standard*	all	1,200		Reagent strip	16.6 [12.0–22.1]
			0.12	QCUF	24.9 [19.4–31.0]
				CAA2000-	69.4 [63.0–75.3]
				CAA2000+	95.2 [91.6–97.6]
Combination of QCUF and UCAA2000+ as ‘gold’ standard*	<2%	546		Reagent strip	8.3 [3.4–16.4]
			0.05 [0.02–0.09]	QCUF	16.7 [9.4–26.4]
				CAA2000-	65.5 [54.3–75.5]
				CAA2000+	96.4 [89.9–99.3]
Combination of QCUF and UCAA2000+ as ‘gold’ standard*	2–5%	326		Reagent strip	24.1 [13.9–37.2]
			0.13 [0.05–0.23]	QCUF	25.9 [15.3–39.0]
				CAA2000-	67.2 [53.7–79.0]
				CAA2000+	96.6 [88.1–99.6]
Combination of QCUF and UCAA2000+ as ‘gold’ standard*	5–10%	328		Reagent strip	19.5 [11.8–29.4]
			0.22 [0.13–0.33]	QCUF	32.2 [22.6–43.1]
				CAA2000-	74.7 [64.3–83.4]
				CAA2000+	93.1 [85.6–97.4]

Diagnostic accuracy of reagent strips, quality control urine filtration slide reading (QCUF), and the up-converting phosphor-lateral flow assay detecting circulating anodic antigen in urine methods (UCAA2000+, and UCAA2000-) for *S*. *haematobium* detection as calculated by comparison against an imperfect ‘gold’ standard (i.e., the combination of UCAA2000+ and QCUF results), stratified by prevalence setting in our study conducted in Pemba, United Republic of Tanzania in 2013.

QCUF: second reading of the urine filtration slide for quality control purposes between November 2013 and January 2014; UCP-LF CAA: up-converting phosphor-lateral flow assay detecting circulating anodic antigen in urine; UCAA2000+: UCP-LF CAA prepared with 1.5 ml of urine, indecisive results were considered as positive; UCAA2000-: UCP-LF CAA prepared with 1.5 ml of urine, indecisive results were considered as negative; UCAA250+: UCP-LF CAA prepared with 250 μl of urine, indecisive results were considered as positive; UCAA250-: UCP-LF CAA prepared with 250 μl of urine, indecisive results were considered as negative; GM: geometric mean

*: Test specificity was assumed to be 100%.

### Accuracy Estimates of Diagnostic Methods Using LCA

Statistical information criteria (i.e., AIC and BIC) indicated that no random effects were needed at the school level, suggesting that neither the diagnostic performance of reagent strip, QCUF, or UCAA2000 tests, nor the model estimated *S*. *haematobium* prevalence varied significantly between the surveyed schools. The assumption of conditional independence between the three diagnostic tests was considered as valid, since inspection of the standardized results from the final selected model (S1 Model 1) did not show extreme values (i.e., residuals for all response patterns were between -2 and 2). Furthermore, when we allowed for partial conditional independence between reagent strip and QCUF results, the model fit was not improved (S1 Model 4), which further strengthened the argument for conditional independence between the tests.

Our final LCA model (S1 Model 1, with the lowest AIC and BIC) revealed a sensitivity of 97.0% (95% CI: 90.5–100%), 85.5% (95% CI: 72.2–98.8%), and 66.7% (95% CI: 52.4–81.0%) for UCAA2000, QCUF, and reagent strip, respectively. The highest specificity was obtained for QCUF (99.1%, 95% CI: 98.5–99.7%), followed by reagent strip (98.9%, 95% CI: 98.3–99.5%), and UCAA2000 (90.1%, 95% CI: 88.3–91.9%). The model estimated *S*. *haematobium* prevalence including all schools was 4.5%.

## Discussion

Enhanced efforts to achieve the schistosomiasis control and elimination goals put forth by WHO for the years 2020 and 2025 will likely reduce the *Schistosoma* prevalence and infection intensities in targeted populations. To discover and investigate continuing transmission and to reliably confirm schistosomiasis elimination without missing very light infection intensities, diagnostic tools with high sensitivity and specificity are needed [11,19–22,41].

We assessed the accuracy of the UCAA2000 assay for *S*. *haematobium* diagnosis in low-endemicity settings on Pemba Island. Based on a single urine filtration, we selected schools with a prevalence of *S*. *haematobium* <2%, 2–5%, and 5–10%. LCA revealed an overall sensitivity and specificity of a single UCAA2000 of 97.0% and 90.1%, single QCUF of 85.5% and 99.1%, and single reagent strips of 66.7% and 98.9%, respectively, and a model estimated prevalence of 4.5%. No significant drop in the empirical sensitivity from highest to lowest investigated endemicity scenario was revealed, but we observed a clear tendency of decreasing sensitivity of particularly the QCUF and reagent strip test results with lower prevalence and geometric mean egg count levels. The overall *S*. *haematobium* prevalence empirically determined with the UCAA2000+, UCAA2000-, urine filtration, and reagent strips were 18.2%, 13.3%, 4.8%, and 4.1%, respectively.

Our results show that empirically, a single UCAA2000 test detects a considerably higher *S*. *haematobium* prevalence than microscopy or reagent strips. Even if indecisive results obtained with the UCAA2000 were considered as negative, the overall *S*. *haematobium* prevalence was almost three times higher than that elucidated by QCUF. Particularly evident were the differences in the lowest endemicity setting, where UCAA2000+ and UCAA2000- revealed prevalences of 14.8% and 10.1%, respectively, while QCUF and reagent strips found considerably lower prevalences of 2.6% and 1.8%, respectively. If indeed correct, this finding would have important ramifications for the schistosomiasis elimination program in Zanzibar. Since egg output and microhematuria were reasonably low, according to the current definitions, elimination of schistosomiasis as a public health problem had been reached and the setting was on the way toward interruption of transmission. The UCAA2000 revealed, however that more than 10% of the surveyed population excreted CAA and thus harbored living worms, which potentially could produce eggs at some point passed in urine. Given these persistent very low intensity infections in the presence of ongoing control interventions and the potential for disease recrudescence or the parasite’s reintroduction into parasite-free environments also by very modest external inputs, the situation would require an adequate response in terms of more effective locally targeted control strategies [42].

The model estimated overall *S*. *haematobium* prevalence, in contrast, was only 4.5%, taking into account an imperfect specificity of the UCAA2000, which was estimated by LCA at 90.1%. Considering this imperfect specificity, a considerable amount of *S*. *haematobium* cases detected by the UCAA2000 might have been false-positives. However, a specificity of 90.1% is below the specificity of circulating antigen assays and the UCP-LF CAA test as postulated elsewhere [27,29,43,44]. Moreover, studies with the UCAA2000 in non-endemic African settings using samples from previous studies, banked at Leiden University Medical Center revealed that no false-positives were detected by the method. One also has to note that CAA is released by living worms that might or might not produce eggs. In case children were reached by the latest MDA conducted in November 2012 and knowing that CAA clears within a few days or weeks after successful treatment [45, 46], it might be that the CAA-positive results in the egg-negative urines collected between March and May 2013 indicate worms that survived but were sterilized by the previous praziquantel treatment, or worms that were schistosomula at the time of treatment and not affected by praziquantel, or new infections with schistosomes that were not yet producing eggs. Since we were working in an elimination setting where transmission is mostly low, it might also be that CAA-positive but egg-negative individuals were infected with single worms rather than worm pairs, and hence no eggs were produced. The sensitivity of the UCAA2000 of 97.0% determined with the LCA and of the UCAA2000+ of 95.2% determined with an imperfect ‘gold’ standard in our study, is in line with findings from the People’s Republic of China, where the UCAA2000+ sensitivity for *S*. *japonicum* detection was 93% [29].

A limitation of our study and approaches to estimate sensitivity and specificity is that the UCAA2000 was only compared to two diagnostic techniques with a limited accuracy, particularly for examining samples from a low-endemic area and when only a single urine sample was examined. Clearly, the UCAA2000 has a very high sensitivity and a sufficient specificity to serve as a tool for diagnosing urogenital schistosomiasis in low-endemicity settings targeting elimination. Whether its specificity is high enough to deserve also the title “confirmation of elimination tool” remains to be elucidated in future studies comparing its performance not only with microscopy and reagent strips but also with more accurate methods, for example with polymerase chain reaction (PCR) [47], in a similar close-to-elimination setting.

The considerable number of “potentially positive” individuals determined by “indecisive” UCAA2000 results is another limitation of our study. Indecisive results appeared due to the selection of a higher specificity and a lower specificity cut-off. As described in Corstjens *et al*. (2014), the cut-off thresholds may be influenced by technical factors such as batch-to-batch variation, as well as (immuno-)epidemiological settings (e.g., co-infections, age, and geography) [27]. In the current setting, suboptimal sample volume and centrifugation capacities were considered the main reason for the need of a higher cut-off threshold. Moreover, the definition of a precise cut-off for any UCP-LF CAA approach is pending and can only be developed for large reagent strip batches and by testing a large number of clearly uninfected people from endemic and non-endemic settings. Currently, to obtain a clear result for potentially positive individuals, their urine sample would either need to be repeatedly tested with the UCAA2000, or larger amounts of their urines would need to be examined, for example with the UCAA7500, which has a lower detection limit of 0.03 pg/ml [27], or their CAA level could be retested after praziquantel treatment to investigate whether it decreased.

It is worth mentioning that a rigorous second examination of the urine filtration slides by an experienced slide reader (i.e., QCUF) revealed a higher number of *S*. *haematobium* egg-positive slides than the initial urine filtration reading of the same slides by local technicians. This finding confirms that (i) the glycerol soaked cellophane cover method we used to preserve our urine filtration slides for more than 6 months for quality control purposes works; and (ii) that conducting quality control of urine filtration slides is essential to achieve more reliable results for studies on diagnostic accuracy as well as for prevalence estimates in schistosomiasis control and elimination programs.

Our study shows that the UCAA2000 is a highly sensitive diagnostic tool that is able to diagnose *S*. *haematobium* infections in very low endemicity settings. The dry format allows convenient transport of dry reagents without a cold chain to third-party laboratories [27]. The assay can be implemented by trained local technicians in laboratories in endemic settings, given they are adequately equipped such as the PHL-IdC in Pemba. When sufficient centrifugation capacities and a UCP-Quant reader are available, up to 100 samples can be processed by one technician per day, and hence, the test has a higher throughput than parasitological approaches requiring microscopy. However, in the current format, the UCAA2000 cannot be applied in field laboratories without centrifugation and pipetting capacities.

Moreover, the costs for a single UCAA2000 are high and the test is not commercialized. Hence, at the time being, this test is out of reach for most control programs and large-scale studies in endemic areas, but is only used in collaborative projects. Efforts to develop a simple-to-use but still highly sensitive point-of-care (POC)-CAA rapid test for commercialization at an affordable price are underway.

One also has to consider that the UCAA2000 is a highly sensitive test to reflect active infection, but that it does not encapsulate morbidity. In low endemic settings, where the primary aim is to assess transmission, this test holds particular potential. For indicating morbidity caused by urogenital schistosomiasis, rapid tests such as reagent strips showing the grade of hematuria, and detecting proteinuria and leukocyturia or urine albumin and creatinine have clear advantages [22,48,49].

Once standardized, commercialized, and widely available at reasonable costs, we consider the UCAA2000 as a suitable tool for large-scale monitoring of urogenital schistosomiasis in control programs in low-endemicity settings targeting elimination and for surveillance in areas that achieved elimination. For surveillance at a smaller scale, including testing of suspected cases in remote public health care centres without laboratory equipment, a simple-to-use but still highly sensitive POC-CAA rapid test is highly desirable.

## Supporting Information

S1 Checklist STARD checklist. (PDF)

S1 Data Dataset used for analysis of the data presented in this manuscript. (PDF)

S1 Models Latent class analysis (LCA) modeling approaches for sensitivity and specificity estimation. (PDF)

S1 Translation Translation of abstract into Dutch, French, and German. (PDF)
