# Supplementary material for: Sensitivity and Specificity of a Urine Circulating Anodic Antigen Test for the Diagnosis of Schistosoma haematobium in Low Endemic Settings
Source: PLoS Negl Trop Dis. 2015 May 14;9(5):e0003752. doi: 10.1371/journal.pntd.0003752 (PMC4431728; doi:10.1371/journal.pntd.0003752)
Supplement: S1 Checklist — (PDF) [file pntd.0003752.s001.pdf]

STARD checklist for reporting of studies of diagnostic accuracy
(version January 2003)

Section and Topic	Item #		On page #	Text
TITLE/ABSTRACT/ KEYWORDS	1	Identify the article as a study of diagnostic accuracy (recommend MeSH heading 'sensitivity and specificity').	1	Sensitivity and Specificity of a Urine Circulating Anodic Antigen Test for the Diagnosis of *Schistosoma haematobium* in Low Endemic Settings
INTRODUCTION	2	State the research questions or study aims, such as estimating diagnostic accuracy or comparing accuracy between tests or across participant groups.	8	Here, we assess the accuracy of the UCAA2000 for the diagnosis of *S. haematobium* in three low-prevalence scenarios (<2%, 2-5%, and 5-10%), as determined with a single urine filtration. In the absence of a true “gold” standard, sensitivity and specificity were determined empirically and by means of latent class analysis (LCA).
METHODS				
Participants	3	The study population: The inclusion and exclusion criteria, setting and locations where data were collected.	10	The urine samples used for the diagnostic investigations presented here were collected from children aged 9-12 years visiting primary schools in 16 shehias on Pemba Island between March and May 2013.

	4	Participant recruitment: Was recruitment based on presenting symptoms, results from previous tests, or the fact that the participants had received the index tests or the reference standard?	13,14	The selection of shehias with *S. haematobium* prevalences of <2%, 2-5%, and 5-10% for inclusion into the present study was based on results of the initial urine filtration examination performed on the day of sample collection and including all children with written informed consent, microhematuria, and urine filtration results. For assessing diagnostic accuracy, however, we only included data from individuals with complete diagnostic results on (i) reagent strip testing; (ii) urine filtration reading; (iii) UCAA2000 testing (considering indecisive results either as positive (UCAA2000+) or as negative (UCAA2000-) or as missing); and (iv) QCUF reading into the final analysis. While urine samples stored for UCP-LF CAA examination were not selected at full random (i.e., only urine samples of sufficient amount of the first 100 among 130 collected samples per school were stored), we yet considered this approach as valid and assumed complete randomness of missing samples (and that missing values are unrelated to the status of *S. haematobium* infection), since the overall percentage of positive individuals detected by the initial urine filtration did not differ between the initially sampled group (3.3%; Table 1) and the group included into the final analysis (3.4%; Table 2).

	5	Participant sampling: Was the study population a consecutive series of participants defined by the selection criteria in item 3 and 4? If not, specify how participants were further selected.	15, Figure 1	To meet the prevalence thresholds and sample size for the study, we selected eight primary schools with a prevalence of *S. haematobium* <2%, four schools with a prevalence of 2-5%, and four schools with a prevalence of 5-10% based on single urine filtration readings per child. Overall, from the 16 selected schools, 2,067 children were randomly selected to participate in the annual parasitological survey in 2013. Among them, 298 did not provide written informed consent from their parents and were therefore not asked to submit a urine sample (Figure 1). An additional 29 children did not submit a urine sample of sufficient amount to perform reagent strip and urine filtration examinations. Hence, the initial *S. haematobium* prevalence at the unit of the school was calculated from urine filtration results of 1,740 children. Table 1 shows the baseline results, stratified by school and prevalence setting. UCP-LF CAA and QCUF readings were available from 1,284 children. The UCAA2000 and UCAA250 were applied on 1,200 and 84 urine samples, respectively.

	6	Data collection: Was data collection planned before the index test and reference standard were performed (prospective study) or after (retrospective study)?	11, 12	At the day of collection, all urine samples of sufficient amount (at least 10 ml) were examined by trained laboratory technicians for microhematuria using reagent strips (Hemastix; *Siemens* Healthcare Diagnostics GmbH, Eschborn, Germany), and for the presence and number of eggs detected under a microscope using the urine filtration method with polycarbonate filters (Sterlitech, Kent, WA, USA). All urine filters were covered with hydrophilic cellophane soaked in glycerol solution and the slides were stored for a potential second reading for quality control. At the day of collection, before reagent strip and urine filtration were performed, an amount of 1.8 ml urine was frozen and stored at -20℃ from children with IDs 1-100 from each shehia for future examinations. The frozen samples from children from the 16 shehias selected for this study were examined with the UCAA2000 or UCAA250 assays in November 2013 at PHL-IdC.<br><br>The stored urine filtration slides from all individuals, whose urines were examined with a UCP-LF CAA test, were retrospectively re-read between November 2013 and January 2014 by a post-doctoral fellow (CIC) blinded to the reagent strip, initial urine filtration, and UCP-LF CAA results.

Test methods	7	The reference standard and its rationale.		Diagnostic accuracy parameters including 95% confidence intervals (CIs) were assessed using two different approaches. In the first approach, we considered the combined results of QCUF and UCAA2000+ as imperfect "gold" standard and calculated the sensitivity of each test by comparing its performance against the imperfect "gold" standard. Assuming a specificity of 100%, the sensitivity of all diagnostic tests was calculated for (i) combined data from all individuals included into the final analysis and (ii) stratified data according to the originally selected different prevalence levels (<2%, 2-5%, and 5-10%). To assess a correlation between CAA pg/ml levels and the number of eggs detected in 10 ml urines or microhematuria grading identified with reagent strips, we applied the non-parametric Spearman's rank correlation test.<br>In the second approach, in the absence of a true "gold" standard, we used LCA to estimate the sensitivity, specificity, and model estimated prevalences for reagent strip, QCUF, and UCAA2000 [35-37]. Four LCA models were applied and validated. The exact procedure is presented in supplementary file 1 (S1) and model details have been described by Ibironke and colleagues (2012) [36]. The four LCA models were fitted using MPlus V7 [34] with full information maximum likelihood estimation and assuming that data were missing at random. We included the indecisive results of the UCAA2000 in all LCA models by considering them as 'missing' and not forcing them in a positive or negative category [38]. The four LCA models were evaluated according to the lowest Bayesian information criterion (BIC) and Akaike information criterion (AIC) as indications of the best model fit and parsimony in combination with different biological plausible scenarios and tests of assumptions. Below, we present results from LCA model 1 (S1: Table S1, Model 1).

	8	Technical specifications of material and methods involved including how and when measurements were taken, and/or cite references for index tests and reference standard.	11,12	Please see full chapter of Laboratory Procedures
	9	Definition of and rationale for the units, cut-offs and/or categories of the results of the index tests and the reference standard.	13	Microhematuria was graded into negative, trace, 1+, 2+, and 3+ according to the color chart provided by the manufacturer. *S. haematobium* egg numbers were recorded per 10 ml of urine. The concentration of CAA in urine was calculated using standard curves derived from daily freshly prepared concentration series of partly purified antigen and expressed as pg/ml. High and low specificity cut-offs were determined as described elsewhere [23,26]. A sample was considered positive at CAA values of >0.4 pg/ml, as indecisive at 0.2-0.4 pg/ml, and as negative at <0.2 pg/ml for the UCAA2000 assay. Samples tested with the UCAA250 were considered as positive at CAA levels of >1.4 pg/ml, indecisive at 0.7-1.4 pg/ml, and as negative at <0.7 pg/ml. Of note, applied cut-off values are slightly different from those described by Corstjens et al. 2014 [26], and directly related to the (slightly smaller) sample volume input and the concentration factor obtained with the Amicon concentration devices.

	10	The number, training and expertise of the persons executing and reading the index tests and the reference standard.	11	At the day of collection, all urine samples of sufficient amount (at least 10 ml) were examined by trained laboratory technicians for microhematuria using reagent strips (Hemastix; Siemens Healthcare Diagnostics GmbH, Eschborn, Germany), and for the presence and number of eggs detected under a microscope using the urine filtration method with polycarbonate filters (Sterlitech, Kent, WA, USA).<br><br>Four laboratory technicians received an in-depth training in the preparation of samples and conduction of the UCAA2000 and UCAA250 by two of the authors (GJvD and PLAMC) at PHL-IdC. Supervised by, and in collaboration with a trained post-doctoral fellow (CIC), the technicians examined the samples as described elsewhere [25,26] blinded to the reagent strip and initial urine filtration reading results.
	11	Whether or not the readers of the index tests and reference standard were blind (masked) to the results of the other test and describe any other clinical information available to the readers.	11,12	Supervised by, and in collaboration with a trained post-doctoral fellow (CIC), the technicians examined the samples as described elsewhere [25,26] blinded to the reagent strip and initial urine filtration reading results.<br><br>The stored urine filtration slides from all individuals, whose urines were examined with a UCP-LF CAA test, were retrospectively re-read between November 2013 and January 2014 by a post-doctoral fellow (CIC) blinded to the reagent strip, initial urine filtration, and UCP-LF CAA results.

Statistical methods	12	Methods for calculating or comparing measures of diagnostic accuracy, and the statistical methods used to quantify uncertainty (e.g. 95% confidence intervals).	11,12,13,	While urine samples stored for UCP-LF CAA examination were not selected at full random (i.e., only urine samples of sufficient amount of the first 100 among 130 collected samples per school were stored), we yet considered this approach as valid and assumed complete randomness of missing samples (and that missing values are unrelated to the status of *S. haematobium* infection), since the overall percentage of positive individuals detected by the initial urine filtration did not differ between the initially sampled group (3.3%; Table 1) and the group included into the final analysis (3.4%; Table 2).<br><br>From this subsample, we calculated 'empirical' prevalences obtained by each diagnostic method assuming a 100% test specificity. Diagnostic accuracy parameters including 95% confidence intervals (CIs) were assessed using two different approaches. In the first approach, we considered the combined results of QCUF and UCAA2000+ as imperfect "gold" standard and calculated the sensitivity of each test by comparing its performance against the imperfect "gold" standard. Assuming a specificity of 100%, the sensitivity of all diagnostic tests was calculated for (i) combined data from all individuals included into the final analysis and (ii) stratified data according to the originally selected different prevalence levels (<2%, 2-5%, and 5-10%). To assess a correlation between CAA pg/ml levels and the number of eggs detected in 10 ml urines or microhematuria grading identified with reagent strips, we applied the non-parametric Spearman's rank correlation test.<br><br>In the second approach, in the absence of a true "gold" standard, we used LCA to estimate the sensitivity, specificity, and model estimated prevalences for reagent strip, QCUF, and UCAA2000 [35-37]. Four LCA models were applied and validated. The exact procedure is presented in supplementary file 1 (S1) and model details have been described by Ibironke and colleagues (2012) [36]. The four LCA models were fitted using MPlus V7 [34] with full information maximum likelihood estimation and assuming that data were missing at random. We included the indecisive results of the UCAA2000 in all LCA [illegible]

	13	Methods for calculating test reproducibility, if done.		Not done
RESULTS				
Participants	14	When study was performed, including beginning and end dates of recruitment.	11,12	At the day of collection, between March and May 2013, all urine samples of sufficient amount (at least 10 ml) were examined by trained laboratory technicians for microhematuria using reagent strips (Hemastix; Siemens Healthcare Diagnostics GmbH, Eschborn, Germany), and for the presence and number of eggs detected under a microscope using the urine filtration method with polycarbonate filters (Sterlitech, Kent, WA, USA).<br><br>The frozen samples from children from the 16 shehias selected for this study were examined with the UCAA2000 or UCAA250 assays in November 2013 at PHL-IdC.<br><br>The stored urine filtration slides from all individuals, whose urines were examined with a UCP-LF CAA test, were retrospectively re-read between November 2013 and January 2014 by a post-doctoral fellow (CIC) blinded to the reagent strip, initial urine filtration, and UCP-LF CAA results.
	15	Clinical and demographic characteristics of the study population (at least information on age, gender, spectrum of presenting symptoms).	Table 1	Please see Table 1
	16	The number of participants satisfying the criteria for inclusion who did or did not undergo the index tests and/or the reference standard; describe why participants failed to undergo either test (a flow diagram is strongly recommended).	Figure 1	Please see Figure 1

Test results	17	Time-interval between the index tests and the reference standard, and any treatment administered in between.	11	At the day of collection, between March and May 2013, all urine samples of sufficient amount (at least 10 ml) were examined by trained laboratory technicians for microhematuria using reagent strips (Hemastix; Siemens Healthcare Diagnostics GmbH, Eschborn, Germany), and for the presence and number of eggs detected under a microscope using the urine filtration method with polycarbonate filters (Sterlitech, Kent, WA, USA).<br><br>At the day of collection, before reagent strip and urine filtration were performed, an amount of 1.8 ml urine was frozen and stored at -20°C from children with IDs 1-100 from each shehia for future examinations. The frozen samples from children from the 16 shehias selected for this study were examined with the UCAA2000 or UCAA250 assays in November 2013 at PHL-IdC.<br><br>All tests were done from the same urine samples, hence no treatment was given between sample collection
	18	Distribution of severity of disease (define criteria) in those with the target condition; other diagnoses in participants without the target condition.	16, Table 4	Noteworthy, the geometric mean egg count decreased significantly from highest to lowest prevalence settings from 0.22 eggs/10 ml urine to 0.05 eggs/10 ml urine.
	19	A cross tabulation of the results of the index tests (including indeterminate and missing results) by the results of the reference standard; for continuous results, the distribution of the test results by the results of the reference standard.	Table 3	Please see Table 3
	20	Any adverse events from performing the index tests or the reference standard.	NA	The test was performed on urine; no adverse events occur from urine collection.

Estimates	21	Estimates of diagnostic accuracy and measures of statistical uncertainty (e.g. 95% confidence intervals).	16, 17 Table 4,	Applying a combination of the QCUF and UCAA2000+ as imperfect diagnostic “gold” standard, the UCAA2000+ had the highest overall sensitivity of 95.2%, followed by the UCAA2000- with a sensitivity of 69.4% (Table 4). The QCUF and reagent strips showed very low sensitivities (24.9% and 16.6%, respectively).<br><br>Our final LCA model (S1: Model 1, with the lowest AIC and BIC) revealed a sensitivity of 97.0% (95% CI: 90.5-100%), 85.5% (95% CI: 72.2-98.8%), and 66.7% (95% CI: 52.4-81.0%) for UCAA2000, QCUF, and reagent strip, respectively. The highest specificity was obtained for QCUF (99.1%, 95% CI: 98.5-99.7%), followed by reagent strip (98.9%, 95% CI: 98.3-99.5%), and UCAA2000 (90.1%, 95% CI: 88.3-91.9%). The model estimated *S. haematobium* prevalence including all schools was 4.5%.

	22	How indeterminate results, missing data and outliers of the index tests were handled.	14,15,17,S3	In the second approach, in the absence of a true “gold” standard, we used LCA to estimate the sensitivity, specificity, and model estimated prevalences for reagent strip, QCUF, and UCAA2000 [35-37]. Four LCA models were applied and validated. The exact procedure is presented in supplementary file 1 (S1) and model details have been described by Ibironke and colleagues (2012) [36]. The four LCA models were fitted using MPlus V7 [34] with full information maximum likelihood estimation and assuming that data were missing at random. We included the indecisive results of the UCAA2000 in all LCA models by considering them as ‘missing’ and not forcing them in a positive or negative category [38]. The four LCA models were evaluated according to the lowest Bayesian information criterion (BIC) and Akaike information criterion (AIC) as indications of the best model fit and parsimony in combination with different biological plausible scenarios and tests of assumptions. Below, we present results from LCA model 1 (S1: Table S1, Model 1).<br><br>The assumption of conditional independence between the three diagnostic tests was regarded as valid, since inspection of the standardized results from the final selected model (S1: Model 1) did not show extreme values (i.e., residuals for all response patterns were between -2 and 2).
	23	Estimates of variability of diagnostic accuracy between subgroups of participants, readers or centers, if done.	16, Table 5	A considerable drop in the sensitivity of reagent strip results only occurred in the <2% prevalence setting. Changes in sensitivity were, however, not statistically significant.
	24	Estimates of test reproducibility, if done.		Not done

DISCUSSION	25	Discuss the clinical applicability of the study findings.	21	Our study shows that the UCAA2000 is a highly sensitive and specific diagnostic tool that is able to diagnose *S. haematobium* infections reliably in very low endemicity settings. The dry format allows convenient transport of dry reagents without a cold chain to third-party laboratories [26]. The assay can be implemented by trained local technicians in laboratories in endemic settings, given they are adequately equipped such as the PHL-IdC in Pemba. When sufficient centrifugation capacities and a UCP-Quant reader are available, up to 100 samples can be processed by one technician per day, and hence, the test has a much higher throughput potential than parasitological approaches requiring microscopy. Since large sample sizes can be screened with a very high sensitivity, we consider the UCAA2000 as a suitable tool for large-scale monitoring of urogenital schistosomiasis in control programs in low-endemicity settings targeting elimination and for surveillance in areas that achieved elimination. For surveillance at a smaller scale, including testing of suspected cases in remote public health care centres without laboratory equipment, a simple to use but still highly sensitive point-of-care CAA rapid test is highly desirable.
