# Supplementary material for: Sensitivity and Specificity of a Urine Circulating Anodic Antigen Test for the Diagnosis of Schistosoma haematobium in Low Endemic Settings
Source: PLoS Negl Trop Dis. 2015 May 14;9(5):e0003752. doi: 10.1371/journal.pntd.0003752 (PMC4431728; doi:10.1371/journal.pntd.0003752)
Supplement: S1 Data — (PDF) [file pntd.0003752.s002.pdf]

| Isl | Year | District | Date | School | ID | Sex | Age | Standard1 |
|---|---|---|---|---|---|---|---|---|
| Pemba | 2013 | Chake Chake | 16/4/2013 | KWALE | 02001 | F | 10 | 4 |
| Pemba | 2013 | Chake Chake | 16/4/2013 | KWALE | 02002 | F | 10 | 4 |
| Pemba | 2013 | Chake Chake | 16/4/2013 | KWALE | 02003 | F | 10 | 4 |
| Pemba | 2013 | Chake Chake | 16/4/2013 | KWALE | 02004 | F | 10 | 4 |
| Pemba | 2013 | Chake Chake | 16/4/2013 | KWALE | 02005 | F | 10 | 4 |
| Pemba | 2013 | Chake Chake | 16/4/2013 | KWALE | 02006 | F | 11 | 4 |
| Pemba | 2013 | Chake Chake | 16/4/2013 | KWALE | 02007 | F | 11 | 4 |
| Pemba | 2013 | Chake Chake | 16/4/2013 | KWALE | 02008 | F | 11 | 4 |
| Pemba | 2013 | Chake Chake | 16/4/2013 | KWALE | 02009 | F | 11 | 4 |
| Pemba | 2013 | Chake Chake | 16/4/2013 | KWALE | 02010 | F | 11 | 4 |
| Pemba | 2013 | Chake Chake | 16/4/2013 | KWALE | 02011 | F | 12 | 4 |
| Pemba | 2013 | Chake Chake | 16/4/2013 | KWALE | 02012 | F | 12 | 4 |
| Pemba | 2013 | Chake Chake | 16/4/2013 | KWALE | 02013 | F | 11 | 4 |
| Pemba | 2013 | Chake Chake | 16/4/2013 | KWALE | 02014 | F | 10 | 4 |
| Pemba | 2013 | Chake Chake | 16/4/2013 | KWALE | 02015 | F | 12 | 4 |
| Pemba | 2013 | Chake Chake | 16/4/2013 | KWALE | 02016 | F | 10 | 4 |
| Pemba | 2013 | Chake Chake | 16/4/2013 | KWALE | 02017 | F | 11 | 4 |
| Pemba | 2013 | Chake Chake | 16/4/2013 | KWALE | 02018 | F | 10 | 4 |
| Pemba | 2013 | Chake Chake | 16/4/2013 | KWALE | 02019 | F | 10 | 4 |
| Pemba | 2013 | Chake Chake | 16/4/2013 | KWALE | 02020 | F | 10 | 4 |
| Pemba | 2013 | Chake Chake | 16/4/2013 | KWALE | 02021 | F | 11 | 4 |
| Pemba | 2013 | Chake Chake | 16/4/2013 | KWALE | 02022 | F | 12 | 4 |
| Pemba | 2013 | Chake Chake | 16/4/2013 | KWALE | 02023 | F | 11 | 4 |
| Pemba | 2013 | Chake Chake | 16/4/2013 | KWALE | 02024 | F | 12 | 4 |
| Pemba | 2013 | Chake Chake | 16/4/2013 | KWALE | 02025 | F | 11 | 4 |
| Pemba | 2013 | Chake Chake | 16/4/2013 | KWALE | 02026 | F | 10 | 4 |
| Pemba | 2013 | Chake Chake | 16/4/2013 | KWALE | 02027 | F | 11 | 4 |
| Pemba | 2013 | Chake Chake | 16/4/2013 | KWALE | 02028 | F | 10 | 4 |
| Pemba | 2013 | Chake Chake | 16/4/2013 | KWALE | 02029 | F | 12 | 4 |
| Pemba | 2013 | Chake Chake | 16/4/2013 | KWALE | 02030 | F | 11 | 4 |
| Pemba | 2013 | Chake Chake | 16/4/2013 | KWALE | 02031 | F | 11 | 4 |
| Pemba | 2013 | Chake Chake | 16/4/2013 | KWALE | 02032 | F | 12 | 4 |
| Pemba | 2013 | Chake Chake | 16/4/2013 | KWALE | 02033 | F | 11 | 4 |
| Pemba | 2013 | Chake Chake | 16/4/2013 | KWALE | 02034 | M | 12 | 4 |
| Pemba | 2013 | Chake Chake | 16/4/2013 | KWALE | 02035 | M | 12 | 4 |
| Pemba | 2013 | Chake Chake | 16/4/2013 | KWALE | 02036 | M | 10 | 4 |
| Pemba | 2013 | Chake Chake | 16/4/2013 | KWALE | 02037 | M | 10 | 4 |
| Pemba | 2013 | Chake Chake | 16/4/2013 | KWALE | 02038 | M | 11 | 4 |
| Pemba | 2013 | Chake Chake | 16/4/2013 | KWALE | 02039 | M | 11 | 4 |
| Pemba | 2013 | Chake Chake | 16/4/2013 | KWALE | 02040 | M | 11 | 4 |
| Pemba | 2013 | Chake Chake | 16/4/2013 | KWALE | 02041 | M | 12 | 4 |
| Pemba | 2013 | Chake Chake | 16/4/2013 | KWALE | 02042 | M | 12 | 4 |
| Pemba | 2013 | Chake Chake | 16/4/2013 | KWALE | 02043 | M | 11 | 4 |
| Pemba | 2013 | Chake Chake | 16/4/2013 | KWALE | 02044 | M | 11 | 4 |
| Pemba | 2013 | Chake Chake | 16/4/2013 | KWALE | 02045 | M | 12 | 4 |
| Pemba | 2013 | Chake Chake | 16/4/2013 | KWALE | 02046 | M | 11 | 4 |
| Pemba | 2013 | Chake Chake | 16/4/2013 | KWALE | 02047 | M | 12 | 4 |
| Pemba | 2013 | Chake Chake | 16/4/2013 | KWALE | 02048 | M | 12 | 4 |
| Pemba | 2013 | Chake Chake | 16/4/2013 | KWALE | 02049 | M | 11 | 4 |

| | | | | | | | | |
|---|---|---|---|---|---|---|---|---|
| Pemba | 2013 | Chake Chake | 16/4/2013 | KWALE | 02050 | M | 12 | 4 |
| Pemba | 2013 | Chake Chake | 16/4/2013 | KWALE | 02051 | M | 12 | 4 |
| Pemba | 2013 | Chake Chake | 16/4/2013 | KWALE | 02052 | M | 11 | 4 |
| Pemba | 2013 | Chake Chake | 16/4/2013 | KWALE | 02053 | M | 12 | 4 |
| Pemba | 2013 | Chake Chake | 16/4/2013 | KWALE | 02054 | M | 12 | 4 |
| Pemba | 2013 | Chake Chake | 16/4/2013 | KWALE | 02055 | M | 10 | 4 |
| Pemba | 2013 | Chake Chake | 16/4/2013 | KWALE | 02056 | M | 12 | 4 |
| Pemba | 2013 | Chake Chake | 16/4/2013 | KWALE | 02057 | M | 12 | 4 |
| Pemba | 2013 | Chake Chake | 16/4/2013 | KWALE | 02058 | M | 11 | 4 |
| Pemba | 2013 | Chake Chake | 16/4/2013 | KWALE | 02059 | M | 11 | 4 |
| Pemba | 2013 | Chake Chake | 16/4/2013 | KWALE | 02060 | M | 12 | 4 |
| Pemba | 2013 | Chake Chake | 16/4/2013 | KWALE | 02061 | M | 11 | 4 |
| Pemba | 2013 | Chake Chake | 16/4/2013 | KWALE | 02062 | M | 11 | 4 |
| Pemba | 2013 | Chake Chake | 16/4/2013 | KWALE | 02063 | M | 11 | 4 |
| Pemba | 2013 | Chake Chake | 16/4/2013 | KWALE | 02064 | M | 11 | 4 |
| Pemba | 2013 | Chake Chake | 16/4/2013 | KWALE | 02065 | M | 12 | 4 |
| Pemba | 2013 | Chake Chake | 16/4/2013 | KWALE | 02066 | M | 10 | 3 |
| Pemba | 2013 | Chake Chake | 16/4/2013 | KWALE | 02067 | F | 9 | 3 |
| Pemba | 2013 | Chake Chake | 16/4/2013 | KWALE | 02068 | F | 10 | 3 |
| Pemba | 2013 | Chake Chake | 16/4/2013 | KWALE | 02069 | F | 9 | 3 |
| Pemba | 2013 | Chake Chake | 16/4/2013 | KWALE | 02070 | F | 9 | 3 |
| Pemba | 2013 | Chake Chake | 16/4/2013 | KWALE | 02071 | F | 10 | 3 |
| Pemba | 2013 | Chake Chake | 16/4/2013 | KWALE | 02072 | F | 10 | 3 |
| Pemba | 2013 | Chake Chake | 16/4/2013 | KWALE | 02073 | F | 9 | 3 |
| Pemba | 2013 | Chake Chake | 16/4/2013 | KWALE | 02074 | F | 10 | 3 |
| Pemba | 2013 | Chake Chake | 16/4/2013 | KWALE | 02075 | F | 10 | 3 |
| Pemba | 2013 | Chake Chake | 16/4/2013 | KWALE | 02076 | F | 10 | 3 |
| Pemba | 2013 | Chake Chake | 16/4/2013 | KWALE | 02077 | F | 9 | 3 |
| Pemba | 2013 | Chake Chake | 16/4/2013 | KWALE | 02078 | F | 9 | 3 |
| Pemba | 2013 | Chake Chake | 16/4/2013 | KWALE | 02079 | F | 10 | 3 |
| Pemba | 2013 | Chake Chake | 16/4/2013 | KWALE | 02080 | F | 10 | 3 |
| Pemba | 2013 | Chake Chake | 16/4/2013 | KWALE | 02081 | F | 9 | 3 |
| Pemba | 2013 | Chake Chake | 16/4/2013 | KWALE | 02082 | F | 9 | 3 |
| Pemba | 2013 | Chake Chake | 16/4/2013 | KWALE | 02083 | F | 9 | 3 |
| Pemba | 2013 | Chake Chake | 16/4/2013 | KWALE | 02084 | F | 10 | 3 |
| Pemba | 2013 | Chake Chake | 16/4/2013 | KWALE | 02085 | F | 10 | 3 |
| Pemba | 2013 | Chake Chake | 16/4/2013 | KWALE | 02086 | F | 11 | 3 |
| Pemba | 2013 | Chake Chake | 16/4/2013 | KWALE | 02087 | F | 9 | 3 |
| Pemba | 2013 | Chake Chake | 16/4/2013 | KWALE | 02088 | F | 10 | 3 |
| Pemba | 2013 | Chake Chake | 16/4/2013 | KWALE | 02089 | F | 10 | 3 |
| Pemba | 2013 | Chake Chake | 16/4/2013 | KWALE | 02090 | F | 10 | 3 |
| Pemba | 2013 | Chake Chake | 16/4/2013 | KWALE | 02091 | F | 9 | 3 |
| Pemba | 2013 | Chake Chake | 16/4/2013 | KWALE | 02092 | F | 10 | 3 |
| Pemba | 2013 | Chake Chake | 16/4/2013 | KWALE | 02093 | F | 11 | 3 |
| Pemba | 2013 | Chake Chake | 16/4/2013 | KWALE | 02094 | F | 10 | 3 |
| Pemba | 2013 | Chake Chake | 16/4/2013 | KWALE | 02095 | F | 10 | 3 |
| Pemba | 2013 | Chake Chake | 16/4/2013 | KWALE | 02096 | F | 10 | 3 |
| Pemba | 2013 | Chake Chake | 16/4/2013 | KWALE | 02097 | F | 10 | 3 |
| Pemba | 2013 | Chake Chake | 16/4/2013 | KWALE | 02098 | F | 10 | 3 |
| Pemba | 2013 | Chake Chake | 16/4/2013 | KWALE | 02099 | M | 10 | 3 |

| | | | | | | | | |
|---|---|---|---|---|---|---|---|---|
| Pemba | 2013 | Chake Chake | 16/4/2013 | KWALE | 02100 | M | 10 | 3 |
| Pemba | 2013 | Chake Chake | 16/4/2013 | KWALE | 02101 | M | 12 | 3 |
| Pemba | 2013 | Chake Chake | 16/4/2013 | KWALE | 02102 | M | 11 | 3 |
| Pemba | 2013 | Chake Chake | 16/4/2013 | KWALE | 02103 | M | 11 | 3 |
| Pemba | 2013 | Chake Chake | 16/4/2013 | KWALE | 02104 | M | 10 | 3 |
| Pemba | 2013 | Chake Chake | 16/4/2013 | KWALE | 02105 | M | 12 | 3 |
| Pemba | 2013 | Chake Chake | 16/4/2013 | KWALE | 02106 | M | 12 | 3 |
| Pemba | 2013 | Chake Chake | 16/4/2013 | KWALE | 02107 | M | 11 | 3 |
| Pemba | 2013 | Chake Chake | 16/4/2013 | KWALE | 02108 | M | 10 | 3 |
| Pemba | 2013 | Chake Chake | 16/4/2013 | KWALE | 02109 | M | 11 | 3 |
| Pemba | 2013 | Chake Chake | 16/4/2013 | KWALE | 02110 | M | 10 | 3 |
| Pemba | 2013 | Chake Chake | 16/4/2013 | KWALE | 02111 | M | 11 | 3 |
| Pemba | 2013 | Chake Chake | 16/4/2013 | KWALE | 02112 | M | 11 | 3 |
| Pemba | 2013 | Chake Chake | 16/4/2013 | KWALE | 02113 | M | 11 | 3 |
| Pemba | 2013 | Chake Chake | 16/4/2013 | KWALE | 02114 | M | 10 | 3 |
| Pemba | 2013 | Chake Chake | 16/4/2013 | KWALE | 02115 | M | 11 | 3 |
| Pemba | 2013 | Chake Chake | 16/4/2013 | KWALE | 02116 | M | 10 | 3 |
| Pemba | 2013 | Chake Chake | 16/4/2013 | KWALE | 02117 | M | 10 | 3 |
| Pemba | 2013 | Chake Chake | 16/4/2013 | KWALE | 02118 | M | 10 | 3 |
| Pemba | 2013 | Chake Chake | 16/4/2013 | KWALE | 02119 | M | 10 | 3 |
| Pemba | 2013 | Chake Chake | 16/4/2013 | KWALE | 02120 | M | 11 | 3 |
| Pemba | 2013 | Chake Chake | 16/4/2013 | KWALE | 02121 | M | 10 | 3 |
| Pemba | 2013 | Chake Chake | 16/4/2013 | KWALE | 02122 | M | 11 | 3 |
| Pemba | 2013 | Chake Chake | 16/4/2013 | KWALE | 02123 | M | 11 | 3 |
| Pemba | 2013 | Chake Chake | 16/4/2013 | KWALE | 02124 | M | 11 | 3 |
| Pemba | 2013 | Chake Chake | 16/4/2013 | KWALE | 02125 | M | 10 | 3 |
| Pemba | 2013 | Chake Chake | 16/4/2013 | KWALE | 02126 | M | 11 | 3 |
| Pemba | 2013 | Chake Chake | 16/4/2013 | KWALE | 02127 | M | 10 | 3 |
| Pemba | 2013 | Chake Chake | 16/4/2013 | KWALE | 02128 | M | 10 | 3 |
| Pemba | 2013 | Chake Chake | 16/4/2013 | KWALE | 02129 | M | 11 | 3 |
| Pemba | 2013 | Chake Chake | 16/4/2013 | KWALE | 02130 | M | 11 | 3 |
| Pemba | 2013 | Chake Chake | 03.01.2013 | WESHA | 04001 | F | 11 | 4 |
| Pemba | 2013 | Chake Chake | 03.01.2013 | WESHA | 04002 | F | 12 | 4 |
| Pemba | 2013 | Chake Chake | 03.01.2013 | WESHA | 04003 | F | 10 | 4 |
| Pemba | 2013 | Chake Chake | 03.01.2013 | WESHA | 04004 | F | 10 | 4 |
| Pemba | 2013 | Chake Chake | 03.01.2013 | WESHA | 04005 | F | 13 | 4 |
| Pemba | 2013 | Chake Chake | 03.01.2013 | WESHA | 04006 | F | 11 | 4 |
| Pemba | 2013 | Chake Chake | 03.01.2013 | WESHA | 04007 | F | 12 | 4 |
| Pemba | 2013 | Chake Chake | 03.01.2013 | WESHA | 04008 | F | 13 | 4 |
| Pemba | 2013 | Chake Chake | 03.01.2013 | WESHA | 04009 | F | 11 | 4 |
| Pemba | 2013 | Chake Chake | 03.01.2013 | WESHA | 04010 | F | 10 | 4 |
| Pemba | 2013 | Chake Chake | 03.01.2013 | WESHA | 04011 | F | 11 | 4 |
| Pemba | 2013 | Chake Chake | 03.01.2013 | WESHA | 04012 | F | 11 | 4 |
| Pemba | 2013 | Chake Chake | 03.01.2013 | WESHA | 04013 | F | 12 | 4 |
| Pemba | 2013 | Chake Chake | 03.01.2013 | WESHA | 04014 | F | 10 | 4 |
| Pemba | 2013 | Chake Chake | 03.01.2013 | WESHA | 04015 | F | 12 | 4 |
| Pemba | 2013 | Chake Chake | 03.01.2013 | WESHA | 04016 | F | 12 | 4 |
| Pemba | 2013 | Chake Chake | 03.01.2013 | WESHA | 04017 | F | 10 | 4 |
| Pemba | 2013 | Chake Chake | 03.01.2013 | WESHA | 04018 | F | 11 | 4 |
| Pemba | 2013 | Chake Chake | 03.01.2013 | WESHA | 04019 | F | 9 | 4 |

| | | | | | | | | |
|---|---|---|---|---|---|---|---|---|
| Pemba | 2013 | Chake Chake | 03.01.2013 | WESHA | 04020 | F | 10 | 4 |
| Pemba | 2013 | Chake Chake | 03.01.2013 | WESHA | 04021 | F | 10 | 4 |
| Pemba | 2013 | Chake Chake | 03.01.2013 | WESHA | 04022 | F | 11 | 4 |
| Pemba | 2013 | Chake Chake | 03.01.2013 | WESHA | 04023 | F | 12 | 4 |
| Pemba | 2013 | Chake Chake | 03.01.2013 | WESHA | 04024 | F | 11 | 4 |
| Pemba | 2013 | Chake Chake | 03.01.2013 | WESHA | 04025 | F | 10 | 4 |
| Pemba | 2013 | Chake Chake | 03.01.2013 | WESHA | 04026 | F | 10 | 4 |
| Pemba | 2013 | Chake Chake | 03.01.2013 | WESHA | 04027 | F | 11 | 4 |
| Pemba | 2013 | Chake Chake | 03.01.2013 | WESHA | 04028 | F | 11 | 4 |
| Pemba | 2013 | Chake Chake | 03.01.2013 | WESHA | 04029 | F | 11 | 4 |
| Pemba | 2013 | Chake Chake | 03.01.2013 | WESHA | 04030 | F | 12 | 4 |
| Pemba | 2013 | Chake Chake | 03.01.2013 | WESHA | 04031 | F | 11 | 4 |
| Pemba | 2013 | Chake Chake | 03.01.2013 | WESHA | 04032 | F | 10 | 4 |
| Pemba | 2013 | Chake Chake | 03.01.2013 | WESHA | 04033 | F | 10 | 4 |
| Pemba | 2013 | Chake Chake | 03.01.2013 | WESHA | 04034 | F | 11 | 4 |
| Pemba | 2013 | Chake Chake | 03.01.2013 | WESHA | 04035 | F | 10 | 4 |
| Pemba | 2013 | Chake Chake | 03.01.2013 | WESHA | 04036 | F | 11 | 4 |
| Pemba | 2013 | Chake Chake | 03.01.2013 | WESHA | 04037 | F | 10 | 4 |
| Pemba | 2013 | Chake Chake | 03.01.2013 | WESHA | 04038 | F | 10 | 4 |
| Pemba | 2013 | Chake Chake | 03.01.2013 | WESHA | 04039 | F | 10 | 4 |
| Pemba | 2013 | Chake Chake | 03.01.2013 | WESHA | 04040 | F | 11 | 4 |
| Pemba | 2013 | Chake Chake | 03.01.2013 | WESHA | 04041 | F | 11 | 4 |
| Pemba | 2013 | Chake Chake | 03.01.2013 | WESHA | 04042 | F | 11 | 4 |
| Pemba | 2013 | Chake Chake | 03.01.2013 | WESHA | 04043 | F | 12 | 4 |
| Pemba | 2013 | Chake Chake | 03.01.2013 | WESHA | 04044 | F | 12 | 4 |
| Pemba | 2013 | Chake Chake | 03.01.2013 | WESHA | 04045 | F | 12 | 4 |
| Pemba | 2013 | Chake Chake | 03.01.2013 | WESHA | 04046 | F | 11 | 4 |
| Pemba | 2013 | Chake Chake | 03.01.2013 | WESHA | 04047 | F | 10 | 4 |
| Pemba | 2013 | Chake Chake | 03.01.2013 | WESHA | 04048 | F | 10 | 4 |
| Pemba | 2013 | Chake Chake | 03.01.2013 | WESHA | 04049 | M | 12 | 4 |
| Pemba | 2013 | Chake Chake | 03.01.2013 | WESHA | 04050 | M | 12 | 4 |
| Pemba | 2013 | Chake Chake | 03.01.2013 | WESHA | 04051 | M | 10 | 4 |
| Pemba | 2013 | Chake Chake | 03.01.2013 | WESHA | 04052 | M | 12 | 4 |
| Pemba | 2013 | Chake Chake | 03.01.2013 | WESHA | 04053 | M | 11 | 4 |
| Pemba | 2013 | Chake Chake | 03.01.2013 | WESHA | 04054 | M | 11 | 4 |
| Pemba | 2013 | Chake Chake | 03.01.2013 | WESHA | 04055 | M | 12 | 4 |
| Pemba | 2013 | Chake Chake | 03.01.2013 | WESHA | 04056 | M | 12 | 4 |
| Pemba | 2013 | Chake Chake | 03.01.2013 | WESHA | 04057 | M | 12 | 4 |
| Pemba | 2013 | Chake Chake | 03.01.2013 | WESHA | 04058 | M | 12 | 4 |
| Pemba | 2013 | Chake Chake | 03.01.2013 | WESHA | 04059 | M | 11 | 4 |
| Pemba | 2013 | Chake Chake | 03.01.2013 | WESHA | 04060 | M | 11 | 4 |
| Pemba | 2013 | Chake Chake | 03.01.2013 | WESHA | 04061 | M | 11 | 4 |
| Pemba | 2013 | Chake Chake | 03.01.2013 | WESHA | 04062 | M | 11 | 4 |
| Pemba | 2013 | Chake Chake | 03.01.2013 | WESHA | 04063 | M | 11 | 4 |
| Pemba | 2013 | Chake Chake | 03.01.2013 | WESHA | 04064 | M | 12 | 4 |
| Pemba | 2013 | Chake Chake | 03.01.2013 | WESHA | 04065 | M | 10 | 4 |
| Pemba | 2013 | Chake Chake | 03.01.2013 | WESHA | 04066 | M | 12 | 4 |
| Pemba | 2013 | Chake Chake | 03.01.2013 | WESHA | 04067 | M | 12 | 4 |
| Pemba | 2013 | Chake Chake | 03.01.2013 | WESHA | 04068 | M | 12 | 4 |
| Pemba | 2013 | Chake Chake | 03.01.2013 | WESHA | 04069 | M | 11 | 4 |

| | | | | | | | | |
|---|---|---|---|---|---|---|---|---|
| Pemba | 2013 | Chake Chake | 03.01.2013 | WESHA | 04070 | M | 10 | 4 |
| Pemba | 2013 | Chake Chake | 03.01.2013 | WESHA | 04071 | M | 11 | 4 |
| Pemba | 2013 | Chake Chake | 03.01.2013 | WESHA | 04072 | M | 12 | 4 |
| Pemba | 2013 | Chake Chake | 03.01.2013 | WESHA | 04073 | M | 12 | 4 |
| Pemba | 2013 | Chake Chake | 03.01.2013 | WESHA | 04074 | M | 12 | 4 |
| Pemba | 2013 | Chake Chake | 03.01.2013 | WESHA | 04075 | M | 10 | 4 |
| Pemba | 2013 | Chake Chake | 03.01.2013 | WESHA | 04076 | M | 10 | 4 |
| Pemba | 2013 | Chake Chake | 03.01.2013 | WESHA | 04077 | M | 12 | 4 |
| Pemba | 2013 | Chake Chake | 03.01.2013 | WESHA | 04078 | M | 11 | 4 |
| Pemba | 2013 | Chake Chake | 03.01.2013 | WESHA | 04079 | M | 11 | 4 |
| Pemba | 2013 | Chake Chake | 03.01.2013 | WESHA | 04080 | M | 12 | 4 |
| Pemba | 2013 | Chake Chake | 03.01.2013 | WESHA | 04081 | M | 11 | 4 |
| Pemba | 2013 | Chake Chake | 03.01.2013 | WESHA | 04082 | M | 12 | 4 |
| Pemba | 2013 | Chake Chake | 03.01.2013 | WESHA | 04083 | M | 11 | 4 |
| Pemba | 2013 | Chake Chake | 03.01.2013 | WESHA | 04084 | M | 11 | 4 |
| Pemba | 2013 | Chake Chake | 03.01.2013 | WESHA | 04085 | M | 11 | 4 |
| Pemba | 2013 | Chake Chake | 03.01.2013 | WESHA | 04086 | M | 11 | 4 |
| Pemba | 2013 | Chake Chake | 03.01.2013 | WESHA | 04087 | M | 11 | 4 |
| Pemba | 2013 | Chake Chake | 03.01.2013 | WESHA | 04088 | M | 11 | 4 |
| Pemba | 2013 | Chake Chake | 03.01.2013 | WESHA | 04089 | F | 12 | 3 |
| Pemba | 2013 | Chake Chake | 03.01.2013 | WESHA | 04090 | F | 11 | 3 |
| Pemba | 2013 | Chake Chake | 03.01.2013 | WESHA | 04091 | F | 11 | 3 |
| Pemba | 2013 | Chake Chake | 03.01.2013 | WESHA | 04092 | F | 9 | 3 |
| Pemba | 2013 | Chake Chake | 03.01.2013 | WESHA | 04093 | F | 9 | 3 |
| Pemba | 2013 | Chake Chake | 03.01.2013 | WESHA | 04094 | F | 9 | 3 |
| Pemba | 2013 | Chake Chake | 03.01.2013 | WESHA | 04095 | F | 10 | 3 |
| Pemba | 2013 | Chake Chake | 03.01.2013 | WESHA | 04096 | F | 10 | 3 |
| Pemba | 2013 | Chake Chake | 03.01.2013 | WESHA | 04097 | M | 11 | 3 |
| Pemba | 2013 | Chake Chake | 03.01.2013 | WESHA | 04098 | M | 12 | 3 |
| Pemba | 2013 | Chake Chake | 03.01.2013 | WESHA | 04099 | M | 11 | 3 |
| Pemba | 2013 | Chake Chake | 03.01.2013 | WESHA | 04100 | M | 9 | 3 |
| Pemba | 2013 | Chake Chake | 03.01.2013 | WESHA | 04101 | F | 10 | 3 |
| Pemba | 2013 | Chake Chake | 03.01.2013 | WESHA | 04102 | F | 10 | 3 |
| Pemba | 2013 | Chake Chake | 03.01.2013 | WESHA | 04103 | F | 11 | 3 |
| Pemba | 2013 | Chake Chake | 03.01.2013 | WESHA | 04104 | F | 11 | 3 |
| Pemba | 2013 | Chake Chake | 03.01.2013 | WESHA | 04105 | F | 11 | 3 |
| Pemba | 2013 | Chake Chake | 03.01.2013 | WESHA | 04106 | F | 9 | 3 |
| Pemba | 2013 | Chake Chake | 03.01.2013 | WESHA | 04107 | F | 12 | 3 |
| Pemba | 2013 | Chake Chake | 03.01.2013 | WESHA | 04108 | M | 10 | 3 |
| Pemba | 2013 | Chake Chake | 03.01.2013 | WESHA | 04109 | M | 10 | 3 |
| Pemba | 2013 | Chake Chake | 03.01.2013 | WESHA | 04110 | M | 11 | 3 |
| Pemba | 2013 | Chake Chake | 03.01.2013 | WESHA | 04111 | M | 11 | 3 |
| Pemba | 2013 | Chake Chake | 03.01.2013 | WESHA | 04112 | M | 11 | 3 |
| Pemba | 2013 | Chake Chake | 03.01.2013 | WESHA | 04113 | M | 9 | 3 |
| Pemba | 2013 | Chake Chake | 03.01.2013 | WESHA | 04114 | M | 10 | 3 |
| Pemba | 2013 | Chake Chake | 03.01.2013 | WESHA | 04115 | M | 9 | 3 |
| Pemba | 2013 | Chake Chake | 03.01.2013 | WESHA | 04116 | M | 11 | 3 |
| Pemba | 2013 | Chake Chake | 03.01.2013 | WESHA | 04117 | M | 11 | 3 |
| Pemba | 2013 | Chake Chake | 03.01.2013 | WESHA | 04118 | M | 10 | 3 |
| Pemba | 2013 | Chake Chake | 03.01.2013 | WESHA | 04119 | M | 9 | 3 |

| | | | | | | | | |
|---|---|---|---|---|---|---|---|---|
| Pemba | 2013 | Chake Chake | 03.01.2013 | WESHA | 04120 | M | 10 | 3 |
| Pemba | 2013 | Chake Chake | 03.01.2013 | WESHA | 04121 | M | 10 | 3 |
| Pemba | 2013 | Chake Chake | 03.01.2013 | WESHA | 04122 | M | 10 | 3 |
| Pemba | 2013 | Chake Chake | 03.01.2013 | WESHA | 04123 | M | 10 | 3 |
| Pemba | 2013 | Chake Chake | 03.01.2013 | WESHA | 04124 | F | 10 | 3 |
| Pemba | 2013 | Chake Chake | 03.01.2013 | WESHA | 04125 | F | 10 | 3 |
| Pemba | 2013 | Chake Chake | 03.01.2013 | WESHA | 04126 | F | 9 | 3 |
| Pemba | 2013 | Chake Chake | 03.01.2013 | WESHA | 04127 | F | 9 | 3 |
| Pemba | 2013 | Chake Chake | 03.01.2013 | WESHA | 04128 | F | 10 | 3 |
| Pemba | 2013 | Chake Chake | 03.01.2013 | WESHA | 04129 | F | 11 | 3 |
| Pemba | 2013 | Chake Chake | 03.01.2013 | WESHA | 04130 | F | 11 | 3 |
| Pemba | 2013 | Chake Chake | 03.01.2013 | WESHA | 04257 | M | 12 | 4 |
| Pemba | 2013 | Chake Chake | 19/4/2013 | WAWI | 06001 | F | 10 | 4 |
| Pemba | 2013 | Chake Chake | 19/4/2013 | WAWI | 06002 | F | 9 | 4 |
| Pemba | 2013 | Chake Chake | 19/4/2013 | WAWI | 06003 | F | 11 | 4 |
| Pemba | 2013 | Chake Chake | 19/4/2013 | WAWI | 06004 | F | 11 | 4 |
| Pemba | 2013 | Chake Chake | 19/4/2013 | WAWI | 06005 | F | 11 | 4 |
| Pemba | 2013 | Chake Chake | 19/4/2013 | WAWI | 06006 | F | 10 | 4 |
| Pemba | 2013 | Chake Chake | 19/4/2013 | WAWI | 06007 | F | 11 | 4 |
| Pemba | 2013 | Chake Chake | 19/4/2013 | WAWI | 06008 | F | 11 | 4 |
| Pemba | 2013 | Chake Chake | 19/4/2013 | WAWI | 06009 | F | 10 | 4 |
| Pemba | 2013 | Chake Chake | 19/4/2013 | WAWI | 06010 | F | 11 | 4 |
| Pemba | 2013 | Chake Chake | 19/4/2013 | WAWI | 06011 | F | 11 | 4 |
| Pemba | 2013 | Chake Chake | 19/4/2013 | WAWI | 06012 | F | 10 | 4 |
| Pemba | 2013 | Chake Chake | 19/4/2013 | WAWI | 06013 | F | 9 | 4 |
| Pemba | 2013 | Chake Chake | 19/4/2013 | WAWI | 06014 | F | 9 | 4 |
| Pemba | 2013 | Chake Chake | 19/4/2013 | WAWI | 06015 | F | 11 | 4 |
| Pemba | 2013 | Chake Chake | 19/4/2013 | WAWI | 06016 | F | 11 | 4 |
| Pemba | 2013 | Chake Chake | 19/4/2013 | WAWI | 06017 | F | 11 | 4 |
| Pemba | 2013 | Chake Chake | 19/4/2013 | WAWI | 06018 | F | 10 | 4 |
| Pemba | 2013 | Chake Chake | 19/4/2013 | WAWI | 06019 | F | 10 | 4 |
| Pemba | 2013 | Chake Chake | 19/4/2013 | WAWI | 06020 | F | 11 | 4 |
| Pemba | 2013 | Chake Chake | 19/4/2013 | WAWI | 06021 | F | 10 | 4 |
| Pemba | 2013 | Chake Chake | 19/4/2013 | WAWI | 06022 | F | 9 | 4 |
| Pemba | 2013 | Chake Chake | 19/4/2013 | WAWI | 06023 | F | 11 | 4 |
| Pemba | 2013 | Chake Chake | 19/4/2013 | WAWI | 06024 | F | 11 | 4 |
| Pemba | 2013 | Chake Chake | 19/4/2013 | WAWI | 06025 | F | 10 | 4 |
| Pemba | 2013 | Chake Chake | 19/4/2013 | WAWI | 06026 | F | 11 | 4 |
| Pemba | 2013 | Chake Chake | 19/4/2013 | WAWI | 06027 | F | 10 | 4 |
| Pemba | 2013 | Chake Chake | 19/4/2013 | WAWI | 06028 | F | 11 | 4 |
| Pemba | 2013 | Chake Chake | 19/4/2013 | WAWI | 06029 | F | 10 | 4 |
| Pemba | 2013 | Chake Chake | 19/4/2013 | WAWI | 06030 | F | 11 | 4 |
| Pemba | 2013 | Chake Chake | 19/4/2013 | WAWI | 06031 | F | 12 | 4 |
| Pemba | 2013 | Chake Chake | 19/4/2013 | WAWI | 06032 | F | 10 | 4 |
| Pemba | 2013 | Chake Chake | 19/4/2013 | WAWI | 06033 | F | 11 | 4 |
| Pemba | 2013 | Chake Chake | 19/4/2013 | WAWI | 06034 | M | 10 | 4 |
| Pemba | 2013 | Chake Chake | 19/4/2013 | WAWI | 06035 | M | 10 | 4 |
| Pemba | 2013 | Chake Chake | 19/4/2013 | WAWI | 06036 | M | 11 | 4 |
| Pemba | 2013 | Chake Chake | 19/4/2013 | WAWI | 06037 | M | 10 | 4 |
| Pemba | 2013 | Chake Chake | 19/4/2013 | WAWI | 06038 | M | 10 | 4 |

| | | | | | | | | |
|---|---|---|---|---|---|---|---|---|
| Pemba | 2013 | Chake Chake | 19/4/2013 | WAWI | 06039 | M | 11 | 4 |
| Pemba | 2013 | Chake Chake | 19/4/2013 | WAWI | 06040 | M | 11 | 4 |
| Pemba | 2013 | Chake Chake | 19/4/2013 | WAWI | 06041 | M | 11 | 4 |
| Pemba | 2013 | Chake Chake | 19/4/2013 | WAWI | 06042 | M | 11 | 4 |
| Pemba | 2013 | Chake Chake | 19/4/2013 | WAWI | 06043 | M | 11 | 4 |
| Pemba | 2013 | Chake Chake | 19/4/2013 | WAWI | 06044 | M | 11 | 4 |
| Pemba | 2013 | Chake Chake | 19/4/2013 | WAWI | 06045 | M | 10 | 4 |
| Pemba | 2013 | Chake Chake | 19/4/2013 | WAWI | 06046 | M | 10 | 4 |
| Pemba | 2013 | Chake Chake | 19/4/2013 | WAWI | 06047 | M | 11 | 4 |
| Pemba | 2013 | Chake Chake | 19/4/2013 | WAWI | 06048 | M | 11 | 4 |
| Pemba | 2013 | Chake Chake | 19/4/2013 | WAWI | 06049 | M | 10 | 4 |
| Pemba | 2013 | Chake Chake | 19/4/2013 | WAWI | 06050 | M | 12 | 4 |
| Pemba | 2013 | Chake Chake | 19/4/2013 | WAWI | 06051 | M | 10 | 4 |
| Pemba | 2013 | Chake Chake | 19/4/2013 | WAWI | 06052 | M | 10 | 4 |
| Pemba | 2013 | Chake Chake | 19/4/2013 | WAWI | 06053 | M | 12 | 4 |
| Pemba | 2013 | Chake Chake | 19/4/2013 | WAWI | 06054 | M | 11 | 4 |
| Pemba | 2013 | Chake Chake | 19/4/2013 | WAWI | 06055 | M | 10 | 4 |
| Pemba | 2013 | Chake Chake | 19/4/2013 | WAWI | 06056 | M | 11 | 4 |
| Pemba | 2013 | Chake Chake | 19/4/2013 | WAWI | 06057 | M | 11 | 4 |
| Pemba | 2013 | Chake Chake | 19/4/2013 | WAWI | 06058 | M | 11 | 4 |
| Pemba | 2013 | Chake Chake | 19/4/2013 | WAWI | 06059 | M | 10 | 4 |
| Pemba | 2013 | Chake Chake | 19/4/2013 | WAWI | 06060 | M | 11 | 4 |
| Pemba | 2013 | Chake Chake | 19/4/2013 | WAWI | 06061 | M | 10 | 4 |
| Pemba | 2013 | Chake Chake | 19/4/2013 | WAWI | 06062 | M | 11 | 4 |
| Pemba | 2013 | Chake Chake | 19/4/2013 | WAWI | 06063 | M | 10 | 4 |
| Pemba | 2013 | Chake Chake | 19/4/2013 | WAWI | 06064 | M | 11 | 4 |
| Pemba | 2013 | Chake Chake | 19/4/2013 | WAWI | 06065 | M | 11 | 4 |
| Pemba | 2013 | Chake Chake | 19/4/2013 | WAWI | 06066 | F | 9 | 4 |
| Pemba | 2013 | Chake Chake | 19/4/2013 | WAWI | 06067 | F | 9 | 4 |
| Pemba | 2013 | Chake Chake | 19/4/2013 | WAWI | 06068 | F | 10 | 4 |
| Pemba | 2013 | Chake Chake | 19/4/2013 | WAWI | 06069 | F | 10 | 4 |
| Pemba | 2013 | Chake Chake | 19/4/2013 | WAWI | 06070 | F | 9 | 4 |
| Pemba | 2013 | Chake Chake | 19/4/2013 | WAWI | 06071 | F | 9 | 4 |
| Pemba | 2013 | Chake Chake | 19/4/2013 | WAWI | 06072 | F | 9 | 4 |
| Pemba | 2013 | Chake Chake | 19/4/2013 | WAWI | 06073 | F | 9 | 4 |
| Pemba | 2013 | Chake Chake | 19/4/2013 | WAWI | 06074 | F | 9 | 4 |
| Pemba | 2013 | Chake Chake | 19/4/2013 | WAWI | 06075 | F | 9 | 4 |
| Pemba | 2013 | Chake Chake | 19/4/2013 | WAWI | 06076 | F | 9 | 3 |
| Pemba | 2013 | Chake Chake | 19/4/2013 | WAWI | 06077 | F | 10 | 3 |
| Pemba | 2013 | Chake Chake | 19/4/2013 | WAWI | 06078 | F | 10 | 3 |
| Pemba | 2013 | Chake Chake | 19/4/2013 | WAWI | 06079 | F | 11 | 3 |
| Pemba | 2013 | Chake Chake | 19/4/2013 | WAWI | 06080 | F | 10 | 3 |
| Pemba | 2013 | Chake Chake | 19/4/2013 | WAWI | 06081 | F | 11 | 3 |
| Pemba | 2013 | Chake Chake | 19/4/2013 | WAWI | 06082 | F | 11 | 3 |
| Pemba | 2013 | Chake Chake | 19/4/2013 | WAWI | 06083 | F | 10 | 3 |
| Pemba | 2013 | Chake Chake | 19/4/2013 | WAWI | 06084 | F | 10 | 3 |
| Pemba | 2013 | Chake Chake | 19/4/2013 | WAWI | 06085 | F | 10 | 3 |
| Pemba | 2013 | Chake Chake | 19/4/2013 | WAWI | 06086 | F | 9 | 3 |
| Pemba | 2013 | Chake Chake | 19/4/2013 | WAWI | 06087 | F | 10 | 3 |
| Pemba | 2013 | Chake Chake | 19/4/2013 | WAWI | 06088 | F | 10 | 3 |

| | | | | | | | | |
|---|---|---|---|---|---|---|---|---|
| Pemba | 2013 | Chake Chake | 19/4/2013 | WAWI | 06089 | F | 10 | 3 |
| Pemba | 2013 | Chake Chake | 19/4/2013 | WAWI | 06090 | F | 10 | 3 |
| Pemba | 2013 | Chake Chake | 19/4/2013 | WAWI | 06091 | F | 10 | 3 |
| Pemba | 2013 | Chake Chake | 19/4/2013 | WAWI | 06092 | F | 11 | 3 |
| Pemba | 2013 | Chake Chake | 19/4/2013 | WAWI | 06093 | F | 9 | 3 |
| Pemba | 2013 | Chake Chake | 19/4/2013 | WAWI | 06094 | F | 10 | 3 |
| Pemba | 2013 | Chake Chake | 19/4/2013 | WAWI | 06095 | M | 10 | 3 |
| Pemba | 2013 | Chake Chake | 19/4/2013 | WAWI | 06096 | M | 10 | 3 |
| Pemba | 2013 | Chake Chake | 19/4/2013 | WAWI | 06097 | M | 10 | 3 |
| Pemba | 2013 | Chake Chake | 19/4/2013 | WAWI | 06098 | M | 12 | 3 |
| Pemba | 2013 | Chake Chake | 19/4/2013 | WAWI | 06099 | M | 11 | 3 |
| Pemba | 2013 | Chake Chake | 19/4/2013 | WAWI | 06100 | M | 10 | 3 |
| Pemba | 2013 | Chake Chake | 19/4/2013 | WAWI | 06101 | M | 10 | 3 |
| Pemba | 2013 | Chake Chake | 19/4/2013 | WAWI | 06102 | M | 10 | 3 |
| Pemba | 2013 | Chake Chake | 19/4/2013 | WAWI | 06103 | M | 11 | 3 |
| Pemba | 2013 | Chake Chake | 19/4/2013 | WAWI | 06104 | M | 11 | 3 |
| Pemba | 2013 | Chake Chake | 19/4/2013 | WAWI | 06105 | M | 10 | 3 |
| Pemba | 2013 | Chake Chake | 19/4/2013 | WAWI | 06106 | M | 11 | 3 |
| Pemba | 2013 | Chake Chake | 19/4/2013 | WAWI | 06107 | M | 11 | 3 |
| Pemba | 2013 | Chake Chake | 19/4/2013 | WAWI | 06108 | M | 11 | 3 |
| Pemba | 2013 | Chake Chake | 19/4/2013 | WAWI | 06109 | M | 10 | 3 |
| Pemba | 2013 | Chake Chake | 19/4/2013 | WAWI | 06110 | M | 10 | 3 |
| Pemba | 2013 | Chake Chake | 19/4/2013 | WAWI | 06111 | M | 10 | 3 |
| Pemba | 2013 | Chake Chake | 19/4/2013 | WAWI | 06112 | M | 11 | 3 |
| Pemba | 2013 | Chake Chake | 19/4/2013 | WAWI | 06113 | M | 10 | 3 |
| Pemba | 2013 | Chake Chake | 19/4/2013 | WAWI | 06114 | M | 9 | 3 |
| Pemba | 2013 | Chake Chake | 19/4/2013 | WAWI | 06115 | M | 10 | 3 |
| Pemba | 2013 | Chake Chake | 19/4/2013 | WAWI | 06116 | M | 11 | 3 |
| Pemba | 2013 | Chake Chake | 19/4/2013 | WAWI | 06117 | M | 10 | 3 |
| Pemba | 2013 | Chake Chake | 19/4/2013 | WAWI | 06118 | M | 11 | 3 |
| Pemba | 2013 | Chake Chake | 19/4/2013 | WAWI | 06119 | M | 12 | 3 |
| Pemba | 2013 | Chake Chake | 19/4/2013 | WAWI | 06120 | M | 10 | 3 |
| Pemba | 2013 | Chake Chake | 19/4/2013 | WAWI | 06121 | M | 10 | 3 |
| Pemba | 2013 | Chake Chake | 19/4/2013 | WAWI | 06122 | M | 11 | 3 |
| Pemba | 2013 | Chake Chake | 19/4/2013 | WAWI | 06123 | M | 9 | 3 |
| Pemba | 2013 | Chake Chake | 19/4/2013 | WAWI | 06124 | M | 10 | 3 |
| Pemba | 2013 | Chake Chake | 19/4/2013 | WAWI | 06125 | M | 11 | 3 |
| Pemba | 2013 | Chake Chake | 19/4/2013 | WAWI | 06126 | M | 10 | 3 |
| Pemba | 2013 | Chake Chake | 19/4/2013 | WAWI | 06127 | M | 10 | 3 |
| Pemba | 2013 | Chake Chake | 19/4/2013 | WAWI | 06128 | M | 11 | 3 |
| Pemba | 2013 | Chake Chake | 19/4/2013 | WAWI | 06129 | M | 11 | 3 |
| Pemba | 2013 | Chake Chake | 19/4/2013 | WAWI | 06130 | M | 10 | 3 |
| Pemba | 2013 | Chake Chake | 24/4/2013 | SHUNGI | 07001 | F | 12 | 4 |
| Pemba | 2013 | Chake Chake | 24/4/2013 | SHUNGI | 07002 | F | 10 | 4 |
| Pemba | 2013 | Chake Chake | 24/4/2013 | SHUNGI | 07003 | F | 10 | 4 |
| Pemba | 2013 | Chake Chake | 24/4/2013 | SHUNGI | 07004 | F | 10 | 4 |
| Pemba | 2013 | Chake Chake | 24/4/2013 | SHUNGI | 07005 | F | 12 | 4 |
| Pemba | 2013 | Chake Chake | 24/4/2013 | SHUNGI | 07006 | F | 11 | 4 |
| Pemba | 2013 | Chake Chake | 24/4/2013 | SHUNGI | 07007 | F | 12 | 4 |
| Pemba | 2013 | Chake Chake | 24/4/2013 | SHUNGI | 07008 | F | 11 | 4 |

| | | | | | | | | |
|---|---|---|---|---|---|---|---|---|
| Pemba | 2013 | Chake Chake | 24/4/2013 | SHUNGI | 07009 | F | 12 | 4 |
| Pemba | 2013 | Chake Chake | 24/4/2013 | SHUNGI | 07010 | F | 12 | 4 |
| Pemba | 2013 | Chake Chake | 24/4/2013 | SHUNGI | 07011 | F | 11 | 4 |
| Pemba | 2013 | Chake Chake | 24/4/2013 | SHUNGI | 07012 | F | 12 | 4 |
| Pemba | 2013 | Chake Chake | 24/4/2013 | SHUNGI | 07013 | F | 10 | 4 |
| Pemba | 2013 | Chake Chake | 24/4/2013 | SHUNGI | 07014 | F | 10 | 4 |
| Pemba | 2013 | Chake Chake | 24/4/2013 | SHUNGI | 07015 | F | 11 | 4 |
| Pemba | 2013 | Chake Chake | 24/4/2013 | SHUNGI | 07016 | F | 11 | 4 |
| Pemba | 2013 | Chake Chake | 24/4/2013 | SHUNGI | 07017 | F | 10 | 4 |
| Pemba | 2013 | Chake Chake | 24/4/2013 | SHUNGI | 07018 | F | 9 | 4 |
| Pemba | 2013 | Chake Chake | 24/4/2013 | SHUNGI | 07019 | F | 12 | 4 |
| Pemba | 2013 | Chake Chake | 24/4/2013 | SHUNGI | 07020 | F | 12 | 4 |
| Pemba | 2013 | Chake Chake | 24/4/2013 | SHUNGI | 07021 | F | 12 | 4 |
| Pemba | 2013 | Chake Chake | 24/4/2013 | SHUNGI | 07022 | M | 10 | 4 |
| Pemba | 2013 | Chake Chake | 24/4/2013 | SHUNGI | 07023 | M | 10 | 4 |
| Pemba | 2013 | Chake Chake | 24/4/2013 | SHUNGI | 07024 | M | 11 | 4 |
| Pemba | 2013 | Chake Chake | 24/4/2013 | SHUNGI | 07025 | M | 12 | 4 |
| Pemba | 2013 | Chake Chake | 24/4/2013 | SHUNGI | 07026 | M | 11 | 4 |
| Pemba | 2013 | Chake Chake | 24/4/2013 | SHUNGI | 07027 | M | 11 | 4 |
| Pemba | 2013 | Chake Chake | 24/4/2013 | SHUNGI | 07028 | M | 11 | 4 |
| Pemba | 2013 | Chake Chake | 24/4/2013 | SHUNGI | 07029 | M | 11 | 4 |
| Pemba | 2013 | Chake Chake | 24/4/2013 | SHUNGI | 07030 | M | 10 | 4 |
| Pemba | 2013 | Chake Chake | 24/4/2013 | SHUNGI | 07031 | M | 10 | 4 |
| Pemba | 2013 | Chake Chake | 24/4/2013 | SHUNGI | 07032 | M | 11 | 4 |
| Pemba | 2013 | Chake Chake | 24/4/2013 | SHUNGI | 07033 | M | 11 | 4 |
| Pemba | 2013 | Chake Chake | 24/4/2013 | SHUNGI | 07034 | M | 12 | 4 |
| Pemba | 2013 | Chake Chake | 24/4/2013 | SHUNGI | 07035 | M | 12 | 4 |
| Pemba | 2013 | Chake Chake | 24/4/2013 | SHUNGI | 07036 | M | 10 | 4 |
| Pemba | 2013 | Chake Chake | 24/4/2013 | SHUNGI | 07037 | M | 11 | 4 |
| Pemba | 2013 | Chake Chake | 24/4/2013 | SHUNGI | 07038 | M | 11 | 4 |
| Pemba | 2013 | Chake Chake | 24/4/2013 | SHUNGI | 07039 | M | 11 | 4 |
| Pemba | 2013 | Chake Chake | 24/4/2013 | SHUNGI | 07040 | M | 10 | 4 |
| Pemba | 2013 | Chake Chake | 24/4/2013 | SHUNGI | 07041 | M | 12 | 4 |
| Pemba | 2013 | Chake Chake | 24/4/2013 | SHUNGI | 07042 | M | 11 | 4 |
| Pemba | 2013 | Chake Chake | 24/4/2013 | SHUNGI | 07043 | M | 12 | 4 |
| Pemba | 2013 | Chake Chake | 24/4/2013 | SHUNGI | 07044 | M | 10 | 4 |
| Pemba | 2013 | Chake Chake | 24/4/2013 | SHUNGI | 07045 | F | 10 | 4 |
| Pemba | 2013 | Chake Chake | 24/4/2013 | SHUNGI | 07046 | F | 11 | 4 |
| Pemba | 2013 | Chake Chake | 24/4/2013 | SHUNGI | 07047 | F | 10 | 4 |
| Pemba | 2013 | Chake Chake | 24/4/2013 | SHUNGI | 07048 | F | 10 | 4 |
| Pemba | 2013 | Chake Chake | 24/4/2013 | SHUNGI | 07049 | F | 10 | 4 |
| Pemba | 2013 | Chake Chake | 24/4/2013 | SHUNGI | 07050 | F | 10 | 4 |
| Pemba | 2013 | Chake Chake | 24/4/2013 | SHUNGI | 07051 | F | 9 | 3 |
| Pemba | 2013 | Chake Chake | 24/4/2013 | SHUNGI | 07052 | F | 9 | 3 |
| Pemba | 2013 | Chake Chake | 24/4/2013 | SHUNGI | 07053 | F | 10 | 3 |
| Pemba | 2013 | Chake Chake | 24/4/2013 | SHUNGI | 07054 | F | 11 | 3 |
| Pemba | 2013 | Chake Chake | 24/4/2013 | SHUNGI | 07055 | F | 10 | 3 |
| Pemba | 2013 | Chake Chake | 24/4/2013 | SHUNGI | 07056 | F | 10 | 3 |
| Pemba | 2013 | Chake Chake | 24/4/2013 | SHUNGI | 07057 | F | 10 | 3 |
| Pemba | 2013 | Chake Chake | 24/4/2013 | SHUNGI | 07058 | F | 11 | 3 |

| | | | | | | | | |
|---|---|---|---|---|---|---|---|---|
| Pemba | 2013 | Chake Chake | 24/4/2013 | SHUNGI | 07059 | F | 10 | 3 |
| Pemba | 2013 | Chake Chake | 24/4/2013 | SHUNGI | 07060 | F | 10 | 3 |
| Pemba | 2013 | Chake Chake | 24/4/2013 | SHUNGI | 07061 | F | 12 | 3 |
| Pemba | 2013 | Chake Chake | 24/4/2013 | SHUNGI | 07062 | F | 11 | 3 |
| Pemba | 2013 | Chake Chake | 24/4/2013 | SHUNGI | 07063 | F | 11 | 3 |
| Pemba | 2013 | Chake Chake | 24/4/2013 | SHUNGI | 07064 | F | 10 | 3 |
| Pemba | 2013 | Chake Chake | 24/4/2013 | SHUNGI | 07065 | F | 10 | 3 |
| Pemba | 2013 | Chake Chake | 24/4/2013 | SHUNGI | 07066 | F | 10 | 3 |
| Pemba | 2013 | Chake Chake | 24/4/2013 | SHUNGI | 07067 | F | 11 | 3 |
| Pemba | 2013 | Chake Chake | 24/4/2013 | SHUNGI | 07068 | F | 9 | 3 |
| Pemba | 2013 | Chake Chake | 24/4/2013 | SHUNGI | 07069 | F | 10 | 3 |
| Pemba | 2013 | Chake Chake | 24/4/2013 | SHUNGI | 07070 | F | 9 | 3 |
| Pemba | 2013 | Chake Chake | 24/4/2013 | SHUNGI | 07071 | F | 11 | 3 |
| Pemba | 2013 | Chake Chake | 24/4/2013 | SHUNGI | 07072 | F | 10 | 3 |
| Pemba | 2013 | Chake Chake | 24/4/2013 | SHUNGI | 07073 | F | 11 | 3 |
| Pemba | 2013 | Chake Chake | 24/4/2013 | SHUNGI | 07074 | F | 11 | 3 |
| Pemba | 2013 | Chake Chake | 24/4/2013 | SHUNGI | 07075 | F | 10 | 3 |
| Pemba | 2013 | Chake Chake | 24/4/2013 | SHUNGI | 07076 | F | 11 | 3 |
| Pemba | 2013 | Chake Chake | 24/4/2013 | SHUNGI | 07077 | F | 10 | 3 |
| Pemba | 2013 | Chake Chake | 24/4/2013 | SHUNGI | 07078 | M | 9 | 3 |
| Pemba | 2013 | Chake Chake | 24/4/2013 | SHUNGI | 07079 | M | 12 | 3 |
| Pemba | 2013 | Chake Chake | 24/4/2013 | SHUNGI | 07080 | M | 10 | 3 |
| Pemba | 2013 | Chake Chake | 24/4/2013 | SHUNGI | 07081 | M | 9 | 3 |
| Pemba | 2013 | Chake Chake | 24/4/2013 | SHUNGI | 07082 | M | 11 | 3 |
| Pemba | 2013 | Chake Chake | 24/4/2013 | SHUNGI | 07083 | M | 12 | 3 |
| Pemba | 2013 | Chake Chake | 24/4/2013 | SHUNGI | 07084 | M | 10 | 3 |
| Pemba | 2013 | Chake Chake | 24/4/2013 | SHUNGI | 07085 | M | 11 | 3 |
| Pemba | 2013 | Chake Chake | 24/4/2013 | SHUNGI | 07086 | M | 11 | 3 |
| Pemba | 2013 | Chake Chake | 24/4/2013 | SHUNGI | 07087 | M | 9 | 3 |
| Pemba | 2013 | Chake Chake | 24/4/2013 | SHUNGI | 07088 | M | 9 | 3 |
| Pemba | 2013 | Chake Chake | 24/4/2013 | SHUNGI | 07089 | M | 9 | 3 |
| Pemba | 2013 | Chake Chake | 24/4/2013 | SHUNGI | 07090 | M | 11 | 3 |
| Pemba | 2013 | Chake Chake | 24/4/2013 | SHUNGI | 07091 | M | 10 | 3 |
| Pemba | 2013 | Chake Chake | 24/4/2013 | SHUNGI | 07092 | M | 10 | 3 |
| Pemba | 2013 | Chake Chake | 24/4/2013 | SHUNGI | 07093 | M | 11 | 3 |
| Pemba | 2013 | Chake Chake | 24/4/2013 | SHUNGI | 07094 | M | 10 | 3 |
| Pemba | 2013 | Chake Chake | 24/4/2013 | SHUNGI | 07095 | M | 10 | 3 |
| Pemba | 2013 | Chake Chake | 24/4/2013 | SHUNGI | 07096 | M | 11 | 3 |
| Pemba | 2013 | Chake Chake | 24/4/2013 | SHUNGI | 07097 | M | 11 | 3 |
| Pemba | 2013 | Chake Chake | 24/4/2013 | SHUNGI | 07098 | M | 10 | 3 |
| Pemba | 2013 | Chake Chake | 24/4/2013 | SHUNGI | 07099 | M | 11 | 3 |
| Pemba | 2013 | Chake Chake | 24/4/2013 | SHUNGI | 07100 | M | 11 | 3 |
| Pemba | 2013 | Chake Chake | 24/4/2013 | SHUNGI | 07101 | M | 11 | 3 |
| Pemba | 2013 | Chake Chake | 24/4/2013 | SHUNGI | 07102 | M | 10 | 3 |
| Pemba | 2013 | Chake Chake | 24/4/2013 | SHUNGI | 07103 | M | 10 | 3 |
| Pemba | 2013 | Chake Chake | 24/4/2013 | SHUNGI | 07104 | M | 10 | 3 |
| Pemba | 2013 | Chake Chake | 24/4/2013 | SHUNGI | 07105 | M | 9 | 3 |
| Pemba | 2013 | Chake Chake | 24/4/2013 | SHUNGI | 07106 | M | 11 | 3 |
| Pemba | 2013 | Chake Chake | 24/4/2013 | SHUNGI | 07107 | M | 11 | 3 |
| Pemba | 2013 | Chake Chake | 24/4/2013 | SHUNGI | 07108 | M | 12 | 3 |

| | | | | | | | | |
|---|---|---|---|---|---|---|---|---|
| Pemba | 2013 | Chake Chake | 24/4/2013 | SHUNGI | 07109 | M | 10 | 3 |
| Pemba | 2013 | Chake Chake | 24/4/2013 | SHUNGI | 07110 | M | 12 | 3 |
| Pemba | 2013 | Chake Chake | 24/4/2013 | SHUNGI | 07111 | M | 10 | 3 |
| Pemba | 2013 | Chake Chake | 24/4/2013 | SHUNGI | 07112 | M | 11 | 3 |
| Pemba | 2013 | Chake Chake | 24/4/2013 | SHUNGI | 07113 | M | 10 | 3 |
| Pemba | 2013 | Chake Chake | 24/4/2013 | SHUNGI | 07114 | M | 11 | 3 |
| Pemba | 2013 | Chake Chake | 24/4/2013 | SHUNGI | 07115 | M | 11 | 3 |
| Pemba | 2013 | Chake Chake | 24/4/2013 | SHUNGI | 07116 | M | 12 | 3 |
| Pemba | 2013 | Chake Chake | 26/2/2013 | CHANJAMJAWIRI | 08001 | F | 12 | 4 |
| Pemba | 2013 | Chake Chake | 26/2/2013 | CHANJAMJAWIRI | 08002 | F | 12 | 4 |
| Pemba | 2013 | Chake Chake | 26/2/2013 | CHANJAMJAWIRI | 08003 | F | 11 | 4 |
| Pemba | 2013 | Chake Chake | 26/2/2013 | CHANJAMJAWIRI | 08004 | F | 12 | 4 |
| Pemba | 2013 | Chake Chake | 26/2/2013 | CHANJAMJAWIRI | 08005 | F | 11 | 4 |
| Pemba | 2013 | Chake Chake | 26/2/2013 | CHANJAMJAWIRI | 08006 | F | 10 | 4 |
| Pemba | 2013 | Chake Chake | 26/2/2013 | CHANJAMJAWIRI | 08007 | F | 11 | 4 |
| Pemba | 2013 | Chake Chake | 26/2/2013 | CHANJAMJAWIRI | 08008 | F | 11 | 4 |
| Pemba | 2013 | Chake Chake | 26/2/2013 | CHANJAMJAWIRI | 08009 | F | 11 | 4 |
| Pemba | 2013 | Chake Chake | 26/2/2013 | CHANJAMJAWIRI | 08010 | F | 11 | 4 |
| Pemba | 2013 | Chake Chake | 26/2/2013 | CHANJAMJAWIRI | 08011 | F | 10 | 4 |
| Pemba | 2013 | Chake Chake | 26/2/2013 | CHANJAMJAWIRI | 08012 | F | 12 | 4 |
| Pemba | 2013 | Chake Chake | 26/2/2013 | CHANJAMJAWIRI | 08013 | F | 12 | 4 |
| Pemba | 2013 | Chake Chake | 26/2/2013 | CHANJAMJAWIRI | 08014 | F | 10 | 4 |
| Pemba | 2013 | Chake Chake | 26/2/2013 | CHANJAMJAWIRI | 08015 | F | 10 | 4 |
| Pemba | 2013 | Chake Chake | 26/2/2013 | CHANJAMJAWIRI | 08016 | F | 11 | 4 |
| Pemba | 2013 | Chake Chake | 26/2/2013 | CHANJAMJAWIRI | 08017 | F | 10 | 4 |
| Pemba | 2013 | Chake Chake | 26/2/2013 | CHANJAMJAWIRI | 08018 | F | 10 | 4 |
| Pemba | 2013 | Chake Chake | 26/2/2013 | CHANJAMJAWIRI | 08019 | F | 10 | 4 |
| Pemba | 2013 | Chake Chake | 26/2/2013 | CHANJAMJAWIRI | 08020 | F | 10 | 4 |
| Pemba | 2013 | Chake Chake | 26/2/2013 | CHANJAMJAWIRI | 08021 | F | 12 | 4 |
| Pemba | 2013 | Chake Chake | 26/2/2013 | CHANJAMJAWIRI | 08022 | F | 11 | 4 |
| Pemba | 2013 | Chake Chake | 26/2/2013 | CHANJAMJAWIRI | 08023 | F | 12 | 4 |
| Pemba | 2013 | Chake Chake | 26/2/2013 | CHANJAMJAWIRI | 08024 | F | 11 | 4 |
| Pemba | 2013 | Chake Chake | 26/2/2013 | CHANJAMJAWIRI | 08025 | F | 10 | 4 |
| Pemba | 2013 | Chake Chake | 26/2/2013 | CHANJAMJAWIRI | 08026 | F | 12 | 4 |
| Pemba | 2013 | Chake Chake | 26/2/2013 | CHANJAMJAWIRI | 08027 | F | 10 | 4 |
| Pemba | 2013 | Chake Chake | 26/2/2013 | CHANJAMJAWIRI | 08028 | F | 11 | 4 |
| Pemba | 2013 | Chake Chake | 26/2/2013 | CHANJAMJAWIRI | 08029 | F | 12 | 4 |
| Pemba | 2013 | Chake Chake | 26/2/2013 | CHANJAMJAWIRI | 08030 | F | 11 | 4 |
| Pemba | 2013 | Chake Chake | 26/2/2013 | CHANJAMJAWIRI | 08031 | F | 11 | 4 |
| Pemba | 2013 | Chake Chake | 26/2/2013 | CHANJAMJAWIRI | 08032 | F | 10 | 4 |
| Pemba | 2013 | Chake Chake | 26/2/2013 | CHANJAMJAWIRI | 08033 | F | 11 | 4 |
| Pemba | 2013 | Chake Chake | 26/2/2013 | CHANJAMJAWIRI | 08034 | F | 11 | 4 |
| Pemba | 2013 | Chake Chake | 26/2/2013 | CHANJAMJAWIRI | 08035 | F | 11 | 4 |
| Pemba | 2013 | Chake Chake | 26/2/2013 | CHANJAMJAWIRI | 08036 | F | 11 | 4 |
| Pemba | 2013 | Chake Chake | 26/2/2013 | CHANJAMJAWIRI | 08037 | F | 11 | 4 |
| Pemba | 2013 | Chake Chake | 26/2/2013 | CHANJAMJAWIRI | 08038 | F | 11 | 4 |
| Pemba | 2013 | Chake Chake | 26/2/2013 | CHANJAMJAWIRI | 08039 | F | 11 | 4 |
| Pemba | 2013 | Chake Chake | 26/2/2013 | CHANJAMJAWIRI | 08040 | F | 11 | 4 |
| Pemba | 2013 | Chake Chake | 26/2/2013 | CHANJAMJAWIRI | 08041 | F | 11 | 4 |
| Pemba | 2013 | Chake Chake | 26/2/2013 | CHANJAMJAWIRI | 08042 | F | 11 | 4 |

| | | | | | | | | |
|---|---|---|---|---|---|---|---|---|
| Pemba | 2013 | Chake Chake | 26/2/2013 | CHANJAMJAWIRI | 08043 | F | 12 | 4 |
| Pemba | 2013 | Chake Chake | 26/2/2013 | CHANJAMJAWIRI | 08044 | F | 11 | 4 |
| Pemba | 2013 | Chake Chake | 26/2/2013 | CHANJAMJAWIRI | 08045 | F | 12 | 4 |
| Pemba | 2013 | Chake Chake | 26/2/2013 | CHANJAMJAWIRI | 08046 | F | 10 | 4 |
| Pemba | 2013 | Chake Chake | 26/2/2013 | CHANJAMJAWIRI | 08047 | M | 12 | 4 |
| Pemba | 2013 | Chake Chake | 26/2/2013 | CHANJAMJAWIRI | 08048 | M | 11 | 4 |
| Pemba | 2013 | Chake Chake | 26/2/2013 | CHANJAMJAWIRI | 08049 | M | 12 | 4 |
| Pemba | 2013 | Chake Chake | 26/2/2013 | CHANJAMJAWIRI | 08050 | M | 11 | 4 |
| Pemba | 2013 | Chake Chake | 26/2/2013 | CHANJAMJAWIRI | 08051 | M | 11 | 4 |
| Pemba | 2013 | Chake Chake | 26/2/2013 | CHANJAMJAWIRI | 08052 | M | 12 | 4 |
| Pemba | 2013 | Chake Chake | 26/2/2013 | CHANJAMJAWIRI | 08053 | M | 11 | 4 |
| Pemba | 2013 | Chake Chake | 26/2/2013 | CHANJAMJAWIRI | 08054 | M | 11 | 4 |
| Pemba | 2013 | Chake Chake | 26/2/2013 | CHANJAMJAWIRI | 08055 | M | 11 | 4 |
| Pemba | 2013 | Chake Chake | 26/2/2013 | CHANJAMJAWIRI | 08056 | M | 12 | 4 |
| Pemba | 2013 | Chake Chake | 26/2/2013 | CHANJAMJAWIRI | 08057 | M | 11 | 4 |
| Pemba | 2013 | Chake Chake | 26/2/2013 | CHANJAMJAWIRI | 08058 | M | 11 | 4 |
| Pemba | 2013 | Chake Chake | 26/2/2013 | CHANJAMJAWIRI | 08059 | M | 12 | 4 |
| Pemba | 2013 | Chake Chake | 26/2/2013 | CHANJAMJAWIRI | 08060 | M | 11 | 4 |
| Pemba | 2013 | Chake Chake | 26/2/2013 | CHANJAMJAWIRI | 08061 | M | 10 | 4 |
| Pemba | 2013 | Chake Chake | 26/2/2013 | CHANJAMJAWIRI | 08062 | M | 11 | 4 |
| Pemba | 2013 | Chake Chake | 26/2/2013 | CHANJAMJAWIRI | 08063 | M | 12 | 4 |
| Pemba | 2013 | Chake Chake | 26/2/2013 | CHANJAMJAWIRI | 08064 | M | 11 | 4 |
| Pemba | 2013 | Chake Chake | 26/2/2013 | CHANJAMJAWIRI | 08065 | M | 12 | 4 |
| Pemba | 2013 | Chake Chake | 26/2/2013 | CHANJAMJAWIRI | 08066 | M | 11 | 4 |
| Pemba | 2013 | Chake Chake | 26/2/2013 | CHANJAMJAWIRI | 08067 | M | 12 | 4 |
| Pemba | 2013 | Chake Chake | 26/2/2013 | CHANJAMJAWIRI | 08068 | M | 12 | 4 |
| Pemba | 2013 | Chake Chake | 26/2/2013 | CHANJAMJAWIRI | 08069 | M | 11 | 4 |
| Pemba | 2013 | Chake Chake | 26/2/2013 | CHANJAMJAWIRI | 08070 | M | 12 | 4 |
| Pemba | 2013 | Chake Chake | 26/2/2013 | CHANJAMJAWIRI | 08071 | M | 11 | 4 |
| Pemba | 2013 | Chake Chake | 26/2/2013 | CHANJAMJAWIRI | 08072 | M | 12 | 4 |
| Pemba | 2013 | Chake Chake | 26/2/2013 | CHANJAMJAWIRI | 08073 | M | 11 | 4 |
| Pemba | 2013 | Chake Chake | 26/2/2013 | CHANJAMJAWIRI | 08074 | M | 10 | 4 |
| Pemba | 2013 | Chake Chake | 26/2/2013 | CHANJAMJAWIRI | 08075 | M | 12 | 4 |
| Pemba | 2013 | Chake Chake | 26/2/2013 | CHANJAMJAWIRI | 08076 | M | 12 | 4 |
| Pemba | 2013 | Chake Chake | 26/2/2013 | CHANJAMJAWIRI | 08077 | M | 10 | 4 |
| Pemba | 2013 | Chake Chake | 26/2/2013 | CHANJAMJAWIRI | 08078 | M | 12 | 4 |
| Pemba | 2013 | Chake Chake | 26/2/2013 | CHANJAMJAWIRI | 08079 | M | 12 | 4 |
| Pemba | 2013 | Chake Chake | 26/2/2013 | CHANJAMJAWIRI | 08080 | M | 12 | 4 |
| Pemba | 2013 | Chake Chake | 26/2/2013 | CHANJAMJAWIRI | 08081 | M | 11 | 4 |
| Pemba | 2013 | Chake Chake | 26/2/2013 | CHANJAMJAWIRI | 08082 | M | 10 | 4 |
| Pemba | 2013 | Chake Chake | 26/2/2013 | CHANJAMJAWIRI | 08083 | M | 10 | 4 |
| Pemba | 2013 | Chake Chake | 26/2/2013 | CHANJAMJAWIRI | 08084 | M | 12 | 4 |
| Pemba | 2013 | Chake Chake | 26/2/2013 | CHANJAMJAWIRI | 08085 | M | 11 | 4 |
| Pemba | 2013 | Chake Chake | 26/2/2013 | CHANJAMJAWIRI | 08086 | M | 12 | 4 |
| Pemba | 2013 | Chake Chake | 26/2/2013 | CHANJAMJAWIRI | 08087 | M | 12 | 4 |
| Pemba | 2013 | Chake Chake | 26/2/2013 | CHANJAMJAWIRI | 08088 | M | 11 | 4 |
| Pemba | 2013 | Chake Chake | 26/2/2013 | CHANJAMJAWIRI | 08089 | M | 12 | 4 |
| Pemba | 2013 | Chake Chake | 26/2/2013 | CHANJAMJAWIRI | 08090 | M | 11 | 4 |
| Pemba | 2013 | Chake Chake | 26/2/2013 | CHANJAMJAWIRI | 08091 | M | 11 | 4 |
| Pemba | 2013 | Chake Chake | 26/2/2013 | CHANJAMJAWIRI | 08092 | M | 11 | 4 |

| | | | | | | | | |
|---|---|---|---|---|---|---|---|---|
| Pemba | 2013 | Chake Chake | 26/2/2013 | CHANJAMJAWIRI | 08093 | M | 11 | 4 |
| Pemba | 2013 | Chake Chake | 26/2/2013 | CHANJAMJAWIRI | 08094 | M | 12 | 4 |
| Pemba | 2013 | Chake Chake | 26/2/2013 | CHANJAMJAWIRI | 08095 | M | 11 | 4 |
| Pemba | 2013 | Chake Chake | 26/2/2013 | CHANJAMJAWIRI | 08096 | M | 10 | 4 |
| Pemba | 2013 | Chake Chake | 26/2/2013 | CHANJAMJAWIRI | 08097 | M | 11 | 4 |
| Pemba | 2013 | Chake Chake | 26/2/2013 | CHANJAMJAWIRI | 08098 | M | 11 | 4 |
| Pemba | 2013 | Chake Chake | 26/2/2013 | CHANJAMJAWIRI | 08099 | M | 11 | 4 |
| Pemba | 2013 | Chake Chake | 26/2/2013 | CHANJAMJAWIRI | 08100 | M | 11 | 4 |
| Pemba | 2013 | Chake Chake | 26/2/2013 | CHANJAMJAWIRI | 08101 | F | 10 | 3 |
| Pemba | 2013 | Chake Chake | 26/2/2013 | CHANJAMJAWIRI | 08102 | F | 9 | 3 |
| Pemba | 2013 | Chake Chake | 26/2/2013 | CHANJAMJAWIRI | 08103 | F | 10 | 3 |
| Pemba | 2013 | Chake Chake | 26/2/2013 | CHANJAMJAWIRI | 08104 | F | 9 | 3 |
| Pemba | 2013 | Chake Chake | 26/2/2013 | CHANJAMJAWIRI | 08105 | F | 9 | 3 |
| Pemba | 2013 | Chake Chake | 26/2/2013 | CHANJAMJAWIRI | 08106 | F | 9 | 3 |
| Pemba | 2013 | Chake Chake | 26/2/2013 | CHANJAMJAWIRI | 08107 | F | 9 | 3 |
| Pemba | 2013 | Chake Chake | 26/2/2013 | CHANJAMJAWIRI | 08108 | F | 9 | 3 |
| Pemba | 2013 | Chake Chake | 26/2/2013 | CHANJAMJAWIRI | 08109 | F | 10 | 3 |
| Pemba | 2013 | Chake Chake | 26/2/2013 | CHANJAMJAWIRI | 08110 | F | 9 | 3 |
| Pemba | 2013 | Chake Chake | 26/2/2013 | CHANJAMJAWIRI | 08111 | F | 9 | 3 |
| Pemba | 2013 | Chake Chake | 26/2/2013 | CHANJAMJAWIRI | 08112 | F | 9 | 3 |
| Pemba | 2013 | Chake Chake | 26/2/2013 | CHANJAMJAWIRI | 08113 | F | 9 | 3 |
| Pemba | 2013 | Chake Chake | 26/2/2013 | CHANJAMJAWIRI | 08114 | F | 10 | 3 |
| Pemba | 2013 | Chake Chake | 26/2/2013 | CHANJAMJAWIRI | 08115 | F | 9 | 3 |
| Pemba | 2013 | Chake Chake | 26/2/2013 | CHANJAMJAWIRI | 08116 | F | 9 | 3 |
| Pemba | 2013 | Chake Chake | 26/2/2013 | CHANJAMJAWIRI | 08117 | F | 10 | 3 |
| Pemba | 2013 | Chake Chake | 26/2/2013 | CHANJAMJAWIRI | 08118 | F | 9 | 3 |
| Pemba | 2013 | Chake Chake | 26/2/2013 | CHANJAMJAWIRI | 08119 | F | 9 | 3 |
| Pemba | 2013 | Chake Chake | 26/2/2013 | CHANJAMJAWIRI | 08120 | F | 9 | 3 |
| Pemba | 2013 | Chake Chake | 26/2/2013 | CHANJAMJAWIRI | 08121 | F | 10 | 3 |
| Pemba | 2013 | Chake Chake | 26/2/2013 | CHANJAMJAWIRI | 08122 | M | 10 | 3 |
| Pemba | 2013 | Chake Chake | 26/2/2013 | CHANJAMJAWIRI | 08123 | M | 10 | 3 |
| Pemba | 2013 | Chake Chake | 26/2/2013 | CHANJAMJAWIRI | 08124 | M | 11 | 3 |
| Pemba | 2013 | Chake Chake | 26/2/2013 | CHANJAMJAWIRI | 08125 | M | 10 | 3 |
| Pemba | 2013 | Chake Chake | 26/2/2013 | CHANJAMJAWIRI | 08126 | M | 11 | 3 |
| Pemba | 2013 | Chake Chake | 26/2/2013 | CHANJAMJAWIRI | 08127 | M | 11 | 3 |
| Pemba | 2013 | Chake Chake | 26/2/2013 | CHANJAMJAWIRI | 08128 | M | 11 | 3 |
| Pemba | 2013 | Chake Chake | 26/2/2013 | CHANJAMJAWIRI | 08129 | M | 10 | 3 |
| Pemba | 2013 | Chake Chake | 26/2/2013 | CHANJAMJAWIRI | 08130 | M | 10 | 3 |
| Pemba | 2013 | Chake Chake | 27/3/2013 | MBUZINI | 10001 | F | 9 | 3 |
| Pemba | 2013 | Chake Chake | 27/3/2013 | MBUZINI | 10002 | F | 11 | 3 |
| Pemba | 2013 | Chake Chake | 27/3/2013 | MBUZINI | 10003 | F | 11 | 3 |
| Pemba | 2013 | Chake Chake | 27/3/2013 | MBUZINI | 10004 | F | 11 | 3 |
| Pemba | 2013 | Chake Chake | 27/3/2013 | MBUZINI | 10005 | F | 10 | 3 |
| Pemba | 2013 | Chake Chake | 27/3/2013 | MBUZINI | 10006 | F | 12 | 3 |
| Pemba | 2013 | Chake Chake | 27/3/2013 | MBUZINI | 10007 | F | 11 | 3 |
| Pemba | 2013 | Chake Chake | 27/3/2013 | MBUZINI | 10008 | F | 11 | 3 |
| Pemba | 2013 | Chake Chake | 27/3/2013 | MBUZINI | 10009 | F | 9 | 3 |
| Pemba | 2013 | Chake Chake | 27/3/2013 | MBUZINI | 10010 | F | 12 | 3 |
| Pemba | 2013 | Chake Chake | 27/3/2013 | MBUZINI | 10011 | F | 12 | 3 |
| Pemba | 2013 | Chake Chake | 27/3/2013 | MBUZINI | 10012 | F | 9 | 3 |

| | | | | | | | | |
|---|---|---|---|---|---|---|---|---|
| Pemba | 2013 | Chake Chake | 27/3/2013 | MBUZINI | 10013 | F | 9 | 3 |
| Pemba | 2013 | Chake Chake | 27/3/2013 | MBUZINI | 10014 | F | 12 | 3 |
| Pemba | 2013 | Chake Chake | 27/3/2013 | MBUZINI | 10015 | F | 11 | 3 |
| Pemba | 2013 | Chake Chake | 27/3/2013 | MBUZINI | 10016 | F | 10 | 3 |
| Pemba | 2013 | Chake Chake | 27/3/2013 | MBUZINI | 10017 | F | 9 | 3 |
| Pemba | 2013 | Chake Chake | 27/3/2013 | MBUZINI | 10018 | F | 11 | 3 |
| Pemba | 2013 | Chake Chake | 27/3/2013 | MBUZINI | 10019 | F | 9 | 3 |
| Pemba | 2013 | Chake Chake | 27/3/2013 | MBUZINI | 10020 | F | 11 | 3 |
| Pemba | 2013 | Chake Chake | 27/3/2013 | MBUZINI | 10021 | F | 9 | 3 |
| Pemba | 2013 | Chake Chake | 27/3/2013 | MBUZINI | 10022 | F | 11 | 3 |
| Pemba | 2013 | Chake Chake | 27/3/2013 | MBUZINI | 10023 | F | 11 | 3 |
| Pemba | 2013 | Chake Chake | 27/3/2013 | MBUZINI | 10024 | F | 8 | 3 |
| Pemba | 2013 | Chake Chake | 27/3/2013 | MBUZINI | 10025 | F | 11 | 3 |
| Pemba | 2013 | Chake Chake | 27/3/2013 | MBUZINI | 10026 | F | 11 | 3 |
| Pemba | 2013 | Chake Chake | 27/3/2013 | MBUZINI | 10027 | F | 10 | 3 |
| Pemba | 2013 | Chake Chake | 27/3/2013 | MBUZINI | 10028 | F | 12 | 3 |
| Pemba | 2013 | Chake Chake | 27/3/2013 | MBUZINI | 10029 | F | 11 | 3 |
| Pemba | 2013 | Chake Chake | 27/3/2013 | MBUZINI | 10030 | F | 10 | 3 |
| Pemba | 2013 | Chake Chake | 27/3/2013 | MBUZINI | 10031 | F | 10 | 3 |
| Pemba | 2013 | Chake Chake | 27/3/2013 | MBUZINI | 10032 | F | 9 | 3 |
| Pemba | 2013 | Chake Chake | 27/3/2013 | MBUZINI | 10033 | F | 8 | 3 |
| Pemba | 2013 | Chake Chake | 27/3/2013 | MBUZINI | 10034 | M | 9 | 3 |
| Pemba | 2013 | Chake Chake | 27/3/2013 | MBUZINI | 10035 | M | 10 | 3 |
| Pemba | 2013 | Chake Chake | 27/3/2013 | MBUZINI | 10036 | M | 10 | 3 |
| Pemba | 2013 | Chake Chake | 27/3/2013 | MBUZINI | 10037 | M | 12 | 3 |
| Pemba | 2013 | Chake Chake | 27/3/2013 | MBUZINI | 10038 | M | 11 | 3 |
| Pemba | 2013 | Chake Chake | 27/3/2013 | MBUZINI | 10039 | M | 11 | 3 |
| Pemba | 2013 | Chake Chake | 27/3/2013 | MBUZINI | 10040 | M | 11 | 3 |
| Pemba | 2013 | Chake Chake | 27/3/2013 | MBUZINI | 10041 | M | 11 | 3 |
| Pemba | 2013 | Chake Chake | 27/3/2013 | MBUZINI | 10042 | M | 12 | 3 |
| Pemba | 2013 | Chake Chake | 27/3/2013 | MBUZINI | 10043 | M | 10 | 3 |
| Pemba | 2013 | Chake Chake | 27/3/2013 | MBUZINI | 10044 | M | 10 | 3 |
| Pemba | 2013 | Chake Chake | 27/3/2013 | MBUZINI | 10045 | M | 9 | 3 |
| Pemba | 2013 | Chake Chake | 27/3/2013 | MBUZINI | 10046 | M | 10 | 3 |
| Pemba | 2013 | Chake Chake | 27/3/2013 | MBUZINI | 10047 | M | 10 | 3 |
| Pemba | 2013 | Chake Chake | 27/3/2013 | MBUZINI | 10048 | M | 9 | 3 |
| Pemba | 2013 | Chake Chake | 27/3/2013 | MBUZINI | 10049 | M | 12 | 3 |
| Pemba | 2013 | Chake Chake | 27/3/2013 | MBUZINI | 10050 | M | 10 | 3 |
| Pemba | 2013 | Chake Chake | 27/3/2013 | MBUZINI | 10051 | M | 10 | 3 |
| Pemba | 2013 | Chake Chake | 27/3/2013 | MBUZINI | 10052 | M | 12 | 3 |
| Pemba | 2013 | Chake Chake | 27/3/2013 | MBUZINI | 10053 | M | 10 | 3 |
| Pemba | 2013 | Chake Chake | 27/3/2013 | MBUZINI | 10054 | M | 10 | 3 |
| Pemba | 2013 | Chake Chake | 27/3/2013 | MBUZINI | 10055 | M | 11 | 3 |
| Pemba | 2013 | Chake Chake | 27/3/2013 | MBUZINI | 10056 | M | 10 | 3 |
| Pemba | 2013 | Chake Chake | 27/3/2013 | MBUZINI | 10057 | M | 10 | 3 |
| Pemba | 2013 | Chake Chake | 27/3/2013 | MBUZINI | 10058 | M | 10 | 3 |
| Pemba | 2013 | Chake Chake | 27/3/2013 | MBUZINI | 10059 | M | 10 | 3 |
| Pemba | 2013 | Chake Chake | 27/3/2013 | MBUZINI | 10060 | M | 10 | 3 |
| Pemba | 2013 | Chake Chake | 27/3/2013 | MBUZINI | 10061 | M | 12 | 3 |
| Pemba | 2013 | Chake Chake | 27/3/2013 | MBUZINI | 10062 | M | 11 | 3 |

| | | | | | | | | |
|---|---|---|---|---|---|---|---|---|
| Pemba | 2013 | Chake Chake | 27/3/2013 | MBUZINI | 10063 | M | 10 | 3 |
| Pemba | 2013 | Chake Chake | 27/3/2013 | MBUZINI | 10064 | M | 11 | 3 |
| Pemba | 2013 | Chake Chake | 27/3/2013 | MBUZINI | 10065 | M | 11 | 4 |
| Pemba | 2013 | Chake Chake | 27/3/2013 | MBUZINI | 10066 | M | 10 | 4 |
| Pemba | 2013 | Chake Chake | 27/3/2013 | MBUZINI | 10067 | M | 11 | 4 |
| Pemba | 2013 | Chake Chake | 27/3/2013 | MBUZINI | 10068 | M | 10 | 4 |
| Pemba | 2013 | Chake Chake | 27/3/2013 | MBUZINI | 10069 | M | 12 | 4 |
| Pemba | 2013 | Chake Chake | 27/3/2013 | MBUZINI | 10070 | M | 12 | 4 |
| Pemba | 2013 | Chake Chake | 27/3/2013 | MBUZINI | 10071 | M | 11 | 4 |
| Pemba | 2013 | Chake Chake | 27/3/2013 | MBUZINI | 10072 | M | 12 | 4 |
| Pemba | 2013 | Chake Chake | 27/3/2013 | MBUZINI | 10073 | M | 12 | 4 |
| Pemba | 2013 | Chake Chake | 27/3/2013 | MBUZINI | 10074 | M | 12 | 4 |
| Pemba | 2013 | Chake Chake | 27/3/2013 | MBUZINI | 10075 | M | 12 | 4 |
| Pemba | 2013 | Chake Chake | 27/3/2013 | MBUZINI | 10076 | M | 10 | 4 |
| Pemba | 2013 | Chake Chake | 27/3/2013 | MBUZINI | 10077 | M | 11 | 4 |
| Pemba | 2013 | Chake Chake | 27/3/2013 | MBUZINI | 10078 | M | 10 | 4 |
| Pemba | 2013 | Chake Chake | 27/3/2013 | MBUZINI | 10079 | M | 12 | 4 |
| Pemba | 2013 | Chake Chake | 27/3/2013 | MBUZINI | 10080 | M | 11 | 4 |
| Pemba | 2013 | Chake Chake | 27/3/2013 | MBUZINI | 10081 | M | 11 | 4 |
| Pemba | 2013 | Chake Chake | 27/3/2013 | MBUZINI | 10082 | M | 12 | 4 |
| Pemba | 2013 | Chake Chake | 27/3/2013 | MBUZINI | 10083 | M | 11 | 4 |
| Pemba | 2013 | Chake Chake | 27/3/2013 | MBUZINI | 10084 | M | 10 | 4 |
| Pemba | 2013 | Chake Chake | 27/3/2013 | MBUZINI | 10085 | M | 11 | 4 |
| Pemba | 2013 | Chake Chake | 27/3/2013 | MBUZINI | 10086 | M | 12 | 4 |
| Pemba | 2013 | Chake Chake | 27/3/2013 | MBUZINI | 10087 | M | 11 | 4 |
| Pemba | 2013 | Chake Chake | 27/3/2013 | MBUZINI | 10088 | M | 12 | 4 |
| Pemba | 2013 | Chake Chake | 27/3/2013 | MBUZINI | 10089 | M | 11 | 4 |
| Pemba | 2013 | Chake Chake | 27/3/2013 | MBUZINI | 10090 | M | 11 | 4 |
| Pemba | 2013 | Chake Chake | 27/3/2013 | MBUZINI | 10091 | M | 12 | 4 |
| Pemba | 2013 | Chake Chake | 27/3/2013 | MBUZINI | 10092 | M | 12 | 4 |
| Pemba | 2013 | Chake Chake | 27/3/2013 | MBUZINI | 10093 | M | 12 | 4 |
| Pemba | 2013 | Chake Chake | 27/3/2013 | MBUZINI | 10094 | M | 12 | 4 |
| Pemba | 2013 | Chake Chake | 27/3/2013 | MBUZINI | 10095 | M | 12 | 4 |
| Pemba | 2013 | Chake Chake | 27/3/2013 | MBUZINI | 10096 | M | 11 | 4 |
| Pemba | 2013 | Chake Chake | 27/3/2013 | MBUZINI | 10097 | M | 11 | 4 |
| Pemba | 2013 | Chake Chake | 27/3/2013 | MBUZINI | 10098 | M | 11 | 4 |
| Pemba | 2013 | Chake Chake | 27/3/2013 | MBUZINI | 10099 | M | 11 | 4 |
| Pemba | 2013 | Chake Chake | 27/3/2013 | MBUZINI | 10100 | M | 10 | 4 |
| Pemba | 2013 | Chake Chake | 27/3/2013 | MBUZINI | 10101 | F | 12 | 4 |
| Pemba | 2013 | Chake Chake | 27/3/2013 | MBUZINI | 10102 | F | 12 | 4 |
| Pemba | 2013 | Chake Chake | 27/3/2013 | MBUZINI | 10103 | F | 11 | 4 |
| Pemba | 2013 | Chake Chake | 27/3/2013 | MBUZINI | 10104 | F | 12 | 4 |
| Pemba | 2013 | Chake Chake | 27/3/2013 | MBUZINI | 10105 | F | 10 | 4 |
| Pemba | 2013 | Chake Chake | 27/3/2013 | MBUZINI | 10106 | F | 10 | 4 |
| Pemba | 2013 | Chake Chake | 27/3/2013 | MBUZINI | 10107 | F | 11 | 4 |
| Pemba | 2013 | Chake Chake | 27/3/2013 | MBUZINI | 10108 | F | 11 | 4 |
| Pemba | 2013 | Chake Chake | 27/3/2013 | MBUZINI | 10109 | F | 10 | 4 |
| Pemba | 2013 | Chake Chake | 27/3/2013 | MBUZINI | 10110 | F | 10 | 4 |
| Pemba | 2013 | Chake Chake | 27/3/2013 | MBUZINI | 10111 | F | 10 | 4 |
| Pemba | 2013 | Chake Chake | 27/3/2013 | MBUZINI | 10112 | F | 12 | 4 |

| | | | | | | | | |
|---|---|---|---|---|---|---|---|---|
| Pemba | 2013 | Chake Chake | 27/3/2013 | MBUZINI | 10113 | F | 11 | 4 |
| Pemba | 2013 | Chake Chake | 27/3/2013 | MBUZINI | 10114 | F | 10 | 4 |
| Pemba | 2013 | Chake Chake | 27/3/2013 | MBUZINI | 10115 | F | 9 | 4 |
| Pemba | 2013 | Chake Chake | 27/3/2013 | MBUZINI | 10116 | F | 12 | 4 |
| Pemba | 2013 | Chake Chake | 27/3/2013 | MBUZINI | 10117 | F | 12 | 4 |
| Pemba | 2013 | Chake Chake | 27/3/2013 | MBUZINI | 10118 | F | 10 | 4 |
| Pemba | 2013 | Chake Chake | 27/3/2013 | MBUZINI | 10119 | F | 12 | 4 |
| Pemba | 2013 | Chake Chake | 27/3/2013 | MBUZINI | 10120 | F | 12 | 4 |
| Pemba | 2013 | Chake Chake | 27/3/2013 | MBUZINI | 10121 | F | 11 | 4 |
| Pemba | 2013 | Chake Chake | 27/3/2013 | MBUZINI | 10122 | F | 11 | 4 |
| Pemba | 2013 | Chake Chake | 27/3/2013 | MBUZINI | 10123 | F | 11 | 4 |
| Pemba | 2013 | Chake Chake | 27/3/2013 | MBUZINI | 10124 | F | 11 | 4 |
| Pemba | 2013 | Chake Chake | 27/3/2013 | MBUZINI | 10125 | F | 12 | 4 |
| Pemba | 2013 | Chake Chake | 27/3/2013 | MBUZINI | 10126 | F | 10 | 4 |
| Pemba | 2013 | Chake Chake | 27/3/2013 | MBUZINI | 10127 | F | 11 | 4 |
| Pemba | 2013 | Chake Chake | 27/3/2013 | MBUZINI | 10128 | F | 10 | 4 |
| Pemba | 2013 | Chake Chake | 27/3/2013 | MBUZINI | 10129 | M | 11 | 4 |
| Pemba | 2013 | Chake Chake | 27/3/2013 | MBUZINI | 10130 | M | 10 | 4 |
| Pemba | 2013 | Mkoani | 19/1/2013 | NG'OMBENI | 16001 | F | 9 | 3 |
| Pemba | 2013 | Mkoani | 19/1/2013 | NG'OMBENI | 16002 | F | 10 | 3 |
| Pemba | 2013 | Mkoani | 19/1/2013 | NG'OMBENI | 16003 | F | 10 | 3 |
| Pemba | 2013 | Mkoani | 19/1/2013 | NG'OMBENI | 16004 | F | 9 | 3 |
| Pemba | 2013 | Mkoani | 19/1/2013 | NG'OMBENI | 16005 | F | 10 | 3 |
| Pemba | 2013 | Mkoani | 19/1/2013 | NG'OMBENI | 16006 | F | 9 | 3 |
| Pemba | 2013 | Mkoani | 19/1/2013 | NG'OMBENI | 16007 | F | 10 | 3 |
| Pemba | 2013 | Mkoani | 19/1/2013 | NG'OMBENI | 16008 | F | 9 | 3 |
| Pemba | 2013 | Mkoani | 19/1/2013 | NG'OMBENI | 16009 | F | 10 | 3 |
| Pemba | 2013 | Mkoani | 19/1/2013 | NG'OMBENI | 16010 | F | 9 | 3 |
| Pemba | 2013 | Mkoani | 19/1/2013 | NG'OMBENI | 16011 | F | 10 | 3 |
| Pemba | 2013 | Mkoani | 19/1/2013 | NG'OMBENI | 16012 | F | 9 | 3 |
| Pemba | 2013 | Mkoani | 19/1/2013 | NG'OMBENI | 16013 | F | 9 | 3 |
| Pemba | 2013 | Mkoani | 19/1/2013 | NG'OMBENI | 16014 | F | 9 | 3 |
| Pemba | 2013 | Mkoani | 19/1/2013 | NG'OMBENI | 16015 | F | 9 | 3 |
| Pemba | 2013 | Mkoani | 19/1/2013 | NG'OMBENI | 16016 | F | 10 | 3 |
| Pemba | 2013 | Mkoani | 19/1/2013 | NG'OMBENI | 16017 | F | 9 | 3 |
| Pemba | 2013 | Mkoani | 19/1/2013 | NG'OMBENI | 16018 | F | 9 | 3 |
| Pemba | 2013 | Mkoani | 19/1/2013 | NG'OMBENI | 16019 | F | 9 | 3 |
| Pemba | 2013 | Mkoani | 19/1/2013 | NG'OMBENI | 16020 | F | 10 | 3 |
| Pemba | 2013 | Mkoani | 19/1/2013 | NG'OMBENI | 16021 | F | 10 | 3 |
| Pemba | 2013 | Mkoani | 19/1/2013 | NG'OMBENI | 16022 | F | 10 | 3 |
| Pemba | 2013 | Mkoani | 19/1/2013 | NG'OMBENI | 16023 | F | 10 | 3 |
| Pemba | 2013 | Mkoani | 19/1/2013 | NG'OMBENI | 16024 | F | 10 | 3 |
| Pemba | 2013 | Mkoani | 19/1/2013 | NG'OMBENI | 16025 | F | 10 | 3 |
| Pemba | 2013 | Mkoani | 19/1/2013 | NG'OMBENI | 16026 | F | 9 | 3 |
| Pemba | 2013 | Mkoani | 19/1/2013 | NG'OMBENI | 16027 | F | 10 | 3 |
| Pemba | 2013 | Mkoani | 19/1/2013 | NG'OMBENI | 16028 | F | 10 | 3 |
| Pemba | 2013 | Mkoani | 19/1/2013 | NG'OMBENI | 16029 | F | 10 | 3 |
| Pemba | 2013 | Mkoani | 19/1/2013 | NG'OMBENI | 16030 | F | 9 | 3 |
| Pemba | 2013 | Mkoani | 19/1/2013 | NG'OMBENI | 16031 | F | 10 | 3 |
| Pemba | 2013 | Mkoani | 19/1/2013 | NG'OMBENI | 16032 | F | 10 | 3 |

| | | | | | | | | |
|---|---|---|---|---|---|---|---|---|
| Pemba | 2013 | Mkoani | 19/1/2013 | NG'OMBENI | 16033 | F | 10 | 3 |
| Pemba | 2013 | Mkoani | 19/1/2013 | NG'OMBENI | 16034 | F | 10 | 3 |
| Pemba | 2013 | Mkoani | 19/1/2013 | NG'OMBENI | 16035 | F | 9 | 3 |
| Pemba | 2013 | Mkoani | 19/1/2013 | NG'OMBENI | 16036 | F | 9 | 3 |
| Pemba | 2013 | Mkoani | 19/1/2013 | NG'OMBENI | 16037 | F | 10 | 3 |
| Pemba | 2013 | Mkoani | 19/1/2013 | NG'OMBENI | 16038 | F | 10 | 3 |
| Pemba | 2013 | Mkoani | 19/1/2013 | NG'OMBENI | 16039 | F | 10 | 3 |
| Pemba | 2013 | Mkoani | 19/1/2013 | NG'OMBENI | 16040 | F | 10 | 3 |
| Pemba | 2013 | Mkoani | 19/1/2013 | NG'OMBENI | 16041 | F | 10 | 3 |
| Pemba | 2013 | Mkoani | 19/1/2013 | NG'OMBENI | 16042 | F | 10 | 3 |
| Pemba | 2013 | Mkoani | 19/1/2013 | NG'OMBENI | 16043 | F | 10 | 3 |
| Pemba | 2013 | Mkoani | 19/1/2013 | NG'OMBENI | 16044 | F | 9 | 3 |
| Pemba | 2013 | Mkoani | 19/1/2013 | NG'OMBENI | 16045 | F | 10 | 3 |
| Pemba | 2013 | Mkoani | 19/1/2013 | NG'OMBENI | 16046 | F | 9 | 3 |
| Pemba | 2013 | Mkoani | 19/1/2013 | NG'OMBENI | 16047 | F | 11 | 3 |
| Pemba | 2013 | Mkoani | 19/1/2013 | NG'OMBENI | 16048 | F | 10 | 3 |
| Pemba | 2013 | Mkoani | 19/1/2013 | NG'OMBENI | 16049 | F | 10 | 3 |
| Pemba | 2013 | Mkoani | 19/1/2013 | NG'OMBENI | 16050 | F | 9 | 3 |
| Pemba | 2013 | Mkoani | 19/1/2013 | NG'OMBENI | 16051 | F | 9 | 3 |
| Pemba | 2013 | Mkoani | 19/1/2013 | NG'OMBENI | 16052 | F | 10 | 3 |
| Pemba | 2013 | Mkoani | 19/1/2013 | NG'OMBENI | 16053 | F | 9 | 3 |
| Pemba | 2013 | Mkoani | 19/1/2013 | NG'OMBENI | 16054 | F | 9 | 3 |
| Pemba | 2013 | Mkoani | 19/1/2013 | NG'OMBENI | 16055 | F | 9 | 3 |
| Pemba | 2013 | Mkoani | 19/1/2013 | NG'OMBENI | 16056 | F | 9 | 3 |
| Pemba | 2013 | Mkoani | 19/1/2013 | NG'OMBENI | 16057 | F | 10 | 3 |
| Pemba | 2013 | Mkoani | 19/1/2013 | NG'OMBENI | 16058 | F | 9 | 3 |
| Pemba | 2013 | Mkoani | 19/1/2013 | NG'OMBENI | 16059 | F | 9 | 3 |
| Pemba | 2013 | Mkoani | 19/1/2013 | NG'OMBENI | 16060 | F | 10 | 3 |
| Pemba | 2013 | Mkoani | 19/1/2013 | NG'OMBENI | 16061 | F | 10 | 3 |
| Pemba | 2013 | Mkoani | 19/1/2013 | NG'OMBENI | 16062 | M | 10 | 3 |
| Pemba | 2013 | Mkoani | 19/1/2013 | NG'OMBENI | 16063 | M | 9 | 3 |
| Pemba | 2013 | Mkoani | 19/1/2013 | NG'OMBENI | 16064 | M | 10 | 3 |
| Pemba | 2013 | Mkoani | 19/1/2013 | NG'OMBENI | 16065 | M | 10 | 3 |
| Pemba | 2013 | Mkoani | 19/1/2013 | NG'OMBENI | 16066 | M | 10 | 3 |
| Pemba | 2013 | Mkoani | 19/1/2013 | NG'OMBENI | 16067 | M | 9 | 3 |
| Pemba | 2013 | Mkoani | 19/1/2013 | NG'OMBENI | 16068 | M | 9 | 3 |
| Pemba | 2013 | Mkoani | 19/1/2013 | NG'OMBENI | 16069 | M | 10 | 3 |
| Pemba | 2013 | Mkoani | 19/1/2013 | NG'OMBENI | 16070 | M | 9 | 3 |
| Pemba | 2013 | Mkoani | 19/1/2013 | NG'OMBENI | 16071 | M | 9 | 3 |
| Pemba | 2013 | Mkoani | 19/1/2013 | NG'OMBENI | 16072 | M | 10 | 3 |
| Pemba | 2013 | Mkoani | 19/1/2013 | NG'OMBENI | 16073 | M | 9 | 3 |
| Pemba | 2013 | Mkoani | 19/1/2013 | NG'OMBENI | 16074 | M | 10 | 3 |
| Pemba | 2013 | Mkoani | 19/1/2013 | NG'OMBENI | 16075 | M | 9 | 3 |
| Pemba | 2013 | Mkoani | 19/1/2013 | NG'OMBENI | 16076 | M | 10 | 3 |
| Pemba | 2013 | Mkoani | 19/1/2013 | NG'OMBENI | 16077 | M | 9 | 3 |
| Pemba | 2013 | Mkoani | 19/1/2013 | NG'OMBENI | 16078 | M | 10 | 3 |
| Pemba | 2013 | Mkoani | 19/1/2013 | NG'OMBENI | 16079 | M | 9 | 3 |
| Pemba | 2013 | Mkoani | 19/1/2013 | NG'OMBENI | 16080 | M | 9 | 3 |
| Pemba | 2013 | Mkoani | 19/1/2013 | NG'OMBENI | 16081 | M | 10 | 3 |
| Pemba | 2013 | Mkoani | 19/1/2013 | NG'OMBENI | 16082 | M | 10 | 3 |

| | | | | | | | | |
|---|---|---|---|---|---|---|---|---|
| Pemba | 2013 | Mkoani | 19/1/2013 | NG'OMBENI | 16083 | M | 9 | 3 |
| Pemba | 2013 | Mkoani | 19/1/2013 | NG'OMBENI | 16084 | M | 10 | 3 |
| Pemba | 2013 | Mkoani | 19/1/2013 | NG'OMBENI | 16085 | M | 10 | 3 |
| Pemba | 2013 | Mkoani | 19/1/2013 | NG'OMBENI | 16086 | M | 9 | 3 |
| Pemba | 2013 | Mkoani | 19/1/2013 | NG'OMBENI | 16087 | M | 10 | 3 |
| Pemba | 2013 | Mkoani | 19/1/2013 | NG'OMBENI | 16088 | M | 9 | 3 |
| Pemba | 2013 | Mkoani | 19/1/2013 | NG'OMBENI | 16089 | M | 10 | 3 |
| Pemba | 2013 | Mkoani | 19/1/2013 | NG'OMBENI | 16090 | M | 10 | 3 |
| Pemba | 2013 | Mkoani | 19/1/2013 | NG'OMBENI | 16091 | M | 10 | 3 |
| Pemba | 2013 | Mkoani | 19/1/2013 | NG'OMBENI | 16092 | M | 10 | 3 |
| Pemba | 2013 | Mkoani | 19/1/2013 | NG'OMBENI | 16093 | M | 10 | 3 |
| Pemba | 2013 | Mkoani | 19/1/2013 | NG'OMBENI | 16094 | M | 9 | 3 |
| Pemba | 2013 | Mkoani | 19/1/2013 | NG'OMBENI | 16095 | M | 10 | 3 |
| Pemba | 2013 | Mkoani | 19/1/2013 | NG'OMBENI | 16096 | M | 9 | 3 |
| Pemba | 2013 | Mkoani | 19/1/2013 | NG'OMBENI | 16097 | M | 9 | 3 |
| Pemba | 2013 | Mkoani | 19/1/2013 | NG'OMBENI | 16098 | M | 9 | 3 |
| Pemba | 2013 | Mkoani | 19/1/2013 | NG'OMBENI | 16099 | M | 9 | 3 |
| Pemba | 2013 | Mkoani | 19/1/2013 | NG'OMBENI | 16100 | M | 10 | 3 |
| Pemba | 2013 | Mkoani | 19/1/2013 | NG'OMBENI | 16101 | M | 10 | 3 |
| Pemba | 2013 | Mkoani | 19/1/2013 | NG'OMBENI | 16102 | M | 10 | 3 |
| Pemba | 2013 | Mkoani | 19/1/2013 | NG'OMBENI | 16103 | M | 10 | 3 |
| Pemba | 2013 | Mkoani | 19/1/2013 | NG'OMBENI | 16104 | M | 9 | 3 |
| Pemba | 2013 | Mkoani | 19/1/2013 | NG'OMBENI | 16105 | M | 10 | 3 |
| Pemba | 2013 | Mkoani | 19/1/2013 | NG'OMBENI | 16106 | M | 9 | 3 |
| Pemba | 2013 | Mkoani | 19/1/2013 | NG'OMBENI | 16107 | M | 9 | 3 |
| Pemba | 2013 | Mkoani | 19/1/2013 | NG'OMBENI | 16108 | M | 9 | 3 |
| Pemba | 2013 | Mkoani | 19/1/2013 | NG'OMBENI | 16109 | M | 9 | 3 |
| Pemba | 2013 | Mkoani | 19/1/2013 | NG'OMBENI | 16110 | M | 10 | 3 |
| Pemba | 2013 | Mkoani | 19/1/2013 | NG'OMBENI | 16111 | M | 10 | 3 |
| Pemba | 2013 | Mkoani | 19/1/2013 | NG'OMBENI | 16112 | M | 10 | 3 |
| Pemba | 2013 | Mkoani | 19/1/2013 | NG'OMBENI | 16113 | M | 9 | 3 |
| Pemba | 2013 | Mkoani | 19/1/2013 | NG'OMBENI | 16114 | M | 9 | 3 |
| Pemba | 2013 | Mkoani | 19/1/2013 | NG'OMBENI | 16115 | M | 10 | 3 |
| Pemba | 2013 | Mkoani | 19/1/2013 | NG'OMBENI | 16116 | M | 10 | 3 |
| Pemba | 2013 | Mkoani | 19/1/2013 | NG'OMBENI | 16117 | M | 10 | 3 |
| Pemba | 2013 | Mkoani | 19/1/2013 | NG'OMBENI | 16118 | M | 10 | 3 |
| Pemba | 2013 | Mkoani | 19/1/2013 | NG'OMBENI | 16119 | M | 10 | 3 |
| Pemba | 2013 | Mkoani | 19/1/2013 | NG'OMBENI | 16120 | M | 9 | 3 |
| Pemba | 2013 | Mkoani | 19/1/2013 | NG'OMBENI | 16121 | M | 10 | 3 |
| Pemba | 2013 | Mkoani | 19/1/2013 | NG'OMBENI | 16122 | M | 9 | 3 |
| Pemba | 2013 | Mkoani | 19/1/2013 | NG'OMBENI | 16123 | M | 9 | 3 |
| Pemba | 2013 | Mkoani | 19/1/2013 | NG'OMBENI | 16124 | M | 9 | 3 |
| Pemba | 2013 | Mkoani | 19/1/2013 | NG'OMBENI | 16125 | M | 10 | 3 |
| Pemba | 2013 | Mkoani | 19/1/2013 | NG'OMBENI | 16126 | M | 10 | 3 |
| Pemba | 2013 | Mkoani | 19/1/2013 | NG'OMBENI | 16127 | M | 9 | 3 |
| Pemba | 2013 | Mkoani | 19/1/2013 | NG'OMBENI | 16128 | M | 10 | 3 |
| Pemba | 2013 | Mkoani | 19/1/2013 | NG'OMBENI | 16129 | M | 9 | 3 |
| Pemba | 2013 | Mkoani | 19/1/2013 | NG'OMBENI | 16130 | M | 9 | 3 |
| Pemba | 2013 | Mkoani | 20/2/2013 | MKANYAGENI | 17001 | F | 9 | 3 |
| Pemba | 2013 | Mkoani | 20/2/2013 | MKANYAGENI | 17002 | F | 9 | 3 |

| | | | | | | | | |
|---|---|---|---|---|---|---|---|---|
| Pemba | 2013 | Mkoani | 20/2/2013 | MKANYAGENI | 17003 | F | 9 | 3 |
| Pemba | 2013 | Mkoani | 20/2/2013 | MKANYAGENI | 17004 | F | 10 | 3 |
| Pemba | 2013 | Mkoani | 20/2/2013 | MKANYAGENI | 17005 | F | 12 | 3 |
| Pemba | 2013 | Mkoani | 20/2/2013 | MKANYAGENI | 17006 | F | 9 | 3 |
| Pemba | 2013 | Mkoani | 20/2/2013 | MKANYAGENI | 17007 | F | 11 | 3 |
| Pemba | 2013 | Mkoani | 20/2/2013 | MKANYAGENI | 17008 | F | 10 | 3 |
| Pemba | 2013 | Mkoani | 20/2/2013 | MKANYAGENI | 17009 | F | 10 | 3 |
| Pemba | 2013 | Mkoani | 20/2/2013 | MKANYAGENI | 17010 | F | 11 | 3 |
| Pemba | 2013 | Mkoani | 20/2/2013 | MKANYAGENI | 17011 | F | 11 | 3 |
| Pemba | 2013 | Mkoani | 20/2/2013 | MKANYAGENI | 17012 | F | 10 | 3 |
| Pemba | 2013 | Mkoani | 20/2/2013 | MKANYAGENI | 17013 | F | 10 | 3 |
| Pemba | 2013 | Mkoani | 20/2/2013 | MKANYAGENI | 17014 | F | 11 | 3 |
| Pemba | 2013 | Mkoani | 20/2/2013 | MKANYAGENI | 17015 | F | 10 | 3 |
| Pemba | 2013 | Mkoani | 20/2/2013 | MKANYAGENI | 17016 | F | 10 | 3 |
| Pemba | 2013 | Mkoani | 20/2/2013 | MKANYAGENI | 17017 | F | 10 | 3 |
| Pemba | 2013 | Mkoani | 20/2/2013 | MKANYAGENI | 17018 | F | 9 | 3 |
| Pemba | 2013 | Mkoani | 20/2/2013 | MKANYAGENI | 17019 | F | 11 | 3 |
| Pemba | 2013 | Mkoani | 20/2/2013 | MKANYAGENI | 17020 | F | 12 | 3 |
| Pemba | 2013 | Mkoani | 20/2/2013 | MKANYAGENI | 17021 | F | 10 | 3 |
| Pemba | 2013 | Mkoani | 20/2/2013 | MKANYAGENI | 17022 | F | 10 | 3 |
| Pemba | 2013 | Mkoani | 20/2/2013 | MKANYAGENI | 17023 | F | 9 | 3 |
| Pemba | 2013 | Mkoani | 20/2/2013 | MKANYAGENI | 17024 | F | 10 | 3 |
| Pemba | 2013 | Mkoani | 20/2/2013 | MKANYAGENI | 17025 | F | 10 | 3 |
| Pemba | 2013 | Mkoani | 20/2/2013 | MKANYAGENI | 17026 | F | 10 | 3 |
| Pemba | 2013 | Mkoani | 20/2/2013 | MKANYAGENI | 17027 | F | 11 | 3 |
| Pemba | 2013 | Mkoani | 20/2/2013 | MKANYAGENI | 17028 | F | 11 | 3 |
| Pemba | 2013 | Mkoani | 20/2/2013 | MKANYAGENI | 17029 | F | 11 | 3 |
| Pemba | 2013 | Mkoani | 20/2/2013 | MKANYAGENI | 17030 | F | 10 | 3 |
| Pemba | 2013 | Mkoani | 20/2/2013 | MKANYAGENI | 17031 | F | 10 | 3 |
| Pemba | 2013 | Mkoani | 20/2/2013 | MKANYAGENI | 17032 | F | 12 | 3 |
| Pemba | 2013 | Mkoani | 20/2/2013 | MKANYAGENI | 17033 | F | 9 | 3 |
| Pemba | 2013 | Mkoani | 20/2/2013 | MKANYAGENI | 17034 | F | 10 | 3 |
| Pemba | 2013 | Mkoani | 20/2/2013 | MKANYAGENI | 17035 | M | 12 | 3 |
| Pemba | 2013 | Mkoani | 20/2/2013 | MKANYAGENI | 17036 | M | 11 | 3 |
| Pemba | 2013 | Mkoani | 20/2/2013 | MKANYAGENI | 17037 | M | 11 | 3 |
| Pemba | 2013 | Mkoani | 20/2/2013 | MKANYAGENI | 17038 | M | 11 | 3 |
| Pemba | 2013 | Mkoani | 20/2/2013 | MKANYAGENI | 17039 | M | 11 | 3 |
| Pemba | 2013 | Mkoani | 20/2/2013 | MKANYAGENI | 17040 | M | 10 | 3 |
| Pemba | 2013 | Mkoani | 20/2/2013 | MKANYAGENI | 17041 | M | 10 | 3 |
| Pemba | 2013 | Mkoani | 20/2/2013 | MKANYAGENI | 17042 | M | 11 | 3 |
| Pemba | 2013 | Mkoani | 20/2/2013 | MKANYAGENI | 17043 | M | 11 | 3 |
| Pemba | 2013 | Mkoani | 20/2/2013 | MKANYAGENI | 17044 | M | 10 | 3 |
| Pemba | 2013 | Mkoani | 20/2/2013 | MKANYAGENI | 17045 | M | 11 | 3 |
| Pemba | 2013 | Mkoani | 20/2/2013 | MKANYAGENI | 17046 | M | 10 | 3 |
| Pemba | 2013 | Mkoani | 20/2/2013 | MKANYAGENI | 17047 | M | 11 | 3 |
| Pemba | 2013 | Mkoani | 20/2/2013 | MKANYAGENI | 17048 | M | 10 | 3 |
| Pemba | 2013 | Mkoani | 20/2/2013 | MKANYAGENI | 17049 | M | 11 | 3 |
| Pemba | 2013 | Mkoani | 20/2/2013 | MKANYAGENI | 17050 | M | 10 | 3 |
| Pemba | 2013 | Mkoani | 20/2/2013 | MKANYAGENI | 17051 | M | 10 | 3 |
| Pemba | 2013 | Mkoani | 20/2/2013 | MKANYAGENI | 17052 | M | 10 | 3 |

| | | | | | | | | |
|---|---|---|---|---|---|---|---|---|
| Pemba | 2013 | Mkoani | 20/2/2013 | MKANYAGENI | 17053 | M | 11 | 3 |
| Pemba | 2013 | Mkoani | 20/2/2013 | MKANYAGENI | 17054 | M | 10 | 3 |
| Pemba | 2013 | Mkoani | 20/2/2013 | MKANYAGENI | 17055 | M | 10 | 3 |
| Pemba | 2013 | Mkoani | 20/2/2013 | MKANYAGENI | 17056 | M | 10 | 3 |
| Pemba | 2013 | Mkoani | 20/2/2013 | MKANYAGENI | 17057 | M | 11 | 3 |
| Pemba | 2013 | Mkoani | 20/2/2013 | MKANYAGENI | 17058 | M | 11 | 3 |
| Pemba | 2013 | Mkoani | 20/2/2013 | MKANYAGENI | 17059 | M | 10 | 3 |
| Pemba | 2013 | Mkoani | 20/2/2013 | MKANYAGENI | 17060 | M | 11 | 3 |
| Pemba | 2013 | Mkoani | 20/2/2013 | MKANYAGENI | 17061 | M | 10 | 3 |
| Pemba | 2013 | Mkoani | 20/2/2013 | MKANYAGENI | 17062 | M | 10 | 3 |
| Pemba | 2013 | Mkoani | 20/2/2013 | MKANYAGENI | 17063 | M | 11 | 3 |
| Pemba | 2013 | Mkoani | 20/2/2013 | MKANYAGENI | 17064 | M | 11 | 3 |
| Pemba | 2013 | Mkoani | 20/2/2013 | MKANYAGENI | 17065 | M | 11 | 3 |
| Pemba | 2013 | Mkoani | 20/2/2013 | MKANYAGENI | 17066 | M | 11 | 3 |
| Pemba | 2013 | Mkoani | 20/2/2013 | MKANYAGENI | 17067 | M | 10 | 3 |
| Pemba | 2013 | Mkoani | 20/2/2013 | MKANYAGENI | 17068 | M | 10 | 3 |
| Pemba | 2013 | Mkoani | 20/2/2013 | MKANYAGENI | 17069 | M | 10 | 3 |
| Pemba | 2013 | Mkoani | 20/2/2013 | MKANYAGENI | 17070 | M | 11 | 3 |
| Pemba | 2013 | Mkoani | 20/2/2013 | MKANYAGENI | 17071 | F | 10 | 4 |
| Pemba | 2013 | Mkoani | 20/2/2013 | MKANYAGENI | 17072 | F | 12 | 4 |
| Pemba | 2013 | Mkoani | 20/2/2013 | MKANYAGENI | 17073 | F | 12 | 4 |
| Pemba | 2013 | Mkoani | 20/2/2013 | MKANYAGENI | 17074 | F | 12 | 4 |
| Pemba | 2013 | Mkoani | 20/2/2013 | MKANYAGENI | 17075 | F | 12 | 4 |
| Pemba | 2013 | Mkoani | 20/2/2013 | MKANYAGENI | 17076 | F | 11 | 4 |
| Pemba | 2013 | Mkoani | 20/2/2013 | MKANYAGENI | 17077 | F | 10 | 4 |
| Pemba | 2013 | Mkoani | 20/2/2013 | MKANYAGENI | 17078 | F | 12 | 4 |
| Pemba | 2013 | Mkoani | 20/2/2013 | MKANYAGENI | 17079 | F | 12 | 4 |
| Pemba | 2013 | Mkoani | 20/2/2013 | MKANYAGENI | 17080 | F | 11 | 4 |
| Pemba | 2013 | Mkoani | 20/2/2013 | MKANYAGENI | 17081 | F | 10 | 4 |
| Pemba | 2013 | Mkoani | 20/2/2013 | MKANYAGENI | 17082 | F | 12 | 4 |
| Pemba | 2013 | Mkoani | 20/2/2013 | MKANYAGENI | 17083 | F | 11 | 4 |
| Pemba | 2013 | Mkoani | 20/2/2013 | MKANYAGENI | 17084 | F | 10 | 4 |
| Pemba | 2013 | Mkoani | 20/2/2013 | MKANYAGENI | 17085 | F | 10 | 4 |
| Pemba | 2013 | Mkoani | 20/2/2013 | MKANYAGENI | 17086 | F | 12 | 4 |
| Pemba | 2013 | Mkoani | 20/2/2013 | MKANYAGENI | 17087 | F | 10 | 4 |
| Pemba | 2013 | Mkoani | 20/2/2013 | MKANYAGENI | 17088 | F | 12 | 4 |
| Pemba | 2013 | Mkoani | 20/2/2013 | MKANYAGENI | 17089 | F | 11 | 4 |
| Pemba | 2013 | Mkoani | 20/2/2013 | MKANYAGENI | 17090 | F | 10 | 4 |
| Pemba | 2013 | Mkoani | 20/2/2013 | MKANYAGENI | 17091 | F | 11 | 4 |
| Pemba | 2013 | Mkoani | 20/2/2013 | MKANYAGENI | 17092 | F | 10 | 4 |
| Pemba | 2013 | Mkoani | 20/2/2013 | MKANYAGENI | 17093 | F | 11 | 4 |
| Pemba | 2013 | Mkoani | 20/2/2013 | MKANYAGENI | 17094 | F | 10 | 4 |
| Pemba | 2013 | Mkoani | 20/2/2013 | MKANYAGENI | 17095 | F | 11 | 4 |
| Pemba | 2013 | Mkoani | 20/2/2013 | MKANYAGENI | 17096 | F | 11 | 4 |
| Pemba | 2013 | Mkoani | 20/2/2013 | MKANYAGENI | 17097 | F | 11 | 4 |
| Pemba | 2013 | Mkoani | 20/2/2013 | MKANYAGENI | 17098 | F | 11 | 4 |
| Pemba | 2013 | Mkoani | 20/2/2013 | MKANYAGENI | 17099 | F | 10 | 4 |
| Pemba | 2013 | Mkoani | 20/2/2013 | MKANYAGENI | 17100 | F | 11 | 4 |
| Pemba | 2013 | Mkoani | 20/2/2013 | MKANYAGENI | 17101 | F | 11 | 4 |
| Pemba | 2013 | Mkoani | 20/2/2013 | MKANYAGENI | 17102 | F | 11 | 4 |

| | | | | | | | | |
|---|---|---|---|---|---|---|---|---|
| Pemba | 2013 | Mkoani | 20/2/2013 | MKANYAGENI | 17103 | F | 11 | 4 |
| Pemba | 2013 | Mkoani | 20/2/2013 | MKANYAGENI | 17104 | F | 11 | 4 |
| Pemba | 2013 | Mkoani | 20/2/2013 | MKANYAGENI | 17105 | F | 10 | 4 |
| Pemba | 2013 | Mkoani | 20/2/2013 | MKANYAGENI | 17106 | F | 11 | 4 |
| Pemba | 2013 | Mkoani | 20/2/2013 | MKANYAGENI | 17107 | F | 10 | 4 |
| Pemba | 2013 | Mkoani | 20/2/2013 | MKANYAGENI | 17108 | F | 12 | 4 |
| Pemba | 2013 | Mkoani | 20/2/2013 | MKANYAGENI | 17109 | F | 11 | 4 |
| Pemba | 2013 | Mkoani | 20/2/2013 | MKANYAGENI | 17110 | F | 11 | 4 |
| Pemba | 2013 | Mkoani | 20/2/2013 | MKANYAGENI | 17111 | F | 10 | 4 |
| Pemba | 2013 | Mkoani | 20/2/2013 | MKANYAGENI | 17112 | F | 11 | 4 |
| Pemba | 2013 | Mkoani | 20/2/2013 | MKANYAGENI | 17113 | M | 11 | 4 |
| Pemba | 2013 | Mkoani | 20/2/2013 | MKANYAGENI | 17114 | M | 10 | 4 |
| Pemba | 2013 | Mkoani | 20/2/2013 | MKANYAGENI | 17115 | M | 11 | 4 |
| Pemba | 2013 | Mkoani | 20/2/2013 | MKANYAGENI | 17116 | M | 11 | 4 |
| Pemba | 2013 | Mkoani | 20/2/2013 | MKANYAGENI | 17117 | M | 11 | 4 |
| Pemba | 2013 | Mkoani | 20/2/2013 | MKANYAGENI | 17118 | M | 11 | 4 |
| Pemba | 2013 | Mkoani | 20/2/2013 | MKANYAGENI | 17119 | M | 11 | 4 |
| Pemba | 2013 | Mkoani | 20/2/2013 | MKANYAGENI | 17120 | M | 11 | 4 |
| Pemba | 2013 | Mkoani | 20/2/2013 | MKANYAGENI | 17121 | M | 10 | 4 |
| Pemba | 2013 | Mkoani | 20/2/2013 | MKANYAGENI | 17122 | M | 11 | 4 |
| Pemba | 2013 | Mkoani | 20/2/2013 | MKANYAGENI | 17123 | M | 11 | 4 |
| Pemba | 2013 | Mkoani | 20/2/2013 | MKANYAGENI | 17124 | M | 11 | 4 |
| Pemba | 2013 | Mkoani | 20/2/2013 | MKANYAGENI | 17125 | M | 11 | 4 |
| Pemba | 2013 | Mkoani | 20/2/2013 | MKANYAGENI | 17126 | M | 11 | 4 |
| Pemba | 2013 | Mkoani | 20/2/2013 | MKANYAGENI | 17127 | M | 11 | 4 |
| Pemba | 2013 | Mkoani | 20/2/2013 | MKANYAGENI | 17128 | M | 11 | 4 |
| Pemba | 2013 | Mkoani | 20/2/2013 | MKANYAGENI | 17129 | M | 11 | 4 |
| Pemba | 2013 | Mkoani | 20/2/2013 | MKANYAGENI | 17130 | M | 11 | 4 |
| Pemba | 2013 | Mkoani | 21/2/2013 | MTAMBILE | 19001 | F | 10 | 4 |
| Pemba | 2013 | Mkoani | 21/2/2013 | MTAMBILE | 19002 | F | 11 | 4 |
| Pemba | 2013 | Mkoani | 21/2/2013 | MTAMBILE | 19003 | F | 11 | 4 |
| Pemba | 2013 | Mkoani | 21/2/2013 | MTAMBILE | 19004 | F | 10 | 4 |
| Pemba | 2013 | Mkoani | 21/2/2013 | MTAMBILE | 19005 | F | 10 | 4 |
| Pemba | 2013 | Mkoani | 21/2/2013 | MTAMBILE | 19006 | F | 9 | 4 |
| Pemba | 2013 | Mkoani | 21/2/2013 | MTAMBILE | 19007 | F | 10 | 4 |
| Pemba | 2013 | Mkoani | 21/2/2013 | MTAMBILE | 19008 | F | 11 | 4 |
| Pemba | 2013 | Mkoani | 21/2/2013 | MTAMBILE | 19009 | F | 11 | 4 |
| Pemba | 2013 | Mkoani | 21/2/2013 | MTAMBILE | 19010 | F | 11 | 4 |
| Pemba | 2013 | Mkoani | 21/2/2013 | MTAMBILE | 19011 | F | 10 | 4 |
| Pemba | 2013 | Mkoani | 21/2/2013 | MTAMBILE | 19012 | F | 10 | 4 |
| Pemba | 2013 | Mkoani | 21/2/2013 | MTAMBILE | 19013 | F | 10 | 4 |
| Pemba | 2013 | Mkoani | 21/2/2013 | MTAMBILE | 19014 | F | 10 | 4 |
| Pemba | 2013 | Mkoani | 21/2/2013 | MTAMBILE | 19015 | F | 12 | 4 |
| Pemba | 2013 | Mkoani | 21/2/2013 | MTAMBILE | 19016 | F | 10 | 4 |
| Pemba | 2013 | Mkoani | 21/2/2013 | MTAMBILE | 19017 | F | 11 | 4 |
| Pemba | 2013 | Mkoani | 21/2/2013 | MTAMBILE | 19018 | F | 10 | 4 |
| Pemba | 2013 | Mkoani | 21/2/2013 | MTAMBILE | 19019 | F | 12 | 4 |
| Pemba | 2013 | Mkoani | 21/2/2013 | MTAMBILE | 19020 | F | 12 | 4 |
| Pemba | 2013 | Mkoani | 21/2/2013 | MTAMBILE | 19021 | F | 11 | 4 |
| Pemba | 2013 | Mkoani | 21/2/2013 | MTAMBILE | 19022 | F | 11 | 4 |

| | | | | | | | | |
|---|---|---|---|---|---|---|---|---|
| Pemba | 2013 | Mkoani | 21/2/2013 | MTAMBILE | 19023 | F | 10 | 4 |
| Pemba | 2013 | Mkoani | 21/2/2013 | MTAMBILE | 19024 | F | 11 | 4 |
| Pemba | 2013 | Mkoani | 21/2/2013 | MTAMBILE | 19025 | F | 10 | 4 |
| Pemba | 2013 | Mkoani | 21/2/2013 | MTAMBILE | 19026 | F | 10 | 4 |
| Pemba | 2013 | Mkoani | 21/2/2013 | MTAMBILE | 19027 | F | 12 | 4 |
| Pemba | 2013 | Mkoani | 21/2/2013 | MTAMBILE | 19028 | F | 10 | 4 |
| Pemba | 2013 | Mkoani | 21/2/2013 | MTAMBILE | 19029 | F | 10 | 4 |
| Pemba | 2013 | Mkoani | 21/2/2013 | MTAMBILE | 19030 | F | 10 | 4 |
| Pemba | 2013 | Mkoani | 21/2/2013 | MTAMBILE | 19031 | F | 9 | 4 |
| Pemba | 2013 | Mkoani | 21/2/2013 | MTAMBILE | 19032 | F | 10 | 4 |
| Pemba | 2013 | Mkoani | 21/2/2013 | MTAMBILE | 19033 | F | 10 | 4 |
| Pemba | 2013 | Mkoani | 21/2/2013 | MTAMBILE | 19034 | F | 12 | 4 |
| Pemba | 2013 | Mkoani | 21/2/2013 | MTAMBILE | 19035 | F | 12 | 4 |
| Pemba | 2013 | Mkoani | 21/2/2013 | MTAMBILE | 19036 | F | 11 | 4 |
| Pemba | 2013 | Mkoani | 21/2/2013 | MTAMBILE | 19037 | F | 10 | 4 |
| Pemba | 2013 | Mkoani | 21/2/2013 | MTAMBILE | 19038 | F | 10 | 4 |
| Pemba | 2013 | Mkoani | 21/2/2013 | MTAMBILE | 19039 | F | 10 | 4 |
| Pemba | 2013 | Mkoani | 21/2/2013 | MTAMBILE | 19040 | F | 11 | 4 |
| Pemba | 2013 | Mkoani | 21/2/2013 | MTAMBILE | 19041 | F | 10 | 4 |
| Pemba | 2013 | Mkoani | 21/2/2013 | MTAMBILE | 19042 | F | 10 | 4 |
| Pemba | 2013 | Mkoani | 21/2/2013 | MTAMBILE | 19043 | F | 11 | 4 |
| Pemba | 2013 | Mkoani | 21/2/2013 | MTAMBILE | 19044 | F | 11 | 4 |
| Pemba | 2013 | Mkoani | 21/2/2013 | MTAMBILE | 19045 | F | 11 | 4 |
| Pemba | 2013 | Mkoani | 21/2/2013 | MTAMBILE | 19046 | F | 11 | 4 |
| Pemba | 2013 | Mkoani | 21/2/2013 | MTAMBILE | 19047 | F | 10 | 4 |
| Pemba | 2013 | Mkoani | 21/2/2013 | MTAMBILE | 19048 | F | 11 | 4 |
| Pemba | 2013 | Mkoani | 21/2/2013 | MTAMBILE | 19049 | F | 10 | 4 |
| Pemba | 2013 | Mkoani | 21/2/2013 | MTAMBILE | 19050 | F | 10 | 4 |
| Pemba | 2013 | Mkoani | 21/2/2013 | MTAMBILE | 19051 | F | 12 | 4 |
| Pemba | 2013 | Mkoani | 21/2/2013 | MTAMBILE | 19052 | F | 10 | 4 |
| Pemba | 2013 | Mkoani | 21/2/2013 | MTAMBILE | 19053 | F | 11 | 4 |
| Pemba | 2013 | Mkoani | 21/2/2013 | MTAMBILE | 19054 | F | 11 | 4 |
| Pemba | 2013 | Mkoani | 21/2/2013 | MTAMBILE | 19055 | F | 10 | 4 |
| Pemba | 2013 | Mkoani | 21/2/2013 | MTAMBILE | 19056 | F | 12 | 4 |
| Pemba | 2013 | Mkoani | 21/2/2013 | MTAMBILE | 19057 | F | 12 | 4 |
| Pemba | 2013 | Mkoani | 21/2/2013 | MTAMBILE | 19058 | F | 11 | 4 |
| Pemba | 2013 | Mkoani | 21/2/2013 | MTAMBILE | 19059 | F | 11 | 4 |
| Pemba | 2013 | Mkoani | 21/2/2013 | MTAMBILE | 19060 | F | 11 | 4 |
| Pemba | 2013 | Mkoani | 21/2/2013 | MTAMBILE | 19061 | F | 12 | 4 |
| Pemba | 2013 | Mkoani | 21/2/2013 | MTAMBILE | 19062 | F | 12 | 4 |
| Pemba | 2013 | Mkoani | 21/2/2013 | MTAMBILE | 19063 | F | 10 | 4 |
| Pemba | 2013 | Mkoani | 21/2/2013 | MTAMBILE | 19064 | M | 10 | 4 |
| Pemba | 2013 | Mkoani | 21/2/2013 | MTAMBILE | 19065 | M | 10 | 4 |
| Pemba | 2013 | Mkoani | 21/2/2013 | MTAMBILE | 19066 | M | 11 | 4 |
| Pemba | 2013 | Mkoani | 21/2/2013 | MTAMBILE | 19067 | M | 10 | 4 |
| Pemba | 2013 | Mkoani | 21/2/2013 | MTAMBILE | 19068 | M | 12 | 4 |
| Pemba | 2013 | Mkoani | 21/2/2013 | MTAMBILE | 19069 | M | 10 | 4 |
| Pemba | 2013 | Mkoani | 21/2/2013 | MTAMBILE | 19070 | M | 12 | 4 |
| Pemba | 2013 | Mkoani | 21/2/2013 | MTAMBILE | 19071 | M | 12 | 4 |
| Pemba | 2013 | Mkoani | 21/2/2013 | MTAMBILE | 19072 | M | 11 | 4 |

| | | | | | | | | |
|---|---|---|---|---|---|---|---|---|
| Pemba | 2013 | Mkoani | 21/2/2013 | MTAMBILE | 19073 | M | 10 | 4 |
| Pemba | 2013 | Mkoani | 21/2/2013 | MTAMBILE | 19074 | M | 11 | 4 |
| Pemba | 2013 | Mkoani | 21/2/2013 | MTAMBILE | 19075 | M | 11 | 4 |
| Pemba | 2013 | Mkoani | 21/2/2013 | MTAMBILE | 19076 | M | 11 | 4 |
| Pemba | 2013 | Mkoani | 21/2/2013 | MTAMBILE | 19077 | M | 11 | 4 |
| Pemba | 2013 | Mkoani | 21/2/2013 | MTAMBILE | 19078 | M | 11 | 4 |
| Pemba | 2013 | Mkoani | 21/2/2013 | MTAMBILE | 19079 | M | 10 | 4 |
| Pemba | 2013 | Mkoani | 21/2/2013 | MTAMBILE | 19080 | M | 10 | 4 |
| Pemba | 2013 | Mkoani | 21/2/2013 | MTAMBILE | 19081 | M | 10 | 4 |
| Pemba | 2013 | Mkoani | 21/2/2013 | MTAMBILE | 19082 | M | 11 | 4 |
| Pemba | 2013 | Mkoani | 21/2/2013 | MTAMBILE | 19083 | M | 11 | 4 |
| Pemba | 2013 | Mkoani | 21/2/2013 | MTAMBILE | 19084 | M | 12 | 4 |
| Pemba | 2013 | Mkoani | 21/2/2013 | MTAMBILE | 19085 | M | 11 | 4 |
| Pemba | 2013 | Mkoani | 21/2/2013 | MTAMBILE | 19086 | M | 10 | 4 |
| Pemba | 2013 | Mkoani | 21/2/2013 | MTAMBILE | 19087 | M | 11 | 4 |
| Pemba | 2013 | Mkoani | 21/2/2013 | MTAMBILE | 19088 | M | 10 | 4 |
| Pemba | 2013 | Mkoani | 21/2/2013 | MTAMBILE | 19089 | M | 11 | 4 |
| Pemba | 2013 | Mkoani | 21/2/2013 | MTAMBILE | 19090 | M | 10 | 4 |
| Pemba | 2013 | Mkoani | 21/2/2013 | MTAMBILE | 19091 | M | 10 | 4 |
| Pemba | 2013 | Mkoani | 21/2/2013 | MTAMBILE | 19092 | M | 11 | 4 |
| Pemba | 2013 | Mkoani | 21/2/2013 | MTAMBILE | 19093 | M | 11 | 4 |
| Pemba | 2013 | Mkoani | 21/2/2013 | MTAMBILE | 19094 | M | 11 | 4 |
| Pemba | 2013 | Mkoani | 21/2/2013 | MTAMBILE | 19095 | M | 12 | 4 |
| Pemba | 2013 | Mkoani | 21/2/2013 | MTAMBILE | 19096 | M | 10 | 4 |
| Pemba | 2013 | Mkoani | 21/2/2013 | MTAMBILE | 19097 | M | 11 | 4 |
| Pemba | 2013 | Mkoani | 21/2/2013 | MTAMBILE | 19098 | M | 10 | 4 |
| Pemba | 2013 | Mkoani | 21/2/2013 | MTAMBILE | 19099 | M | 10 | 4 |
| Pemba | 2013 | Mkoani | 21/2/2013 | MTAMBILE | 19100 | M | 10 | 4 |
| Pemba | 2013 | Mkoani | 21/2/2013 | MTAMBILE | 19101 | M | 12 | 4 |
| Pemba | 2013 | Mkoani | 21/2/2013 | MTAMBILE | 19102 | M | 11 | 4 |
| Pemba | 2013 | Mkoani | 21/2/2013 | MTAMBILE | 19103 | M | 11 | 4 |
| Pemba | 2013 | Mkoani | 21/2/2013 | MTAMBILE | 19104 | M | 10 | 4 |
| Pemba | 2013 | Mkoani | 21/2/2013 | MTAMBILE | 19105 | M | 10 | 4 |
| Pemba | 2013 | Mkoani | 21/2/2013 | MTAMBILE | 19106 | M | 10 | 4 |
| Pemba | 2013 | Mkoani | 21/2/2013 | MTAMBILE | 19107 | M | 10 | 4 |
| Pemba | 2013 | Mkoani | 21/2/2013 | MTAMBILE | 19108 | F | 10 | 4 |
| Pemba | 2013 | Mkoani | 21/2/2013 | MTAMBILE | 19109 | F | 10 | 4 |
| Pemba | 2013 | Mkoani | 21/2/2013 | MTAMBILE | 19110 | F | 10 | 3 |
| Pemba | 2013 | Mkoani | 21/2/2013 | MTAMBILE | 19111 | F | 11 | 3 |
| Pemba | 2013 | Mkoani | 21/2/2013 | MTAMBILE | 19112 | M | 11 | 3 |
| Pemba | 2013 | Mkoani | 21/2/2013 | MTAMBILE | 19113 | M | 9 | 3 |
| Pemba | 2013 | Mkoani | 21/2/2013 | MTAMBILE | 19114 | M | 12 | 3 |
| Pemba | 2013 | Mkoani | 21/2/2013 | MTAMBILE | 19115 | M | 10 | 3 |
| Pemba | 2013 | Mkoani | 21/2/2013 | MTAMBILE | 19116 | M | 10 | 3 |
| Pemba | 2013 | Mkoani | 21/2/2013 | MTAMBILE | 19117 | M | 10 | 3 |
| Pemba | 2013 | Mkoani | 21/2/2013 | MTAMBILE | 19118 | M | 10 | 3 |
| Pemba | 2013 | Mkoani | 21/2/2013 | MTAMBILE | 19119 | F | 9 | 3 |
| Pemba | 2013 | Mkoani | 21/2/2013 | MTAMBILE | 19120 | F | 9 | 3 |
| Pemba | 2013 | Mkoani | 21/2/2013 | MTAMBILE | 19121 | F | 11 | 3 |
| Pemba | 2013 | Mkoani | 21/2/2013 | MTAMBILE | 19122 | F | 10 | 3 |

| | | | | | | | | |
|---|---|---|---|---|---|---|---|---|
| Pemba | 2013 | Mkoani | 21/2/2013 | MTAMBILE | 19123 | F | 10 | 3 |
| Pemba | 2013 | Mkoani | 21/2/2013 | MTAMBILE | 19124 | F | 10 | 3 |
| Pemba | 2013 | Mkoani | 21/2/2013 | MTAMBILE | 19125 | F | 10 | 3 |
| Pemba | 2013 | Mkoani | 21/2/2013 | MTAMBILE | 19126 | F | 9 | 3 |
| Pemba | 2013 | Mkoani | 21/2/2013 | MTAMBILE | 19127 | M | 10 | 3 |
| Pemba | 2013 | Mkoani | 21/2/2013 | MTAMBILE | 19128 | M | 10 | 3 |
| Pemba | 2013 | Mkoani | 21/2/2013 | MTAMBILE | 19129 | M | 10 | 3 |
| Pemba | 2013 | Mkoani | 21/2/2013 | MTAMBILE | 19130 | M | 9 | 3 |
| Pemba | 2013 | Mkoani | 05.02.2013 | KANGANI | 20001 | F | 11 | 4 |
| Pemba | 2013 | Mkoani | 05.02.2013 | KANGANI | 20002 | F | 10 | 4 |
| Pemba | 2013 | Mkoani | 05.02.2013 | KANGANI | 20003 | F | 11 | 4 |
| Pemba | 2013 | Mkoani | 05.02.2013 | KANGANI | 20004 | F | 11 | 4 |
| Pemba | 2013 | Mkoani | 05.02.2013 | KANGANI | 20005 | F | 11 | 4 |
| Pemba | 2013 | Mkoani | 05.02.2013 | KANGANI | 20006 | F | 10 | 4 |
| Pemba | 2013 | Mkoani | 05.02.2013 | KANGANI | 20007 | F | 11 | 4 |
| Pemba | 2013 | Mkoani | 05.02.2013 | KANGANI | 20008 | F | 10 | 4 |
| Pemba | 2013 | Mkoani | 05.02.2013 | KANGANI | 20009 | F | 11 | 4 |
| Pemba | 2013 | Mkoani | 05.02.2013 | KANGANI | 20010 | F | 12 | 4 |
| Pemba | 2013 | Mkoani | 05.02.2013 | KANGANI | 20011 | F | 10 | 4 |
| Pemba | 2013 | Mkoani | 05.02.2013 | KANGANI | 20012 | F | 12 | 4 |
| Pemba | 2013 | Mkoani | 05.02.2013 | KANGANI | 20013 | F | 11 | 4 |
| Pemba | 2013 | Mkoani | 05.02.2013 | KANGANI | 20014 | F | 10 | 4 |
| Pemba | 2013 | Mkoani | 05.02.2013 | KANGANI | 20015 | F | 10 | 4 |
| Pemba | 2013 | Mkoani | 05.02.2013 | KANGANI | 20016 | F | 10 | 4 |
| Pemba | 2013 | Mkoani | 05.02.2013 | KANGANI | 20017 | F | 11 | 4 |
| Pemba | 2013 | Mkoani | 05.02.2013 | KANGANI | 20018 | F | 11 | 4 |
| Pemba | 2013 | Mkoani | 05.02.2013 | KANGANI | 20019 | F | 10 | 4 |
| Pemba | 2013 | Mkoani | 05.02.2013 | KANGANI | 20020 | F | 11 | 4 |
| Pemba | 2013 | Mkoani | 05.02.2013 | KANGANI | 20021 | F | 10 | 4 |
| Pemba | 2013 | Mkoani | 05.02.2013 | KANGANI | 20022 | F | 11 | 4 |
| Pemba | 2013 | Mkoani | 05.02.2013 | KANGANI | 20023 | F | 11 | 4 |
| Pemba | 2013 | Mkoani | 05.02.2013 | KANGANI | 20024 | F | 11 | 4 |
| Pemba | 2013 | Mkoani | 05.02.2013 | KANGANI | 20025 | F | 11 | 4 |
| Pemba | 2013 | Mkoani | 05.02.2013 | KANGANI | 20026 | F | 11 | 4 |
| Pemba | 2013 | Mkoani | 05.02.2013 | KANGANI | 20027 | F | 11 | 4 |
| Pemba | 2013 | Mkoani | 05.02.2013 | KANGANI | 20028 | F | 11 | 4 |
| Pemba | 2013 | Mkoani | 05.02.2013 | KANGANI | 20029 | F | 11 | 4 |
| Pemba | 2013 | Mkoani | 05.02.2013 | KANGANI | 20030 | F | 12 | 4 |
| Pemba | 2013 | Mkoani | 05.02.2013 | KANGANI | 20031 | F | 12 | 4 |
| Pemba | 2013 | Mkoani | 05.02.2013 | KANGANI | 20032 | F | 10 | 4 |
| Pemba | 2013 | Mkoani | 05.02.2013 | KANGANI | 20033 | F | 10 | 4 |
| Pemba | 2013 | Mkoani | 05.02.2013 | KANGANI | 20034 | F | 11 | 4 |
| Pemba | 2013 | Mkoani | 05.02.2013 | KANGANI | 20035 | F | 11 | 4 |
| Pemba | 2013 | Mkoani | 05.02.2013 | KANGANI | 20036 | F | 10 | 4 |
| Pemba | 2013 | Mkoani | 05.02.2013 | KANGANI | 20037 | F | 11 | 4 |
| Pemba | 2013 | Mkoani | 05.02.2013 | KANGANI | 20038 | F | 12 | 4 |
| Pemba | 2013 | Mkoani | 05.02.2013 | KANGANI | 20039 | F | 11 | 4 |
| Pemba | 2013 | Mkoani | 05.02.2013 | KANGANI | 20040 | F | 11 | 4 |
| Pemba | 2013 | Mkoani | 05.02.2013 | KANGANI | 20041 | F | 10 | 4 |
| Pemba | 2013 | Mkoani | 05.02.2013 | KANGANI | 20042 | F | 12 | 4 |

| | | | | | | | | |
|---|---|---|---|---|---|---|---|---|
| Pemba | 2013 | Mkoani | 05.02.2013 | KANGANI | 20043 | M | 10 | 4 |
| Pemba | 2013 | Mkoani | 05.02.2013 | KANGANI | 20044 | M | 10 | 4 |
| Pemba | 2013 | Mkoani | 05.02.2013 | KANGANI | 20045 | M | 10 | 4 |
| Pemba | 2013 | Mkoani | 05.02.2013 | KANGANI | 20046 | M | 11 | 4 |
| Pemba | 2013 | Mkoani | 05.02.2013 | KANGANI | 20047 | M | 11 | 4 |
| Pemba | 2013 | Mkoani | 05.02.2013 | KANGANI | 20048 | M | 12 | 4 |
| Pemba | 2013 | Mkoani | 05.02.2013 | KANGANI | 20049 | M | 11 | 4 |
| Pemba | 2013 | Mkoani | 05.02.2013 | KANGANI | 20050 | M | 11 | 4 |
| Pemba | 2013 | Mkoani | 05.02.2013 | KANGANI | 20051 | M | 10 | 4 |
| Pemba | 2013 | Mkoani | 05.02.2013 | KANGANI | 20052 | M | 10 | 4 |
| Pemba | 2013 | Mkoani | 05.02.2013 | KANGANI | 20053 | M | 10 | 4 |
| Pemba | 2013 | Mkoani | 05.02.2013 | KANGANI | 20054 | M | 11 | 4 |
| Pemba | 2013 | Mkoani | 05.02.2013 | KANGANI | 20055 | M | 10 | 4 |
| Pemba | 2013 | Mkoani | 05.02.2013 | KANGANI | 20056 | M | 10 | 4 |
| Pemba | 2013 | Mkoani | 05.02.2013 | KANGANI | 20057 | M | 11 | 4 |
| Pemba | 2013 | Mkoani | 05.02.2013 | KANGANI | 20058 | M | 11 | 4 |
| Pemba | 2013 | Mkoani | 05.02.2013 | KANGANI | 20059 | M | 11 | 4 |
| Pemba | 2013 | Mkoani | 05.02.2013 | KANGANI | 20060 | M | 10 | 4 |
| Pemba | 2013 | Mkoani | 05.02.2013 | KANGANI | 20061 | M | 11 | 4 |
| Pemba | 2013 | Mkoani | 05.02.2013 | KANGANI | 20062 | M | 11 | 4 |
| Pemba | 2013 | Mkoani | 05.02.2013 | KANGANI | 20063 | M | 10 | 4 |
| Pemba | 2013 | Mkoani | 05.02.2013 | KANGANI | 20064 | M | 12 | 4 |
| Pemba | 2013 | Mkoani | 05.02.2013 | KANGANI | 20065 | M | 10 | 4 |
| Pemba | 2013 | Mkoani | 05.02.2013 | KANGANI | 20066 | M | 12 | 4 |
| Pemba | 2013 | Mkoani | 05.02.2013 | KANGANI | 20067 | M | 12 | 4 |
| Pemba | 2013 | Mkoani | 05.02.2013 | KANGANI | 20068 | M | 12 | 4 |
| Pemba | 2013 | Mkoani | 05.02.2013 | KANGANI | 20069 | F | 11 | 4 |
| Pemba | 2013 | Mkoani | 05.02.2013 | KANGANI | 20070 | F | 10 | 4 |
| Pemba | 2013 | Mkoani | 05.02.2013 | KANGANI | 20071 | F | 10 | 4 |
| Pemba | 2013 | Mkoani | 05.02.2013 | KANGANI | 20072 | F | 10 | 4 |
| Pemba | 2013 | Mkoani | 05.02.2013 | KANGANI | 20073 | F | 11 | 4 |
| Pemba | 2013 | Mkoani | 05.02.2013 | KANGANI | 20074 | F | 11 | 4 |
| Pemba | 2013 | Mkoani | 05.02.2013 | KANGANI | 20075 | F | 10 | 4 |
| Pemba | 2013 | Mkoani | 05.02.2013 | KANGANI | 20076 | F | 10 | 3 |
| Pemba | 2013 | Mkoani | 05.02.2013 | KANGANI | 20077 | F | 11 | 3 |
| Pemba | 2013 | Mkoani | 05.02.2013 | KANGANI | 20078 | F | 12 | 3 |
| Pemba | 2013 | Mkoani | 05.02.2013 | KANGANI | 20079 | F | 11 | 3 |
| Pemba | 2013 | Mkoani | 05.02.2013 | KANGANI | 20080 | F | 10 | 3 |
| Pemba | 2013 | Mkoani | 05.02.2013 | KANGANI | 20081 | F | 10 | 3 |
| Pemba | 2013 | Mkoani | 05.02.2013 | KANGANI | 20082 | F | 11 | 3 |
| Pemba | 2013 | Mkoani | 05.02.2013 | KANGANI | 20083 | F | 10 | 3 |
| Pemba | 2013 | Mkoani | 05.02.2013 | KANGANI | 20084 | F | 11 | 3 |
| Pemba | 2013 | Mkoani | 05.02.2013 | KANGANI | 20085 | F | 11 | 3 |
| Pemba | 2013 | Mkoani | 05.02.2013 | KANGANI | 20086 | F | 10 | 3 |
| Pemba | 2013 | Mkoani | 05.02.2013 | KANGANI | 20087 | F | 10 | 3 |
| Pemba | 2013 | Mkoani | 05.02.2013 | KANGANI | 20088 | F | 11 | 3 |
| Pemba | 2013 | Mkoani | 05.02.2013 | KANGANI | 20089 | F | 10 | 3 |
| Pemba | 2013 | Mkoani | 05.02.2013 | KANGANI | 20090 | F | 11 | 3 |
| Pemba | 2013 | Mkoani | 05.02.2013 | KANGANI | 20091 | F | 11 | 3 |
| Pemba | 2013 | Mkoani | 05.02.2013 | KANGANI | 20092 | F | 10 | 3 |

| | | | | | | | | |
|---|---|---|---|---|---|---|---|---|
| Pemba | 2013 | Mkoani | 05.02.2013 | KANGANI | 20093 | F | 10 | 3 |
| Pemba | 2013 | Mkoani | 05.02.2013 | KANGANI | 20094 | F | 11 | 3 |
| Pemba | 2013 | Mkoani | 05.02.2013 | KANGANI | 20095 | F | 10 | 3 |
| Pemba | 2013 | Mkoani | 05.02.2013 | KANGANI | 20096 | F | 10 | 3 |
| Pemba | 2013 | Mkoani | 05.02.2013 | KANGANI | 20097 | F | 11 | 3 |
| Pemba | 2013 | Mkoani | 05.02.2013 | KANGANI | 20098 | F | 10 | 3 |
| Pemba | 2013 | Mkoani | 05.02.2013 | KANGANI | 20099 | F | 10 | 3 |
| Pemba | 2013 | Mkoani | 05.02.2013 | KANGANI | 20100 | F | 11 | 3 |
| Pemba | 2013 | Mkoani | 05.02.2013 | KANGANI | 20101 | M | 10 | 3 |
| Pemba | 2013 | Mkoani | 05.02.2013 | KANGANI | 20102 | M | 10 | 3 |
| Pemba | 2013 | Mkoani | 05.02.2013 | KANGANI | 20103 | M | 11 | 3 |
| Pemba | 2013 | Mkoani | 05.02.2013 | KANGANI | 20104 | M | 10 | 3 |
| Pemba | 2013 | Mkoani | 05.02.2013 | KANGANI | 20105 | M | 10 | 3 |
| Pemba | 2013 | Mkoani | 05.02.2013 | KANGANI | 20106 | M | 10 | 3 |
| Pemba | 2013 | Mkoani | 05.02.2013 | KANGANI | 20107 | M | 11 | 3 |
| Pemba | 2013 | Mkoani | 05.02.2013 | KANGANI | 20108 | M | 11 | 3 |
| Pemba | 2013 | Mkoani | 05.02.2013 | KANGANI | 20109 | M | 10 | 3 |
| Pemba | 2013 | Mkoani | 05.02.2013 | KANGANI | 20110 | M | 11 | 3 |
| Pemba | 2013 | Mkoani | 05.02.2013 | KANGANI | 20111 | M | 10 | 3 |
| Pemba | 2013 | Mkoani | 05.02.2013 | KANGANI | 20112 | M | 11 | 3 |
| Pemba | 2013 | Mkoani | 05.02.2013 | KANGANI | 20113 | M | 10 | 3 |
| Pemba | 2013 | Mkoani | 05.02.2013 | KANGANI | 20114 | M | 10 | 3 |
| Pemba | 2013 | Mkoani | 05.02.2013 | KANGANI | 20115 | M | 10 | 3 |
| Pemba | 2013 | Mkoani | 05.02.2013 | KANGANI | 20116 | M | 11 | 3 |
| Pemba | 2013 | Mkoani | 05.02.2013 | KANGANI | 20117 | M | 11 | 3 |
| Pemba | 2013 | Mkoani | 05.02.2013 | KANGANI | 20118 | M | 11 | 3 |
| Pemba | 2013 | Mkoani | 05.02.2013 | KANGANI | 20119 | M | 11 | 3 |
| Pemba | 2013 | Mkoani | 05.02.2013 | KANGANI | 20120 | M | 10 | 3 |
| Pemba | 2013 | Mkoani | 05.02.2013 | KANGANI | 20121 | M | 11 | 3 |
| Pemba | 2013 | Mkoani | 05.02.2013 | KANGANI | 20122 | M | 10 | 3 |
| Pemba | 2013 | Mkoani | 05.02.2013 | KANGANI | 20123 | M | 10 | 3 |
| Pemba | 2013 | Mkoani | 05.02.2013 | KANGANI | 20124 | M | 10 | 3 |
| Pemba | 2013 | Mkoani | 05.02.2013 | KANGANI | 20125 | M | 10 | 3 |
| Pemba | 2013 | Mkoani | 05.02.2013 | KANGANI | 20126 | M | 10 | 3 |
| Pemba | 2013 | Mkoani | 05.02.2013 | KANGANI | 20127 | M | 11 | 3 |
| Pemba | 2013 | Mkoani | 05.02.2013 | KANGANI | 20128 | M | 10 | 3 |
| Pemba | 2013 | Mkoani | 05.02.2013 | KANGANI | 20129 | M | 11 | 3 |
| Pemba | 2013 | Mkoani | 05.02.2013 | KANGANI | 20130 | M | 10 | 3 |
| Pemba | 2013 | Mkoani | 30/4/2013 | KENGEJA | 21001 | F | 12 | 4 |
| Pemba | 2013 | Mkoani | 30/4/2013 | KENGEJA | 21002 | F | 10 | 4 |
| Pemba | 2013 | Mkoani | 30/4/2013 | KENGEJA | 21003 | F | 10 | 4 |
| Pemba | 2013 | Mkoani | 30/4/2013 | KENGEJA | 21004 | F | 11 | 4 |
| Pemba | 2013 | Mkoani | 30/4/2013 | KENGEJA | 21005 | F | 10 | 4 |
| Pemba | 2013 | Mkoani | 30/4/2013 | KENGEJA | 21006 | F | 12 | 4 |
| Pemba | 2013 | Mkoani | 30/4/2013 | KENGEJA | 21007 | F | 11 | 4 |
| Pemba | 2013 | Mkoani | 30/4/2013 | KENGEJA | 21008 | F | 10 | 4 |
| Pemba | 2013 | Mkoani | 30/4/2013 | KENGEJA | 21009 | F | 11 | 4 |
| Pemba | 2013 | Mkoani | 30/4/2013 | KENGEJA | 21010 | F | 11 | 4 |
| Pemba | 2013 | Mkoani | 30/4/2013 | KENGEJA | 21011 | F | 10 | 4 |
| Pemba | 2013 | Mkoani | 30/4/2013 | KENGEJA | 21012 | F | 10 | 4 |

| | | | | | | | | |
|---|---|---|---|---|---|---|---|---|
| Pemba | 2013 | Mkoani | 30/4/2013 | KENGEJA | 21013 | F | 11 | 4 |
| Pemba | 2013 | Mkoani | 30/4/2013 | KENGEJA | 21014 | F | 11 | 4 |
| Pemba | 2013 | Mkoani | 30/4/2013 | KENGEJA | 21015 | F | 12 | 4 |
| Pemba | 2013 | Mkoani | 30/4/2013 | KENGEJA | 21016 | F | 11 | 4 |
| Pemba | 2013 | Mkoani | 30/4/2013 | KENGEJA | 21017 | F | 11 | 4 |
| Pemba | 2013 | Mkoani | 30/4/2013 | KENGEJA | 21018 | F | 10 | 4 |
| Pemba | 2013 | Mkoani | 30/4/2013 | KENGEJA | 21019 | F | 11 | 4 |
| Pemba | 2013 | Mkoani | 30/4/2013 | KENGEJA | 21020 | F | 12 | 4 |
| Pemba | 2013 | Mkoani | 30/4/2013 | KENGEJA | 21021 | F | 10 | 4 |
| Pemba | 2013 | Mkoani | 30/4/2013 | KENGEJA | 21022 | F | 11 | 4 |
| Pemba | 2013 | Mkoani | 30/4/2013 | KENGEJA | 21023 | F | 10 | 4 |
| Pemba | 2013 | Mkoani | 30/4/2013 | KENGEJA | 21024 | F | 10 | 4 |
| Pemba | 2013 | Mkoani | 30/4/2013 | KENGEJA | 21025 | M | 11 | 4 |
| Pemba | 2013 | Mkoani | 30/4/2013 | KENGEJA | 21026 | M | 12 | 4 |
| Pemba | 2013 | Mkoani | 30/4/2013 | KENGEJA | 21027 | M | 12 | 4 |
| Pemba | 2013 | Mkoani | 30/4/2013 | KENGEJA | 21028 | M | 11 | 4 |
| Pemba | 2013 | Mkoani | 30/4/2013 | KENGEJA | 21029 | M | 10 | 4 |
| Pemba | 2013 | Mkoani | 30/4/2013 | KENGEJA | 21030 | M | 11 | 4 |
| Pemba | 2013 | Mkoani | 30/4/2013 | KENGEJA | 21031 | M | 11 | 4 |
| Pemba | 2013 | Mkoani | 30/4/2013 | KENGEJA | 21032 | M | 11 | 4 |
| Pemba | 2013 | Mkoani | 30/4/2013 | KENGEJA | 21033 | M | 10 | 4 |
| Pemba | 2013 | Mkoani | 30/4/2013 | KENGEJA | 21034 | M | 10 | 4 |
| Pemba | 2013 | Mkoani | 30/4/2013 | KENGEJA | 21035 | M | 12 | 4 |
| Pemba | 2013 | Mkoani | 30/4/2013 | KENGEJA | 21036 | M | 11 | 4 |
| Pemba | 2013 | Mkoani | 30/4/2013 | KENGEJA | 21037 | M | 11 | 4 |
| Pemba | 2013 | Mkoani | 30/4/2013 | KENGEJA | 21038 | M | 11 | 4 |
| Pemba | 2013 | Mkoani | 30/4/2013 | KENGEJA | 21039 | M | 12 | 4 |
| Pemba | 2013 | Mkoani | 30/4/2013 | KENGEJA | 21040 | M | 10 | 4 |
| Pemba | 2013 | Mkoani | 30/4/2013 | KENGEJA | 21041 | M | 10 | 4 |
| Pemba | 2013 | Mkoani | 30/4/2013 | KENGEJA | 21042 | M | 12 | 4 |
| Pemba | 2013 | Mkoani | 30/4/2013 | KENGEJA | 21043 | M | 10 | 4 |
| Pemba | 2013 | Mkoani | 30/4/2013 | KENGEJA | 21044 | M | 10 | 4 |
| Pemba | 2013 | Mkoani | 30/4/2013 | KENGEJA | 21045 | M | 11 | 4 |
| Pemba | 2013 | Mkoani | 30/4/2013 | KENGEJA | 21046 | M | 12 | 4 |
| Pemba | 2013 | Mkoani | 30/4/2013 | KENGEJA | 21047 | M | 10 | 4 |
| Pemba | 2013 | Mkoani | 30/4/2013 | KENGEJA | 21048 | M | 10 | 4 |
| Pemba | 2013 | Mkoani | 30/4/2013 | KENGEJA | 21049 | M | 11 | 4 |
| Pemba | 2013 | Mkoani | 30/4/2013 | KENGEJA | 21050 | F | 11 | 4 |
| Pemba | 2013 | Mkoani | 30/4/2013 | KENGEJA | 21051 | F | 10 | 4 |
| Pemba | 2013 | Mkoani | 30/4/2013 | KENGEJA | 21052 | F | 11 | 4 |
| Pemba | 2013 | Mkoani | 30/4/2013 | KENGEJA | 21053 | F | 11 | 4 |
| Pemba | 2013 | Mkoani | 30/4/2013 | KENGEJA | 21054 | F | 11 | 4 |
| Pemba | 2013 | Mkoani | 30/4/2013 | KENGEJA | 21055 | M | 11 | 4 |
| Pemba | 2013 | Mkoani | 30/4/2013 | KENGEJA | 21056 | F | 10 | 4 |
| Pemba | 2013 | Mkoani | 30/4/2013 | KENGEJA | 21057 | F | 12 | 4 |
| Pemba | 2013 | Mkoani | 30/4/2013 | KENGEJA | 21058 | M | 11 | 4 |
| Pemba | 2013 | Mkoani | 30/4/2013 | KENGEJA | 21059 | M | 10 | 4 |
| Pemba | 2013 | Mkoani | 30/4/2013 | KENGEJA | 21060 | M | 10 | 4 |
| Pemba | 2013 | Mkoani | 30/4/2013 | KENGEJA | 21061 | M | 11 | 4 |
| Pemba | 2013 | Mkoani | 30/4/2013 | KENGEJA | 21062 | M | 11 | 4 |

| | | | | | | | | |
|---|---|---|---|---|---|---|---|---|
| Pemba | 2013 | Mkoani | 30/4/2013 | KENGEJA | 21063 | M | 11 | 4 |
| Pemba | 2013 | Mkoani | 30/4/2013 | KENGEJA | 21064 | M | 10 | 4 |
| Pemba | 2013 | Mkoani | 30/4/2013 | KENGEJA | 21065 | M | 10 | 4 |
| Pemba | 2013 | Mkoani | 30/4/2013 | KENGEJA | 21066 | M | 11 | 4 |
| Pemba | 2013 | Mkoani | 30/4/2013 | KENGEJA | 21067 | F | 12 | 4 |
| Pemba | 2013 | Mkoani | 30/4/2013 | KENGEJA | 21068 | F | 12 | 4 |
| Pemba | 2013 | Mkoani | 30/4/2013 | KENGEJA | 21069 | M | 11 | 4 |
| Pemba | 2013 | Mkoani | 30/4/2013 | KENGEJA | 21070 | M | 11 | 4 |
| Pemba | 2013 | Mkoani | 30/4/2013 | KENGEJA | 21071 | M | 12 | 4 |
| Pemba | 2013 | Mkoani | 30/4/2013 | KENGEJA | 21072 | M | 11 | 4 |
| Pemba | 2013 | Mkoani | 30/4/2013 | KENGEJA | 21073 | M | 12 | 4 |
| Pemba | 2013 | Mkoani | 30/4/2013 | KENGEJA | 21074 | M | 12 | 4 |
| Pemba | 2013 | Mkoani | 30/4/2013 | KENGEJA | 21075 | M | 11 | 4 |
| Pemba | 2013 | Mkoani | 30/4/2013 | KENGEJA | 21076 | M | 9 | 3 |
| Pemba | 2013 | Mkoani | 30/4/2013 | KENGEJA | 21077 | M | 9 | 3 |
| Pemba | 2013 | Mkoani | 30/4/2013 | KENGEJA | 21078 | M | 9 | 3 |
| Pemba | 2013 | Mkoani | 30/4/2013 | KENGEJA | 21079 | M | 9 | 3 |
| Pemba | 2013 | Mkoani | 30/4/2013 | KENGEJA | 21080 | M | 9 | 3 |
| Pemba | 2013 | Mkoani | 30/4/2013 | KENGEJA | 21081 | F | 9 | 3 |
| Pemba | 2013 | Mkoani | 30/4/2013 | KENGEJA | 21082 | F | 9 | 3 |
| Pemba | 2013 | Mkoani | 30/4/2013 | KENGEJA | 21083 | F | 9 | 3 |
| Pemba | 2013 | Mkoani | 30/4/2013 | KENGEJA | 21084 | F | 10 | 3 |
| Pemba | 2013 | Mkoani | 30/4/2013 | KENGEJA | 21085 | F | 9 | 3 |
| Pemba | 2013 | Mkoani | 30/4/2013 | KENGEJA | 21086 | F | 9 | 3 |
| Pemba | 2013 | Mkoani | 30/4/2013 | KENGEJA | 21087 | F | 9 | 3 |
| Pemba | 2013 | Mkoani | 30/4/2013 | KENGEJA | 21088 | F | 9 | 3 |
| Pemba | 2013 | Mkoani | 30/4/2013 | KENGEJA | 21089 | F | 10 | 3 |
| Pemba | 2013 | Mkoani | 30/4/2013 | KENGEJA | 21090 | F | 9 | 3 |
| Pemba | 2013 | Mkoani | 30/4/2013 | KENGEJA | 21091 | F | 9 | 3 |
| Pemba | 2013 | Mkoani | 30/4/2013 | KENGEJA | 21092 | F | 9 | 3 |
| Pemba | 2013 | Mkoani | 30/4/2013 | KENGEJA | 21093 | M | 9 | 3 |
| Pemba | 2013 | Mkoani | 30/4/2013 | KENGEJA | 21094 | M | 10 | 3 |
| Pemba | 2013 | Mkoani | 30/4/2013 | KENGEJA | 21095 | M | 10 | 3 |
| Pemba | 2013 | Mkoani | 30/4/2013 | KENGEJA | 21096 | M | 9 | 3 |
| Pemba | 2013 | Mkoani | 30/4/2013 | KENGEJA | 21097 | M | 11 | 3 |
| Pemba | 2013 | Mkoani | 30/4/2013 | KENGEJA | 21098 | M | 11 | 3 |
| Pemba | 2013 | Mkoani | 30/4/2013 | KENGEJA | 21099 | M | 9 | 3 |
| Pemba | 2013 | Mkoani | 30/4/2013 | KENGEJA | 21100 | M | 9 | 3 |
| Pemba | 2013 | Mkoani | 30/4/2013 | KENGEJA | 21101 | M | 9 | 3 |
| Pemba | 2013 | Mkoani | 30/4/2013 | KENGEJA | 21102 | M | 9 | 3 |
| Pemba | 2013 | Mkoani | 30/4/2013 | KENGEJA | 21103 | F | 10 | 3 |
| Pemba | 2013 | Mkoani | 30/4/2013 | KENGEJA | 21104 | M | 10 | 3 |
| Pemba | 2013 | Mkoani | 30/4/2013 | KENGEJA | 21105 | M | 9 | 3 |
| Pemba | 2013 | Mkoani | 30/4/2013 | KENGEJA | 21106 | M | 9 | 3 |
| Pemba | 2013 | Mkoani | 30/4/2013 | KENGEJA | 21107 | F | 9 | 3 |
| Pemba | 2013 | Mkoani | 30/4/2013 | KENGEJA | 21108 | F | 9 | 3 |
| Pemba | 2013 | Mkoani | 30/4/2013 | KENGEJA | 21109 | F | 9 | 3 |
| Pemba | 2013 | Mkoani | 30/4/2013 | KENGEJA | 21110 | F | 9 | 3 |
| Pemba | 2013 | Mkoani | 30/4/2013 | KENGEJA | 21111 | F | 9 | 3 |
| Pemba | 2013 | Mkoani | 30/4/2013 | KENGEJA | 21112 | F | 10 | 3 |

| | | | | | | | | |
|---|---|---|---|---|---|---|---|---|
| Pemba | 2013 | Mkoani | 30/4/2013 | KENGEJA | 21113 | F | 9 | 3 |
| Pemba | 2013 | Mkoani | 30/4/2013 | KENGEJA | 21114 | F | 9 | 3 |
| Pemba | 2013 | Mkoani | 30/4/2013 | KENGEJA | 21115 | F | 9 | 3 |
| Pemba | 2013 | Mkoani | 30/4/2013 | KENGEJA | 21116 | F | 9 | 3 |
| Pemba | 2013 | Mkoani | 30/4/2013 | KENGEJA | 21117 | F | 10 | 3 |
| Pemba | 2013 | Mkoani | 30/4/2013 | KENGEJA | 21118 | F | 11 | 3 |
| Pemba | 2013 | Mkoani | 30/4/2013 | KENGEJA | 21119 | F | 9 | 3 |
| Pemba | 2013 | Mkoani | 30/4/2013 | KENGEJA | 21120 | F | 9 | 3 |
| Pemba | 2013 | Mkoani | 30/4/2013 | KENGEJA | 21121 | F | 9 | 3 |
| Pemba | 2013 | Mkoani | 30/4/2013 | KENGEJA | 21122 | F | 9 | 3 |
| Pemba | 2013 | Mkoani | 30/4/2013 | KENGEJA | 21123 | F | 10 | 3 |
| Pemba | 2013 | Mkoani | 30/4/2013 | KENGEJA | 21124 | F | 9 | 3 |
| Pemba | 2013 | Mkoani | 30/4/2013 | KENGEJA | 21125 | F | 9 | 3 |
| Pemba | 2013 | Mkoani | 30/4/2013 | KENGEJA | 21126 | M | 9 | 3 |
| Pemba | 2013 | Mkoani | 30/4/2013 | KENGEJA | 21127 | F | 9 | 3 |
| Pemba | 2013 | Mkoani | 30/4/2013 | KENGEJA | 21128 | M | 9 | 3 |
| Pemba | 2013 | Mkoani | 30/4/2013 | KENGEJA | 21129 | M | 9 | 3 |
| Pemba | 2013 | Mkoani | 30/4/2013 | KENGEJA | 21130 | M | 10 | 3 |
| Pemba | 2013 | Mkoani | 22/2/2013 | NGWACHANI | 25001 | F | 11 | 4 |
| Pemba | 2013 | Mkoani | 22/2/2013 | NGWACHANI | 25002 | F | 10 | 4 |
| Pemba | 2013 | Mkoani | 22/2/2013 | NGWACHANI | 25003 | F | 11 | 4 |
| Pemba | 2013 | Mkoani | 22/2/2013 | NGWACHANI | 25004 | F | 11 | 4 |
| Pemba | 2013 | Mkoani | 22/2/2013 | NGWACHANI | 25005 | F | 11 | 4 |
| Pemba | 2013 | Mkoani | 22/2/2013 | NGWACHANI | 25006 | F | 11 | 4 |
| Pemba | 2013 | Mkoani | 22/2/2013 | NGWACHANI | 25007 | F | 11 | 4 |
| Pemba | 2013 | Mkoani | 22/2/2013 | NGWACHANI | 25008 | F | 11 | 4 |
| Pemba | 2013 | Mkoani | 22/2/2013 | NGWACHANI | 25009 | F | 12 | 4 |
| Pemba | 2013 | Mkoani | 22/2/2013 | NGWACHANI | 25010 | F | 12 | 4 |
| Pemba | 2013 | Mkoani | 22/2/2013 | NGWACHANI | 25011 | F | 11 | 4 |
| Pemba | 2013 | Mkoani | 22/2/2013 | NGWACHANI | 25012 | F | 12 | 4 |
| Pemba | 2013 | Mkoani | 22/2/2013 | NGWACHANI | 25013 | F | 12 | 4 |
| Pemba | 2013 | Mkoani | 22/2/2013 | NGWACHANI | 25014 | F | 12 | 4 |
| Pemba | 2013 | Mkoani | 22/2/2013 | NGWACHANI | 25015 | F | 11 | 4 |
| Pemba | 2013 | Mkoani | 22/2/2013 | NGWACHANI | 25016 | M | 12 | 4 |
| Pemba | 2013 | Mkoani | 22/2/2013 | NGWACHANI | 25017 | M | 12 | 4 |
| Pemba | 2013 | Mkoani | 22/2/2013 | NGWACHANI | 25018 | M | 12 | 4 |
| Pemba | 2013 | Mkoani | 22/2/2013 | NGWACHANI | 25019 | M | 12 | 4 |
| Pemba | 2013 | Mkoani | 22/2/2013 | NGWACHANI | 25020 | M | 11 | 4 |
| Pemba | 2013 | Mkoani | 22/2/2013 | NGWACHANI | 25021 | M | 11 | 4 |
| Pemba | 2013 | Mkoani | 22/2/2013 | NGWACHANI | 25022 | M | 11 | 4 |
| Pemba | 2013 | Mkoani | 22/2/2013 | NGWACHANI | 25023 | M | 12 | 4 |
| Pemba | 2013 | Mkoani | 22/2/2013 | NGWACHANI | 25024 | M | 12 | 4 |
| Pemba | 2013 | Mkoani | 22/2/2013 | NGWACHANI | 25025 | M | 11 | 4 |
| Pemba | 2013 | Mkoani | 22/2/2013 | NGWACHANI | 25026 | M | 11 | 4 |
| Pemba | 2013 | Mkoani | 22/2/2013 | NGWACHANI | 25027 | M | 11 | 4 |
| Pemba | 2013 | Mkoani | 22/2/2013 | NGWACHANI | 25028 | M | 11 | 4 |
| Pemba | 2013 | Mkoani | 22/2/2013 | NGWACHANI | 25029 | M | 11 | 4 |
| Pemba | 2013 | Mkoani | 22/2/2013 | NGWACHANI | 25030 | M | 12 | 4 |
| Pemba | 2013 | Mkoani | 22/2/2013 | NGWACHANI | 25031 | M | 11 | 4 |
| Pemba | 2013 | Mkoani | 22/2/2013 | NGWACHANI | 25032 | M | 11 | 4 |

| | | | | | | | | |
|---|---|---|---|---|---|---|---|---|
| Pemba | 2013 | Mkoani | 22/2/2013 | NGWACHANI | 25033 | M | 11 | 4 |
| Pemba | 2013 | Mkoani | 22/2/2013 | NGWACHANI | 25034 | M | 12 | 4 |
| Pemba | 2013 | Mkoani | 22/2/2013 | NGWACHANI | 25035 | M | 12 | 4 |
| Pemba | 2013 | Mkoani | 22/2/2013 | NGWACHANI | 25036 | M | 12 | 4 |
| Pemba | 2013 | Mkoani | 22/2/2013 | NGWACHANI | 25037 | M | 12 | 4 |
| Pemba | 2013 | Mkoani | 22/2/2013 | NGWACHANI | 25038 | M | 10 | 4 |
| Pemba | 2013 | Mkoani | 22/2/2013 | NGWACHANI | 25039 | M | 11 | 4 |
| Pemba | 2013 | Mkoani | 22/2/2013 | NGWACHANI | 25040 | M | 12 | 4 |
| Pemba | 2013 | Mkoani | 22/2/2013 | NGWACHANI | 25041 | M | 12 | 4 |
| Pemba | 2013 | Mkoani | 22/2/2013 | NGWACHANI | 25042 | M | 12 | 4 |
| Pemba | 2013 | Mkoani | 22/2/2013 | NGWACHANI | 25043 | M | 12 | 4 |
| Pemba | 2013 | Mkoani | 22/2/2013 | NGWACHANI | 25044 | M | 11 | 4 |
| Pemba | 2013 | Mkoani | 22/2/2013 | NGWACHANI | 25045 | M | 10 | 4 |
| Pemba | 2013 | Mkoani | 22/2/2013 | NGWACHANI | 25046 | M | 10 | 4 |
| Pemba | 2013 | Mkoani | 22/2/2013 | NGWACHANI | 25047 | M | 11 | 4 |
| Pemba | 2013 | Mkoani | 22/2/2013 | NGWACHANI | 25048 | M | 10 | 4 |
| Pemba | 2013 | Mkoani | 22/2/2013 | NGWACHANI | 25049 | M | 12 | 4 |
| Pemba | 2013 | Mkoani | 22/2/2013 | NGWACHANI | 25050 | M | 12 | 4 |
| Pemba | 2013 | Mkoani | 22/2/2013 | NGWACHANI | 25051 | M | 12 | 4 |
| Pemba | 2013 | Mkoani | 22/2/2013 | NGWACHANI | 25052 | M | 11 | 4 |
| Pemba | 2013 | Mkoani | 22/2/2013 | NGWACHANI | 25053 | F | 10 | 4 |
| Pemba | 2013 | Mkoani | 22/2/2013 | NGWACHANI | 25054 | F | 11 | 4 |
| Pemba | 2013 | Mkoani | 22/2/2013 | NGWACHANI | 25055 | F | 11 | 4 |
| Pemba | 2013 | Mkoani | 22/2/2013 | NGWACHANI | 25056 | F | 11 | 4 |
| Pemba | 2013 | Mkoani | 22/2/2013 | NGWACHANI | 25057 | F | 10 | 4 |
| Pemba | 2013 | Mkoani | 22/2/2013 | NGWACHANI | 25058 | F | 11 | 4 |
| Pemba | 2013 | Mkoani | 22/2/2013 | NGWACHANI | 25059 | F | 10 | 4 |
| Pemba | 2013 | Mkoani | 22/2/2013 | NGWACHANI | 25060 | F | 10 | 4 |
| Pemba | 2013 | Mkoani | 22/2/2013 | NGWACHANI | 25061 | F | 11 | 4 |
| Pemba | 2013 | Mkoani | 22/2/2013 | NGWACHANI | 25062 | F | 10 | 4 |
| Pemba | 2013 | Mkoani | 22/2/2013 | NGWACHANI | 25063 | F | 10 | 4 |
| Pemba | 2013 | Mkoani | 22/2/2013 | NGWACHANI | 25064 | F | 12 | 4 |
| Pemba | 2013 | Mkoani | 22/2/2013 | NGWACHANI | 25065 | F | 10 | 4 |
| Pemba | 2013 | Mkoani | 22/2/2013 | NGWACHANI | 25066 | F | 10 | 4 |
| Pemba | 2013 | Mkoani | 22/2/2013 | NGWACHANI | 25067 | F | 11 | 4 |
| Pemba | 2013 | Mkoani | 22/2/2013 | NGWACHANI | 25068 | F | 11 | 4 |
| Pemba | 2013 | Mkoani | 22/2/2013 | NGWACHANI | 25069 | F | 11 | 4 |
| Pemba | 2013 | Mkoani | 22/2/2013 | NGWACHANI | 25070 | F | 10 | 4 |
| Pemba | 2013 | Mkoani | 22/2/2013 | NGWACHANI | 25071 | F | 10 | 4 |
| Pemba | 2013 | Mkoani | 22/2/2013 | NGWACHANI | 25072 | F | 11 | 4 |
| Pemba | 2013 | Mkoani | 22/2/2013 | NGWACHANI | 25073 | F | 10 | 4 |
| Pemba | 2013 | Mkoani | 22/2/2013 | NGWACHANI | 25074 | F | 10 | 4 |
| Pemba | 2013 | Mkoani | 22/2/2013 | NGWACHANI | 25075 | F | 11 | 4 |
| Pemba | 2013 | Mkoani | 22/2/2013 | NGWACHANI | 25076 | F | 10 | 4 |
| Pemba | 2013 | Mkoani | 22/2/2013 | NGWACHANI | 25077 | F | 11 | 4 |
| Pemba | 2013 | Mkoani | 22/2/2013 | NGWACHANI | 25078 | F | 11 | 4 |
| Pemba | 2013 | Mkoani | 22/2/2013 | NGWACHANI | 25079 | F | 10 | 4 |
| Pemba | 2013 | Mkoani | 22/2/2013 | NGWACHANI | 25080 | F | 11 | 4 |
| Pemba | 2013 | Mkoani | 22/2/2013 | NGWACHANI | 25081 | F | 12 | 4 |
| Pemba | 2013 | Mkoani | 22/2/2013 | NGWACHANI | 25082 | F | 12 | 4 |

| | | | | | | | | |
|---|---|---|---|---|---|---|---|---|
| Pemba | 2013 | Mkoani | 22/2/2013 | NGWACHANI | 25083 | F | 10 | 4 |
| Pemba | 2013 | Mkoani | 22/2/2013 | NGWACHANI | 25084 | F | 11 | 4 |
| Pemba | 2013 | Mkoani | 22/2/2013 | NGWACHANI | 25085 | F | 11 | 4 |
| Pemba | 2013 | Mkoani | 22/2/2013 | NGWACHANI | 25086 | F | 10 | 4 |
| Pemba | 2013 | Mkoani | 22/2/2013 | NGWACHANI | 25087 | F | 11 | 4 |
| Pemba | 2013 | Mkoani | 22/2/2013 | NGWACHANI | 25088 | F | 10 | 4 |
| Pemba | 2013 | Mkoani | 22/2/2013 | NGWACHANI | 25089 | M | 10 | 4 |
| Pemba | 2013 | Mkoani | 22/2/2013 | NGWACHANI | 25090 | M | 11 | 4 |
| Pemba | 2013 | Mkoani | 22/2/2013 | NGWACHANI | 25091 | M | 11 | 4 |
| Pemba | 2013 | Mkoani | 22/2/2013 | NGWACHANI | 25092 | M | 10 | 4 |
| Pemba | 2013 | Mkoani | 22/2/2013 | NGWACHANI | 25093 | M | 10 | 4 |
| Pemba | 2013 | Mkoani | 22/2/2013 | NGWACHANI | 25094 | M | 12 | 4 |
| Pemba | 2013 | Mkoani | 22/2/2013 | NGWACHANI | 25095 | M | 10 | 4 |
| Pemba | 2013 | Mkoani | 22/2/2013 | NGWACHANI | 25096 | M | 11 | 4 |
| Pemba | 2013 | Mkoani | 22/2/2013 | NGWACHANI | 25097 | M | 11 | 4 |
| Pemba | 2013 | Mkoani | 22/2/2013 | NGWACHANI | 25098 | M | 11 | 4 |
| Pemba | 2013 | Mkoani | 22/2/2013 | NGWACHANI | 25099 | M | 10 | 4 |
| Pemba | 2013 | Mkoani | 22/2/2013 | NGWACHANI | 25100 | M | 10 | 4 |
| Pemba | 2013 | Mkoani | 22/2/2013 | NGWACHANI | 25101 | M | 10 | 3 |
| Pemba | 2013 | Mkoani | 22/2/2013 | NGWACHANI | 25102 | M | 10 | 3 |
| Pemba | 2013 | Mkoani | 22/2/2013 | NGWACHANI | 25103 | M | 10 | 3 |
| Pemba | 2013 | Mkoani | 22/2/2013 | NGWACHANI | 25104 | M | 11 | 3 |
| Pemba | 2013 | Mkoani | 22/2/2013 | NGWACHANI | 25105 | M | 10 | 3 |
| Pemba | 2013 | Mkoani | 22/2/2013 | NGWACHANI | 25106 | M | 10 | 3 |
| Pemba | 2013 | Mkoani | 22/2/2013 | NGWACHANI | 25107 | M | 10 | 3 |
| Pemba | 2013 | Mkoani | 22/2/2013 | NGWACHANI | 25108 | M | 11 | 3 |
| Pemba | 2013 | Mkoani | 22/2/2013 | NGWACHANI | 25109 | M | 10 | 3 |
| Pemba | 2013 | Mkoani | 22/2/2013 | NGWACHANI | 25110 | M | 9 | 3 |
| Pemba | 2013 | Mkoani | 22/2/2013 | NGWACHANI | 25111 | M | 9 | 3 |
| Pemba | 2013 | Mkoani | 22/2/2013 | NGWACHANI | 25112 | M | 10 | 3 |
| Pemba | 2013 | Mkoani | 22/2/2013 | NGWACHANI | 25113 | M | 10 | 3 |
| Pemba | 2013 | Mkoani | 22/2/2013 | NGWACHANI | 25114 | M | 10 | 3 |
| Pemba | 2013 | Mkoani | 22/2/2013 | NGWACHANI | 25115 | M | 9 | 3 |
| Pemba | 2013 | Mkoani | 22/2/2013 | NGWACHANI | 25116 | F | 10 | 3 |
| Pemba | 2013 | Mkoani | 22/2/2013 | NGWACHANI | 25117 | F | 10 | 3 |
| Pemba | 2013 | Mkoani | 22/2/2013 | NGWACHANI | 25118 | F | 11 | 3 |
| Pemba | 2013 | Mkoani | 22/2/2013 | NGWACHANI | 25119 | F | 11 | 3 |
| Pemba | 2013 | Mkoani | 22/2/2013 | NGWACHANI | 25120 | F | 10 | 3 |
| Pemba | 2013 | Mkoani | 22/2/2013 | NGWACHANI | 25121 | F | 10 | 3 |
| Pemba | 2013 | Mkoani | 22/2/2013 | NGWACHANI | 25122 | F | 10 | 3 |
| Pemba | 2013 | Mkoani | 22/2/2013 | NGWACHANI | 25123 | F | 9 | 3 |
| Pemba | 2013 | Mkoani | 22/2/2013 | NGWACHANI | 25124 | F | 9 | 3 |
| Pemba | 2013 | Mkoani | 22/2/2013 | NGWACHANI | 25125 | F | 10 | 3 |
| Pemba | 2013 | Mkoani | 22/2/2013 | NGWACHANI | 25126 | F | 10 | 3 |
| Pemba | 2013 | Mkoani | 22/2/2013 | NGWACHANI | 25127 | F | 9 | 3 |
| Pemba | 2013 | Mkoani | 22/2/2013 | NGWACHANI | 25128 | F | 10 | 3 |
| Pemba | 2013 | Mkoani | 22/2/2013 | NGWACHANI | 25129 | F | 9 | 3 |
| Pemba | 2013 | Mkoani | 22/2/2013 | NGWACHANI | 25130 | F | 10 | 3 |
| Pemba | 2013 | Wete | 17/4/2013 | OLE | 26001 | F | 12 | 4 |
| Pemba | 2013 | Wete | 17/4/2013 | OLE | 26002 | F | 11 | 4 |

| | | | | | | | | |
|---|---|---|---|---|---|---|---|---|
| Pemba | 2013 | Wete | 17/4/2013 | OLE | 26003 | F | 11 | 4 |
| Pemba | 2013 | Wete | 17/4/2013 | OLE | 26004 | F | 12 | 4 |
| Pemba | 2013 | Wete | 17/4/2013 | OLE | 26005 | F | 12 | 4 |
| Pemba | 2013 | Wete | 17/4/2013 | OLE | 26006 | F | 10 | 4 |
| Pemba | 2013 | Wete | 17/4/2013 | OLE | 26007 | F | 10 | 4 |
| Pemba | 2013 | Wete | 17/4/2013 | OLE | 26008 | F | 11 | 4 |
| Pemba | 2013 | Wete | 17/4/2013 | OLE | 26009 | F | 11 | 4 |
| Pemba | 2013 | Wete | 17/4/2013 | OLE | 26010 | F | 11 | 4 |
| Pemba | 2013 | Wete | 17/4/2013 | OLE | 26011 | F | 11 | 4 |
| Pemba | 2013 | Wete | 17/4/2013 | OLE | 26012 | F | 10 | 4 |
| Pemba | 2013 | Wete | 17/4/2013 | OLE | 26013 | F | 12 | 4 |
| Pemba | 2013 | Wete | 17/4/2013 | OLE | 26014 | F | 10 | 4 |
| Pemba | 2013 | Wete | 17/4/2013 | OLE | 26015 | F | 11 | 4 |
| Pemba | 2013 | Wete | 17/4/2013 | OLE | 26016 | F | 12 | 4 |
| Pemba | 2013 | Wete | 17/4/2013 | OLE | 26017 | F | 12 | 4 |
| Pemba | 2013 | Wete | 17/4/2013 | OLE | 26018 | F | 12 | 4 |
| Pemba | 2013 | Wete | 17/4/2013 | OLE | 26019 | F | 12 | 4 |
| Pemba | 2013 | Wete | 17/4/2013 | OLE | 26020 | F | 11 | 4 |
| Pemba | 2013 | Wete | 17/4/2013 | OLE | 26021 | F | 11 | 4 |
| Pemba | 2013 | Wete | 17/4/2013 | OLE | 26022 | F | 12 | 4 |
| Pemba | 2013 | Wete | 17/4/2013 | OLE | 26023 | F | 10 | 4 |
| Pemba | 2013 | Wete | 17/4/2013 | OLE | 26024 | F | 10 | 4 |
| Pemba | 2013 | Wete | 17/4/2013 | OLE | 26025 | F | 10 | 4 |
| Pemba | 2013 | Wete | 17/4/2013 | OLE | 26026 | F | 11 | 4 |
| Pemba | 2013 | Wete | 17/4/2013 | OLE | 26027 | F | 11 | 4 |
| Pemba | 2013 | Wete | 17/4/2013 | OLE | 26028 | F | 11 | 4 |
| Pemba | 2013 | Wete | 17/4/2013 | OLE | 26029 | F | 11 | 4 |
| Pemba | 2013 | Wete | 17/4/2013 | OLE | 26030 | F | 11 | 4 |
| Pemba | 2013 | Wete | 17/4/2013 | OLE | 26031 | F | 11 | 4 |
| Pemba | 2013 | Wete | 17/4/2013 | OLE | 26032 | F | 10 | 4 |
| Pemba | 2013 | Wete | 17/4/2013 | OLE | 26033 | F | 10 | 4 |
| Pemba | 2013 | Wete | 17/4/2013 | OLE | 26034 | F | 10 | 4 |
| Pemba | 2013 | Wete | 17/4/2013 | OLE | 26035 | M | 12 | 4 |
| Pemba | 2013 | Wete | 17/4/2013 | OLE | 26036 | M | 10 | 4 |
| Pemba | 2013 | Wete | 17/4/2013 | OLE | 26037 | M | 11 | 4 |
| Pemba | 2013 | Wete | 17/4/2013 | OLE | 26038 | M | 12 | 4 |
| Pemba | 2013 | Wete | 17/4/2013 | OLE | 26039 | M | 11 | 4 |
| Pemba | 2013 | Wete | 17/4/2013 | OLE | 26040 | M | 12 | 4 |
| Pemba | 2013 | Wete | 17/4/2013 | OLE | 26041 | M | 11 | 4 |
| Pemba | 2013 | Wete | 17/4/2013 | OLE | 26042 | M | 12 | 4 |
| Pemba | 2013 | Wete | 17/4/2013 | OLE | 26043 | M | 10 | 4 |
| Pemba | 2013 | Wete | 17/4/2013 | OLE | 26044 | M | 11 | 4 |
| Pemba | 2013 | Wete | 17/4/2013 | OLE | 26045 | M | 10 | 4 |
| Pemba | 2013 | Wete | 17/4/2013 | OLE | 26046 | M | 12 | 4 |
| Pemba | 2013 | Wete | 17/4/2013 | OLE | 26047 | M | 11 | 4 |
| Pemba | 2013 | Wete | 17/4/2013 | OLE | 26048 | M | 11 | 4 |
| Pemba | 2013 | Wete | 17/4/2013 | OLE | 26049 | M | 11 | 4 |
| Pemba | 2013 | Wete | 17/4/2013 | OLE | 26050 | M | 11 | 4 |
| Pemba | 2013 | Wete | 17/4/2013 | OLE | 26051 | M | 10 | 4 |
| Pemba | 2013 | Wete | 17/4/2013 | OLE | 26052 | M | 12 | 4 |

| | | | | | | | | |
|---|---|---|---|---|---|---|---|---|
| Pemba | 2013 | Wete | 17/4/2013 | OLE | 26053 | M | 11 | 4 |
| Pemba | 2013 | Wete | 17/4/2013 | OLE | 26054 | M | 11 | 4 |
| Pemba | 2013 | Wete | 17/4/2013 | OLE | 26055 | M | 12 | 4 |
| Pemba | 2013 | Wete | 17/4/2013 | OLE | 26056 | M | 12 | 4 |
| Pemba | 2013 | Wete | 17/4/2013 | OLE | 26057 | M | 12 | 4 |
| Pemba | 2013 | Wete | 17/4/2013 | OLE | 26058 | M | 12 | 4 |
| Pemba | 2013 | Wete | 17/4/2013 | OLE | 26059 | M | 11 | 4 |
| Pemba | 2013 | Wete | 17/4/2013 | OLE | 26060 | M | 9 | 4 |
| Pemba | 2013 | Wete | 17/4/2013 | OLE | 26061 | M | 11 | 4 |
| Pemba | 2013 | Wete | 17/4/2013 | OLE | 26062 | M | 10 | 4 |
| Pemba | 2013 | Wete | 17/4/2013 | OLE | 26063 | M | 12 | 4 |
| Pemba | 2013 | Wete | 17/4/2013 | OLE | 26064 | M | 11 | 4 |
| Pemba | 2013 | Wete | 17/4/2013 | OLE | 26065 | M | 12 | 4 |
| Pemba | 2013 | Wete | 17/4/2013 | OLE | 26066 | M | 9 | 3 |
| Pemba | 2013 | Wete | 17/4/2013 | OLE | 26067 | F | 10 | 3 |
| Pemba | 2013 | Wete | 17/4/2013 | OLE | 26068 | F | 10 | 3 |
| Pemba | 2013 | Wete | 17/4/2013 | OLE | 26069 | F | 9 | 3 |
| Pemba | 2013 | Wete | 17/4/2013 | OLE | 26070 | F | 9 | 3 |
| Pemba | 2013 | Wete | 17/4/2013 | OLE | 26071 | F | 9 | 3 |
| Pemba | 2013 | Wete | 17/4/2013 | OLE | 26072 | F | 11 | 3 |
| Pemba | 2013 | Wete | 17/4/2013 | OLE | 26073 | F | 9 | 3 |
| Pemba | 2013 | Wete | 17/4/2013 | OLE | 26074 | F | 9 | 3 |
| Pemba | 2013 | Wete | 17/4/2013 | OLE | 26075 | F | 9 | 3 |
| Pemba | 2013 | Wete | 17/4/2013 | OLE | 26076 | F | 9 | 3 |
| Pemba | 2013 | Wete | 17/4/2013 | OLE | 26077 | F | 9 | 3 |
| Pemba | 2013 | Wete | 17/4/2013 | OLE | 26078 | F | 9 | 3 |
| Pemba | 2013 | Wete | 17/4/2013 | OLE | 26079 | F | 10 | 3 |
| Pemba | 2013 | Wete | 17/4/2013 | OLE | 26080 | F | 10 | 3 |
| Pemba | 2013 | Wete | 17/4/2013 | OLE | 26081 | F | 9 | 3 |
| Pemba | 2013 | Wete | 17/4/2013 | OLE | 26082 | F | 10 | 3 |
| Pemba | 2013 | Wete | 17/4/2013 | OLE | 26083 | F | 10 | 3 |
| Pemba | 2013 | Wete | 17/4/2013 | OLE | 26084 | F | 10 | 3 |
| Pemba | 2013 | Wete | 17/4/2013 | OLE | 26085 | M | 11 | 3 |
| Pemba | 2013 | Wete | 17/4/2013 | OLE | 26086 | M | 9 | 3 |
| Pemba | 2013 | Wete | 17/4/2013 | OLE | 26087 | M | 9 | 3 |
| Pemba | 2013 | Wete | 17/4/2013 | OLE | 26088 | M | 10 | 3 |
| Pemba | 2013 | Wete | 17/4/2013 | OLE | 26089 | M | 11 | 3 |
| Pemba | 2013 | Wete | 17/4/2013 | OLE | 26090 | M | 11 | 3 |
| Pemba | 2013 | Wete | 17/4/2013 | OLE | 26091 | M | 12 | 3 |
| Pemba | 2013 | Wete | 17/4/2013 | OLE | 26092 | M | 11 | 3 |
| Pemba | 2013 | Wete | 17/4/2013 | OLE | 26093 | M | 9 | 3 |
| Pemba | 2013 | Wete | 17/4/2013 | OLE | 26094 | M | 11 | 3 |
| Pemba | 2013 | Wete | 17/4/2013 | OLE | 26095 | M | 12 | 3 |
| Pemba | 2013 | Wete | 17/4/2013 | OLE | 26096 | M | 11 | 3 |
| Pemba | 2013 | Wete | 17/4/2013 | OLE | 26097 | M | 10 | 3 |
| Pemba | 2013 | Wete | 17/4/2013 | OLE | 26098 | M | 9 | 3 |
| Pemba | 2013 | Wete | 17/4/2013 | OLE | 26099 | M | 10 | 3 |
| Pemba | 2013 | Wete | 17/4/2013 | OLE | 26100 | M | 10 | 3 |
| Pemba | 2013 | Wete | 17/4/2013 | OLE | 26101 | M | 11 | 3 |
| Pemba | 2013 | Wete | 17/4/2013 | OLE | 26102 | F | 10 | 3 |

| | | | | | | | | |
|---|---|---|---|---|---|---|---|---|
| Pemba | 2013 | Wete | 17/4/2013 | OLE | 26103 | F | 9 | 3 |
| Pemba | 2013 | Wete | 17/4/2013 | OLE | 26104 | F | 9 | 3 |
| Pemba | 2013 | Wete | 17/4/2013 | OLE | 26105 | F | 10 | 3 |
| Pemba | 2013 | Wete | 17/4/2013 | OLE | 26106 | F | 9 | 3 |
| Pemba | 2013 | Wete | 17/4/2013 | OLE | 26107 | F | 8 | 3 |
| Pemba | 2013 | Wete | 17/4/2013 | OLE | 26108 | F | 9 | 3 |
| Pemba | 2013 | Wete | 17/4/2013 | OLE | 26109 | F | 10 | 3 |
| Pemba | 2013 | Wete | 17/4/2013 | OLE | 26110 | F | 9 | 3 |
| Pemba | 2013 | Wete | 17/4/2013 | OLE | 26111 | F | 9 | 3 |
| Pemba | 2013 | Wete | 17/4/2013 | OLE | 26112 | F | 9 | 3 |
| Pemba | 2013 | Wete | 17/4/2013 | OLE | 26113 | F | 9 | 3 |
| Pemba | 2013 | Wete | 17/4/2013 | OLE | 26114 | F | 9 | 3 |
| Pemba | 2013 | Wete | 17/4/2013 | OLE | 26115 | F | 9 | 3 |
| Pemba | 2013 | Wete | 17/4/2013 | OLE | 26116 | F | 10 | 3 |
| Pemba | 2013 | Wete | 17/4/2013 | OLE | 26117 | F | 9 | 3 |
| Pemba | 2013 | Wete | 17/4/2013 | OLE | 26118 | F | 9 | 3 |
| Pemba | 2013 | Wete | 17/4/2013 | OLE | 26119 | F | 9 | 3 |
| Pemba | 2013 | Wete | 17/4/2013 | OLE | 26120 | F | 10 | 3 |
| Pemba | 2013 | Wete | 17/4/2013 | OLE | 26121 | M | 12 | 3 |
| Pemba | 2013 | Wete | 17/4/2013 | OLE | 26122 | M | 11 | 3 |
| Pemba | 2013 | Wete | 17/4/2013 | OLE | 26123 | M | 10 | 3 |
| Pemba | 2013 | Wete | 17/4/2013 | OLE | 26124 | M | 11 | 3 |
| Pemba | 2013 | Wete | 17/4/2013 | OLE | 26125 | M | 10 | 3 |
| Pemba | 2013 | Wete | 17/4/2013 | OLE | 26126 | M | 12 | 3 |
| Pemba | 2013 | Wete | 17/4/2013 | OLE | 26127 | M | 12 | 3 |
| Pemba | 2013 | Wete | 17/4/2013 | OLE | 26128 | M | 12 | 3 |
| Pemba | 2013 | Wete | 17/4/2013 | OLE | 26129 | M | 12 | 3 |
| Pemba | 2013 | Wete | 17/4/2013 | OLE | 26130 | M | 10 | 3 |
| Pemba | 2013 | Micheweni | 18/3/2013 | MAKANGALE | 40001 | F | 12 | 4 |
| Pemba | 2013 | Micheweni | 18/3/2013 | MAKANGALE | 40002 | F | 12 | 4 |
| Pemba | 2013 | Micheweni | 18/3/2013 | MAKANGALE | 40003 | F | 10 | 4 |
| Pemba | 2013 | Micheweni | 18/3/2013 | MAKANGALE | 40004 | F | 10 | 4 |
| Pemba | 2013 | Micheweni | 18/3/2013 | MAKANGALE | 40005 | F | 12 | 4 |
| Pemba | 2013 | Micheweni | 18/3/2013 | MAKANGALE | 40006 | F | 10 | 4 |
| Pemba | 2013 | Micheweni | 18/3/2013 | MAKANGALE | 40007 | F | 10 | 4 |
| Pemba | 2013 | Micheweni | 18/3/2013 | MAKANGALE | 40008 | F | 11 | 4 |
| Pemba | 2013 | Micheweni | 18/3/2013 | MAKANGALE | 40009 | F | 10 | 4 |
| Pemba | 2013 | Micheweni | 18/3/2013 | MAKANGALE | 40010 | F | 10 | 4 |
| Pemba | 2013 | Micheweni | 18/3/2013 | MAKANGALE | 40011 | F | 12 | 4 |
| Pemba | 2013 | Micheweni | 18/3/2013 | MAKANGALE | 40012 | F | 9 | 4 |
| Pemba | 2013 | Micheweni | 18/3/2013 | MAKANGALE | 40013 | F | 9 | 4 |
| Pemba | 2013 | Micheweni | 18/3/2013 | MAKANGALE | 40014 | F | 10 | 4 |
| Pemba | 2013 | Micheweni | 18/3/2013 | MAKANGALE | 40015 | F | 11 | 4 |
| Pemba | 2013 | Micheweni | 18/3/2013 | MAKANGALE | 40016 | F | 12 | 4 |
| Pemba | 2013 | Micheweni | 18/3/2013 | MAKANGALE | 40017 | F | 10 | 4 |
| Pemba | 2013 | Micheweni | 18/3/2013 | MAKANGALE | 40018 | F | 10 | 4 |
| Pemba | 2013 | Micheweni | 18/3/2013 | MAKANGALE | 40019 | F | 10 | 4 |
| Pemba | 2013 | Micheweni | 18/3/2013 | MAKANGALE | 40020 | F | 12 | 4 |
| Pemba | 2013 | Micheweni | 18/3/2013 | MAKANGALE | 40021 | F | 10 | 4 |
| Pemba | 2013 | Micheweni | 18/3/2013 | MAKANGALE | 40022 | F | 10 | 4 |

| | | | | | | | | |
|---|---|---|---|---|---|---|---|---|
| Pemba | 2013 | Micheweni | 18/3/2013 | MAKANGALE | 40023 | F | 10 | 4 |
| Pemba | 2013 | Micheweni | 18/3/2013 | MAKANGALE | 40024 | F | 10 | 4 |
| Pemba | 2013 | Micheweni | 18/3/2013 | MAKANGALE | 40025 | F | 12 | 4 |
| Pemba | 2013 | Micheweni | 18/3/2013 | MAKANGALE | 40026 | F | 10 | 4 |
| Pemba | 2013 | Micheweni | 18/3/2013 | MAKANGALE | 40027 | F | 12 | 4 |
| Pemba | 2013 | Micheweni | 18/3/2013 | MAKANGALE | 40028 | F | 9 | 4 |
| Pemba | 2013 | Micheweni | 18/3/2013 | MAKANGALE | 40029 | F | 9 | 4 |
| Pemba | 2013 | Micheweni | 18/3/2013 | MAKANGALE | 40030 | F | 9 | 4 |
| Pemba | 2013 | Micheweni | 18/3/2013 | MAKANGALE | 40031 | F | 12 | 4 |
| Pemba | 2013 | Micheweni | 18/3/2013 | MAKANGALE | 40032 | F | 10 | 4 |
| Pemba | 2013 | Micheweni | 18/3/2013 | MAKANGALE | 40033 | F | 12 | 4 |
| Pemba | 2013 | Micheweni | 18/3/2013 | MAKANGALE | 40034 | F | 10 | 4 |
| Pemba | 2013 | Micheweni | 18/3/2013 | MAKANGALE | 40035 | F | 10 | 4 |
| Pemba | 2013 | Micheweni | 18/3/2013 | MAKANGALE | 40036 | F | 9 | 4 |
| Pemba | 2013 | Micheweni | 18/3/2013 | MAKANGALE | 40037 | F | 10 | 4 |
| Pemba | 2013 | Micheweni | 18/3/2013 | MAKANGALE | 40038 | F | 11 | 4 |
| Pemba | 2013 | Micheweni | 18/3/2013 | MAKANGALE | 40039 | F | 10 | 4 |
| Pemba | 2013 | Micheweni | 18/3/2013 | MAKANGALE | 40040 | M | 12 | 4 |
| Pemba | 2013 | Micheweni | 18/3/2013 | MAKANGALE | 40041 | M | 12 | 4 |
| Pemba | 2013 | Micheweni | 18/3/2013 | MAKANGALE | 40042 | M | 12 | 4 |
| Pemba | 2013 | Micheweni | 18/3/2013 | MAKANGALE | 40043 | M | 12 | 4 |
| Pemba | 2013 | Micheweni | 18/3/2013 | MAKANGALE | 40044 | M | 11 | 4 |
| Pemba | 2013 | Micheweni | 18/3/2013 | MAKANGALE | 40045 | M | 10 | 4 |
| Pemba | 2013 | Micheweni | 18/3/2013 | MAKANGALE | 40046 | M | 10 | 4 |
| Pemba | 2013 | Micheweni | 18/3/2013 | MAKANGALE | 40047 | M | 12 | 4 |
| Pemba | 2013 | Micheweni | 18/3/2013 | MAKANGALE | 40048 | M | 12 | 4 |
| Pemba | 2013 | Micheweni | 18/3/2013 | MAKANGALE | 40049 | M | 11 | 4 |
| Pemba | 2013 | Micheweni | 18/3/2013 | MAKANGALE | 40050 | M | 12 | 4 |
| Pemba | 2013 | Micheweni | 18/3/2013 | MAKANGALE | 40051 | M | 10 | 4 |
| Pemba | 2013 | Micheweni | 18/3/2013 | MAKANGALE | 40052 | M | 12 | 4 |
| Pemba | 2013 | Micheweni | 18/3/2013 | MAKANGALE | 40053 | M | 12 | 4 |
| Pemba | 2013 | Micheweni | 18/3/2013 | MAKANGALE | 40054 | M | 10 | 4 |
| Pemba | 2013 | Micheweni | 18/3/2013 | MAKANGALE | 40055 | M | 10 | 4 |
| Pemba | 2013 | Micheweni | 18/3/2013 | MAKANGALE | 40056 | M | 11 | 4 |
| Pemba | 2013 | Micheweni | 18/3/2013 | MAKANGALE | 40057 | M | 12 | 4 |
| Pemba | 2013 | Micheweni | 18/3/2013 | MAKANGALE | 40058 | M | 10 | 4 |
| Pemba | 2013 | Micheweni | 18/3/2013 | MAKANGALE | 40059 | M | 11 | 4 |
| Pemba | 2013 | Micheweni | 18/3/2013 | MAKANGALE | 40060 | M | 12 | 4 |
| Pemba | 2013 | Micheweni | 18/3/2013 | MAKANGALE | 40061 | M | 10 | 4 |
| Pemba | 2013 | Micheweni | 18/3/2013 | MAKANGALE | 40062 | M | 12 | 4 |
| Pemba | 2013 | Micheweni | 18/3/2013 | MAKANGALE | 40063 | M | 10 | 4 |
| Pemba | 2013 | Micheweni | 18/3/2013 | MAKANGALE | 40064 | M | 10 | 4 |
| Pemba | 2013 | Micheweni | 18/3/2013 | MAKANGALE | 40065 | M | 12 | 4 |
| Pemba | 2013 | Micheweni | 18/3/2013 | MAKANGALE | 40066 | F | 12 | 4 |
| Pemba | 2013 | Micheweni | 18/3/2013 | MAKANGALE | 40067 | F | 9 | 4 |
| Pemba | 2013 | Micheweni | 18/3/2013 | MAKANGALE | 40068 | F | 9 | 4 |
| Pemba | 2013 | Micheweni | 18/3/2013 | MAKANGALE | 40069 | F | 9 | 4 |
| Pemba | 2013 | Micheweni | 18/3/2013 | MAKANGALE | 40070 | F | 11 | 4 |
| Pemba | 2013 | Micheweni | 18/3/2013 | MAKANGALE | 40071 | F | 11 | 3 |
| Pemba | 2013 | Micheweni | 18/3/2013 | MAKANGALE | 40072 | F | 10 | 3 |

| | | | | | | | | |
|---|---|---|---|---|---|---|---|---|
| Pemba | 2013 | Micheweni | 18/3/2013 | MAKANGALE | 40073 | F | 11 | 3 |
| Pemba | 2013 | Micheweni | 18/3/2013 | MAKANGALE | 40074 | F | 11 | 3 |
| Pemba | 2013 | Micheweni | 18/3/2013 | MAKANGALE | 40075 | F | 10 | 3 |
| Pemba | 2013 | Micheweni | 18/3/2013 | MAKANGALE | 40076 | F | 9 | 3 |
| Pemba | 2013 | Micheweni | 18/3/2013 | MAKANGALE | 40077 | F | 10 | 3 |
| Pemba | 2013 | Micheweni | 18/3/2013 | MAKANGALE | 40078 | F | 10 | 3 |
| Pemba | 2013 | Micheweni | 18/3/2013 | MAKANGALE | 40079 | F | 12 | 3 |
| Pemba | 2013 | Micheweni | 18/3/2013 | MAKANGALE | 40080 | F | 10 | 3 |
| Pemba | 2013 | Micheweni | 18/3/2013 | MAKANGALE | 40081 | F | 10 | 3 |
| Pemba | 2013 | Micheweni | 18/3/2013 | MAKANGALE | 40082 | F | 10 | 3 |
| Pemba | 2013 | Micheweni | 18/3/2013 | MAKANGALE | 40083 | F | 11 | 3 |
| Pemba | 2013 | Micheweni | 18/3/2013 | MAKANGALE | 40084 | F | 10 | 3 |
| Pemba | 2013 | Micheweni | 18/3/2013 | MAKANGALE | 40085 | F | 11 | 3 |
| Pemba | 2013 | Micheweni | 18/3/2013 | MAKANGALE | 40086 | F | 10 | 3 |
| Pemba | 2013 | Micheweni | 18/3/2013 | MAKANGALE | 40087 | F | 10 | 3 |
| Pemba | 2013 | Micheweni | 18/3/2013 | MAKANGALE | 40088 | F | 11 | 3 |
| Pemba | 2013 | Micheweni | 18/3/2013 | MAKANGALE | 40089 | F | 11 | 3 |
| Pemba | 2013 | Micheweni | 18/3/2013 | MAKANGALE | 40090 | F | 10 | 3 |
| Pemba | 2013 | Micheweni | 18/3/2013 | MAKANGALE | 40091 | F | 10 | 3 |
| Pemba | 2013 | Micheweni | 18/3/2013 | MAKANGALE | 40092 | F | 10 | 3 |
| Pemba | 2013 | Micheweni | 18/3/2013 | MAKANGALE | 40093 | F | 9 | 3 |
| Pemba | 2013 | Micheweni | 18/3/2013 | MAKANGALE | 40094 | F | 9 | 3 |
| Pemba | 2013 | Micheweni | 18/3/2013 | MAKANGALE | 40095 | F | 9 | 3 |
| Pemba | 2013 | Micheweni | 18/3/2013 | MAKANGALE | 40096 | F | 11 | 3 |
| Pemba | 2013 | Micheweni | 18/3/2013 | MAKANGALE | 40097 | F | 9 | 3 |
| Pemba | 2013 | Micheweni | 18/3/2013 | MAKANGALE | 40098 | F | 11 | 3 |
| Pemba | 2013 | Micheweni | 18/3/2013 | MAKANGALE | 40099 | F | 9 | 3 |
| Pemba | 2013 | Micheweni | 18/3/2013 | MAKANGALE | 40100 | F | 9 | 3 |
| Pemba | 2013 | Micheweni | 18/3/2013 | MAKANGALE | 40101 | F | 10 | 3 |
| Pemba | 2013 | Micheweni | 18/3/2013 | MAKANGALE | 40102 | F | 9 | 3 |
| Pemba | 2013 | Micheweni | 18/3/2013 | MAKANGALE | 40103 | M | 10 | 3 |
| Pemba | 2013 | Micheweni | 18/3/2013 | MAKANGALE | 40104 | M | 11 | 3 |
| Pemba | 2013 | Micheweni | 18/3/2013 | MAKANGALE | 40105 | M | 12 | 3 |
| Pemba | 2013 | Micheweni | 18/3/2013 | MAKANGALE | 40106 | M | 11 | 3 |
| Pemba | 2013 | Micheweni | 18/3/2013 | MAKANGALE | 40107 | M | 9 | 3 |
| Pemba | 2013 | Micheweni | 18/3/2013 | MAKANGALE | 40108 | M | 12 | 3 |
| Pemba | 2013 | Micheweni | 18/3/2013 | MAKANGALE | 40109 | M | 9 | 3 |
| Pemba | 2013 | Micheweni | 18/3/2013 | MAKANGALE | 40110 | M | 12 | 3 |
| Pemba | 2013 | Micheweni | 18/3/2013 | MAKANGALE | 40111 | M | 11 | 3 |
| Pemba | 2013 | Micheweni | 18/3/2013 | MAKANGALE | 40112 | M | 12 | 3 |
| Pemba | 2013 | Micheweni | 18/3/2013 | MAKANGALE | 40113 | M | 12 | 3 |
| Pemba | 2013 | Micheweni | 18/3/2013 | MAKANGALE | 40114 | M | 11 | 3 |
| Pemba | 2013 | Micheweni | 18/3/2013 | MAKANGALE | 40115 | M | 12 | 3 |
| Pemba | 2013 | Micheweni | 18/3/2013 | MAKANGALE | 40116 | M | 12 | 3 |
| Pemba | 2013 | Micheweni | 18/3/2013 | MAKANGALE | 40117 | M | 12 | 3 |
| Pemba | 2013 | Micheweni | 18/3/2013 | MAKANGALE | 40118 | M | 12 | 3 |
| Pemba | 2013 | Micheweni | 18/3/2013 | MAKANGALE | 40119 | M | 11 | 3 |
| Pemba | 2013 | Micheweni | 18/3/2013 | MAKANGALE | 40120 | M | 9 | 3 |
| Pemba | 2013 | Micheweni | 18/3/2013 | MAKANGALE | 40121 | M | 9 | 3 |
| Pemba | 2013 | Micheweni | 18/3/2013 | MAKANGALE | 40122 | M | 12 | 3 |

| | | | | | | | | |
|---|---|---|---|---|---|---|---|---|
| Pemba | 2013 | Micheweni | 18/3/2013 | MAKANGALE | 40123 | M | 12 | 3 |
| Pemba | 2013 | Micheweni | 18/3/2013 | MAKANGALE | 40124 | M | 9 | 3 |
| Pemba | 2013 | Micheweni | 18/3/2013 | MAKANGALE | 40125 | M | 12 | 3 |
| Pemba | 2013 | Micheweni | 18/3/2013 | MAKANGALE | 40126 | M | 12 | 3 |
| Pemba | 2013 | Micheweni | 18/3/2013 | MAKANGALE | 40127 | M | 10 | 3 |
| Pemba | 2013 | Micheweni | 18/3/2013 | MAKANGALE | 40128 | M | 11 | 3 |
| Pemba | 2013 | Micheweni | 18/3/2013 | MAKANGALE | 40129 | M | 10 | 3 |
| Pemba | 2013 | Micheweni | 18/3/2013 | MAKANGALE | 40130 | M | 10 | 3 |
| Pemba | 2013 | Micheweni | 13/3/2013 | KINOWE | 44001 | F | 12 | 4 |
| Pemba | 2013 | Micheweni | 13/3/2013 | KINOWE | 44002 | F | 10 | 4 |
| Pemba | 2013 | Micheweni | 13/3/2013 | KINOWE | 44003 | F | 12 | 4 |
| Pemba | 2013 | Micheweni | 13/3/2013 | KINOWE | 44004 | F | 10 | 4 |
| Pemba | 2013 | Micheweni | 13/3/2013 | KINOWE | 44005 | F | 12 | 4 |
| Pemba | 2013 | Micheweni | 13/3/2013 | KINOWE | 44006 | F | 12 | 4 |
| Pemba | 2013 | Micheweni | 13/3/2013 | KINOWE | 44007 | F | 12 | 4 |
| Pemba | 2013 | Micheweni | 13/3/2013 | KINOWE | 44008 | F | 9 | 4 |
| Pemba | 2013 | Micheweni | 13/3/2013 | KINOWE | 44009 | F | 12 | 4 |
| Pemba | 2013 | Micheweni | 13/3/2013 | KINOWE | 44010 | F | 11 | 4 |
| Pemba | 2013 | Micheweni | 13/3/2013 | KINOWE | 44011 | F | 11 | 4 |
| Pemba | 2013 | Micheweni | 13/3/2013 | KINOWE | 44012 | F | 12 | 4 |
| Pemba | 2013 | Micheweni | 13/3/2013 | KINOWE | 44013 | F | 11 | 4 |
| Pemba | 2013 | Micheweni | 13/3/2013 | KINOWE | 44014 | F | 11 | 4 |
| Pemba | 2013 | Micheweni | 13/3/2013 | KINOWE | 44015 | F | 10 | 4 |
| Pemba | 2013 | Micheweni | 13/3/2013 | KINOWE | 44016 | F | 12 | 4 |
| Pemba | 2013 | Micheweni | 13/3/2013 | KINOWE | 44017 | F | 11 | 4 |
| Pemba | 2013 | Micheweni | 13/3/2013 | KINOWE | 44018 | F | 10 | 4 |
| Pemba | 2013 | Micheweni | 13/3/2013 | KINOWE | 44019 | F | 11 | 4 |
| Pemba | 2013 | Micheweni | 13/3/2013 | KINOWE | 44020 | F | 10 | 4 |
| Pemba | 2013 | Micheweni | 13/3/2013 | KINOWE | 44021 | F | 12 | 4 |
| Pemba | 2013 | Micheweni | 13/3/2013 | KINOWE | 44022 | F | 12 | 4 |
| Pemba | 2013 | Micheweni | 13/3/2013 | KINOWE | 44023 | F | 10 | 4 |
| Pemba | 2013 | Micheweni | 13/3/2013 | KINOWE | 44024 | F | 10 | 4 |
| Pemba | 2013 | Micheweni | 13/3/2013 | KINOWE | 44025 | F | 12 | 4 |
| Pemba | 2013 | Micheweni | 13/3/2013 | KINOWE | 44026 | F | 11 | 4 |
| Pemba | 2013 | Micheweni | 13/3/2013 | KINOWE | 44027 | F | 10 | 4 |
| Pemba | 2013 | Micheweni | 13/3/2013 | KINOWE | 44028 | F | 12 | 4 |
| Pemba | 2013 | Micheweni | 13/3/2013 | KINOWE | 44029 | F | 11 | 4 |
| Pemba | 2013 | Micheweni | 13/3/2013 | KINOWE | 44030 | F | 10 | 4 |
| Pemba | 2013 | Micheweni | 13/3/2013 | KINOWE | 44031 | F | 10 | 4 |
| Pemba | 2013 | Micheweni | 13/3/2013 | KINOWE | 44032 | F | 11 | 4 |
| Pemba | 2013 | Micheweni | 13/3/2013 | KINOWE | 44033 | F | 10 | 4 |
| Pemba | 2013 | Micheweni | 13/3/2013 | KINOWE | 44034 | F | 10 | 4 |
| Pemba | 2013 | Micheweni | 13/3/2013 | KINOWE | 44035 | F | 12 | 4 |
| Pemba | 2013 | Micheweni | 13/3/2013 | KINOWE | 44036 | F | 11 | 4 |
| Pemba | 2013 | Micheweni | 13/3/2013 | KINOWE | 44037 | F | 10 | 4 |
| Pemba | 2013 | Micheweni | 13/3/2013 | KINOWE | 44038 | F | 11 | 4 |
| Pemba | 2013 | Micheweni | 13/3/2013 | KINOWE | 44039 | F | 10 | 4 |
| Pemba | 2013 | Micheweni | 13/3/2013 | KINOWE | 44040 | F | 11 | 4 |
| Pemba | 2013 | Micheweni | 13/3/2013 | KINOWE | 44041 | F | 12 | 4 |
| Pemba | 2013 | Micheweni | 13/3/2013 | KINOWE | 44042 | F | 11 | 4 |

| | | | | | | | | |
|---|---|---|---|---|---|---|---|---|
| Pemba | 2013 | Micheweni | 13/3/2013 | KINOWE | 44043 | F | 12 | 4 |
| Pemba | 2013 | Micheweni | 13/3/2013 | KINOWE | 44044 | F | 11 | 4 |
| Pemba | 2013 | Micheweni | 13/3/2013 | KINOWE | 44045 | F | 10 | 4 |
| Pemba | 2013 | Micheweni | 13/3/2013 | KINOWE | 44046 | F | 10 | 4 |
| Pemba | 2013 | Micheweni | 13/3/2013 | KINOWE | 44047 | F | 10 | 4 |
| Pemba | 2013 | Micheweni | 13/3/2013 | KINOWE | 44048 | F | 10 | 4 |
| Pemba | 2013 | Micheweni | 13/3/2013 | KINOWE | 44049 | F | 12 | 4 |
| Pemba | 2013 | Micheweni | 13/3/2013 | KINOWE | 44050 | F | 11 | 4 |
| Pemba | 2013 | Micheweni | 13/3/2013 | KINOWE | 44051 | M | 12 | 4 |
| Pemba | 2013 | Micheweni | 13/3/2013 | KINOWE | 44052 | M | 12 | 4 |
| Pemba | 2013 | Micheweni | 13/3/2013 | KINOWE | 44053 | M | 11 | 4 |
| Pemba | 2013 | Micheweni | 13/3/2013 | KINOWE | 44054 | M | 12 | 4 |
| Pemba | 2013 | Micheweni | 13/3/2013 | KINOWE | 44055 | M | 12 | 4 |
| Pemba | 2013 | Micheweni | 13/3/2013 | KINOWE | 44056 | M | 12 | 4 |
| Pemba | 2013 | Micheweni | 13/3/2013 | KINOWE | 44057 | M | 12 | 4 |
| Pemba | 2013 | Micheweni | 13/3/2013 | KINOWE | 44058 | M | 12 | 4 |
| Pemba | 2013 | Micheweni | 13/3/2013 | KINOWE | 44059 | M | 12 | 4 |
| Pemba | 2013 | Micheweni | 13/3/2013 | KINOWE | 44060 | M | 12 | 4 |
| Pemba | 2013 | Micheweni | 13/3/2013 | KINOWE | 44061 | M | 12 | 4 |
| Pemba | 2013 | Micheweni | 13/3/2013 | KINOWE | 44062 | M | 11 | 4 |
| Pemba | 2013 | Micheweni | 13/3/2013 | KINOWE | 44063 | M | 11 | 4 |
| Pemba | 2013 | Micheweni | 13/3/2013 | KINOWE | 44064 | M | 9 | 4 |
| Pemba | 2013 | Micheweni | 13/3/2013 | KINOWE | 44065 | M | 12 | 4 |
| Pemba | 2013 | Micheweni | 13/3/2013 | KINOWE | 44066 | M | 11 | 4 |
| Pemba | 2013 | Micheweni | 13/3/2013 | KINOWE | 44067 | M | 12 | 4 |
| Pemba | 2013 | Micheweni | 13/3/2013 | KINOWE | 44068 | M | 12 | 4 |
| Pemba | 2013 | Micheweni | 13/3/2013 | KINOWE | 44069 | M | 12 | 4 |
| Pemba | 2013 | Micheweni | 13/3/2013 | KINOWE | 44070 | M | 10 | 4 |
| Pemba | 2013 | Micheweni | 13/3/2013 | KINOWE | 44071 | M | 12 | 4 |
| Pemba | 2013 | Micheweni | 13/3/2013 | KINOWE | 44072 | M | 10 | 4 |
| Pemba | 2013 | Micheweni | 13/3/2013 | KINOWE | 44073 | M | 11 | 4 |
| Pemba | 2013 | Micheweni | 13/3/2013 | KINOWE | 44074 | M | 12 | 4 |
| Pemba | 2013 | Micheweni | 13/3/2013 | KINOWE | 44075 | M | 12 | 4 |
| Pemba | 2013 | Micheweni | 13/3/2013 | KINOWE | 44076 | M | 12 | 4 |
| Pemba | 2013 | Micheweni | 13/3/2013 | KINOWE | 44077 | M | 12 | 4 |
| Pemba | 2013 | Micheweni | 13/3/2013 | KINOWE | 44078 | M | 10 | 4 |
| Pemba | 2013 | Micheweni | 13/3/2013 | KINOWE | 44079 | M | 11 | 4 |
| Pemba | 2013 | Micheweni | 13/3/2013 | KINOWE | 44080 | M | 11 | 4 |
| Pemba | 2013 | Micheweni | 13/3/2013 | KINOWE | 44081 | M | 10 | 4 |
| Pemba | 2013 | Micheweni | 13/3/2013 | KINOWE | 44082 | M | 12 | 4 |
| Pemba | 2013 | Micheweni | 13/3/2013 | KINOWE | 44083 | M | 10 | 4 |
| Pemba | 2013 | Micheweni | 13/3/2013 | KINOWE | 44084 | M | 11 | 4 |
| Pemba | 2013 | Micheweni | 13/3/2013 | KINOWE | 44085 | M | 11 | 4 |
| Pemba | 2013 | Micheweni | 13/3/2013 | KINOWE | 44086 | M | 11 | 4 |
| Pemba | 2013 | Micheweni | 13/3/2013 | KINOWE | 44087 | M | 11 | 4 |
| Pemba | 2013 | Micheweni | 13/3/2013 | KINOWE | 44088 | M | 11 | 4 |
| Pemba | 2013 | Micheweni | 13/3/2013 | KINOWE | 44089 | M | 12 | 4 |
| Pemba | 2013 | Micheweni | 13/3/2013 | KINOWE | 44090 | M | 12 | 4 |
| Pemba | 2013 | Micheweni | 13/3/2013 | KINOWE | 44091 | M | 12 | 4 |
| Pemba | 2013 | Micheweni | 13/3/2013 | KINOWE | 44092 | M | 11 | 4 |

| | | | | | | | | |
|---|---|---|---|---|---|---|---|---|
| Pemba | 2013 | Micheweni | 13/3/2013 | KINOWE | 44093 | M | 10 | 4 |
| Pemba | 2013 | Micheweni | 13/3/2013 | KINOWE | 44094 | M | 12 | 4 |
| Pemba | 2013 | Micheweni | 13/3/2013 | KINOWE | 44095 | M | 12 | 4 |
| Pemba | 2013 | Micheweni | 13/3/2013 | KINOWE | 44096 | M | 12 | 4 |
| Pemba | 2013 | Micheweni | 13/3/2013 | KINOWE | 44097 | M | 11 | 4 |
| Pemba | 2013 | Micheweni | 13/3/2013 | KINOWE | 44098 | M | 11 | 4 |
| Pemba | 2013 | Micheweni | 13/3/2013 | KINOWE | 44099 | M | 12 | 4 |
| Pemba | 2013 | Micheweni | 13/3/2013 | KINOWE | 44100 | M | 11 | 4 |
| Pemba | 2013 | Micheweni | 13/3/2013 | KINOWE | 44101 | F | 10 | 3 |
| Pemba | 2013 | Micheweni | 13/3/2013 | KINOWE | 44102 | F | 9 | 3 |
| Pemba | 2013 | Micheweni | 13/3/2013 | KINOWE | 44103 | F | 10 | 3 |
| Pemba | 2013 | Micheweni | 13/3/2013 | KINOWE | 44104 | F | 12 | 3 |
| Pemba | 2013 | Micheweni | 13/3/2013 | KINOWE | 44105 | F | 11 | 3 |
| Pemba | 2013 | Micheweni | 13/3/2013 | KINOWE | 44106 | F | 12 | 3 |
| Pemba | 2013 | Micheweni | 13/3/2013 | KINOWE | 44107 | F | 10 | 3 |
| Pemba | 2013 | Micheweni | 13/3/2013 | KINOWE | 44108 | F | 11 | 3 |
| Pemba | 2013 | Micheweni | 13/3/2013 | KINOWE | 44109 | F | 12 | 3 |
| Pemba | 2013 | Micheweni | 13/3/2013 | KINOWE | 44110 | F | 10 | 3 |
| Pemba | 2013 | Micheweni | 13/3/2013 | KINOWE | 44111 | F | 10 | 3 |
| Pemba | 2013 | Micheweni | 13/3/2013 | KINOWE | 44112 | F | 12 | 3 |
| Pemba | 2013 | Micheweni | 13/3/2013 | KINOWE | 44113 | F | 10 | 3 |
| Pemba | 2013 | Micheweni | 13/3/2013 | KINOWE | 44114 | F | 10 | 3 |
| Pemba | 2013 | Micheweni | 13/3/2013 | KINOWE | 44115 | F | 10 | 3 |
| Pemba | 2013 | Micheweni | 13/3/2013 | KINOWE | 44116 | M | 9 | 3 |
| Pemba | 2013 | Micheweni | 13/3/2013 | KINOWE | 44117 | M | 12 | 3 |
| Pemba | 2013 | Micheweni | 13/3/2013 | KINOWE | 44118 | M | 9 | 3 |
| Pemba | 2013 | Micheweni | 13/3/2013 | KINOWE | 44119 | M | 9 | 3 |
| Pemba | 2013 | Micheweni | 13/3/2013 | KINOWE | 44120 | M | 10 | 3 |
| Pemba | 2013 | Micheweni | 13/3/2013 | KINOWE | 44121 | M | 12 | 3 |
| Pemba | 2013 | Micheweni | 13/3/2013 | KINOWE | 44122 | M | 10 | 3 |
| Pemba | 2013 | Micheweni | 13/3/2013 | KINOWE | 44123 | M | 10 | 3 |
| Pemba | 2013 | Micheweni | 13/3/2013 | KINOWE | 44124 | M | 12 | 3 |
| Pemba | 2013 | Micheweni | 13/3/2013 | KINOWE | 44125 | M | 10 | 3 |
| Pemba | 2013 | Micheweni | 13/3/2013 | KINOWE | 44126 | M | 10 | 3 |
| Pemba | 2013 | Micheweni | 13/3/2013 | KINOWE | 44127 | M | 12 | 3 |
| Pemba | 2013 | Micheweni | 13/3/2013 | KINOWE | 44128 | M | 11 | 3 |
| Pemba | 2013 | Micheweni | 13/3/2013 | KINOWE | 44129 | M | 9 | 3 |
| Pemba | 2013 | Micheweni | 13/3/2013 | KINOWE | 44130 | M | 12 | 3 |
| Pemba | 2013 | Micheweni | 14/3/2013 | KONDE A | 45001 | M | 11 | 4 |
| Pemba | 2013 | Micheweni | 14/3/2013 | KONDE A | 45002 | F | 11 | 4 |
| Pemba | 2013 | Micheweni | 14/3/2013 | KONDE A | 45003 | F | 10 | 4 |
| Pemba | 2013 | Micheweni | 14/3/2013 | KONDE A | 45004 | F | 10 | 4 |
| Pemba | 2013 | Micheweni | 14/3/2013 | KONDE A | 45005 | F | 12 | 4 |
| Pemba | 2013 | Micheweni | 14/3/2013 | KONDE A | 45006 | F | 11 | 4 |
| Pemba | 2013 | Micheweni | 14/3/2013 | KONDE A | 45007 | F | 10 | 4 |
| Pemba | 2013 | Micheweni | 14/3/2013 | KONDE A | 45008 | F | 11 | 4 |
| Pemba | 2013 | Micheweni | 14/3/2013 | KONDE A | 45009 | F | 10 | 4 |
| Pemba | 2013 | Micheweni | 14/3/2013 | KONDE A | 45010 | F | 10 | 4 |
| Pemba | 2013 | Micheweni | 14/3/2013 | KONDE A | 45011 | F | 10 | 4 |
| Pemba | 2013 | Micheweni | 14/3/2013 | KONDE A | 45012 | F | 11 | 4 |

| | | | | | | | | |
|---|---|---|---|---|---|---|---|---|
| Pemba | 2013 | Micheweni | 14/3/2013 | KONDE A | 45013 | F | 10 | 4 |
| Pemba | 2013 | Micheweni | 14/3/2013 | KONDE A | 45014 | F | 12 | 4 |
| Pemba | 2013 | Micheweni | 14/3/2013 | KONDE A | 45015 | F | 10 | 4 |
| Pemba | 2013 | Micheweni | 14/3/2013 | KONDE A | 45016 | F | 10 | 4 |
| Pemba | 2013 | Micheweni | 14/3/2013 | KONDE A | 45017 | F | 10 | 4 |
| Pemba | 2013 | Micheweni | 14/3/2013 | KONDE A | 45018 | F | 11 | 4 |
| Pemba | 2013 | Micheweni | 14/3/2013 | KONDE A | 45019 | F | 12 | 4 |
| Pemba | 2013 | Micheweni | 14/3/2013 | KONDE A | 45020 | F | 11 | 4 |
| Pemba | 2013 | Micheweni | 14/3/2013 | KONDE A | 45021 | F | 9 | 4 |
| Pemba | 2013 | Micheweni | 14/3/2013 | KONDE A | 45022 | F | 10 | 4 |
| Pemba | 2013 | Micheweni | 14/3/2013 | KONDE A | 45023 | F | 10 | 4 |
| Pemba | 2013 | Micheweni | 14/3/2013 | KONDE A | 45024 | F | 11 | 4 |
| Pemba | 2013 | Micheweni | 14/3/2013 | KONDE A | 45025 | M | 10 | 4 |
| Pemba | 2013 | Micheweni | 14/3/2013 | KONDE A | 45026 | M | 11 | 4 |
| Pemba | 2013 | Micheweni | 14/3/2013 | KONDE A | 45027 | M | 11 | 4 |
| Pemba | 2013 | Micheweni | 14/3/2013 | KONDE A | 45028 | M | 10 | 4 |
| Pemba | 2013 | Micheweni | 14/3/2013 | KONDE A | 45029 | M | 11 | 4 |
| Pemba | 2013 | Micheweni | 14/3/2013 | KONDE A | 45030 | M | 11 | 4 |
| Pemba | 2013 | Micheweni | 14/3/2013 | KONDE A | 45031 | M | 12 | 4 |
| Pemba | 2013 | Micheweni | 14/3/2013 | KONDE A | 45032 | M | 10 | 4 |
| Pemba | 2013 | Micheweni | 14/3/2013 | KONDE A | 45033 | M | 11 | 4 |
| Pemba | 2013 | Micheweni | 14/3/2013 | KONDE A | 45034 | M | 11 | 4 |
| Pemba | 2013 | Micheweni | 14/3/2013 | KONDE A | 45035 | M | 11 | 4 |
| Pemba | 2013 | Micheweni | 14/3/2013 | KONDE A | 45036 | M | 10 | 4 |
| Pemba | 2013 | Micheweni | 14/3/2013 | KONDE A | 45037 | M | 12 | 4 |
| Pemba | 2013 | Micheweni | 14/3/2013 | KONDE A | 45038 | M | 10 | 4 |
| Pemba | 2013 | Micheweni | 14/3/2013 | KONDE A | 45039 | M | 11 | 4 |
| Pemba | 2013 | Micheweni | 14/3/2013 | KONDE A | 45040 | M | 11 | 4 |
| Pemba | 2013 | Micheweni | 14/3/2013 | KONDE A | 45041 | M | 11 | 4 |
| Pemba | 2013 | Micheweni | 14/3/2013 | KONDE A | 45042 | M | 12 | 4 |
| Pemba | 2013 | Micheweni | 14/3/2013 | KONDE A | 45043 | M | 10 | 4 |
| Pemba | 2013 | Micheweni | 14/3/2013 | KONDE A | 45044 | M | 11 | 4 |
| Pemba | 2013 | Micheweni | 14/3/2013 | KONDE A | 45045 | M | 12 | 4 |
| Pemba | 2013 | Micheweni | 14/3/2013 | KONDE A | 45046 | M | 10 | 4 |
| Pemba | 2013 | Micheweni | 14/3/2013 | KONDE A | 45047 | M | 11 | 4 |
| Pemba | 2013 | Micheweni | 14/3/2013 | KONDE A | 45048 | M | 11 | 4 |
| Pemba | 2013 | Micheweni | 14/3/2013 | KONDE A | 45049 | M | 11 | 4 |
| Pemba | 2013 | Micheweni | 14/3/2013 | KONDE A | 45050 | M | 10 | 4 |
| Pemba | 2013 | Micheweni | 14/3/2013 | KONDE A | 45051 | M | 10 | 4 |
| Pemba | 2013 | Micheweni | 14/3/2013 | KONDE A | 45052 | M | 11 | 4 |
| Pemba | 2013 | Micheweni | 14/3/2013 | KONDE A | 45053 | M | 11 | 4 |
| Pemba | 2013 | Micheweni | 14/3/2013 | KONDE A | 45054 | M | 11 | 4 |
| Pemba | 2013 | Micheweni | 14/3/2013 | KONDE A | 45055 | M | 11 | 4 |
| Pemba | 2013 | Micheweni | 14/3/2013 | KONDE A | 45056 | M | 11 | 4 |
| Pemba | 2013 | Micheweni | 14/3/2013 | KONDE A | 45057 | M | 11 | 4 |
| Pemba | 2013 | Micheweni | 14/3/2013 | KONDE A | 45058 | M | 11 | 4 |
| Pemba | 2013 | Micheweni | 14/3/2013 | KONDE A | 45059 | M | 11 | 4 |
| Pemba | 2013 | Micheweni | 14/3/2013 | KONDE A | 45060 | M | 10 | 4 |
| Pemba | 2013 | Micheweni | 14/3/2013 | KONDE A | 45061 | M | 11 | 4 |
| Pemba | 2013 | Micheweni | 14/3/2013 | KONDE A | 45062 | M | 11 | 4 |

| | | | | | | | | |
|---|---|---|---|---|---|---|---|---|
| Pemba | 2013 | Micheweni | 14/3/2013 | KONDE A | 45063 | M | 11 | 4 |
| Pemba | 2013 | Micheweni | 14/3/2013 | KONDE A | 45064 | M | 11 | 4 |
| Pemba | 2013 | Micheweni | 14/3/2013 | KONDE A | 45065 | M | 9 | 4 |
| Pemba | 2013 | Micheweni | 14/3/2013 | KONDE A | 45066 | M | 10 | 4 |
| Pemba | 2013 | Micheweni | 14/3/2013 | KONDE A | 45067 | M | 10 | 4 |
| Pemba | 2013 | Micheweni | 14/3/2013 | KONDE A | 45068 | M | 10 | 4 |
| Pemba | 2013 | Micheweni | 14/3/2013 | KONDE A | 45069 | M | 10 | 4 |
| Pemba | 2013 | Micheweni | 14/3/2013 | KONDE A | 45070 | F | 11 | 4 |
| Pemba | 2013 | Micheweni | 14/3/2013 | KONDE A | 45071 | F | 11 | 4 |
| Pemba | 2013 | Micheweni | 14/3/2013 | KONDE A | 45072 | F | 10 | 4 |
| Pemba | 2013 | Micheweni | 14/3/2013 | KONDE A | 45073 | F | 10 | 4 |
| Pemba | 2013 | Micheweni | 14/3/2013 | KONDE A | 45074 | F | 9 | 4 |
| Pemba | 2013 | Micheweni | 14/3/2013 | KONDE A | 45075 | F | 10 | 4 |
| Pemba | 2013 | Micheweni | 14/3/2013 | KONDE A | 45076 | F | 12 | 4 |
| Pemba | 2013 | Micheweni | 14/3/2013 | KONDE A | 45077 | F | 11 | 4 |
| Pemba | 2013 | Micheweni | 14/3/2013 | KONDE A | 45078 | F | 10 | 4 |
| Pemba | 2013 | Micheweni | 14/3/2013 | KONDE A | 45079 | F | 11 | 4 |
| Pemba | 2013 | Micheweni | 14/3/2013 | KONDE A | 45080 | F | 9 | 4 |
| Pemba | 2013 | Micheweni | 14/3/2013 | KONDE A | 45081 | F | 10 | 4 |
| Pemba | 2013 | Micheweni | 14/3/2013 | KONDE A | 45082 | F | 11 | 4 |
| Pemba | 2013 | Micheweni | 14/3/2013 | KONDE A | 45083 | F | 12 | 4 |
| Pemba | 2013 | Micheweni | 14/3/2013 | KONDE A | 45084 | F | 10 | 4 |
| Pemba | 2013 | Micheweni | 14/3/2013 | KONDE A | 45085 | F | 10 | 4 |
| Pemba | 2013 | Micheweni | 14/3/2013 | KONDE A | 45086 | F | 10 | 4 |
| Pemba | 2013 | Micheweni | 14/3/2013 | KONDE A | 45087 | F | 11 | 4 |
| Pemba | 2013 | Micheweni | 14/3/2013 | KONDE A | 45088 | F | 10 | 4 |
| Pemba | 2013 | Micheweni | 14/3/2013 | KONDE A | 45089 | F | 10 | 4 |
| Pemba | 2013 | Micheweni | 14/3/2013 | KONDE A | 45090 | F | 11 | 4 |
| Pemba | 2013 | Micheweni | 14/3/2013 | KONDE A | 45091 | F | 10 | 4 |
| Pemba | 2013 | Micheweni | 14/3/2013 | KONDE A | 45092 | F | 10 | 4 |
| Pemba | 2013 | Micheweni | 14/3/2013 | KONDE A | 45093 | F | 11 | 4 |
| Pemba | 2013 | Micheweni | 14/3/2013 | KONDE A | 45094 | F | 11 | 4 |
| Pemba | 2013 | Micheweni | 14/3/2013 | KONDE A | 45095 | F | 10 | 4 |
| Pemba | 2013 | Micheweni | 14/3/2013 | KONDE A | 45096 | F | 10 | 4 |
| Pemba | 2013 | Micheweni | 14/3/2013 | KONDE A | 45097 | F | 11 | 4 |
| Pemba | 2013 | Micheweni | 14/3/2013 | KONDE A | 45098 | F | 10 | 4 |
| Pemba | 2013 | Micheweni | 14/3/2013 | KONDE A | 45099 | F | 10 | 4 |
| Pemba | 2013 | Micheweni | 14/3/2013 | KONDE A | 45100 | F | 11 | 4 |
| Pemba | 2013 | Micheweni | 14/3/2013 | KONDE A | 45101 | F | 11 | 3 |
| Pemba | 2013 | Micheweni | 14/3/2013 | KONDE A | 45102 | F | 11 | 3 |
| Pemba | 2013 | Micheweni | 14/3/2013 | KONDE A | 45103 | F | 12 | 3 |
| Pemba | 2013 | Micheweni | 14/3/2013 | KONDE A | 45104 | F | 11 | 3 |
| Pemba | 2013 | Micheweni | 14/3/2013 | KONDE A | 45105 | F | 9 | 3 |
| Pemba | 2013 | Micheweni | 14/3/2013 | KONDE A | 45106 | F | 11 | 3 |
| Pemba | 2013 | Micheweni | 14/3/2013 | KONDE A | 45107 | F | 10 | 3 |
| Pemba | 2013 | Micheweni | 14/3/2013 | KONDE A | 45108 | F | 12 | 3 |
| Pemba | 2013 | Micheweni | 14/3/2013 | KONDE A | 45109 | F | 10 | 3 |
| Pemba | 2013 | Micheweni | 14/3/2013 | KONDE A | 45110 | F | 9 | 3 |
| Pemba | 2013 | Micheweni | 14/3/2013 | KONDE A | 45111 | F | 11 | 3 |
| Pemba | 2013 | Micheweni | 14/3/2013 | KONDE A | 45112 | F | 9 | 3 |

| | | | | | | | | |
|---|---|---|---|---|---|---|---|---|
| Pemba | 2013 | Micheweni | 14/3/2013 | KONDE A | 45113 | F | 11 | 3 |
| Pemba | 2013 | Micheweni | 14/3/2013 | KONDE A | 45114 | F | 11 | 3 |
| Pemba | 2013 | Micheweni | 14/3/2013 | KONDE A | 45115 | F | 11 | 3 |
| Pemba | 2013 | Micheweni | 14/3/2013 | KONDE A | 45116 | M | 11 | 3 |
| Pemba | 2013 | Micheweni | 14/3/2013 | KONDE A | 45117 | M | 10 | 3 |
| Pemba | 2013 | Micheweni | 14/3/2013 | KONDE A | 45118 | M | 9 | 3 |
| Pemba | 2013 | Micheweni | 14/3/2013 | KONDE A | 45119 | M | 10 | 3 |
| Pemba | 2013 | Micheweni | 14/3/2013 | KONDE A | 45120 | M | 11 | 3 |
| Pemba | 2013 | Micheweni | 14/3/2013 | KONDE A | 45121 | M | 11 | 3 |
| Pemba | 2013 | Micheweni | 14/3/2013 | KONDE A | 45122 | M | 9 | 3 |
| Pemba | 2013 | Micheweni | 14/3/2013 | KONDE A | 45123 | M | 12 | 3 |
| Pemba | 2013 | Micheweni | 14/3/2013 | KONDE A | 45124 | M | 11 | 3 |
| Pemba | 2013 | Micheweni | 14/3/2013 | KONDE A | 45125 | M | 10 | 3 |
| Pemba | 2013 | Micheweni | 14/3/2013 | KONDE A | 45126 | M | 10 | 3 |
| Pemba | 2013 | Micheweni | 14/3/2013 | KONDE A | 45127 | M | 10 | 3 |
| Pemba | 2013 | Micheweni | 14/3/2013 | KONDE A | 45128 | M | 10 | 3 |
| Pemba | 2013 | Micheweni | 14/3/2013 | KONDE A | 45129 | M | 10 | 3 |
| Pemba | 2013 | Micheweni | 14/3/2013 | KONDE A | 45130 | M | 11 | 3 |

| Consent given | Haemastix_haem | Haem_Date | UF_Sh | UF_Date | QC_UF_Sh |
|---|---|---|---|---|---|
| 1 | 0 | 17/4/2013 | 0 | 17/4/2013 | 0 |
| 1 | 0 | 17/4/2013 | 0 | 17/4/2013 | 0 |
| 1 | 0 | 17/4/2013 | 0 | 17/4/2013 | 0 |
| 1 | 0 | 17/4/2013 | 0 | 17/4/2013 | 0 |
| 1 | 0 | 17/4/2013 | 0 | 17/4/2013 | 0 |
| 1 | 0 | 17/4/2013 | 0 | 17/4/2013 | 0 |
| 1 | 0 | 17/4/2013 | 0 | 17/4/2013 | 0 |
| 1 | 0 | 17/4/2013 | 0 | 17/4/2013 | 0 |
| 1 | 0 | 17/4/2013 | 0 | 17/4/2013 | 0 |
| 1 | 0 | 17/4/2013 | 0 | 17/4/2013 | 0 |
| 1 | 0 | 17/4/2013 | 0 | 17/4/2013 | 0 |
| 1 | 0 | 17/4/2013 | 0 | 17/4/2013 | 0 |
| 1 | 0 | 17/4/2013 | 0 | 17/4/2013 | 0 |
| 1 | 0 | 17/4/2013 | 0 | 17/4/2013 | 0 |
| 1 | 0 | 17/4/2013 | 0 | 17/4/2013 | 0 |
| 1 | 0 | 17/4/2013 | 0 | 17/4/2013 | 0 |
| 1 | 0 | 17/4/2013 | 0 | 17/4/2013 | 0 |
| 1 | 0 | 17/4/2013 | 0 | 17/4/2013 | 0 |
| 1 | 0 | 17/4/2013 | 0 | 17/4/2013 | 0 |
| 1 | 0 | 17/4/2013 | 0 | 17/4/2013 | 0 |
| 1 | 0 | 17/4/2013 | 0 | 17/4/2013 | 0 |
| 1 | 0 | 17/4/2013 | 0 | 17/4/2013 | 0 |
| 1 | 0 | 17/4/2013 | 0 | 17/4/2013 | 0 |
| 1 | 0 | 17/4/2013 | 0 | 17/4/2013 | 0 |
| 1 | 0 | 17/4/2013 | 0 | 17/4/2013 | 0 |
| 1 | 0 | 17/4/2013 | 0 | 17/4/2013 | 0 |
| 1 | 0 | 17/4/2013 | 0 | 17/4/2013 | 0 |
| 1 | 0 | 17/4/2013 | 0 | 17/4/2013 | 0 |
| 1 | 0 | 17/4/2013 | 0 | 17/4/2013 | 0 |
| 1 | 1 | 17/4/2013 | 0 | 17/4/2013 | MISSING |
| 1 | 0 | 17/4/2013 | 0 | 17/4/2013 | 0 |
| 1 | 0 | 17/4/2013 | 0 | 17/4/2013 | 0 |
| 1 | 0 | 17/4/2013 | 0 | 17/4/2013 | 0 |
| 1 | 0 | 17/4/2013 | 0 | 17/4/2013 | 0 |
| 1 | 0 | 17/4/2013 | 0 | 17/4/2013 | 0 |
| 1 | 0 | 17/4/2013 | 0 | 17/4/2013 | 0 |
| 1 | | 17/4/2013 | | | |
| 1 | 0 | 17/4/2013 | 0 | 17/4/2013 | 0 |
| 1 | 0 | 17/4/2013 | 0 | 17/4/2013 | 0 |
| 1 | 0 | 17/4/2013 | 0 | 17/4/2013 | 0 |
| 1 | 0 | 17/4/2013 | 0 | 17/4/2013 | 0 |
| 1 | 0 | 17/4/2013 | 0 | 17/4/2013 | 0 |
| 1 | 0 | 17/4/2013 | 0 | 17/4/2013 | 0 |
| 1 | 0 | 17/4/2013 | 0 | 17/4/2013 | 0 |
| 1 | 0 | 17/4/2013 | 0 | 17/4/2013 | 0 |
| 1 | 0 | 17/4/2013 | 0 | 17/4/2013 | 0 |
| 0 | | 17/4/2013 | | | |
| 1 | 0 | 17/4/2013 | 0 | 17/4/2013 | 0 |
| 1 | 1 | 17/4/2013 | 0 | 17/4/2013 | 0 |

| | | | | | |
|---|---|---|---|---|---|
| 1 | | 17/4/2013 | | | |
| 1 | 0 | 17/4/2013 | 0 | 17/4/2013 | 0 |
| 1 | 0 | 17/4/2013 | 0 | 17/4/2013 | 0 |
| 0 | | 17/4/2013 | | | |
| 0 | | 17/4/2013 | | | |
| 0 | | 17/4/2013 | | | |
| 0 | | 17/4/2013 | | | |
| 1 | 0 | 17/4/2013 | 0 | 17/4/2013 | 0 |
| 1 | 0 | 17/4/2013 | 0 | 17/4/2013 | 0 |
| 0 | | 17/4/2013 | | | |
| 0 | | 17/4/2013 | | | |
| 0 | | 17/4/2013 | | | |
| 1 | 0 | 17/4/2013 | 0 | 17/4/2013 | 0 |
| 1 | 0 | 17/4/2013 | 0 | 17/4/2013 | 0 |
| 1 | 0 | 17/4/2013 | 0 | 17/4/2013 | 0 |
| 1 | 0 | 17/4/2013 | 0 | 17/4/2013 | 0 |
| 1 | 0 | 17/4/2013 | 0 | 17/4/2013 | 0 |
| 1 | 0 | 17/4/2013 | 0 | 17/4/2013 | 0 |
| 1 | 0 | 17/4/2013 | 0 | 17/4/2013 | 0 |
| 1 | 0 | 17/4/2013 | 0 | 17/4/2013 | 0 |
| 1 | 0 | 17/4/2013 | 0 | 17/4/2013 | 0 |
| 1 | 0 | 17/4/2013 | 0 | 17/4/2013 | 0 |
| 1 | 0 | 17/4/2013 | 0 | 17/4/2013 | 0 |
| 1 | 0 | 17/4/2013 | 0 | 17/4/2013 | 0 |
| 1 | 0 | 17/4/2013 | 0 | 17/4/2013 | 0 |
| 1 | 0 | 17/4/2013 | 0 | 17/4/2013 | 0 |
| 1 | 0 | 17/4/2013 | 0 | 17/4/2013 | 0 |
| 1 | 0 | 17/4/2013 | 0 | 17/4/2013 | 0 |
| 1 | 0 | 17/4/2013 | 0 | 17/4/2013 | 0 |
| 1 | 0 | 17/4/2013 | 0 | 17/4/2013 | 0 |
| 1 | 0 | 17/4/2013 | 0 | 17/4/2013 | 0 |
| 1 | 0 | 17/4/2013 | 0 | 17/4/2013 | 0 |
| 1 | 0 | 17/4/2013 | 0 | 17/4/2013 | 0 |
| 1 | 0 | 17/4/2013 | 0 | 17/4/2013 | 0 |
| 1 | 0 | 17/4/2013 | 0 | 17/4/2013 | 0 |
| 1 | 0 | 17/4/2013 | 0 | 17/4/2013 | 0 |
| 1 | 0 | 17/4/2013 | 0 | 17/4/2013 | 0 |
| 1 | 0 | 17/4/2013 | 0 | 17/4/2013 | 0 |
| 1 | 0 | 17/4/2013 | 0 | 17/4/2013 | 0 |
| 1 | 0 | 17/4/2013 | 0 | 17/4/2013 | 0 |
| 1 | 0 | 17/4/2013 | 0 | 17/4/2013 | 0 |
| 1 | 0 | 17/4/2013 | 0 | 17/4/2013 | 0 |
| 1 | 0 | 17/4/2013 | 0 | 17/4/2013 | 0 |
| 1 | 0 | 17/4/2013 | 0 | 17/4/2013 | 0 |
| 1 | 0 | 17/4/2013 | 0 | 17/4/2013 | 0 |
| 1 | 0 | 17/4/2013 | 0 | 17/4/2013 | 0 |
| 1 | 0 | 17/4/2013 | 0 | 17/4/2013 | 0 |
| 0 | | 17/4/2013 | | | |
| 1 | 0 | 17/4/2013 | 0 | 17/4/2013 | 0 |
| 1 | 0 | 17/4/2013 | 0 | 17/4/2013 | 0 |
| 1 | 0 | 17/4/2013 | 0 | 17/4/2013 | 0 |
| 1 | 0 | 17/4/2013 | 0 | 17/4/2013 | MISSING |

| | | | | | |
|---|---|---|---|---|---|
| 1 | 0 | 17/4/2013 | 0 | 17/4/2013 | MISSING |
| 1 | 0 | 17/4/2013 | 0 | 17/4/2013 | |
| 1 | 0 | 17/4/2013 | 0 | 17/4/2013 | |
| 0 | | 17/4/2013 | | | |
| 1 | 0 | 17/4/2013 | 0 | 17/4/2013 | |
| 1 | 0 | 17/4/2013 | 0 | 17/4/2013 | |
| 1 | 0 | 17/4/2013 | 0 | 17/4/2013 | |
| 1 | 0 | 17/4/2013 | 0 | 17/4/2013 | |
| 1 | 4 | 17/4/2013 | 2 | 17/4/2013 | 2 |
| 1 | | 17/4/2013 | | | |
| 1 | 0 | 17/4/2013 | 0 | 17/4/2013 | |
| 0 | | 17/4/2013 | | | |
| 1 | 0 | 17/4/2013 | 0 | 17/4/2013 | |
| 1 | 4 | 17/4/2013 | 5 | 17/4/2013 | 4 |
| 0 | | 17/4/2013 | | | |
| 0 | | 17/4/2013 | | | |
| 1 | 0 | 17/4/2013 | 0 | 17/4/2013 | |
| 1 | 0 | 17/4/2013 | 0 | 17/4/2013 | |
| 1 | 0 | 17/4/2013 | 0 | 17/4/2013 | |
| 1 | 0 | 17/4/2013 | 0 | 17/4/2013 | |
| 1 | 0 | 17/4/2013 | 0 | 17/4/2013 | 0 |
| 1 | 0 | 17/4/2013 | 0 | 17/4/2013 | |
| 1 | 0 | 17/4/2013 | 0 | 17/4/2013 | |
| 1 | 0 | 17/4/2013 | 0 | 17/4/2013 | |
| 1 | 0 | 17/4/2013 | 0 | 17/4/2013 | |
| 1 | 0 | 17/4/2013 | | | |
| 0 | | 17/4/2013 | | | |
| 0 | | 17/4/2013 | | | |
| 0 | | 17/4/2013 | | | |
| 0 | | 17/4/2013 | | | |
| 1 | 0 | 17/4/2013 | 0 | 17/4/2013 | |
| 1 | 0 | 04/03/2013 | 0 | 04/03/2013 | 0 |
| 1 | 0 | 04/03/2013 | 0 | 04/03/2013 | |
| 1 | 0 | 04/03/2013 | 0 | 04/03/2013 | |
| 1 | 0 | 04/03/2013 | 0 | 04/03/2013 | |
| 1 | 0 | 04/03/2013 | 0 | 04/03/2013 | |
| 1 | 0 | 04/03/2013 | 0 | 04/03/2013 | |
| 1 | 0 | 04/03/2013 | 0 | 04/03/2013 | |
| 1 | 0 | 04/03/2013 | 0 | 04/03/2013 | 0 |
| 1 | 0 | 04/03/2013 | 0 | 04/03/2013 | |
| 1 | 0 | 04/03/2013 | 0 | 04/03/2013 | 0 |
| 1 | 0 | 04/03/2013 | 0 | 04/03/2013 | 0 |
| 1 | 0 | 04/03/2013 | 0 | 04/03/2013 | 0 |
| 0 | | 04/03/2013 | | | |
| 1 | 0 | 04/03/2013 | 0 | 04/03/2013 | 0 |
| 1 | 0 | 04/03/2013 | 0 | 04/03/2013 | 0 |
| 1 | 0 | 04/03/2013 | 0 | 04/03/2013 | 0 |
| 1 | 0 | 04/03/2013 | 0 | 04/03/2013 | 0 |
| 1 | 0 | 04/03/2013 | 0 | 04/03/2013 | 0 |
| 1 | 0 | 04/03/2013 | 0 | 04/03/2013 | 0 |

| | | | | | |
|---|---|---|---|---|---|
| 1 | 0 | 04/03/2013 | 0 | 04/03/2013 | 0 |
| 1 | 0 | 04/03/2013 | 0 | 04/03/2013 | 0 |
| 1 | 0 | 04/03/2013 | 0 | 04/03/2013 | 0 |
| 1 | 0 | 04/03/2013 | 1 | 04/03/2013 | 1 |
| 1 | 0 | 04/03/2013 | 0 | 04/03/2013 | 0 |
| 1 | 0 | 04/03/2013 | 0 | 04/03/2013 | 0 |
| 1 | 0 | 04/03/2013 | 0 | 04/03/2013 | 0 |
| 1 | 0 | 04/03/2013 | 0 | 04/03/2013 | 0 |
| 1 | 0 | 04/03/2013 | 0 | 04/03/2013 | 0 |
| 1 | 0 | 04/03/2013 | 0 | 04/03/2013 | 0 |
| 1 | 2 | 04/03/2013 | 53 | 04/03/2013 | 57 |
| 1 | 0 | 04/03/2013 | 0 | 04/03/2013 | 0 |
| 1 | 0 | 04/03/2013 | 0 | 04/03/2013 | 0 |
| 1 | 0 | 04/03/2013 | 0 | 04/03/2013 | 0 |
| 1 | 0 | 04/03/2013 | 0 | 04/03/2013 | 0 |
| 1 | 0 | 04/03/2013 | 0 | 04/03/2013 | 0 |
| 0 | | 04/03/2013 | | | |
| 1 | 0 | 04/03/2013 | 0 | 04/03/2013 | 0 |
| 0 | | 04/03/2013 | | | |
| 1 | 0 | 04/03/2013 | 0 | 04/03/2013 | MISSING |
| 1 | 0 | 04/03/2013 | 0 | 04/03/2013 | |
| 1 | 0 | 04/03/2013 | 0 | 04/03/2013 | |
| 1 | 0 | 04/03/2013 | 0 | 04/03/2013 | |
| 1 | 0 | 04/03/2013 | 0 | 04/03/2013 | |
| 1 | 0 | 04/03/2013 | 0 | 04/03/2013 | 0 |
| 1 | 0 | 04/03/2013 | 0 | 04/03/2013 | 0 |
| 0 | | 04/03/2013 | | | |
| 1 | 0 | 04/03/2013 | 0 | 04/03/2013 | 0 |
| 1 | 0 | 04/03/2013 | 0 | 04/03/2013 | 0 |
| 1 | 0 | 04/03/2013 | 0 | 04/03/2013 | 0 |
| 0 | | 04/03/2013 | | | |
| 1 | 0 | 04/03/2013 | 0 | 04/03/2013 | |
| 1 | 0 | 04/03/2013 | 0 | 04/03/2013 | 0 |
| 1 | 0 | 04/03/2013 | 0 | 04/03/2013 | 0 |
| 1 | 0 | 04/03/2013 | 0 | 04/03/2013 | |
| 1 | 0 | 04/03/2013 | 0 | 04/03/2013 | 0 |
| 0 | | 04/03/2013 | | | |
| 1 | 0 | 04/03/2013 | 0 | 04/03/2013 | 0 |
| 0 | | 04/03/2013 | | | |
| 1 | 0 | 04/03/2013 | 0 | 04/03/2013 | 0 |
| 1 | 0 | 04/03/2013 | 0 | 04/03/2013 | 0 |
| 1 | 0 | 04/03/2013 | 0 | 04/03/2013 | 0 |
| 1 | 0 | 04/03/2013 | 0 | 04/03/2013 | 0 |
| 1 | 0 | 04/03/2013 | 0 | 04/03/2013 | |
| 1 | 0 | 04/03/2013 | 0 | 04/03/2013 | 0 |
| 1 | 0 | 04/03/2013 | 0 | 04/03/2013 | 0 |
| 1 | 0 | 04/03/2013 | 0 | 04/03/2013 | 0 |
| 1 | 0 | 04/03/2013 | 0 | 04/03/2013 | 20 |
| 0 | | 04/03/2013 | | | |
| 1 | 0 | 04/03/2013 | 0 | 04/03/2013 | 0 |

| | | | | | |
|---|---|---|---|---|---|
| 1 | 0 | 04/03/2013 | 0 | 04/03/2013 | 0 |
| 1 | 0 | 04/03/2013 | 0 | 04/03/2013 | 0 |
| 1 | 0 | 04/03/2013 | 0 | 04/03/2013 | 0 |
| 1 | 0 | 04/03/2013 | 0 | 04/03/2013 | 0 |
| 1 | 0 | 04/03/2013 | 0 | 04/03/2013 | 0 |
| 1 | 0 | 04/03/2013 | 0 | 04/03/2013 | 0 |
| 1 | 0 | 04/03/2013 | 0 | 04/03/2013 | |
| 1 | 0 | 04/03/2013 | 0 | 04/03/2013 | 0 |
| 0 | | 04/03/2013 | | | |
| 1 | 0 | 04/03/2013 | 0 | 04/03/2013 | 0 |
| 1 | 0 | 04/03/2013 | 0 | 04/03/2013 | 0 |
| 1 | 0 | 04/03/2013 | 0 | 04/03/2013 | 0 |
| 1 | 0 | 04/03/2013 | 0 | 04/03/2013 | 0 |
| 1 | 0 | 04/03/2013 | 0 | 04/03/2013 | 0 |
| 1 | 0 | 04/03/2013 | 0 | 04/03/2013 | 0 |
| 1 | 0 | 04/03/2013 | 0 | 04/03/2013 | 0 |
| 1 | 0 | 04/03/2013 | 0 | 04/03/2013 | 0 |
| 1 | 0 | 04/03/2013 | 0 | 04/03/2013 | 4 |
| 1 | 0 | 04/03/2013 | 0 | 04/03/2013 | 0 |
| 1 | 0 | 04/03/2013 | 0 | 04/03/2013 | 0 |
| 1 | 0 | 04/03/2013 | 0 | 04/03/2013 | 0 |
| 1 | 0 | 04/03/2013 | 0 | 04/03/2013 | 0 |
| 1 | 0 | 04/03/2013 | 0 | 04/03/2013 | 0 |
| 0 | | 04/03/2013 | | | |
| 1 | 0 | 04/03/2013 | 0 | 04/03/2013 | |
| 1 | 0 | 04/03/2013 | 0 | 04/03/2013 | 0 |
| 1 | 0 | 04/03/2013 | 0 | 04/03/2013 | 0 |
| 1 | 0 | 04/03/2013 | 0 | 04/03/2013 | 0 |
| 1 | 0 | 04/03/2013 | 0 | 04/03/2013 | 0 |
| 0 | | 04/03/2013 | | | |
| 0 | | 04/03/2013 | | | |
| 1 | 0 | 04/03/2013 | 0 | 04/03/2013 | |
| 1 | 0 | 04/03/2013 | 0 | 04/03/2013 | |
| 1 | 0 | 04/03/2013 | 0 | 04/03/2013 | 0 |
| 1 | 0 | 04/03/2013 | 0 | 04/03/2013 | |
| 1 | 0 | 04/03/2013 | 0 | 04/03/2013 | |
| 1 | 0 | 04/03/2013 | 0 | 04/03/2013 | |
| 1 | 0 | 04/03/2013 | 0 | 04/03/2013 | |
| 1 | 0 | 04/03/2013 | 0 | 04/03/2013 | |
| 1 | 0 | 04/03/2013 | 0 | 04/03/2013 | |
| 1 | 0 | 04/03/2013 | 0 | 04/03/2013 | |
| 1 | 0 | 04/03/2013 | 0 | 04/03/2013 | |
| 0 | | 04/03/2013 | | | |
| 1 | 0 | 04/03/2013 | 0 | 04/03/2013 | 0 |
| 1 | 0 | 04/03/2013 | 0 | 04/03/2013 | |
| 0 | | 04/03/2013 | | | |
| 1 | 0 | 04/03/2013 | 0 | 04/03/2013 | |
| 1 | 0 | 04/03/2013 | 0 | 04/03/2013 | |
| 1 | 0 | 04/03/2013 | 0 | 04/03/2013 | |
| 1 | 0 | 04/03/2013 | 0 | 04/03/2013 | |

| | | | | | |
|---|---|---|---|---|---|
| 1 | 0 | 04/03/2013 | 0 | 04/03/2013 | |
| 1 | 0 | 04/03/2013 | 0 | 04/03/2013 | |
| 1 | 0 | 04/03/2013 | 0 | 04/03/2013 | |
| 1 | 0 | 04/03/2013 | 0 | 04/03/2013 | |
| 1 | 0 | 04/03/2013 | 0 | 04/03/2013 | |
| 1 | 0 | 04/03/2013 | 0 | 04/03/2013 | |
| 1 | 0 | 04/03/2013 | 0 | 04/03/2013 | |
| 1 | 0 | 04/03/2013 | 0 | 04/03/2013 | |
| 1 | 0 | 04/03/2013 | 0 | 04/03/2013 | |
| 1 | 0 | 04/03/2013 | 0 | 04/03/2013 | |
| 1 | 0 | 04/03/2013 | 0 | 04/03/2013 | |
| 1 | | | | | |
| 1 | 2 | 22/4/2013 | 32 | 22/4/2013 | 35 |
| 1 | 0 | 22/4/2013 | 0 | 22/4/2013 | 0 |
| 1 | 2 | 22/4/2013 | 0 | 22/4/2013 | 0 |
| 0 | | 22/4/2013 | | | |
| 1 | 2 | 22/4/2013 | 0 | 22/4/2013 | 0 |
| 1 | 0 | 22/4/2013 | 0 | 22/4/2013 | 0 |
| 1 | 0 | 22/4/2013 | 0 | 22/4/2013 | 0 |
| 1 | 0 | 22/4/2013 | 0 | 22/4/2013 | 0 |
| 1 | 0 | 22/4/2013 | 0 | 22/4/2013 | 0 |
| 1 | 0 | 22/4/2013 | 0 | 22/4/2013 | 0 |
| 1 | 0 | 22/4/2013 | 0 | 22/4/2013 | 0 |
| 1 | 0 | 22/4/2013 | 0 | 22/4/2013 | 0 |
| 1 | 0 | 22/4/2013 | 0 | 22/4/2013 | 0 |
| 1 | 0 | 22/4/2013 | 0 | 22/4/2013 | 0 |
| 1 | 0 | 22/4/2013 | 0 | 22/4/2013 | 0 |
| 0 | | 22/4/2013 | | | |
| 0 | | 22/4/2013 | | | |
| 1 | 0 | 22/4/2013 | 0 | 22/4/2013 | 0 |
| 0 | | 22/4/2013 | | | |
| 1 | 0 | 22/4/2013 | 0 | 22/4/2013 | 0 |
| 0 | | 22/4/2013 | | | |
| 1 | 0 | 22/4/2013 | 0 | 22/4/2013 | 0 |
| 1 | 0 | 22/4/2013 | 0 | 22/4/2013 | 0 |
| 1 | 0 | 22/4/2013 | 0 | 22/4/2013 | 0 |
| 1 | 0 | 22/4/2013 | 0 | 22/4/2013 | 0 |
| 1 | 0 | 22/4/2013 | 0 | 22/4/2013 | 0 |
| 0 | | 22/4/2013 | | | |
| 1 | 0 | 22/4/2013 | 0 | 22/4/2013 | 0 |
| 1 | 0 | 22/4/2013 | 0 | 22/4/2013 | 0 |
| 1 | 4 | 22/4/2013 | 0 | 22/4/2013 | 2 |
| 1 | 0 | 22/4/2013 | 0 | 22/4/2013 | 0 |
| 1 | 0 | 22/4/2013 | 0 | 22/4/2013 | 0 |
| 1 | 0 | 22/4/2013 | 0 | 22/4/2013 | 0 |
| 0 | | 22/4/2013 | | | |
| 1 | 0 | 22/4/2013 | 0 | 22/4/2013 | 0 |
| 1 | 0 | 22/4/2013 | 0 | 22/4/2013 | 0 |
| 1 | 0 | 22/4/2013 | 0 | 22/4/2013 | 0 |
| 0 | | 22/4/2013 | | | |

| | | | | | |
|---|---|---|---|---|---|
| 1 | 0 | 22/4/2013 | 0 | 22/4/2013 | 0 |
| 0 | | 22/4/2013 | | | |
| 1 | 0 | 22/4/2013 | 0 | 22/4/2013 | 0 |
| 0 | | 22/4/2013 | | | |
| 1 | 0 | 22/4/2013 | 0 | 22/4/2013 | 0 |
| 0 | | 22/4/2013 | | | |
| 1 | 0 | 22/4/2013 | 0 | 22/4/2013 | 0 |
| 1 | 0 | 22/4/2013 | 0 | 22/4/2013 | 0 |
| 0 | | 22/4/2013 | | | |
| 1 | 2 | 22/4/2013 | 0 | 22/4/2013 | 2 |
| 1 | | 22/4/2013 | | | |
| 1 | 0 | 22/4/2013 | 0 | 22/4/2013 | 0 |
| 1 | 0 | 22/4/2013 | 0 | 22/4/2013 | 0 |
| 1 | 0 | 22/4/2013 | 0 | 22/4/2013 | 0 |
| 1 | 0 | 22/4/2013 | 0 | 22/4/2013 | 0 |
| 1 | 0 | 22/4/2013 | 0 | 22/4/2013 | 0 |
| 0 | | 22/4/2013 | | | |
| 1 | 0 | 22/4/2013 | 0 | 22/4/2013 | 0 |
| 0 | | 22/4/2013 | | | |
| 1 | 0 | 22/4/2013 | 0 | 22/4/2013 | 0 |
| 0 | | 22/4/2013 | | | |
| 0 | | 22/4/2013 | | | |
| 0 | | 22/4/2013 | | | |
| 1 | 0 | 22/4/2013 | 0 | 22/4/2013 | 0 |
| 1 | 0 | 22/4/2013 | 0 | 22/4/2013 | 0 |
| 0 | | 22/4/2013 | | | |
| 1 | 3 | 22/4/2013 | 110 | 22/4/2013 | 8 |
| 1 | 0 | 22/4/2013 | 0 | 22/4/2013 | 0 |
| 1 | 0 | 22/4/2013 | 0 | 22/4/2013 | 0 |
| 1 | 0 | 22/4/2013 | 0 | 22/4/2013 | 0 |
| 1 | 0 | 22/4/2013 | 0 | 22/4/2013 | MISSING |
| 1 | 0 | 22/4/2013 | 0 | 22/4/2013 | 0 |
| 1 | 0 | 22/4/2013 | 0 | 22/4/2013 | 0 |
| 1 | 0 | 22/4/2013 | 0 | 22/4/2013 | 0 |
| 1 | 0 | 22/4/2013 | 0 | 22/4/2013 | 0 |
| 1 | 0 | 22/4/2013 | 0 | 22/4/2013 | 0 |
| 1 | 0 | 22/4/2013 | 0 | 22/4/2013 | 0 |
| 1 | 0 | 22/4/2013 | 0 | 22/4/2013 | 0 |
| 0 | | 22/4/2013 | | | |
| 1 | 0 | 22/4/2013 | 0 | 22/4/2013 | 0 |
| 1 | 0 | 22/4/2013 | 0 | 22/4/2013 | 0 |
| 1 | 0 | 22/4/2013 | 0 | 22/4/2013 | 0 |
| 1 | 0 | 22/4/2013 | 0 | 22/4/2013 | 0 |
| 1 | 0 | 22/4/2013 | 0 | 22/4/2013 | 0 |
| 1 | 0 | 22/4/2013 | 0 | 22/4/2013 | 0 |
| 1 | 0 | 22/4/2013 | 0 | 22/4/2013 | 0 |
| 0 | | 22/4/2013 | | | |
| 0 | | 22/4/2013 | | | |
| 0 | | 22/4/2013 | | | |
| 1 | 0 | 22/4/2013 | 0 | 22/4/2013 | 0 |

| | | | | | |
|---|---|---|---|---|---|
| 1 | 0 | 22/4/2013 | 0 | 22/4/2013 | 0 |
| 1 | 0 | 22/4/2013 | 0 | 22/4/2013 | 0 |
| 1 | 0 | 22/4/2013 | 0 | 22/4/2013 | 0 |
| 1 | 0 | 22/4/2013 | 0 | 22/4/2013 | 0 |
| 1 | 0 | 22/4/2013 | 0 | 22/4/2013 | 0 |
| 1 | 0 | 22/4/2013 | 0 | 22/4/2013 | 0 |
| 1 | 0 | 22/4/2013 | 0 | 22/4/2013 | 0 |
| 1 | 0 | 22/4/2013 | 0 | 22/4/2013 | 0 |
| 1 | 0 | 22/4/2013 | 0 | 22/4/2013 | 0 |
| 1 | 0 | 22/4/2013 | 0 | 22/4/2013 | 0 |
| 1 | 0 | 22/4/2013 | 0 | 22/4/2013 | 0 |
| 1 | 0 | 22/4/2013 | 0 | 22/4/2013 | 0 |
| 1 | 0 | 22/4/2013 | 0 | 22/4/2013 | |
| 1 | 0 | 22/4/2013 | 0 | 22/4/2013 | |
| 1 | 0 | 22/4/2013 | 0 | 22/4/2013 | |
| 1 | 3 | 22/4/2013 | 0 | 22/4/2013 | 0 |
| 1 | 0 | 22/4/2013 | 0 | 22/4/2013 | |
| 0 | | 22/4/2013 | | | |
| 1 | 0 | 22/4/2013 | 0 | 22/4/2013 | |
| 1 | 0 | 22/4/2013 | 0 | 22/4/2013 | |
| 1 | 0 | 22/4/2013 | 0 | 22/4/2013 | |
| 1 | 0 | 22/4/2013 | 0 | 22/4/2013 | |
| 1 | 0 | 22/4/2013 | 0 | 22/4/2013 | |
| 1 | 0 | 22/4/2013 | 0 | 22/4/2013 | |
| 1 | 2 | 22/4/2013 | 98 | 22/4/2013 | 110 |
| 1 | 0 | 22/4/2013 | 0 | 22/4/2013 | |
| 1 | 0 | 22/4/2013 | 0 | 22/4/2013 | |
| 1 | 0 | 22/4/2013 | 0 | 22/4/2013 | |
| 1 | 0 | 22/4/2013 | 0 | 22/4/2013 | |
| 0 | | 22/4/2013 | | | |
| 1 | 0 | 22/4/2013 | 0 | 22/4/2013 | |
| 1 | 0 | 22/4/2013 | 0 | 22/4/2013 | |
| 1 | 0 | 22/4/2013 | 0 | 22/4/2013 | |
| 1 | 0 | 22/4/2013 | 0 | 22/4/2013 | |
| 1 | 0 | 22/4/2013 | 0 | 22/4/2013 | |
| 1 | 0 | 22/4/2013 | 0 | 22/4/2013 | |
| 1 | 0 | 22/4/2013 | 0 | 22/4/2013 | |
| 1 | 0 | 22/4/2013 | 0 | 22/4/2013 | |
| 0 | | 22/4/2013 | | | |
| 0 | | 22/4/2013 | | | |
| 1 | 0 | 22/4/2013 | 0 | 22/4/2013 | |
| 1 | 0 | 22/4/2013 | 0 | 22/4/2013 | |
| 1 | 0 | 25/4/2013 | 0 | 25/4/2013 | 0 |
| 0 | | 25/4/2013 | | | |
| 0 | | 25/4/2013 | | | |
| 1 | 0 | 25/4/2013 | 0 | 25/4/2013 | 0 |
| 0 | | 25/4/2013 | | | |
| 1 | 0 | 25/4/2013 | 0 | 25/4/2013 | 0 |
| 1 | 0 | 25/4/2013 | | | |
| 1 | 0 | 25/4/2013 | 0 | 25/4/2013 | 0 |

| | | | | | |
|---|---|---|---|---|---|
| 1 | 1 | 25/4/2013 | 0 | 25/4/2013 | 0 |
| 1 | 0 | 25/4/2013 | 0 | 25/4/2013 | 0 |
| 1 | 0 | 25/4/2013 | 0 | 25/4/2013 | 0 |
| 1 | 0 | 25/4/2013 | 0 | 25/4/2013 | 0 |
| 0 | | 25/4/2013 | | | |
| 1 | 0 | 25/4/2013 | 0 | 25/4/2013 | 0 |
| 1 | 0 | 25/4/2013 | 0 | 25/4/2013 | 0 |
| 1 | 0 | 25/4/2013 | 0 | 25/4/2013 | 0 |
| 1 | 0 | 25/4/2013 | 0 | 25/4/2013 | 0 |
| 1 | 0 | 25/4/2013 | 0 | 25/4/2013 | 0 |
| 1 | 0 | 25/4/2013 | 0 | 25/4/2013 | 0 |
| 1 | 0 | 25/4/2013 | 0 | 25/4/2013 | 0 |
| 1 | 0 | 25/4/2013 | 0 | 25/4/2013 | 0 |
| 0 | | 25/4/2013 | | | |
| 1 | 0 | 25/4/2013 | 0 | 25/4/2013 | 0 |
| 1 | 0 | 25/4/2013 | 0 | 25/4/2013 | 0 |
| 1 | | 25/4/2013 | | | |
| 1 | 0 | 25/4/2013 | 0 | 25/4/2013 | 0 |
| 1 | 0 | 25/4/2013 | 0 | 25/4/2013 | 0 |
| 1 | 0 | 25/4/2013 | 0 | 25/4/2013 | 0 |
| 1 | 1 | 25/4/2013 | 23 | 25/4/2013 | 30 |
| 0 | | 25/4/2013 | | | |
| 1 | 0 | 25/4/2013 | 0 | 25/4/2013 | 0 |
| 1 | 0 | 25/4/2013 | 0 | 25/4/2013 | 0 |
| 1 | 0 | 25/4/2013 | 0 | 25/4/2013 | 0 |
| 1 | 0 | 25/4/2013 | 0 | 25/4/2013 | 0 |
| 1 | 0 | 25/4/2013 | 0 | 25/4/2013 | 0 |
| 1 | 0 | 25/4/2013 | 0 | 25/4/2013 | 0 |
| 1 | 0 | 25/4/2013 | 0 | 25/4/2013 | 0 |
| 1 | 0 | 25/4/2013 | 0 | 25/4/2013 | 0 |
| 1 | 0 | 25/4/2013 | 0 | 25/4/2013 | 0 |
| 0 | 0 | 25/4/2013 | 0 | 25/4/2013 | 0 |
| 1 | | 25/4/2013 | | | |
| 1 | 0 | 25/4/2013 | 0 | 25/4/2013 | 0 |
| 1 | 3 | 25/4/2013 | 150 | 25/4/2013 | 138 |
| 1 | 0 | 25/4/2013 | 0 | 25/4/2013 | 0 |
| 1 | 0 | 25/4/2013 | 0 | 25/4/2013 | 0 |
| 1 | 0 | 25/4/2013 | 0 | 25/4/2013 | 0 |
| 1 | 3 | 25/4/2013 | 112 | 25/4/2013 | 4 |
| 1 | 0 | 25/4/2013 | 0 | 25/4/2013 | 0 |
| 1 | 0 | 25/4/2013 | 0 | 25/4/2013 | 0 |
| 1 | 0 | 25/4/2013 | 0 | 25/4/2013 | 0 |
| 1 | 0 | 25/4/2013 | 0 | 25/4/2013 | 0 |
| 1 | 0 | 25/4/2013 | 0 | 25/4/2013 | 0 |
| 0 | | 25/4/2013 | | | |
| 1 | 3 | 25/4/2013 | 4 | 25/4/2013 | 5 |
| 1 | 0 | 25/4/2013 | 0 | 25/4/2013 | 0 |
| 1 | 0 | 25/4/2013 | 0 | 25/4/2013 | 0 |
| 1 | | 25/4/2013 | | | |
| 1 | 0 | 25/4/2013 | 0 | 25/4/2013 | 0 |

| | | | | | | |
|---|---|---|---|---|---|---|
| 1 | 0 | 25/4/2013 | 0 | 25/4/2013 | 0 |
| 1 | 0 | 25/4/2013 | 0 | 25/4/2013 | 0 |
| 1 | 0 | 25/4/2013 | 0 | 25/4/2013 | 0 |
| 1 | 0 | 25/4/2013 | 0 | 25/4/2013 | 0 |
| 1 | 0 | 25/4/2013 | 0 | 25/4/2013 | 0 |
| 1 | 0 | 25/4/2013 | 0 | 25/4/2013 | 0 |
| 1 | 3 | 25/4/2013 | 101 | 25/4/2013 | 85 |
| 1 | 0 | 25/4/2013 | 0 | 25/4/2013 | 0 |
| 1 | 3 | 25/4/2013 | 112 | 25/4/2013 | 139 |
| 0 | | 25/4/2013 | | | |
| 1 | 0 | 25/4/2013 | 0 | 25/4/2013 | 0 |
| 0 | | 25/4/2013 | | | |
| 1 | 0 | 25/4/2013 | 0 | 25/4/2013 | 0 |
| 1 | 0 | 25/4/2013 | 0 | 25/4/2013 | 0 |
| 0 | | 25/4/2013 | | | |
| 1 | | 25/4/2013 | | | |
| 1 | 0 | 25/4/2013 | 0 | 25/4/2013 | 0 |
| 0 | | 25/4/2013 | | | |
| 1 | 0 | 25/4/2013 | 0 | 25/4/2013 | 0 |
| 1 | 0 | 25/4/2013 | 0 | 25/4/2013 | 0 |
| 0 | | 25/4/2013 | | | |
| 1 | 0 | 25/4/2013 | 0 | 25/4/2013 | 0 |
| 1 | 0 | 25/4/2013 | 14 | 25/4/2013 | 34 |
| 1 | 4 | 25/4/2013 | 0 | 25/4/2013 | 0 |
| 1 | 0 | 25/4/2013 | 0 | 25/4/2013 | 0 |
| 1 | 0 | 25/4/2013 | 0 | 25/4/2013 | 0 |
| 0 | | 25/4/2013 | | | |
| 1 | 0 | 25/4/2013 | 0 | 25/4/2013 | 0 |
| 1 | 3 | 25/4/2013 | 0 | 25/4/2013 | 0 |
| 1 | 0 | 25/4/2013 | 0 | 25/4/2013 | 0 |
| 1 | 0 | 25/4/2013 | 0 | 25/4/2013 | 0 |
| 1 | 0 | 25/4/2013 | 0 | 25/4/2013 | 0 |
| 1 | 0 | 25/4/2013 | 0 | 25/4/2013 | 0 |
| 1 | 0 | 25/4/2013 | 0 | 25/4/2013 | 0 |
| 1 | 0 | 25/4/2013 | 0 | 25/4/2013 | 0 |
| 1 | 0 | 25/4/2013 | 0 | 25/4/2013 | 0 |
| 0 | | 25/4/2013 | | | |
| 1 | 0 | 25/4/2013 | 0 | 25/4/2013 | 0 |
| 1 | 0 | 25/4/2013 | 0 | 25/4/2013 | 0 |
| 1 | 0 | 25/4/2013 | 0 | 25/4/2013 | 0 |
| 1 | 0 | 25/4/2013 | 0 | 25/4/2013 | 0 |
| 1 | 1 | 25/4/2013 | 23 | 25/4/2013 | 32 |
| 0 | | 25/4/2013 | | | |
| 1 | 0 | 25/4/2013 | 0 | 25/4/2013 | |
| 1 | 0 | 25/4/2013 | 0 | 25/4/2013 | |
| 1 | 0 | 25/4/2013 | 0 | 25/4/2013 | |
| 1 | 0 | 25/4/2013 | 0 | 25/4/2013 | |
| 1 | 0 | 25/4/2013 | 0 | 25/4/2013 | |
| 0 | | 25/4/2013 | | | |
| 1 | 0 | 25/4/2013 | 0 | 25/4/2013 | |

| | | | | | |
|---|---|---|---|---|---|
| 1 | 0 | 25/4/2013 | 0 | 25/4/2013 | |
| 1 | 1 | 25/4/2013 | 0 | 25/4/2013 | 20 |
| 1 | 0 | 25/4/2013 | 0 | 25/4/2013 | |
| 1 | 0 | 25/4/2013 | 0 | 25/4/2013 | |
| 1 | 0 | 25/4/2013 | 0 | 25/4/2013 | |
| 1 | 0 | 25/4/2013 | 0 | 25/4/2013 | |
| 1 | 0 | 25/4/2013 | 0 | 25/4/2013 | 0 |
| 1 | 0 | 25/4/2013 | 0 | 25/4/2013 | |
| 1 | 0 | 27/2/2013 | 0 | 28/2/2013 | 0 |
| 0 | | 27/2/2013 | | | |
| 1 | 0 | 27/2/2013 | 0 | 28/2/2013 | 0 |
| 1 | 0 | 27/2/2013 | 0 | 28/2/2013 | 0 |
| 1 | 0 | 27/2/2013 | 0 | 28/2/2013 | 0 |
| 1 | 0 | 27/2/2013 | 0 | 28/2/2013 | 0 |
| 1 | 0 | 27/2/2013 | 0 | 28/2/2013 | 0 |
| 1 | 0 | 27/2/2013 | 0 | 28/2/2013 | 0 |
| 1 | 0 | 27/2/2013 | 0 | 28/2/2013 | 0 |
| 1 | 0 | 27/2/2013 | 0 | 28/2/2013 | 0 |
| 1 | 0 | 27/2/2013 | 0 | 28/2/2013 | 0 |
| 1 | 0 | 27/2/2013 | | | 0 |
| 1 | 0 | 27/2/2013 | 0 | 28/2/2013 | 0 |
| 1 | 0 | 27/2/2013 | 0 | 28/2/2013 | 0 |
| 0 | | 27/2/2013 | | | |
| 1 | 0 | 27/2/2013 | 0 | 28/2/2013 | 0 |
| 1 | 0 | 27/2/2013 | 0 | 28/2/2013 | 1 |
| 1 | 0 | 27/2/2013 | 0 | 28/2/2013 | 0 |
| 1 | 0 | 27/2/2013 | 0 | 28/2/2013 | 0 |
| 1 | 0 | 27/2/2013 | 0 | 28/2/2013 | 0 |
| 1 | 0 | 27/2/2013 | 0 | 28/2/2013 | 0 |
| 1 | 0 | 27/2/2013 | 0 | 28/2/2013 | 0 |
| 1 | 0 | 27/2/2013 | 0 | 28/2/2013 | 0 |
| 1 | 0 | 27/2/2013 | 0 | 28/2/2013 | 0 |
| 1 | 0 | 27/2/2013 | 0 | 28/2/2013 | 0 |
| 1 | 0 | 27/2/2013 | 0 | 28/2/2013 | 0 |
| 0 | | 27/2/2013 | | | |
| 0 | | 27/2/2013 | | | |
| 1 | 0 | 27/2/2013 | 0 | 28/2/2013 | 0 |
| 1 | 0 | 27/2/2013 | 0 | 28/2/2013 | 0 |
| 1 | 0 | 27/2/2013 | 0 | 28/2/2013 | 0 |
| 1 | 0 | 27/2/2013 | 0 | 28/2/2013 | 0 |
| 1 | 0 | 27/2/2013 | 0 | 28/2/2013 | 0 |
| 1 | 0 | 27/2/2013 | 0 | 28/2/2013 | 0 |
| 1 | 0 | 27/2/2013 | 0 | 28/2/2013 | 0 |
| 1 | 0 | 27/2/2013 | 0 | 28/2/2013 | 0 |
| 1 | 0 | 27/2/2013 | 0 | 28/2/2013 | 0 |
| 1 | 0 | 27/2/2013 | 0 | 28/2/2013 | 0 |
| 1 | 0 | 27/2/2013 | 0 | 28/2/2013 | 0 |
| 0 | | 27/2/2013 | | | |
| 1 | 0 | 27/2/2013 | 0 | 28/2/2013 | 0 |
| 0 | | 27/2/2013 | | | |

| | | | | | |
|---|---|---|---|---|---|
| 1 | 0 | 27/2/2013 | 0 | 28/2/2013 | 0 |
| 1 | 0 | 27/2/2013 | 0 | 28/2/2013 | 0 |
| 1 | 0 | 27/2/2013 | 0 | 28/2/2013 | 0 |
| 1 | 0 | 27/2/2013 | 0 | 28/2/2013 | 0 |
| 0 | | 27/2/2013 | | | |
| 1 | 0 | 27/2/2013 | 0 | 28/2/2013 | 0 |
| 0 | | 27/2/2013 | | | |
| 1 | 0 | 27/2/2013 | 0 | 28/2/2013 | 0 |
| 1 | 0 | 27/2/2013 | 0 | 28/2/2013 | 0 |
| 1 | 0 | 27/2/2013 | 0 | 28/2/2013 | 0 |
| 1 | 0 | 27/2/2013 | 0 | 28/2/2013 | 0 |
| 1 | 0 | 27/2/2013 | 0 | 28/2/2013 | 0 |
| 1 | 0 | 27/2/2013 | 0 | 28/2/2013 | 0 |
| 1 | 0 | 27/2/2013 | 0 | 28/2/2013 | 0 |
| 0 | | 27/2/2013 | | | |
| 1 | 0 | 27/2/2013 | 0 | 28/2/2013 | 0 |
| 1 | 0 | 27/2/2013 | 0 | 28/2/2013 | 0 |
| 1 | 0 | 27/2/2013 | 0 | 28/2/2013 | 0 |
| 1 | 0 | 27/2/2013 | 0 | 28/2/2013 | 0 |
| 1 | 0 | 27/2/2013 | 0 | 28/2/2013 | 2 |
| 1 | 0 | 27/2/2013 | 0 | 28/2/2013 | 0 |
| 0 | | 27/2/2013 | | | |
| 0 | | 27/2/2013 | | | |
| 1 | 0 | 27/2/2013 | 0 | 28/2/2013 | 0 |
| 1 | 0 | 27/2/2013 | 0 | 28/2/2013 | 0 |
| 0 | | 27/2/2013 | | | |
| 1 | 0 | 27/2/2013 | 0 | 28/2/2013 | 0 |
| 1 | 0 | 27/2/2013 | 0 | 28/2/2013 | 0 |
| 1 | 0 | 27/2/2013 | 0 | 28/2/2013 | 0 |
| 1 | 0 | 27/2/2013 | 0 | 28/2/2013 | 0 |
| 1 | 0 | 27/2/2013 | 0 | 28/2/2013 | 0 |
| 1 | 0 | 27/2/2013 | 0 | 28/2/2013 | 0 |
| 1 | 0 | 27/2/2013 | 0 | 28/2/2013 | 0 |
| 1 | 0 | 27/2/2013 | 0 | 28/2/2013 | 0 |
| 1 | 0 | 27/2/2013 | 0 | 28/2/2013 | 0 |
| 0 | | 27/2/2013 | | | |
| 1 | 0 | 27/2/2013 | 0 | 28/2/2013 | 0 |
| 0 | | 27/2/2013 | | | |
| 1 | 0 | 27/2/2013 | 0 | 28/2/2013 | 0 |
| 1 | 0 | 27/2/2013 | 0 | 28/2/2013 | 0 |
| 1 | 0 | 27/2/2013 | 0 | 28/2/2013 | 0 |
| 1 | 0 | 27/2/2013 | 0 | 28/2/2013 | 0 |
| 1 | 0 | 27/2/2013 | 0 | 28/2/2013 | 0 |
| 0 | | 27/2/2013 | | | |
| 1 | 0 | 27/2/2013 | 0 | 28/2/2013 | MISSING |
| 1 | 0 | 27/2/2013 | 0 | 28/2/2013 | 0 |
| 1 | 0 | 27/2/2013 | 0 | 28/2/2013 | 0 |
| 1 | 0 | 27/2/2013 | 0 | 28/2/2013 | 0 |
| 1 | 0 | 27/2/2013 | 0 | 28/2/2013 | 0 |
| 1 | 0 | 27/2/2013 | 8 | 28/2/2013 | 10 |

| | | | | | |
|---|---|---|---|---|---|
| 1 | 0 | 27/2/2013 | 0 | 28/2/2013 | 0 |
| 1 | 0 | 27/2/2013 | 0 | 28/2/2013 | 0 |
| 0 | | 27/2/2013 | | | |
| 1 | 0 | 27/2/2013 | 0 | 28/2/2013 | 3 |
| 0 | | 27/2/2013 | | | |
| 1 | 0 | 27/2/2013 | 0 | 28/2/2013 | 1 |
| 1 | 0 | 27/2/2013 | 0 | 28/2/2013 | 0 |
| 1 | 0 | 27/2/2013 | 0 | 28/2/2013 | 0 |
| 1 | 0 | 27/2/2013 | 0 | 28/2/2013 | |
| 1 | 0 | 27/2/2013 | 0 | 28/2/2013 | |
| 1 | 0 | 27/2/2013 | 0 | 28/2/2013 | |
| 1 | 0 | 27/2/2013 | 0 | 28/2/2013 | |
| 1 | 0 | 27/2/2013 | 0 | 28/2/2013 | |
| 1 | 0 | 27/2/2013 | 0 | 28/2/2013 | 0 |
| 1 | 0 | 27/2/2013 | 0 | 28/2/2013 | |
| 1 | 0 | 27/2/2013 | 0 | 28/2/2013 | |
| 1 | 0 | 27/2/2013 | 0 | 28/2/2013 | |
| 1 | 0 | 27/2/2013 | 0 | 28/2/2013 | |
| 1 | 0 | 27/2/2013 | 0 | 28/2/2013 | |
| 1 | 0 | 27/2/2013 | 0 | 28/2/2013 | |
| 1 | 0 | 27/2/2013 | 0 | 28/2/2013 | 0 |
| 1 | 0 | 27/2/2013 | 0 | 28/2/2013 | |
| 1 | 0 | 27/2/2013 | 0 | 28/2/2013 | |
| 1 | 0 | 27/2/2013 | 0 | 28/2/2013 | |
| 1 | 0 | 27/2/2013 | 0 | 28/2/2013 | |
| 1 | 0 | 27/2/2013 | 12 | 28/2/2013 | |
| 1 | 0 | 27/2/2013 | 0 | 28/2/2013 | |
| 1 | 1 | 27/2/2013 | 0 | 28/2/2013 | 0 |
| 1 | 0 | 27/2/2013 | 0 | 28/2/2013 | |
| 1 | 0 | 27/2/2013 | 0 | 28/2/2013 | |
| 1 | 0 | 27/2/2013 | 0 | 28/2/2013 | |
| 1 | 0 | 27/2/2013 | 0 | 28/2/2013 | |
| 1 | 0 | 27/2/2013 | 0 | 28/2/2013 | |
| 1 | 0 | 27/2/2013 | 0 | 28/2/2013 | |
| 1 | 0 | 27/2/2013 | 0 | 28/2/2013 | |
| 1 | 0 | 27/2/2013 | 0 | 28/2/2013 | |
| 1 | 0 | 27/2/2013 | 0 | 28/2/2013 | |
| 1 | 0 | 27/2/2013 | 0 | 28/2/2013 | |
| 1 | 0 | 28/3/2013 | 0 | 28/3/2013 | |
| 1 | 0 | 28/3/2013 | 0 | 28/3/2013 | 0 |
| 1 | 0 | 28/3/2013 | 0 | 28/3/2013 | 0 |
| 0 | | 28/3/2013 | 0 | 28/3/2013 | |
| 0 | | 28/3/2013 | 0 | 28/3/2013 | |
| 1 | 0 | 28/3/2013 | 0 | 28/3/2013 | 0 |
| 1 | 0 | 28/3/2013 | 0 | 28/3/2013 | 0 |
| 1 | 0 | 28/3/2013 | 0 | 28/3/2013 | 0 |
| 0 | | 28/3/2013 | 0 | 28/3/2013 | 0 |
| 1 | 0 | 28/3/2013 | 0 | 28/3/2013 | |
| 1 | 0 | 28/3/2013 | 0 | 28/3/2013 | 0 |
| 1 | 0 | 28/3/2013 | 0 | 28/3/2013 | 0 |

| | | | | | |
|---|---|---|---|---|---|
| 1 | 0 | 28/3/2013 | 0 | 28/3/2013 | 0 |
| 1 | 0 | 28/3/2013 | 0 | 28/3/2013 | 0 |
| 1 | 4 | 28/3/2013 | 11 | 28/3/2013 | 10 |
| 1 | 0 | 28/3/2013 | 0 | 28/3/2013 | 0 |
| 1 | 0 | 28/3/2013 | 0 | 28/3/2013 | 0 |
| 1 | 0 | 28/3/2013 | 0 | 28/3/2013 | 0 |
| 0 | | 28/3/2013 | 0 | 28/3/2013 | |
| 0 | | 28/3/2013 | 0 | 28/3/2013 | |
| 1 | 0 | 28/3/2013 | 0 | 28/3/2013 | |
| 1 | 0 | 28/3/2013 | 0 | 28/3/2013 | 0 |
| 1 | 0 | 28/3/2013 | 0 | 28/3/2013 | 0 |
| 1 | | 28/3/2013 | 0 | 28/3/2013 | |
| 0 | | 28/3/2013 | 0 | 28/3/2013 | |
| 1 | 0 | 28/3/2013 | 0 | 28/3/2013 | 0 |
| 0 | | 28/3/2013 | 0 | 28/3/2013 | |
| 1 | 0 | 28/3/2013 | 0 | 28/3/2013 | 0 |
| 1 | 0 | 28/3/2013 | 0 | 28/3/2013 | 0 |
| 1 | 0 | 28/3/2013 | 0 | 28/3/2013 | 0 |
| 1 | 0 | 28/3/2013 | 0 | 28/3/2013 | 0 |
| 1 | 0 | 28/3/2013 | 0 | 28/3/2013 | 0 |
| 1 | 0 | 28/3/2013 | 0 | 28/3/2013 | 0 |
| 1 | 0 | 28/3/2013 | 0 | 28/3/2013 | 0 |
| 1 | 0 | 28/3/2013 | 0 | 28/3/2013 | 0 |
| 1 | 0 | 28/3/2013 | 0 | 28/3/2013 | 0 |
| 1 | 0 | 28/3/2013 | 0 | 28/3/2013 | 0 |
| 1 | 0 | 28/3/2013 | 0 | 28/3/2013 | 0 |
| 1 | 0 | 28/3/2013 | 0 | 28/3/2013 | 0 |
| 1 | 0 | 28/3/2013 | 0 | 28/3/2013 | 0 |
| 0 | | 28/3/2013 | 0 | 28/3/2013 | |
| 1 | 0 | 28/3/2013 | 0 | 28/3/2013 | 0 |
| 1 | 0 | 28/3/2013 | 0 | 28/3/2013 | 0 |
| 1 | 0 | 28/3/2013 | 0 | 28/3/2013 | 0 |
| 1 | 0 | 28/3/2013 | 0 | 28/3/2013 | 0 |
| 1 | 0 | 28/3/2013 | 0 | 28/3/2013 | 0 |
| 1 | 0 | 28/3/2013 | 0 | 28/3/2013 | 0 |
| 1 | 0 | 28/3/2013 | 0 | 28/3/2013 | 0 |
| 0 | | 28/3/2013 | 0 | 28/3/2013 | |
| 1 | 0 | 28/3/2013 | 0 | 28/3/2013 | |
| 0 | | 28/3/2013 | 0 | 28/3/2013 | |
| 1 | 0 | 28/3/2013 | 0 | 28/3/2013 | 0 |
| 1 | 0 | 28/3/2013 | 0 | 28/3/2013 | 0 |
| 1 | 0 | 28/3/2013 | 0 | 28/3/2013 | 0 |
| 1 | 0 | 28/3/2013 | 0 | 28/3/2013 | 0 |
| 1 | 0 | 28/3/2013 | 0 | 28/3/2013 | 0 |
| 1 | 0 | 28/3/2013 | 0 | 28/3/2013 | |
| 1 | 0 | 28/3/2013 | 0 | 28/3/2013 | 0 |
| 1 | 0 | 28/3/2013 | 0 | 28/3/2013 | 0 |
| 1 | 0 | 28/3/2013 | 0 | 28/3/2013 | 0 |
| 1 | 0 | 28/3/2013 | 0 | 28/3/2013 | 0 |
| 1 | 0 | 28/3/2013 | 0 | 28/3/2013 | 0 |

| | | | | | |
|---|---|---|---|---|---|
| 1 | 0 | 28/3/2013 | 0 | 28/3/2013 | 0 |
| 1 | 0 | 28/3/2013 | 0 | 28/3/2013 | 0 |
| 1 | 0 | 28/3/2013 | 0 | 28/3/2013 | 0 |
| 1 | 0 | 28/3/2013 | 0 | 28/3/2013 | 0 |
| 1 | 0 | 28/3/2013 | 0 | 28/3/2013 | 0 |
| 0 | | 28/3/2013 | 1 | 28/3/2013 | |
| 1 | 0 | 28/3/2013 | 0 | 28/3/2013 | 0 |
| 1 | 0 | 28/3/2013 | 0 | 28/3/2013 | 0 |
| 1 | 0 | 28/3/2013 | 0 | 28/3/2013 | 0 |
| 1 | 0 | 28/3/2013 | 0 | 28/3/2013 | 0 |
| 1 | 0 | 28/3/2013 | 0 | 28/3/2013 | 0 |
| 1 | 0 | 28/3/2013 | 0 | 28/3/2013 | 0 |
| 1 | 0 | 28/3/2013 | 0 | 28/3/2013 | 0 |
| 1 | 0 | | | | |
| 1 | 0 | 28/3/2013 | 0 | 28/3/2013 | 0 |
| 1 | 0 | 28/3/2013 | 0 | 28/3/2013 | 0 |
| 0 | | 28/3/2013 | 0 | 28/3/2013 | |
| 1 | 0 | 28/3/2013 | 0 | 28/3/2013 | 0 |
| 1 | 0 | 28/3/2013 | 0 | 28/3/2013 | 0 |
| 0 | | 28/3/2013 | 0 | 28/3/2013 | |
| 1 | 0 | 28/3/2013 | 0 | 28/3/2013 | 0 |
| 0 | | 28/3/2013 | 0 | 28/3/2013 | |
| 1 | 0 | 28/3/2013 | 0 | 28/3/2013 | 0 |
| 1 | 0 | 28/3/2013 | 0 | 28/3/2013 | 0 |
| 1 | 0 | 28/3/2013 | 0 | 28/3/2013 | 0 |
| 0 | | 28/3/2013 | 0 | 28/3/2013 | |
| 1 | 0 | 28/3/2013 | 0 | 28/3/2013 | 0 |
| 1 | 0 | 28/3/2013 | 0 | 28/3/2013 | 0 |
| 1 | 0 | 28/3/2013 | 0 | 28/3/2013 | 0 |
| 1 | 0 | 28/3/2013 | 0 | 28/3/2013 | 0 |
| 1 | 0 | 28/3/2013 | 0 | 28/3/2013 | 0 |
| 1 | 0 | 28/3/2013 | 0 | 28/3/2013 | MISSING |
| 1 | 0 | 28/3/2013 | 0 | 28/3/2013 | 0 |
| 1 | 0 | 28/3/2013 | 0 | 28/3/2013 | 0 |
| 0 | | 28/3/2013 | 0 | 28/3/2013 | |
| 1 | 0 | 28/3/2013 | 0 | 28/3/2013 | 0 |
| 1 | 0 | 28/3/2013 | 0 | 28/3/2013 | 0 |
| 1 | 0 | 28/3/2013 | 0 | 28/3/2013 | 0 |
| 1 | 0 | 28/3/2013 | 0 | 28/3/2013 | |
| 1 | 0 | 28/3/2013 | 0 | 28/3/2013 | |
| 1 | 0 | 28/3/2013 | 0 | 28/3/2013 | |
| 1 | 0 | 28/3/2013 | 0 | 28/3/2013 | |
| 1 | 0 | 28/3/2013 | 0 | 28/3/2013 | |
| 1 | 0 | 28/3/2013 | 0 | 28/3/2013 | |
| 1 | 0 | 28/3/2013 | 0 | 28/3/2013 | |
| 1 | 0 | 28/3/2013 | 0 | 28/3/2013 | |
| 1 | 0 | 28/3/2013 | 0 | 28/3/2013 | |
| 1 | 0 | 28/3/2013 | 0 | 28/3/2013 | |
| 1 | 0 | 28/3/2013 | | | |
| 1 | 0 | 28/3/2013 | 0 | 28/3/2013 | |

| | | | | | |
|---|---|---|---|---|---|
| 1 | 0 | 28/3/2013 | 0 | 28/3/2013 | |
| 1 | 0 | 28/3/2013 | 0 | 28/3/2013 | |
| 1 | 0 | 28/3/2013 | 0 | 28/3/2013 | |
| 1 | 0 | 28/3/2013 | 0 | 28/3/2013 | |
| 1 | 0 | 28/3/2013 | 0 | 28/3/2013 | |
| 1 | 0 | 28/3/2013 | 0 | 28/3/2013 | |
| 1 | 4 | 28/3/2013 | 1 | 28/3/2013 | |
| 1 | 0 | 28/3/2013 | 0 | 28/3/2013 | |
| 1 | 0 | 28/3/2013 | 0 | 28/3/2013 | |
| 0 | | 28/3/2013 | 0 | 28/3/2013 | |
| 1 | 0 | 28/3/2013 | 0 | 28/3/2013 | |
| 1 | 0 | 28/3/2013 | 0 | 28/3/2013 | |
| 1 | 0 | 28/3/2013 | 0 | 28/3/2013 | |
| 1 | 0 | 28/3/2013 | 0 | 28/3/2013 | |
| 0 | | 28/3/2013 | 0 | 28/3/2013 | |
| 1 | 0 | 28/3/2013 | 0 | 28/3/2013 | |
| 1 | 0 | 28/3/2013 | 0 | 28/3/2013 | |
| 1 | 0 | 28/3/2013 | 0 | 28/3/2013 | |
| 1 | 0 | 20/2/203 | 0 | 21/02/2013 | 0 |
| 1 | 0 | 20/2/203 | 0 | 21/02/2013 | 0 |
| 1 | 0 | 20/2/203 | 0 | 21/02/2013 | 0 |
| 1 | 0 | 20/2/203 | 0 | 21/02/2013 | 0 |
| 1 | 0 | 20/2/203 | 0 | 21/02/2013 | 0 |
| 1 | 0 | 20/2/203 | 0 | 21/02/2012 | 0 |
| 1 | 0 | 20/2/203 | 0 | 21/02/2012 | 0 |
| 1 | 0 | 20/2/203 | 0 | 21/02/2013 | |
| 0 | | 20/2/203 | | | |
| 0 | | 20/2/203 | | | |
| 1 | 0 | 20/2/203 | 0 | 21/02/2013 | 0 |
| 0 | | 20/2/203 | | | |
| 0 | | 20/2/203 | | | |
| 1 | 0 | 20/2/203 | 0 | 21/02/2013 | 0 |
| 1 | 0 | 20/2/203 | 0 | 21/02/2012 | 0 |
| 1 | 0 | 20/2/203 | 0 | 21/02/2012 | 0 |
| 1 | 0 | 20/2/203 | 0 | 21/02/2012 | 0 |
| 1 | 0 | 20/2/203 | 0 | 21/02/2012 | 0 |
| 1 | 0 | 20/2/203 | 0 | 21/02/2012 | 0 |
| 1 | 0 | 20/2/203 | 0 | 21/02/2012 | 0 |
| 0 | | 20/2/203 | | | |
| 1 | 0 | 20/2/203 | 0 | 21/02/2012 | 0 |
| 1 | 0 | 20/2/203 | 0 | 21/02/2012 | 0 |
| 1 | 0 | 20/2/203 | 0 | 21/02/2012 | 0 |
| 1 | 0 | 20/2/203 | 0 | 21/02/2012 | 0 |
| 1 | 0 | 20/2/2013 | 0 | 21/02/2012 | 0 |
| 1 | 0 | 20/2/2013 | 0 | 21/02/2012 | 0 |
| 1 | 0 | 20/2/2013 | 0 | 21/02/2012 | 0 |
| 1 | 0 | 20/2/2013 | 0 | 21/02/2013 | 0 |
| 1 | 0 | 20/2/2013 | 0 | 21/02/2012 | 0 |
| 0 | | 20/2/2013 | | | |
| 1 | 0 | 20/2/2013 | 0 | 21/02/2013 | 0 |

| | | | | |
|---|---|---|---|---|
| 1 | 2 | 20/2/2013 | 4 | 21/02/2013 | 10 |
| 0 | | 20/2/2013 | | | |
| 1 | 0 | 20/2/2013 | 0 | 21/02/2013 | 0 |
| 1 | 0 | 20/2/2013 | 0 | 21/02/2012 | 0 |
| 1 | 0 | 20/2/2013 | 0 | 21/02/2012 | 0 |
| 1 | 0 | 20/2/2013 | 0 | 21/02/2012 | 0 |
| 1 | 0 | 20/2/2013 | 0 | 21/02/2012 | 0 |
| 0 | | 20/2/2013 | | | |
| 1 | 0 | 20/2/2013 | 0 | 21/02/2012 | 0 |
| 1 | 0 | 20/2/2013 | 0 | 21/02/2012 | 0 |
| 1 | 1 | 20/2/2013 | 0 | 21/02/2013 | 0 |
| 1 | 0 | 20/2/2013 | 0 | 21/02/2012 | 0 |
| 1 | 0 | 20/2/2013 | 0 | 21/02/2012 | 0 |
| 1 | 0 | 20/2/2013 | 0 | 21/02/2013 | 0 |
| 1 | 0 | 20/2/2013 | 0 | 21/02/2012 | 0 |
| 1 | 0 | 20/2/2013 | 0 | 21/02/2013 | |
| 1 | 0 | 20/2/2013 | 0 | 21/02/2012 | 0 |
| 1 | 0 | 20/2/2013 | 0 | 21/02/2013 | 0 |
| 1 | 0 | 20/2/2013 | 0 | 21/02/2013 | 0 |
| 1 | 0 | 20/2/2013 | 0 | 21/02/2013 | 0 |
| 1 | 0 | 20/2/2013 | 0 | 21/02/2013 | 0 |
| 1 | 0 | 20/2/2013 | 0 | 21/02/2012 | 0 |
| 1 | 0 | 20/2/2013 | 0 | 21/02/2012 | 0 |
| 1 | 0 | 20/2/2013 | 0 | 21/02/2013 | 0 |
| 1 | 0 | 20/2/2013 | 0 | 21/02/2012 | 0 |
| 1 | 0 | 20/2/2013 | 0 | 21/02/2012 | 0 |
| 1 | 0 | 20/2/2013 | 0 | 21/02/2013 | 0 |
| 1 | 0 | 20/2/2013 | 0 | 21/02/2013 | 0 |
| 1 | 0 | 20/2/2013 | 0 | 21/02/2013 | |
| 1 | 0 | 20/2/2013 | 0 | 21/02/2012 | 0 |
| 1 | 0 | 20/2/2013 | 0 | 21/02/2013 | 0 |
| 1 | 0 | 20/2/2013 | 0 | 21/02/2012 | 0 |
| 1 | 0 | 20/2/2013 | 0 | 21/02/2012 | 0 |
| 1 | 0 | 20/2/2013 | 0 | 21/02/2013 | 0 |
| 1 | 0 | 20/2/2013 | 0 | 21/02/2013 | 0 |
| 1 | 0 | 20/2/2013 | 0 | 21/02/2012 | 0 |
| 1 | 0 | 20/2/2013 | 0 | 21/02/2013 | 0 |
| 1 | 0 | 20/2/2013 | 0 | 21/02/2012 | 0 |
| 1 | 0 | 20/2/2013 | 0 | 21/02/2013 | 0 |
| 1 | 0 | 20/2/2013 | 0 | 21/02/2012 | 0 |
| 1 | 0 | 20/2/2013 | 0 | 21/02/2013 | 0 |
| 1 | 0 | 20/2/2013 | 0 | 21/02/2013 | |
| 0 | | 20/2/2013 | | | |
| 0 | 0 | 20/2/2013 | 0 | 21/02/2013 | 0 |
| 0 | | 20/2/2013 | | | |
| 1 | 0 | 20/2/2013 | 0 | 21/02/2012 | 0 |
| 0 | | 20/2/2013 | | | |
| 1 | 0 | 20/2/2013 | 0 | 21/02/2012 | MISSING |
| 0 | | 20/2/2013 | | | |
| 1 | 0 | 20/2/2013 | 0 | 21/02/2012 | |

| | | | | | |
|---|---|---|---|---|---|
| 1 | 0 | 20/2/2013 | 0 | 21/02/2012 | 0 |
| 0 | | 20/2/2013 | | | |
| 1 | 0 | 20/2/2013 | 0 | 21/02/2012 | 0 |
| 0 | | 20/2/2013 | | | |
| 0 | | 20/2/2013 | | | |
| 1 | 0 | 20/2/2013 | 0 | 21/02/2012 | 0 |
| 1 | 0 | 20/2/2013 | 0 | 21/02/2012 | 0 |
| 1 | 0 | 20/2/2013 | 0 | 21/02/2013 | 0 |
| 0 | | 20/2/2013 | | | |
| 1 | 0 | 20/2/2013 | 0 | 21/02/2013 | |
| 1 | 0 | 20/2/2013 | 0 | 21/02/2013 | 0 |
| 1 | 0 | 20/2/2013 | 0 | 21/02/2013 | 0 |
| 1 | 0 | 20/2/2013 | 0 | 21/02/2013 | 0 |
| 1 | 0 | 20/2/2013 | 0 | 21/02/2012 | 0 |
| 1 | 0 | 20/2/2013 | 0 | 21/02/2012 | 0 |
| 1 | 0 | 20/2/2013 | 0 | 21/02/2013 | 0 |
| 1 | 0 | 20/2/2013 | 0 | 21/02/2013 | 0 |
| 1 | 0 | 20/2/2013 | 0 | 21/02/2012 | 0 |
| 1 | 0 | 20/2/2013 | 0 | 21/02/2012 | |
| 0 | | 20/2/2013 | | | |
| 0 | | 20/2/2013 | | | |
| 1 | 0 | 20/2/2013 | 0 | 21/02/2012 | |
| 1 | 0 | 20/2/2013 | 0 | 21/02/2013 | |
| 1 | 0 | 20/2/2013 | 0 | 21/02/2013 | |
| 1 | 0 | 20/2/2013 | 0 | 21/02/2012 | |
| 1 | 0 | 20/2/2013 | 0 | 21/02/2012 | |
| 1 | 0 | 20/2/2013 | 0 | 21/02/2012 | |
| 1 | 0 | 20/2/2013 | 0 | 21/02/2012 | 0 |
| 0 | | 20/2/2013 | | | |
| 1 | 0 | 20/2/2013 | 0 | 21/02/2013 | |
| 1 | 0 | 20/2/2013 | 0 | 21/02/2013 | |
| 1 | 0 | 20/2/2013 | 0 | 21/02/2012 | |
| 1 | 0 | 20/2/2013 | 0 | 21/02/2013 | |
| 0 | | 20/2/2013 | | | |
| 0 | | 20/2/2013 | | | |
| 1 | 0 | 20/2/2013 | 0 | 21/02/2012 | |
| 1 | 0 | 20/2/2013 | 0 | 21/02/2012 | |
| 1 | 0 | 20/2/2013 | 0 | 21/02/2012 | |
| 1 | | 20/2/2013 | | | |
| 0 | | 20/2/2013 | | | |
| 0 | | 20/2/2013 | | | |
| 1 | 0 | 20/2/2013 | 0 | 21/02/2012 | |
| 1 | 0 | 20/2/2013 | 0 | 21/02/2013 | |
| 1 | 0 | 20/2/2013 | 0 | 21/02/2013 | |
| 1 | 0 | 20/2/2013 | 0 | 21/02/2013 | |
| 1 | 0 | 20/2/2013 | 0 | 21/02/2013 | |
| 1 | 0 | 20/2/2013 | 0 | 21/02/2012 | |
| 1 | 0 | 20/2/2013 | 0 | 21/02/2012 | |
| 1 | 2 | 21/2/2013 | 117 | 22/2/2043 | 110 |
| 1 | 0 | 21/2/2013 | 0 | 22/2/2043 | 1 |

| | | | | | |
|---|---|---|---|---|---|
| 1 | 0 | 21/2/2013 | 0 | 22/2/2043 | 0 |
| 1 | 0 | 21/2/2013 | 0 | 22/02/2013 | 0 |
| 1 | 0 | 21/2/2013 | 0 | 22/02/2013 | 0 |
| 1 | 0 | 21/2/2013 | 0 | 22/02/2013 | 0 |
| 1 | 0 | 21/2/2013 | 0 | 22/02/2013 | MISSING |
| 1 | 0 | 21/2/2013 | 0 | 22/02/2013 | 0 |
| 1 | 0 | 21/2/2013 | 0 | 22/02/2013 | 0 |
| 1 | 0 | 21/2/2013 | 0 | 22/02/2013 | 0 |
| 1 | 0 | 21/2/2013 | 0 | 22/2/2019 | 0 |
| 1 | 0 | 21/2/2013 | 0 | 22/2/2020 | 0 |
| 1 | 0 | 21/2/2013 | 0 | 22/2/2021 | 0 |
| 1 | 0 | 21/2/2013 | 0 | 22/02/2013 | 0 |
| 1 | 0 | 21/2/2013 | 0 | 22/02/2013 | 0 |
| 1 | 0 | 21/2/2013 | 0 | 22/02/2013 | 0 |
| 1 | 0 | 21/2/2013 | 0 | 22/2/2043 | 0 |
| 1 | 0 | 21/2/2013 | 0 | 22/02/2013 | 0 |
| 1 | 0 | 21/2/2013 | 0 | 22/02/2013 | MISSING |
| 1 | 0 | 21/2/2013 | 0 | 22/02/2013 | |
| 1 | 0 | 21/2/2013 | 1 | 22/2/2043 | |
| 1 | 0 | 21/2/2013 | 0 | 22/2/2043 | MISSING |
| 1 | 0 | 21/2/2013 | 0 | 22/02/2013 | 0 |
| 1 | 0 | 21/2/2013 | 0 | 22/02/2013 | 0 |
| 1 | 0 | 21/2/2013 | 0 | 22/02/2013 | 0 |
| 1 | 0 | 21/2/2013 | 0 | 22/02/2013 | 0 |
| 1 | 0 | 21/2/2013 | 0 | 22/02/2013 | 0 |
| 1 | 0 | 21/2/2013 | 0 | 22/02/2013 | 0 |
| 1 | 2 | 21/2/2013 | 311 | 22/2/2043 | 703 |
| 1 | 0 | 21/2/2013 | 0 | 22/02/2013 | 0 |
| 1 | 0 | 21/2/2013 | 0 | 22/02/2013 | 0 |
| 1 | 0 | 21/2/2013 | 0 | 22/02/2013 | 0 |
| 1 | 0 | 21/2/2013 | 0 | 22/2/2024 | 0 |
| 1 | 0 | 21/2/2013 | 0 | 22/2/2025 | 0 |
| 1 | 0 | 21/2/2013 | 0 | 22/2/2026 | 0 |
| 1 | 0 | 21/2/2013 | 0 | 22/2/2023 | 0 |
| 1 | 3 | 21/2/2013 | 0 | 22/2/2043 | 0 |
| 1 | 0 | 21/2/2013 | 0 | 22/2/2022 | 0 |
| 1 | 0 | 21/2/2013 | 0 | 22/02/2013 | 0 |
| 1 | 0 | 21/2/2013 | 0 | 22/02/2013 | MISSING |
| 1 | 0 | 21/2/2013 | 0 | 22/2/2043 | 0 |
| 1 | 0 | 21/2/2013 | 0 | 22/2/2043 | 0 |
| 1 | 0 | 21/2/2013 | 0 | 22/2/2043 | 0 |
| 1 | 1 | 21/2/2013 | 1 | 22/2/2043 | 1 |
| 1 | 0 | 21/2/2013 | 0 | 22/2/2043 | 0 |
| 1 | 0 | 21/2/2013 | 0 | 22/2/2043 | 0 |
| 1 | 0 | 21/2/2013 | 0 | 22/02/2013 | 0 |
| 1 | 1 | 21/2/2013 | 0 | 22/2/2014 | 0 |
| 1 | 0 | 21/2/2013 | | | 0 |
| 1 | 0 | 21/2/2013 | 0 | 22/2/2027 | 0 |
| 1 | 0 | 21/2/2013 | 1 | 22/2/2028 | 1 |
| 1 | 0 | 21/2/2013 | 0 | 22/2/2029 | 0 |

| | | | | | |
|---|---|---|---|---|---|
| 1 | 0 | 21/2/2013 | 0 | 22/02/2013 | 0 |
| 1 | 0 | 21/2/2013 | 0 | 22/02/2013 | 0 |
| 1 | 0 | 21/2/2013 | 0 | 22/02/2013 | 0 |
| 1 | 0 | 21/2/2013 | 0 | 22/02/2013 | 0 |
| 1 | 0 | 21/2/2013 | 0 | 22/02/2013 | 0 |
| 1 | 0 | 21/2/2013 | 0 | 22/02/2013 | 0 |
| 1 | 0 | 21/2/2013 | 0 | 22/2/2043 | 0 |
| 1 | 0 | 21/2/2013 | 0 | 22/2/2043 | 0 |
| 1 | 0 | 21/2/2013 | 0 | 22/2/2043 | 0 |
| 1 | 0 | 21/2/2013 | 0 | 22/2/2043 | 0 |
| 1 | 0 | 21/2/2013 | 0 | 22/2/2043 | 0 |
| 1 | 0 | 21/2/2013 | 0 | 22/2/2043 | 0 |
| 1 | 0 | 21/2/2013 | 0 | 22/2/2043 | 0 |
| 1 | 0 | 21/2/2013 | 0 | 22/02/2013 | 0 |
| 1 | 0 | 21/2/2013 | 0 | 22/2/2013 | 0 |
| 0 | | 21/2/2013 | | | |
| 1 | 0 | 21/2/2013 | 0 | 22/2/2043 | 0 |
| 1 | 1 | 21/2/2013 | 0 | 22/2/2043 | 0 |
| 1 | 0 | 21/2/2013 | 0 | 22/2/2043 | 0 |
| 0 | | 21/2/2013 | | | |
| 1 | 0 | 21/2/2013 | 0 | 22/2/2043 | 0 |
| 1 | 0 | 21/2/2013 | 0 | 22/2/2043 | |
| 1 | 0 | 21/2/2013 | 0 | 22/2/2030 | 0 |
| 0 | | 21/2/2013 | | | |
| 1 | 0 | 21/2/2013 | 0 | 22/2/2043 | 0 |
| 1 | 0 | 21/2/2013 | 0 | 22/2/2041 | 0 |
| 1 | 0 | 21/2/2013 | 0 | 22/2/2031 | |
| 1 | 0 | 21/2/2013 | 0 | 22/02/2013 | 0 |
| 1 | 0 | 21/2/2013 | 0 | 22/2/2015 | 0 |
| 1 | 0 | 21/2/2013 | 0 | 22/2/2016 | 0 |
| 1 | 0 | 21/2/2013 | 0 | 22/2/2017 | 0 |
| 1 | 0 | 21/2/2013 | 0 | 22/2/2018 | 0 |
| 1 | 0 | 21/2/2013 | 0 | 22/02/2013 | 0 |
| 1 | 0 | 21/2/2013 | 0 | 22/02/2013 | 0 |
| 1 | 0 | 21/2/2013 | 0 | 22/02/2013 | 0 |
| 1 | 0 | 21/2/2013 | 0 | 22/02/2013 | 0 |
| 1 | 0 | 21/2/2013 | 0 | 22/2/2032 | 0 |
| 1 | 0 | 21/2/2013 | 0 | 22/02/2013 | 0 |
| 1 | 0 | 21/2/2013 | 0 | 22/02/2013 | 0 |
| 1 | 0 | 21/2/2013 | 0 | 22/02/2013 | 0 |
| 1 | 0 | 21/2/2013 | 0 | 22/2/2043 | 0 |
| 1 | 0 | 21/2/2013 | 0 | 22/2/2035 | 0 |
| 1 | 0 | 21/2/2013 | 0 | 22/2/2037 | 0 |
| 1 | 0 | 21/2/2013 | 0 | 22/2/2038 | 0 |
| 1 | 0 | 21/2/2013 | 0 | 22/2/2040 | 0 |
| 1 | 0 | 21/2/2013 | 0 | 22/2/2043 | 0 |
| 1 | 0 | 21/2/2013 | 0 | 22/2/2034 | |
| 1 | 0 | 21/2/2013 | 0 | 22/2/2033 | 0 |
| 1 | 0 | 21/2/2013 | 1 | 22/02/2013 | 0 |
| 1 | 0 | 21/2/2013 | 0 | 22/02/2013 | |

| | | | | | |
|---|---|---|---|---|---|
| 1 | 0 | 21/2/2013 | 0 | 22/02/2013 | |
| 1 | 0 | 21/2/2013 | 0 | 22/02/2013 | |
| 1 | 0 | 21/2/2013 | 0 | 22/02/2013 | |
| 1 | 0 | 21/2/2013 | 0 | 22/02/2013 | |
| 1 | 0 | 21/2/2013 | 0 | 22/02/2013 | |
| 1 | 0 | 21/2/2013 | 0 | 22/02/2013 | |
| 1 | 0 | 21/2/2013 | 0 | 22/2/2043 | |
| 1 | 0 | 21/2/2013 | 0 | 22/2/2036 | |
| 1 | 0 | 21/2/2013 | 0 | 22/2/2039 | |
| 1 | 0 | 21/2/2013 | 0 | 22/2/2043 | |
| 1 | 0 | 21/2/2013 | 0 | 22/2/2042 | |
| 1 | 0 | 21/2/2013 | 0 | 22/2/2043 | |
| 0 | | 21/2/2013 | | | |
| 1 | 0 | 21/2/2013 | 0 | 22/2/2043 | |
| 1 | 0 | 21/2/2013 | 0 | 22/2/2043 | |
| 1 | 0 | 21/2/2013 | 0 | 22/2/2043 | |
| 1 | 0 | 21/2/2013 | 0 | 22/2/2043 | |
| 0 | | 21/2/2013 | | | |
| 1 | 0 | 21/2/2013 | 0 | 22/2/2043 | |
| 1 | 0 | 21/2/2013 | 0 | 22/2/2043 | |
| 1 | 0 | 21/2/2013 | 0 | 22/2/2043 | |
| 1 | 0 | 21/2/2013 | 0 | 22/2/2043 | |
| 1 | 1 | 21/2/2013 | 0 | 22/2/2043 | 0 |
| 0 | | 21/2/2013 | | | |
| 1 | 0 | 21/2/2013 | 0 | 22/02/2013 | |
| 1 | 0 | 21/2/2013 | 0 | 22/02/2013 | |
| 1 | 0 | 21/2/2013 | 0 | 22/2/2043 | |
| 1 | 0 | 21/2/2013 | 0 | 22/2/2043 | |
| 1 | 0 | 22/2/2013 | 0 | 25/2/2013 | 0 |
| 1 | 0 | 22/2/2013 | 0 | 25/2/2013 | 0 |
| 1 | 0 | 22/2/2013 | 0 | 25/2/2013 | 0 |
| 1 | 0 | 22/2/2013 | 0 | 25/2/2013 | 0 |
| 1 | 0 | 22/2/2013 | 0 | 25/2/2013 | 0 |
| 1 | 4 | 22/2/2013 | 0 | 25/2/2013 | 0 |
| 1 | 0 | 22/2/2013 | 0 | 25/2/2013 | 0 |
| 1 | 0 | 22/2/2013 | 0 | 25/2/2013 | 0 |
| 1 | 0 | 22/2/2013 | 0 | 25/2/2013 | 0 |
| 1 | 0 | 22/2/2013 | 0 | 25/2/2013 | 0 |
| 1 | 0 | 22/2/2013 | 0 | 25/2/2013 | 0 |
| 1 | 0 | 22/2/2013 | 0 | 25/2/2013 | 0 |
| 1 | 0 | 22/2/2013 | 0 | 25/2/2013 | 0 |
| 1 | 0 | 22/2/2013 | 0 | 25/2/2013 | 0 |
| 1 | 0 | 22/2/2013 | 0 | 25/2/2013 | 0 |
| 0 | | 22/2/2013 | | | |
| 1 | 0 | 22/2/2013 | 0 | 25/2/2013 | 0 |
| 1 | 0 | 22/2/2013 | 0 | 25/2/2013 | 0 |
| 1 | 0 | 22/2/2013 | 0 | 25/2/2013 | 0 |
| 0 | | 22/2/2013 | | | |
| 1 | 0 | 22/2/2013 | 0 | 25/2/2013 | 0 |
| 1 | 0 | 22/2/2013 | 0 | 25/2/2013 | |

| | | | | | |
|---|---|---|---|---|---|
| 1 | 0 | 22/2/2013 | 0 | 25/2/2013 | 0 |
| 1 | 0 | 22/2/2013 | 0 | 25/2/2013 | 0 |
| 1 | 0 | 22/2/2013 | 0 | 25/2/2013 | 0 |
| 1 | 0 | 22/2/2013 | 0 | 25/2/2013 | 0 |
| 1 | 0 | 22/2/2013 | 0 | 25/2/2013 | 0 |
| 1 | 0 | 22/2/2013 | 0 | 25/2/2013 | 0 |
| 1 | 0 | 22/2/2013 | 0 | 25/2/2013 | 0 |
| 1 | 0 | 22/2/2013 | 0 | 25/2/2013 | 0 |
| 1 | 0 | 22/2/2013 | 0 | 25/2/2013 | 0 |
| 1 | 0 | 22/2/2013 | 0 | 25/2/2013 | 0 |
| 1 | 2 | 22/2/2013 | 0 | 25/2/2013 | 1 |
| 1 | 0 | 22/2/2013 | 0 | 25/2/2013 | 0 |
| 1 | 0 | 22/2/2013 | 0 | 25/2/2013 | 0 |
| 1 | 0 | 22/2/2013 | 0 | 25/2/2013 | 0 |
| 1 | 0 | 22/2/2013 | 0 | 25/2/2013 | 0 |
| 1 | 0 | 22/2/2013 | 0 | 25/2/2013 | 0 |
| 1 | 0 | 22/2/2013 | 0 | 25/2/2013 | 0 |
| 0 | | 22/2/2013 | | | |
| 1 | 0 | 22/2/2013 | 0 | 25/2/2013 | 0 |
| 1 | 0 | 22/2/2013 | 0 | 25/2/2013 | 0 |
| 1 | 0 | 22/2/2013 | 0 | 25/2/2013 | 0 |
| 1 | 0 | 22/2/2013 | 0 | 25/2/2013 | 0 |
| 1 | 0 | 22/2/2013 | 0 | 25/2/2013 | 0 |
| 1 | 0 | 22/2/2013 | 0 | 25/2/2013 | 0 |
| 1 | 0 | 22/2/2013 | 0 | 25/2/2013 | 0 |
| 1 | 0 | 22/2/2013 | 0 | 25/2/2013 | 0 |
| 1 | 0 | 22/2/2013 | 0 | 25/2/2013 | 0 |
| 1 | 0 | 22/2/2013 | 0 | 25/2/2013 | 0 |
| 1 | 0 | 22/2/2013 | | | 0 |
| 1 | 0 | 22/2/2013 | 0 | 25/2/2013 | 0 |
| 1 | 0 | 22/2/2013 | 0 | 25/2/2013 | 0 |
| 1 | 0 | 22/2/2013 | 0 | 25/2/2013 | 0 |
| 1 | 0 | 22/2/2013 | 0 | 25/2/2013 | 0 |
| 1 | 0 | 22/2/2013 | 0 | 25/2/2013 | 0 |
| 1 | 0 | 22/2/2013 | 0 | 25/2/2013 | 0 |
| 1 | 0 | 22/2/2013 | 0 | 25/2/2013 | 0 |
| 1 | 0 | 22/2/2013 | 0 | 25/2/2013 | 0 |
| 1 | 0 | 22/2/2013 | 0 | 25/2/2013 | |
| 1 | 0 | 22/2/2013 | 0 | 25/2/2013 | 0 |
| 1 | 0 | 22/2/2013 | 0 | 25/2/2013 | 0 |
| 1 | 0 | 22/2/2013 | 0 | 25/2/2013 | 0 |
| 1 | 0 | 22/2/2013 | 0 | 25/2/2013 | 0 |
| 1 | 0 | 22/2/2013 | 0 | 25/2/2013 | 0 |
| 0 | | 22/2/2013 | | | |
| 0 | | 22/2/2013 | | | |
| 0 | | 22/2/2013 | | | |
| 0 | | 22/2/2013 | | | |
| 1 | 0 | 22/2/2013 | 0 | 25/2/2013 | 0 |
| 1 | 0 | 22/2/2013 | 0 | 25/2/2013 | 0 |
| 1 | 0 | 22/2/2013 | 2 | 25/2/2013 | 5 |

| | | | | | | |
|---|---|---|---|---|---|---|
| 1 | 0 | 22/2/2013 | 0 | 25/2/2013 | 0 |
| 1 | 0 | 22/2/2013 | 0 | 25/2/2013 | 0 |
| 1 | 0 | 22/2/2013 | 0 | 25/2/2013 | 0 |
| 0 | | 22/2/2013 | | | |
| 1 | 0 | 22/2/2013 | 0 | 25/2/2013 | 0 |
| 0 | | 22/2/2013 | | | |
| 1 | 0 | 22/2/2013 | 0 | 25/2/2013 | 0 |
| 1 | 0 | 22/2/2013 | 0 | 25/2/2013 | 0 |
| 0 | | 22/2/2013 | | | |
| 1 | 0 | 22/2/2013 | 0 | 25/2/2013 | 0 |
| 0 | | 22/2/2013 | | | |
| 0 | | 22/2/2013 | | | |
| 0 | | 22/2/2013 | | | |
| 1 | 0 | 22/2/2013 | 0 | 25/2/2013 | 0 |
| 1 | 0 | 22/2/2013 | 0 | 25/2/2013 | 0 |
| 0 | | 22/2/2013 | | | |
| 1 | 0 | 22/2/2013 | 0 | 25/2/2013 | 0 |
| 0 | | 22/2/2013 | | | |
| 0 | | 22/2/2013 | | | |
| 1 | 0 | 22/2/2013 | 0 | 25/2/2013 | 0 |
| 1 | 0 | 22/2/2013 | 0 | 25/2/2013 | 0 |
| 1 | 0 | 22/2/2013 | 0 | 25/2/2013 | 0 |
| 1 | 0 | 22/2/2013 | 0 | 25/2/2013 | 0 |
| 1 | 0 | 22/2/2013 | 0 | 25/2/2013 | 0 |
| 1 | 3 | 22/2/2013 | 0 | 25/2/2013 | 0 |
| 1 | 0 | 22/2/2013 | 0 | 25/2/2013 | 0 |
| 1 | 0 | 22/2/2013 | 0 | 25/2/2013 | 0 |
| 1 | 0 | 22/2/2013 | 0 | 25/2/2013 | 0 |
| 1 | 0 | 22/2/2013 | 0 | 25/2/2013 | |
| 1 | 0 | 22/2/2013 | 0 | 25/2/2013 | 0 |
| 1 | 0 | 22/2/2013 | 0 | 25/2/2013 | |
| 1 | 0 | 22/2/2013 | 0 | 25/2/2013 | |
| 1 | 0 | 22/2/2013 | 0 | 25/2/2013 | |
| 1 | 0 | 22/2/2013 | 0 | 25/2/2013 | |
| 0 | | 22/2/2013 | | | |
| 0 | | 22/2/2013 | | | |
| 1 | 0 | 22/2/2013 | 0 | 25/2/2013 | 0 |
| 1 | 0 | 22/2/2013 | 0 | 25/2/2013 | |
| 1 | 0 | 22/2/2013 | 0 | 25/2/2013 | |
| 0 | | 22/2/2013 | | | |
| 1 | 0 | 22/2/2013 | 0 | 25/2/2013 | |
| 1 | 0 | 22/2/2013 | 0 | 25/2/2013 | |
| 0 | | 22/2/2013 | | | |
| 1 | 0 | 22/2/2013 | 0 | 25/2/2013 | |
| 1 | 0 | 22/2/2013 | 0 | 25/2/2013 | |
| 1 | 0 | 22/2/2013 | 1 | 25/2/2013 | |
| 1 | 0 | 22/2/2013 | 0 | 25/2/2013 | |
| 1 | 0 | 22/2/2013 | 0 | 25/2/2013 | |
| 1 | 0 | 22/2/2013 | 0 | 25/2/2013 | |
| 1 | 0 | 22/2/2013 | 0 | 25/2/2013 | |

| | | | | | |
|---|---|---|---|---|---|
| 1 | 0 | 22/2/2013 | 0 | 25/2/2013 | |
| 1 | 1 | 22/2/2013 | 0 | 25/2/2013 | |
| 1 | 0 | 22/2/2013 | 0 | 25/2/2013 | |
| 1 | 0 | 22/2/2013 | 0 | 25/2/2013 | |
| 1 | 0 | 22/2/2013 | 0 | 25/2/2013 | |
| 1 | 0 | 22/2/2013 | 0 | 25/2/2013 | |
| 1 | 0 | 22/2/2013 | 0 | 25/2/2013 | |
| 1 | 0 | 22/2/2013 | 0 | 25/2/2013 | |
| 1 | 0 | 03/05/2013 | 0 | 03/05/2013 | 0 |
| 1 | 0 | 03/05/2013 | 0 | 03/05/2013 | 0 |
| 1 | 0 | 03/05/2013 | 0 | 03/05/2013 | 0 |
| 1 | 0 | 03/05/2013 | 0 | 03/05/2013 | 0 |
| 1 | 0 | 03/05/2013 | 0 | 03/05/2013 | 0 |
| 1 | 0 | 03/05/2013 | 0 | 03/05/2013 | 0 |
| 1 | 0 | 03/05/2013 | 0 | 03/05/2013 | 0 |
| 1 | 0 | 03/05/2013 | 0 | 03/05/2013 | 0 |
| 1 | 0 | 03/05/2013 | 0 | 03/05/2013 | 0 |
| 1 | 0 | 03/05/2013 | 0 | 03/05/2013 | 0 |
| 1 | 0 | 03/05/2013 | 0 | 03/05/2013 | 0 |
| 1 | 0 | 03/05/2013 | 0 | 03/05/2013 | 0 |
| 1 | 0 | 03/05/2013 | 0 | 03/05/2013 | 0 |
| 1 | 0 | 03/05/2013 | 0 | 03/05/2013 | 0 |
| 1 | 0 | 03/05/2013 | 0 | 03/05/2013 | 0 |
| 1 | 0 | 03/05/2013 | 0 | 03/05/2013 | 0 |
| 1 | 0 | 03/05/2013 | 0 | 03/05/2013 | 0 |
| 1 | 0 | 03/05/2013 | 0 | 03/05/2013 | 0 |
| 1 | 0 | 03/05/2013 | 0 | 03/05/2013 | 0 |
| 1 | 0 | 03/05/2013 | 0 | 03/05/2013 | 0 |
| 1 | 0 | 03/05/2013 | 0 | 03/05/2013 | 0 |
| 1 | 0 | 03/05/2013 | 0 | 03/05/2013 | 0 |
| 1 | 0 | 03/05/2013 | 0 | 03/05/2013 | |
| 1 | 0 | 03/05/2013 | 0 | 03/05/2013 | 0 |
| 1 | 0 | 03/05/2013 | 0 | 03/05/2013 | 0 |
| 0 | | 03/05/2013 | | | |
| 1 | 0 | 03/05/2013 | 0 | 03/05/2013 | 0 |
| 1 | 0 | 03/05/2013 | 0 | 03/05/2013 | 0 |
| 1 | 0 | 03/05/2013 | 0 | 03/05/2013 | 0 |
| 1 | 0 | 03/05/2013 | 0 | 03/05/2013 | 0 |
| 1 | 0 | 03/05/2013 | 0 | 03/05/2013 | 0 |
| 1 | 0 | 03/05/2013 | 0 | 03/05/2013 | 0 |
| 1 | 1 | 03/05/2013 | 24 | 03/05/2013 | 10 |
| 1 | 0 | 03/05/2013 | 0 | 03/05/2013 | 0 |
| 1 | 0 | 03/05/2013 | 0 | 03/05/2013 | 0 |
| 1 | 0 | 03/05/2013 | 0 | 03/05/2013 | 0 |
| 1 | 0 | 03/05/2013 | 0 | 03/05/2013 | 0 |
| 1 | 0 | 03/05/2013 | 0 | 03/05/2013 | 0 |
| 1 | 0 | 03/05/2013 | 0 | 03/05/2013 | 0 |
| 1 | 0 | 05.03.2013 | 0 | 03/05/2013 | 0 |
| 1 | 3 | 05.03.2013 | 86 | 05.03.2013 | 96 |
| 1 | 0 | 03/05/2013 | 0 | 03/05/2013 | 0 |

| | | | | | |
|---|---|---|---|---|---|
| 1 | 0 | 03/05/2013 | 0 | 03/05/2013 | 0 |
| 1 | 0 | 03/05/2013 | 0 | 03/05/2013 | 0 |
| 1 | 0 | 03/05/2013 | 0 | 03/05/2013 | 0 |
| 1 | 0 | 03/05/2013 | 0 | 03/05/2013 | 0 |
| 1 | 0 | 03/05/2013 | 0 | 03/05/2013 | 0 |
| 1 | 0 | 03/05/2013 | 0 | 03/05/2013 | 0 |
| 1 | 0 | 03/05/2013 | 0 | 03/05/2013 | 0 |
| 1 | 0 | 03/05/2013 | 0 | 03/05/2013 | 0 |
| 1 | 0 | 03/05/2013 | 0 | 03/05/2013 | 0 |
| 1 | 0 | 03/05/2013 | 0 | 03/05/2013 | 0 |
| 1 | 0 | 03/05/2013 | 0 | 03/05/2013 | 0 |
| 1 | 0 | 03/05/2013 | 0 | 03/05/2013 | 0 |
| 1 | 0 | 03/05/2013 | 0 | 03/05/2013 | 0 |
| 1 | 0 | 03/05/2013 | 0 | 03/05/2013 | 0 |
| 1 | 0 | 03/05/2013 | 0 | 03/05/2013 | 0 |
| 1 | 0 | 03/05/2013 | 0 | 03/05/2013 | 0 |
| 1 | 0 | 03/05/2013 | 0 | 03/05/2013 | 0 |
| 1 | 0 | 03/05/2013 | 0 | 03/05/2013 | 0 |
| 1 | 0 | 03/05/2013 | 0 | 03/05/2013 | 0 |
| 1 | 0 | 03/05/2013 | 0 | 03/05/2013 | 0 |
| 1 | 0 | 03/05/2013 | 0 | 03/05/2013 | 0 |
| 1 | 0 | 03/05/2013 | 0 | 03/05/2013 | 0 |
| 1 | 0 | 03/05/2013 | 0 | 03/05/2013 | 0 |
| 1 | 0 | 03/05/2013 | 0 | 03/05/2013 | 0 |
| 1 | 0 | 03/05/2013 | 0 | 03/05/2013 | 0 |
| 1 | 0 | 03/05/2013 | 0 | 03/05/2013 | 0 |
| 1 | 0 | 03/05/2013 | 0 | 03/05/2013 | 0 |
| 1 | 0 | 03/05/2013 | 0 | 03/05/2013 | 0 |
| 1 | 0 | 03/05/2013 | 0 | 03/05/2013 | 0 |
| 1 | 0 | 03/05/2013 | 0 | 03/05/2013 | 0 |
| 1 | 0 | 03/05/2013 | 0 | 03/05/2013 | 0 |
| 1 | 0 | 03/05/2013 | 0 | 03/05/2013 | 0 |
| 1 | 0 | 03/05/2013 | 0 | 03/05/2013 | 0 |
| 1 | 0 | 03/05/2013 | 0 | 03/05/2013 | 0 |
| 1 | 0 | 03/05/2013 | 0 | 03/05/2013 | 0 |
| 1 | 0 | 03/05/2013 | 0 | 03/05/2013 | 0 |
| 1 | 0 | 03/05/2013 | 0 | 03/05/2013 | 0 |
| 1 | 0 | 03/05/2013 | 0 | 03/05/2013 | 0 |
| 1 | 0 | 03/05/2013 | 0 | 03/05/2013 | |
| 1 | 0 | 03/05/2013 | 0 | 03/05/2013 | 0 |
| 0 | | 03/05/2013 | | | |
| 1 | 0 | 03/05/2013 | 0 | 03/05/2013 | 0 |
| 1 | 0 | 03/05/2013 | 0 | 03/05/2013 | 0 |
| 1 | 0 | 03/05/2013 | 0 | 03/05/2013 | 0 |
| 1 | 0 | 03/05/2013 | 0 | 03/05/2013 | 0 |
| 0 | | 03/05/2013 | | | |
| 1 | 0 | 03/05/2013 | 0 | 03/05/2013 | 0 |
| 1 | 0 | 03/05/2013 | 0 | 03/05/2013 | 0 |
| 1 | 0 | 03/05/2013 | 0 | 03/05/2013 | 0 |
| 1 | 0 | 03/05/2013 | 0 | 03/05/2013 | 0 |

| | | | | | |
|---|---|---|---|---|---|
| 1 | 0 | 03/05/2013 | 0 | 03/05/2013 | 0 |
| 1 | 0 | 03/05/2013 | 0 | 03/05/2013 | 0 |
| 1 | 0 | 03/05/2013 | 0 | 03/05/2013 | 0 |
| 1 | 0 | 03/05/2013 | 0 | 03/05/2013 | 0 |
| 1 | 0 | 03/05/2013 | 0 | 03/05/2013 | 0 |
| 1 | 0 | 03/05/2013 | 0 | 03/05/2013 | 0 |
| 1 | 0 | 03/05/2013 | 0 | 03/05/2013 | 0 |
| 1 | 0 | 03/05/2013 | 0 | 03/05/2013 | 0 |
| 1 | 0 | 03/05/2013 | 0 | 03/05/2013 | |
| 1 | 0 | 03/05/2013 | 0 | 03/05/2013 | |
| 1 | 0 | 03/05/2013 | 0 | 03/05/2013 | |
| 1 | 0 | 03/05/2013 | 0 | 03/05/2013 | 0 |
| 1 | 0 | 03/05/2013 | 0 | 03/05/2013 | |
| 1 | 0 | 03/05/2013 | 0 | 03/05/2013 | |
| 1 | 0 | 03/05/2013 | 0 | 03/05/2013 | |
| 1 | 0 | 03/05/2013 | 0 | 03/05/2013 | |
| 1 | 0 | 03/05/2013 | 0 | 03/05/2013 | |
| 1 | 0 | 03/05/2013 | 0 | 03/05/2013 | |
| 1 | 0 | 03/05/2013 | 0 | 03/05/2013 | |
| 1 | 0 | 03/05/2013 | 0 | 03/05/2013 | |
| 1 | 0 | 03/05/2013 | 0 | 03/05/2013 | |
| 0 | | 03/05/2013 | | | |
| 1 | 0 | 03/05/2013 | 0 | 03/05/2013 | |
| 1 | 0 | 03/05/2013 | 0 | 03/05/2013 | |
| 1 | 0 | 03/05/2013 | 0 | 03/05/2013 | |
| 1 | 0 | 03/05/2013 | 0 | 03/05/2013 | |
| 1 | 0 | 03/05/2013 | 0 | 03/05/2013 | |
| 1 | 0 | 03/05/2013 | 0 | 03/05/2013 | |
| 1 | 0 | 03/05/2013 | 0 | 03/05/2013 | |
| 0 | | 03/05/2013 | | | |
| 1 | 0 | 03/05/2013 | 0 | 03/05/2013 | |
| 1 | 0 | 03/05/2013 | 0 | 03/05/2013 | 0 |
| 1 | 0 | 03/05/2013 | 0 | 03/05/2013 | |
| 1 | 0 | 03/05/2013 | 0 | 03/05/2013 | |
| 1 | 0 | 03/05/2013 | 0 | 03/05/2013 | |
| 1 | 0 | 03/05/2013 | 0 | 03/05/2013 | |
| 1 | 0 | 03/05/2013 | 0 | 03/05/2013 | |
| 1 | 0 | 03/05/2013 | 0 | 03/05/2013 | |
| 1 | 0 | 02/05/2013 | 0 | 02/05/2013 | 0 |
| 1 | 0 | 02/05/2013 | 0 | 02/05/2013 | 0 |
| 0 | | 02/05/2013 | | | |
| 1 | 0 | 02/05/2013 | 0 | 02/05/2013 | 0 |
| 1 | 0 | 02/05/2013 | 0 | 02/05/2013 | 0 |
| 1 | 0 | 02/05/2013 | 0 | 02/05/2013 | 0 |
| 1 | 0 | 02/05/2013 | 0 | 02/05/2013 | 0 |
| 1 | 0 | 02/05/2013 | 0 | 02/05/2013 | 0 |
| 1 | 0 | 02/05/2013 | 0 | 02/05/2013 | 0 |
| 1 | 0 | 02/05/2013 | | | 0 |
| 1 | 0 | 02/05/2013 | 0 | 02/05/2013 | 0 |
| 1 | 0 | 02/05/2013 | 0 | 02/05/2013 | 0 |

| | | | | | |
|---|---|---|---|---|---|
| 1 | 0 | 02/05/2013 | 0 | 02/05/2013 | 0 |
| 0 | | 02/05/2013 | | | |
| 1 | 0 | 02/05/2013 | 0 | 02/05/2013 | 0 |
| 1 | 0 | 02/05/2013 | 0 | 02/05/2013 | 0 |
| 1 | 0 | 02/05/2013 | 0 | 02/05/2013 | 0 |
| 1 | 0 | 02/05/2013 | 0 | 02/05/2013 | 0 |
| 1 | 0 | 02/05/2013 | 0 | 02/05/2013 | 0 |
| 1 | 0 | 02/05/2013 | 0 | 02/05/2013 | 0 |
| 1 | 1 | 02/05/2013 | 12 | 02/05/2013 | 18 |
| 1 | 0 | 02/05/2013 | 0 | 02/05/2013 | 0 |
| 1 | | 02/05/2013 | | | |
| 1 | 0 | 02/05/2013 | 0 | 02/05/2013 | 0 |
| 0 | | 02/05/2013 | | | |
| 1 | 0 | 02/05/2013 | 0 | 02/05/2013 | 0 |
| 1 | 0 | 02/05/2013 | 0 | 02/05/2013 | 0 |
| 1 | 0 | 02/05/2013 | 0 | 02/05/2013 | 0 |
| 1 | 0 | 02/05/2013 | 0 | 02/05/2013 | 0 |
| 0 | | 02/05/2013 | | | |
| 1 | 0 | 02/05/2013 | 0 | 02/05/2013 | |
| 1 | 0 | 02/05/2013 | 0 | 02/05/2013 | 0 |
| 0 | | 02/05/2013 | | | |
| 1 | 0 | 02/05/2013 | 0 | 02/05/2013 | 0 |
| 1 | 0 | 02/05/2013 | 0 | 02/05/2013 | 0 |
| 1 | 0 | 02/05/2013 | 0 | 02/05/2013 | 0 |
| 1 | 0 | 02/05/2013 | 0 | 02/05/2013 | 0 |
| 0 | | 02/05/2013 | | | |
| 1 | 0 | 02/05/2013 | 0 | 02/05/2013 | |
| 1 | 0 | 02/05/2013 | 0 | 02/05/2013 | 0 |
| 0 | | 02/05/2013 | | | |
| 0 | | 02/05/2013 | | | |
| 0 | | 02/05/2013 | | | |
| 1 | 0 | 02/05/2013 | 0 | 02/05/2013 | 0 |
| 1 | 0 | 02/05/2013 | 0 | 02/05/2013 | 0 |
| 1 | 0 | 02/05/2013 | 0 | 02/05/2013 | 0 |
| 1 | 0 | 02/05/2013 | 0 | 02/05/2013 | 0 |
| 1 | 0 | 02/05/2013 | 0 | 02/05/2013 | 0 |
| 0 | | 02/05/2013 | | | |
| 1 | 0 | 02/05/2013 | 0 | 02/05/2013 | 0 |
| 0 | | 02/05/2013 | | | |
| 0 | | 02/05/2013 | | | |
| 1 | 0 | 02/05/2013 | 0 | 02/05/2013 | 0 |
| 1 | 0 | 02/05/2013 | 0 | 02/05/2013 | 0 |
| 0 | | 02/05/2013 | | | |
| 1 | 0 | 02/05/2013 | 0 | 02/05/2013 | 0 |
| 0 | | 02/05/2013 | | | |
| 1 | 3 | 02/05/2013 | 0 | 02/05/2013 | 28 |
| 1 | 0 | 02/05/2013 | 0 | 02/05/2013 | 0 |
| 0 | | 02/05/2013 | | | |
| 1 | 0 | 02/05/2013 | 0 | 02/05/2013 | 0 |
| 0 | | 02/05/2013 | | | |

| | | | | | |
|---|---|---|---|---|---|
| 0 | | 02/05/2013 | | | |
| 0 | | 02/05/2013 | | | |
| 0 | | 02/05/2013 | | | |
| 0 | | 02/05/2013 | | | |
| 1 | 0 | 02/05/2013 | 0 | 02/05/2013 | 0 |
| 1 | 0 | 02/05/2013 | 0 | 02/05/2013 | 0 |
| 0 | | 02/05/2013 | | | |
| 1 | 0 | 02/05/2013 | 0 | 02/05/2013 | 0 |
| 1 | 0 | 02/05/2013 | 0 | 02/05/2013 | 0 |
| 1 | 0 | 02/05/2013 | 0 | 02/05/2013 | 0 |
| 1 | 0 | 02/05/2013 | 0 | 02/05/2013 | 0 |
| 0 | | 02/05/2013 | | | |
| 1 | 0 | 02/05/2013 | 0 | 02/05/2013 | 0 |
| 0 | | 02/05/2013 | | | |
| 0 | | 02/05/2013 | | | |
| 1 | 0 | 02/05/2013 | 0 | 02/05/2013 | 0 |
| 1 | 0 | 02/05/2013 | 0 | 02/05/2013 | 0 |
| 1 | 0 | 02/05/2013 | 0 | 02/05/2013 | 0 |
| 0 | | 02/05/2013 | | | |
| 1 | 0 | 02/05/2013 | 0 | 02/05/2013 | 0 |
| 1 | 0 | 02/05/2013 | 0 | 02/05/2013 | 0 |
| 1 | 0 | 02/05/2013 | 0 | 02/05/2013 | 0 |
| 1 | 0 | 02/05/2013 | 0 | 02/05/2013 | 0 |
| 1 | 0 | 02/05/2013 | 0 | 02/05/2013 | 0 |
| 1 | 0 | 02/05/2013 | 0 | 02/05/2013 | 0 |
| 1 | 0 | 02/05/2013 | 0 | 02/05/2013 | 0 |
| 1 | 0 | 02/05/2013 | 0 | 02/05/2013 | 0 |
| 1 | 0 | 02/05/2013 | 0 | 02/05/2013 | 0 |
| 1 | 0 | 02/05/2013 | 0 | 02/05/2013 | 0 |
| 1 | 0 | 02/05/2013 | 0 | 02/05/2013 | 0 |
| 1 | 0 | 02/05/2013 | 0 | 02/05/2013 | 0 |
| 1 | 0 | 02/05/2013 | 0 | 02/05/2013 | 0 |
| 0 | | 02/05/2013 | | | |
| 1 | 0 | 02/05/2013 | 0 | 02/05/2013 | 0 |
| 1 | 0 | 02/05/2013 | 0 | 02/05/2013 | 0 |
| 1 | 0 | 02/05/2013 | 0 | 02/05/2013 | 0 |
| 1 | | 02/05/2013 | | | |
| 1 | 0 | 02/05/2013 | 0 | 02/05/2013 | 0 |
| 1 | 0 | 02/05/2013 | 0 | 02/05/2013 | |
| 1 | 0 | 02/05/2013 | 0 | 02/05/2013 | |
| 0 | | 02/05/2013 | | | |
| 1 | 0 | 02/05/2013 | 0 | 02/05/2013 | |
| 0 | | 02/05/2013 | | | |
| 0 | | 02/05/2013 | | | |
| 1 | 0 | 02/05/2013 | 0 | 02/05/2013 | |
| 0 | | 02/05/2013 | | | |
| 1 | 0 | 02/05/2013 | 0 | 02/05/2013 | |
| 0 | | 02/05/2013 | | | |
| 1 | 0 | 02/05/2013 | 0 | 02/05/2013 | |
| 0 | | 02/05/2013 | | | |

| | | | | | |
|---|---|---|---|---|---|
| 1 | 0 | 02/05/2013 | 0 | 02/05/2013 | |
| 1 | 0 | 02/05/2013 | 0 | 02/05/2013 | |
| 1 | 0 | 02/05/2013 | 0 | 02/05/2013 | 0 |
| 1 | 0 | 02/05/2013 | 0 | 02/05/2013 | |
| 1 | 0 | 02/05/2013 | 0 | 02/05/2013 | |
| 1 | 0 | 02/05/2013 | 0 | 02/05/2013 | |
| 1 | 0 | 02/05/2013 | 0 | 02/05/2013 | |
| 1 | 0 | 02/05/2013 | 0 | 02/05/2013 | |
| 1 | 0 | 02/05/2013 | 0 | 02/05/2013 | |
| 1 | 0 | 02/05/2013 | 0 | 02/05/2013 | |
| 1 | 0 | 02/05/2013 | 0 | 02/05/2013 | |
| 0 | | 02/05/2013 | | | |
| 0 | | 02/05/2013 | | | |
| 1 | 0 | 02/05/2013 | 0 | 02/05/2013 | |
| 1 | 0 | 02/05/2013 | 0 | 02/05/2013 | |
| 1 | 0 | 02/05/2013 | 0 | 02/05/2013 | |
| 0 | | 02/05/2013 | | | |
| 0 | | 02/05/2013 | | | |
| 1 | 0 | 22/2/2013 | 0 | 26/2/2013 | 0 |
| 1 | 0 | 22/2/2013 | 0 | 26/2/2013 | 0 |
| 1 | 0 | 22/2/2013 | 0 | 26/2/2013 | 0 |
| 1 | 0 | 22/2/2013 | 0 | 26/2/2013 | 0 |
| 1 | 0 | 22/2/2013 | 3 | 26/2/2013 | 7 |
| 0 | | 22/2/2013 | | | |
| 1 | 0 | 22/2/2013 | 0 | 26/2/2013 | 0 |
| 1 | | 22/2/2013 | | | |
| 1 | 0 | 22/2/2013 | 0 | 26/2/2013 | 0 |
| 1 | 0 | 22/2/2013 | 0 | 26/2/2013 | 0 |
| 1 | 0 | 22/2/2013 | 0 | 26/2/2013 | 0 |
| 1 | 0 | 22/2/2013 | 0 | 26/2/2013 | 0 |
| 1 | 0 | 22/2/2013 | 0 | 26/2/2013 | 0 |
| 1 | | 22/2/2013 | | | |
| 1 | 0 | 22/2/2013 | 0 | 26/2/2013 | 0 |
| 0 | | 22/2/2013 | | | |
| 1 | 2 | 22/2/2013 | 0 | 26/2/2013 | 0 |
| 0 | | 22/2/2013 | | | |
| 1 | 0 | 22/2/2013 | 0 | 26/2/2013 | 0 |
| 0 | | 22/2/2013 | | | |
| 1 | 0 | 22/2/2013 | 0 | 26/2/2013 | 0 |
| 1 | 0 | 22/2/2013 | 0 | 26/2/2013 | 0 |
| 1 | 0 | 22/2/2013 | 0 | 26/2/2013 | 0 |
| 1 | 0 | 22/2/2013 | 0 | 26/2/2013 | 0 |
| 1 | 0 | 22/2/2013 | 0 | 26/2/2013 | 0 |
| 1 | 0 | 22/2/2013 | 0 | 26/2/2013 | 0 |
| 0 | | 22/2/2013 | | | |
| 1 | | 22/2/2013 | | | |
| 0 | | 22/2/2013 | | | |
| 1 | 0 | 22/2/2013 | 0 | 26/2/2013 | 0 |
| 0 | | 22/2/2013 | | | |
| 1 | 0 | 22/2/2013 | 0 | 26/2/2013 | 0 |

| | | | | | |
|---|---|---|---|---|---|
| 1 | 0 | 22/2/2013 | 0 | 26/2/2013 | 0 |
| 1 | 0 | 22/2/2013 | 0 | 26/2/2013 | 0 |
| 1 | 0 | 22/2/2013 | 0 | 26/2/2013 | 0 |
| 1 | 0 | 22/2/2013 | 0 | 26/2/2013 | 0 |
| 1 | 0 | 22/2/2013 | 0 | 26/2/2013 | 0 |
| 1 | 0 | 22/2/2013 | 0 | 26/2/2013 | 0 |
| 1 | 0 | 22/2/2013 | 0 | 26/2/2013 | 0 |
| 1 | 0 | 22/2/2013 | 0 | 26/2/2013 | 0 |
| 0 | | 22/2/2013 | | | |
| 1 | 2 | 22/2/2013 | 0 | 26/2/2013 | 0 |
| 1 | 0 | 22/2/2013 | 0 | 26/2/2013 | 0 |
| 1 | 0 | 22/2/2013 | 0 | 26/2/2013 | |
| 1 | 0 | 22/2/2013 | 0 | 26/2/2013 | 0 |
| 1 | 0 | 22/2/2013 | 0 | 26/2/2013 | 0 |
| 1 | 0 | 22/2/2013 | 0 | 26/2/2013 | 0 |
| 0 | | 22/2/2013 | | | |
| 0 | | 22/2/2013 | | | |
| 1 | 0 | 22/2/2013 | 2 | 26/2/2013 | 2 |
| 1 | 0 | 22/2/2013 | 0 | 26/2/2013 | 0 |
| 1 | 0 | 22/2/2013 | 0 | 26/2/2013 | 0 |
| 0 | | 22/2/2013 | | | |
| 1 | 0 | 22/2/2013 | 0 | 26/2/2013 | 0 |
| 1 | 0 | 22/2/2013 | 0 | 26/2/2013 | 0 |
| 0 | | 22/2/2013 | | | |
| 1 | 0 | 22/2/2013 | 0 | 26/2/2013 | 0 |
| 0 | | 22/2/2013 | | | |
| 0 | | 22/2/2013 | | | |
| 1 | 0 | 22/2/2013 | 0 | 26/2/2013 | 0 |
| 1 | 0 | 22/2/2013 | 0 | 26/2/2013 | 0 |
| 0 | | 22/2/2013 | | | |
| 1 | 0 | 22/2/2013 | 0 | 26/2/2013 | 0 |
| 1 | 0 | 22/2/2013 | 0 | 26/2/2013 | 0 |
| 0 | | 22/2/2013 | | | |
| 1 | 0 | 22/2/2013 | 0 | 26/2/2013 | 0 |
| 1 | 0 | 22/2/2013 | 0 | 26/2/2013 | 0 |
| 1 | 0 | 22/2/2013 | 0 | 26/2/2013 | 0 |
| 1 | 0 | 22/2/2013 | 0 | 26/2/2013 | 0 |
| 1 | 0 | 22/2/2013 | 1 | 26/2/2013 | 0 |
| 1 | 0 | 22/2/2013 | 0 | 26/2/2013 | 0 |
| 0 | | 22/2/2013 | | | |
| 1 | 0 | 22/2/2013 | 0 | 26/2/2013 | 0 |
| 1 | 0 | 22/2/2013 | 0 | 26/2/2013 | 1 |
| 1 | 0 | 22/2/2013 | 0 | 26/2/2013 | 0 |
| 1 | 0 | 22/2/2013 | 0 | 26/2/2013 | |
| 1 | 4 | 22/2/2013 | 28 | 26/2/2013 | 75 |
| 1 | 0 | 22/2/2013 | 0 | 26/2/2013 | 0 |
| 1 | 0 | 22/2/2013 | 0 | 26/2/2013 | 0 |
| 0 | | 22/2/2013 | | | |
| 1 | 0 | 22/2/2013 | 0 | 26/2/2013 | 0 |
| 1 | 1 | 22/2/2013 | 15 | 26/2/2013 | 26 |

| | | | | | |
|---|---|---|---|---|---|
| 0 | | 22/2/2013 | | | |
| 0 | | 22/2/2013 | | | |
| 0 | | 22/2/2013 | | | |
| 1 | 0 | 22/2/2013 | 0 | 26/2/2013 | 0 |
| 1 | 0 | 22/2/2013 | 0 | 26/2/2013 | 0 |
| 1 | 0 | 22/2/2013 | 0 | 26/2/2013 | 0 |
| 0 | | 22/2/2013 | | | |
| 1 | 0 | 22/2/2013 | 0 | 26/2/2013 | 0 |
| 1 | 0 | 22/2/2013 | 0 | 26/2/2013 | 0 |
| 0 | | 22/2/2013 | | | |
| 1 | 0 | 22/2/2013 | 0 | 26/2/2013 | 0 |
| 1 | 0 | 22/2/2013 | 0 | 26/2/2013 | 0 |
| 1 | 1 | 22/2/2013 | 0 | 26/2/2013 | 0 |
| 1 | 0 | 22/2/2013 | 0 | 26/2/2013 | 0 |
| 1 | 0 | 22/2/2013 | 0 | 26/2/2013 | 0 |
| 1 | 0 | 22/2/2013 | 0 | 26/2/2013 | 0 |
| 0 | | 22/2/2013 | | | |
| 0 | | 22/2/2013 | | | |
| 0 | | 22/2/2013 | | | |
| 1 | 0 | 22/2/2013 | 0 | 26/2/2013 | |
| 1 | 0 | 22/2/2013 | 0 | 26/2/2013 | |
| 1 | 0 | 22/2/2013 | 0 | 26/2/2013 | 0 |
| 1 | 0 | 22/2/2013 | 0 | 26/2/2013 | |
| 1 | 0 | 22/2/2013 | 0 | 26/2/2013 | |
| 1 | 0 | 22/2/2013 | 0 | 26/2/2013 | |
| 0 | | 22/2/2013 | | | |
| 1 | 0 | 22/2/2013 | 0 | 26/2/2013 | |
| 1 | 0 | 22/2/2013 | | | |
| 1 | 0 | 22/2/2013 | 0 | 26/2/2013 | |
| 0 | | 22/2/2013 | | | |
| 1 | 0 | 22/2/2013 | 0 | 26/2/2013 | |
| 1 | 0 | 22/2/2013 | 0 | 26/2/2013 | |
| 0 | | 22/2/2013 | | | |
| 1 | 0 | 22/2/2013 | 0 | 26/2/2013 | |
| 1 | 0 | 22/2/2013 | 0 | 26/2/2013 | |
| 1 | 0 | 22/2/2013 | 0 | 26/2/2013 | |
| 1 | 1 | 22/2/2013 | 20 | 26/2/2013 | 33 |
| 0 | | 22/2/2013 | | | |
| 0 | | 22/2/2013 | | | |
| 1 | 0 | 22/2/2013 | | | |
| 1 | 0 | 22/2/2013 | 0 | 26/2/2013 | |
| 1 | 0 | 22/2/2013 | 0 | 26/2/2013 | |
| 1 | 0 | 22/2/2013 | 0 | 26/2/2013 | |
| 1 | 0 | 22/2/2013 | 0 | 26/2/2013 | |
| 0 | | 22/2/2013 | 0 | 26/2/2013 | |
| 1 | 0 | 22/2/2013 | 0 | 26/2/2013 | |
| 1 | 0 | 22/2/2013 | 0 | 26/2/2013 | |
| 1 | 0 | 22/2/2013 | 0 | 26/2/2013 | |
| 1 | 0 | 18/4/2013 | 0 | 18/4/2013 | 0 |
| 1 | 0 | 18/4/2013 | 0 | 18/4/2013 | 0 |

| | | | | | |
|---|---|---|---|---|---|
| 1 | 0 | 18/4/2013 | 0 | 18/4/2013 | 0 |
| 1 | 0 | 18/4/2013 | 0 | 18/4/2013 | 0 |
| 1 | 0 | 18/4/2013 | 0 | 18/4/2013 | 0 |
| 1 | 0 | 18/4/2013 | 0 | 18/4/2013 | 0 |
| 1 | 0 | 18/4/2013 | 0 | 18/4/2013 | 0 |
| 1 | 3 | 18/4/2013 | 590 | 18/4/2013 | 602 |
| 1 | 0 | 18/4/2013 | 0 | 18/4/2013 | 0 |
| 1 | 0 | 18/4/2013 | 0 | 18/4/2013 | 0 |
| 1 | 0 | 18/4/2013 | 0 | 18/4/2013 | 0 |
| 1 | 0 | 18/4/2013 | 0 | 18/4/2013 | 0 |
| 1 | 0 | 18/4/2013 | 0 | 18/4/2013 | 0 |
| 1 | 0 | 18/4/2013 | 0 | 18/4/2013 | 0 |
| 1 | 0 | 18/4/2013 | 0 | 18/4/2013 | 0 |
| 1 | 0 | 18/4/2013 | 0 | 18/4/2013 | 0 |
| 1 | 0 | 18/4/2013 | 0 | 18/4/2013 | 0 |
| 1 | 0 | 18/4/2013 | 0 | 18/4/2013 | 0 |
| 1 | 0 | 18/4/2013 | 0 | 18/4/2013 | 0 |
| 1 | 0 | 18/4/2013 | 0 | 18/4/2013 | 0 |
| 1 | 0 | 18/4/2013 | 0 | 18/4/2013 | |
| 1 | 0 | 18/4/2013 | 0 | 18/4/2013 | 0 |
| 1 | 0 | 18/4/2013 | 0 | 18/4/2013 | 0 |
| 1 | 0 | 18/4/2013 | 0 | 18/4/2013 | 0 |
| 1 | 0 | 18/4/2013 | 0 | 18/4/2013 | 0 |
| 1 | 0 | 18/4/2013 | 0 | 18/4/2013 | 0 |
| 1 | 0 | 18/4/2013 | 0 | 18/4/2013 | 0 |
| 1 | 0 | 18/4/2013 | 0 | 18/4/2013 | 0 |
| 1 | 0 | 18/4/2013 | 0 | 18/4/2013 | 0 |
| 1 | 0 | 18/4/2013 | 0 | 18/4/2013 | 0 |
| 1 | 4 | 18/4/2013 | 0 | 18/4/2013 | 2 |
| 1 | 0 | 18/4/2013 | 0 | 18/4/2013 | 0 |
| 0 | | 18/4/2013 | | | |
| 1 | 0 | 18/4/2013 | 0 | 18/4/2013 | 0 |
| 1 | 0 | 18/4/2013 | 0 | 18/4/2013 | 0 |
| 1 | 0 | 18/4/2013 | 0 | 18/4/2013 | 0 |
| 1 | 0 | 18/4/2013 | | | |
| 1 | 0 | 18/4/2013 | 0 | 18/4/2013 | 0 |
| 1 | 0 | 18/4/2013 | 0 | 18/4/2013 | 0 |
| 1 | 0 | 18/4/2013 | 0 | 18/4/2013 | 0 |
| 1 | 0 | 18/4/2013 | 0 | 18/4/2013 | 0 |
| 1 | 0 | 18/4/2013 | 0 | 18/4/2013 | 0 |
| 1 | 0 | 18/4/2013 | 0 | 18/4/2013 | 0 |
| 1 | 0 | 18/4/2013 | 0 | 18/4/2013 | 0 |
| 1 | 0 | 18/4/2013 | 0 | 18/4/2013 | 0 |
| 1 | 0 | 18/4/2013 | 0 | 18/4/2013 | 0 |
| 1 | 0 | 18/4/2013 | 0 | 18/4/2013 | 0 |
| 0 | | 18/4/2013 | | | |
| 0 | | 18/4/2013 | | | |
| 1 | 0 | 18/4/2013 | 0 | 18/4/2013 | 0 |
| 1 | 0 | 18/4/2013 | 0 | 18/4/2013 | 0 |
| 1 | 0 | 18/4/2013 | 0 | 18/4/2013 | 0 |

| | | | | | |
|---|---|---|---|---|---|
| 1 | 0 | 18/4/2013 | 0 | 18/4/2013 | 0 |
| 1 | 0 | 18/4/2013 | 0 | 18/4/2013 | 0 |
| 1 | 0 | 18/4/2013 | 0 | 18/4/2013 | 0 |
| 1 | 0 | 18/4/2013 | 0 | 18/4/2013 | 0 |
| 1 | 0 | 18/4/2013 | 0 | 18/4/2013 | 0 |
| 1 | 0 | 18/4/2013 | 0 | 18/4/2013 | 0 |
| 1 | 0 | 18/4/2013 | 0 | 18/4/2013 | 0 |
| 1 | 0 | 18/4/2013 | 0 | 18/4/2013 | 0 |
| 1 | 0 | 18/4/2013 | 0 | 18/4/2013 | 0 |
| 1 | 0 | 18/4/2013 | 0 | 18/4/2013 | 0 |
| 1 | 0 | 18/4/2013 | 0 | 18/4/2013 | 0 |
| 1 | 0 | 18/4/2013 | 0 | 18/4/2013 | 0 |
| 1 | 0 | 18/4/2013 | 0 | 18/4/2013 | 0 |
| 1 | 0 | 18/4/2013 | 0 | 18/4/2013 | 0 |
| 1 | 0 | 18/4/2013 | 0 | 18/4/2013 | 0 |
| 1 | 0 | 18/4/2013 | 0 | 18/4/2013 | 0 |
| 1 | 0 | 18/4/2013 | 0 | 18/4/2013 | |
| 1 | 0 | 18/4/2013 | 0 | 18/4/2013 | 0 |
| 1 | 0 | 18/4/2013 | 0 | 18/4/2013 | 0 |
| 1 | 0 | 18/4/2013 | 0 | 18/4/2013 | 0 |
| 1 | 0 | 18/4/2013 | 0 | 18/4/2013 | 0 |
| 1 | 0 | 18/4/2013 | 0 | 18/4/2013 | 0 |
| 1 | 0 | 18/4/2013 | 0 | 18/4/2013 | 0 |
| 1 | 0 | 18/4/2013 | 0 | 18/4/2013 | 0 |
| 1 | 0 | 18/4/2013 | 0 | 18/4/2013 | 0 |
| 1 | 0 | 18/4/2013 | 0 | 18/4/2013 | 0 |
| 1 | 0 | 18/4/2013 | 0 | 18/4/2013 | 0 |
| 1 | 2 | 18/4/2013 | 3 | 18/4/2013 | 11 |
| 1 | 0 | 18/4/2013 | 0 | 18/4/2013 | 0 |
| 1 | | 18/4/2013 | 0 | 18/4/2013 | |
| 1 | 1 | 18/4/2013 | 49 | 18/4/2013 | 99 |
| 1 | 0 | 18/4/2013 | 0 | 18/4/2013 | 0 |
| 1 | 0 | 18/4/2013 | 0 | 18/4/2013 | 0 |
| 1 | 3 | 18/4/2013 | 6 | 18/4/2013 | 45 |
| 1 | 0 | 18/4/2013 | 0 | 18/4/2013 | 0 |
| 1 | 0 | 18/4/2013 | 0 | 18/4/2013 | 0 |
| 1 | 0 | 18/4/2013 | 0 | 18/4/2013 | 0 |
| 1 | 0 | 18/4/2013 | 0 | 18/4/2013 | 0 |
| 1 | 0 | 18/4/2013 | 0 | 18/4/2013 | 0 |
| 1 | 0 | 18/4/2013 | 0 | 18/4/2013 | 0 |
| 1 | 0 | 18/4/2013 | 0 | 18/4/2013 | 0 |
| 1 | 0 | 18/4/2013 | 0 | 18/4/2013 | 0 |
| 1 | 0 | 18/4/2013 | 0 | 18/4/2013 | 0 |
| 1 | 0 | 18/4/2013 | 0 | 18/4/2013 | 0 |
| 1 | 0 | 18/4/2013 | 0 | 18/4/2013 | 0 |
| 0 | | 18/4/2013 | | | |
| 1 | 0 | 18/4/2013 | 0 | 18/4/2013 | 0 |
| 1 | 0 | 18/4/2013 | 0 | 18/4/2013 | 0 |
| 1 | 0 | 18/4/2013 | 0 | 18/4/2013 | |
| 1 | 0 | 18/4/2013 | 0 | 18/4/2013 | |

| | | | | | |
|---|---|---|---|---|---|
| 1 | 0 | 18/4/2013 | 0 | 18/4/2013 | |
| 1 | 0 | 18/4/2013 | 0 | 18/4/2013 | |
| 1 | 0 | 18/4/2013 | 0 | 18/4/2013 | |
| 1 | 0 | 18/4/2013 | 0 | 18/4/2013 | |
| 1 | 0 | 18/4/2013 | 0 | 18/4/2013 | |
| 1 | 0 | 18/4/2013 | 0 | 18/4/2013 | |
| 1 | 0 | 18/4/2013 | 0 | 18/4/2013 | |
| 1 | 0 | 18/4/2013 | 0 | 18/4/2013 | |
| 1 | 0 | 18/4/2013 | 0 | 18/4/2013 | |
| 1 | 0 | 18/4/2013 | 0 | 18/4/2013 | |
| 1 | 0 | 18/4/2013 | 0 | 18/4/2013 | |
| 1 | 0 | 18/4/2013 | 0 | 18/4/2013 | |
| 0 | | 18/4/2013 | | | |
| 1 | 0 | 18/4/2013 | 0 | 18/4/2013 | |
| 1 | 0 | 18/4/2013 | 0 | 18/4/2013 | |
| 1 | 0 | 18/4/2013 | 0 | 18/4/2013 | |
| 1 | 0 | 18/4/2013 | 0 | 18/4/2013 | |
| 1 | 0 | 18/4/2013 | 0 | 18/4/2013 | |
| 1 | 0 | 18/4/2013 | 0 | 18/4/2013 | |
| 1 | 0 | 18/4/2013 | 0 | 18/4/2013 | |
| 1 | 0 | 18/4/2013 | 0 | 18/4/2013 | |
| 1 | 0 | 18/4/2013 | 0 | 18/4/2013 | |
| 1 | 0 | 18/4/2013 | 0 | 18/4/2013 | |
| 0 | | 18/4/2013 | | | |
| 1 | 0 | 18/4/2013 | 0 | 18/4/2013 | |
| 1 | 0 | 18/4/2013 | 0 | 18/4/2013 | |
| 1 | 0 | 18/4/2013 | 0 | 18/4/2013 | |
| 1 | 0 | 18/4/2013 | 0 | 18/4/2013 | |
| 1 | 0 | 19/3/2013 | 0 | 20/3/2013 | 0 |
| 1 | 1 | 19/3/2013 | 0 | 20/3/2013 | 0 |
| 1 | 0 | 19/3/2013 | 0 | 20/3/2013 | 0 |
| 1 | 0 | 19/3/2013 | 0 | 20/3/2013 | 0 |
| 1 | 0 | 19/3/2013 | 0 | 20/3/2013 | 0 |
| 1 | 0 | 19/3/2013 | 0 | 20/3/2013 | 0 |
| 1 | 0 | 19/3/2013 | 0 | 20/3/2013 | 0 |
| 1 | 0 | 19/3/2013 | 0 | 20/3/2013 | 0 |
| 0 | | 19/3/2013 | | | |
| 0 | | 19/3/2013 | | | |
| 0 | | 19/3/2013 | | | |
| 1 | 0 | 19/3/2013 | 0 | 20/3/2013 | 0 |
| 0 | | 19/3/2013 | | | |
| 0 | | 19/3/2013 | | | |
| 1 | 0 | 19/3/2013 | 0 | 20/3/2013 | 0 |
| 1 | 0 | 19/3/2013 | 0 | 20/3/2013 | 0 |
| 0 | | 19/3/2013 | | | |
| 1 | 0 | 19/3/2013 | 0 | 20/3/2013 | 0 |
| 1 | 0 | 19/3/2013 | 0 | 20/3/2013 | 0 |
| 1 | 0 | 19/3/2013 | 0 | 20/3/2013 | 0 |
| 1 | 0 | 19/3/2013 | 0 | 20/3/2013 | 0 |
| 1 | 0 | 19/3/2013 | 0 | 20/3/2013 | 0 |

| | | | | | |
|---|---|---|---|---|---|
| 0 | | 19/3/2013 | | | |
| 1 | 0 | 19/3/2013 | 0 | 20/3/2013 | 0 |
| 1 | 0 | 19/3/2013 | 0 | 20/3/2013 | 0 |
| 0 | | 19/3/2013 | | | |
| 0 | | 19/3/2013 | | | |
| 1 | 0 | 19/3/2013 | 0 | 20/3/2013 | 0 |
| 0 | | 19/3/2013 | | | |
| 1 | 0 | 19/3/2013 | 0 | 20/3/2013 | 0 |
| 1 | 0 | 19/3/2013 | 0 | 20/3/2013 | 0 |
| 1 | 0 | 19/3/2013 | 0 | 20/3/2013 | 0 |
| 1 | 0 | 19/3/2013 | 0 | 20/3/2013 | 0 |
| 1 | 0 | 19/3/2013 | 0 | 20/3/2013 | 0 |
| 0 | | 19/3/2013 | | | |
| 0 | | 19/3/2013 | | | |
| 0 | | 19/3/2013 | | | |
| 0 | | 19/3/2013 | | | |
| 1 | 0 | 19/3/2013 | 0 | 20/3/2013 | 0 |
| 0 | | 19/3/2013 | | | |
| 1 | 0 | 19/3/2013 | 7 | 20/3/2013 | 7 |
| 0 | | 19/3/2013 | | | |
| 0 | | 19/3/2013 | | | |
| 1 | 0 | 19/3/2013 | 0 | 20/3/2013 | 0 |
| 0 | | 19/3/2013 | | | |
| 1 | 0 | 19/3/2013 | 0 | 20/3/2013 | 0 |
| 1 | 0 | 19/3/2013 | 0 | 20/3/2013 | 0 |
| 1 | 0 | 19/3/2013 | 0 | 20/3/2013 | 0 |
| 1 | 0 | 19/3/2013 | 0 | 20/3/2013 | 0 |
| 0 | | 19/3/2013 | | | |
| 0 | | 19/3/2013 | | | |
| 1 | 0 | 19/3/2013 | 0 | 20/3/2013 | 0 |
| 1 | 0 | 19/3/2013 | 0 | 20/3/2013 | 0 |
| 1 | 0 | 19/3/2013 | 0 | 20/3/2013 | 0 |
| 0 | | 19/3/2013 | | | |
| 1 | 0 | 19/3/2013 | 0 | 20/3/2013 | 0 |
| 1 | 0 | 19/3/2013 | 0 | 20/3/2013 | 0 |
| 1 | 0 | 19/3/2013 | 0 | 20/3/2013 | 0 |
| 0 | | 19/3/2013 | | | |
| 1 | 0 | 19/3/2013 | 0 | 20/3/2013 | 0 |
| 0 | | 19/3/2013 | | | |
| 1 | 0 | 19/3/2013 | 0 | 20/3/2013 | 0 |
| 1 | 0 | 19/3/2013 | 0 | 20/3/2013 | 0 |
| 1 | 0 | 19/3/2013 | 0 | 20/3/2013 | 0 |
| 0 | | 19/3/2013 | | | |
| 1 | 0 | 19/3/2013 | 0 | 20/3/2013 | 0 |
| 1 | 0 | 19/3/2013 | 0 | 20/3/2013 | 0 |
| 1 | 0 | 19/3/2013 | 0 | 20/3/2013 | 0 |
| 1 | 0 | 19/3/2013 | 0 | 20/3/2013 | 0 |
| 1 | 0 | 19/3/2013 | 0 | 20/3/2013 | 0 |
| 1 | 0 | 19/3/2013 | 0 | 20/3/2013 | 0 |
| 0 | | 19/3/2013 | | | |

| | | | | | |
|---|---|---|---|---|---|
| 1 | 0 | 19/3/2013 | 0 | 20/3/2013 | 0 |
| 1 | 0 | 19/3/2013 | 0 | 20/3/2013 | 0 |
| 1 | 0 | 19/3/2013 | 0 | 20/3/2013 | 0 |
| 1 | 0 | 19/3/2013 | 0 | 20/3/2013 | 0 |
| 1 | 4 | 19/3/2013 | 0 | 20/3/2013 | 0 |
| 1 | 0 | 19/3/2013 | 0 | 20/3/2013 | 0 |
| 1 | 0 | 19/3/2013 | 0 | 20/3/2013 | 0 |
| 0 | | 19/3/2013 | | | |
| 1 | 0 | 19/3/2013 | 0 | 20/3/2013 | 0 |
| 1 | 0 | 19/3/2013 | 0 | 20/3/2013 | 0 |
| 1 | 0 | 19/3/2013 | 0 | 20/3/2013 | 0 |
| 1 | 0 | 19/3/2013 | 0 | 20/3/2013 | 0 |
| 1 | 0 | 19/3/2013 | 0 | 20/3/2013 | 0 |
| 1 | 0 | 19/3/2013 | 0 | 20/3/2013 | 0 |
| 1 | 0 | 19/3/2013 | 0 | 20/3/2013 | 0 |
| 1 | 0 | 19/3/2013 | 0 | 20/3/2013 | 0 |
| 1 | 0 | 19/3/2013 | 0 | 20/3/2013 | 0 |
| 0 | | 19/3/2013 | | | |
| 1 | 0 | 19/3/2013 | 0 | 20/3/2013 | 0 |
| 1 | 0 | 19/3/2013 | 0 | 20/3/2013 | 0 |
| 1 | 0 | 19/3/2013 | 0 | 20/3/2013 | 0 |
| 1 | 0 | 19/3/2013 | 0 | 20/3/2013 | 0 |
| 1 | 0 | 19/3/2013 | 0 | 27/3/2013 | 0 |
| 1 | 0 | 19/3/2013 | 0 | 20/3/2013 | 0 |
| 1 | 0 | 19/3/2013 | 0 | 20/3/2013 | 0 |
| 1 | 0 | 19/3/2013 | 0 | 20/3/2013 | 0 |
| 0 | | 19/3/2013 | | | |
| 0 | | 19/3/2013 | | | |
| 1 | 0 | 19/3/2013 | 0 | 20/3/2013 | |
| 1 | 0 | 19/3/2013 | 0 | 20/3/2013 | |
| 0 | | 19/3/2013 | | | |
| 1 | 0 | 19/3/2013 | 0 | 20/3/2013 | |
| 1 | 4 | 19/3/2013 | 2 | 20/3/2013 | 3 |
| 0 | | 19/3/2013 | | | |
| 0 | | 19/3/2013 | | | |
| 1 | 0 | 19/3/2013 | 0 | 20/3/2013 | |
| 0 | | 19/3/2013 | | | |
| 0 | | 19/3/2013 | | | |
| 1 | 0 | 19/3/2013 | 0 | 20/3/2013 | |
| 1 | 0 | 19/3/2013 | 0 | 20/3/2013 | |
| 1 | 0 | 19/3/2013 | 2 | 20/3/2013 | |
| 0 | | 19/3/2013 | | | |
| 0 | | 19/3/2013 | | | |
| 1 | 0 | 19/3/2013 | 0 | 20/3/2013 | |
| 0 | | 19/3/2013 | | | |
| 0 | | 19/3/2013 | | | |
| 1 | 0 | 19/3/2013 | 0 | 20/3/2013 | 0 |
| 1 | 3 | 19/3/2013 | 0 | 20/3/2013 | |
| 1 | 0 | 19/3/2013 | 0 | 20/3/2013 | 0 |
| 1 | 0 | 19/3/2013 | 0 | 20/3/2013 | 0 |

| | | | | | |
|---|---|---|---|---|---|
| 1 | 0 | 19/3/2013 | 0 | 20/3/2013 | 0 |
| 0 | | 19/3/2013 | | | |
| 1 | 0 | 19/3/2013 | 0 | 20/3/2013 | |
| 1 | 0 | 19/3/2013 | 0 | 20/3/2013 | |
| 0 | | 19/3/2013 | | | |
| 1 | 0 | 19/3/2013 | 0 | 20/3/2013 | |
| 1 | 0 | 19/3/2013 | 0 | 20/3/2013 | |
| 1 | 0 | 19/3/2013 | 0 | 20/3/2013 | |
| 1 | 0 | 14/3/2013 | 0 | 14/3/2013 | 0 |
| 1 | 0 | 14/3/2013 | 0 | 14/3/2013 | 0 |
| 1 | 0 | 14/3/2013 | 0 | 14/3/2013 | 0 |
| 1 | 0 | 14/3/2013 | 0 | 14/3/2013 | 0 |
| 1 | 0 | 14/3/2013 | 0 | 14/3/2013 | 0 |
| 1 | 0 | 14/3/2013 | 0 | 14/3/2013 | 0 |
| 1 | 0 | 14/3/2013 | 0 | 14/3/2013 | 0 |
| 1 | 0 | 14/3/2013 | 0 | 14/3/2013 | 0 |
| 1 | 4 | 14/3/2013 | 0 | 14/3/2013 | 0 |
| 1 | 0 | 14/3/2013 | 0 | 14/3/2013 | 0 |
| 1 | 0 | 14/3/2013 | 0 | 14/3/2013 | 0 |
| 1 | 0 | 14/3/2013 | 0 | 14/3/2013 | 0 |
| 1 | 0 | 14/3/2013 | 0 | 14/3/2013 | 10 |
| 1 | 0 | 14/3/2013 | 0 | 14/3/2013 | 0 |
| 0 | | 14/3/2013 | | | |
| 1 | 0 | 14/3/2013 | 0 | 14/3/2013 | 0 |
| 1 | 0 | 14/3/2013 | 2 | 14/3/2013 | 2 |
| 1 | 0 | 14/3/2013 | 0 | 14/3/2013 | 0 |
| 1 | 0 | 14/3/2013 | 0 | 14/3/2013 | 0 |
| 1 | 0 | 14/3/2013 | 0 | 14/3/2013 | |
| 1 | 0 | 14/3/2013 | 0 | 14/3/2013 | 0 |
| 1 | 0 | 14/3/2013 | 0 | 14/3/2013 | 0 |
| 1 | 0 | 14/3/2013 | 0 | 14/3/2013 | 0 |
| 1 | 0 | 14/3/2013 | 0 | 14/3/2013 | 0 |
| 1 | 0 | 14/3/2013 | 0 | 14/3/2013 | 0 |
| 0 | | 14/3/2013 | | | |
| 1 | 0 | 14/3/2013 | 0 | 14/3/2013 | 0 |
| 0 | | 14/3/2013 | | | |
| 1 | 0 | 14/3/2013 | 0 | 14/3/2013 | 0 |
| 1 | 0 | 14/3/2013 | 0 | 14/3/2013 | 0 |
| 1 | 0 | 14/3/2013 | 0 | 14/3/2013 | 0 |
| 1 | 0 | 14/3/2013 | 0 | 14/3/2013 | 0 |
| 1 | 0 | 14/3/2013 | 0 | 14/3/2013 | 0 |
| 1 | 0 | 14/3/2013 | 0 | 14/3/2013 | 0 |
| 1 | 0 | 14/3/2013 | 21 | 14/3/2013 | 32 |
| 1 | 0 | 14/3/2013 | 0 | 14/3/2013 | 0 |
| 1 | 0 | 14/3/2013 | 0 | 14/3/2013 | 0 |
| 1 | 0 | 14/3/2013 | 0 | 14/3/2013 | 0 |
| 1 | 0 | 14/3/2013 | 0 | 14/3/2013 | 0 |
| 1 | 0 | 14/3/2013 | 0 | 14/3/2013 | 0 |
| 0 | | 14/3/2013 | | | |
| 1 | 4 | 14/3/2013 | 3 | 14/3/2013 | 5 |

| | | | | | |
|---|---|---|---|---|---|
| 1 | 0 | 14/3/2013 | 0 | 14/3/2013 | 0 |
| 1 | 0 | 14/3/2013 | 0 | 14/3/2013 | 0 |
| 1 | 0 | 14/3/2013 | 0 | 14/3/2013 | 0 |
| 1 | 0 | 14/3/2013 | 0 | 14/3/2013 | 0 |
| 0 | | 14/3/2013 | | | |
| 1 | 0 | 14/3/2013 | 0 | 14/3/2013 | 0 |
| 1 | 0 | 14/3/2013 | 0 | 14/3/2013 | 0 |
| 1 | 0 | 14/3/2013 | 0 | 14/3/2013 | 0 |
| 1 | 0 | 14/3/2013 | 0 | 14/3/2013 | 0 |
| 1 | 0 | 14/3/2013 | 0 | 14/3/2013 | 0 |
| 1 | 0 | 14/3/2013 | 0 | 14/3/2013 | 0 |
| 1 | 0 | 14/3/2013 | 0 | 14/3/2013 | 0 |
| 1 | 0 | 14/3/2013 | 0 | 14/3/2013 | 0 |
| 1 | 0 | 14/3/2013 | 0 | 14/3/2013 | 0 |
| 1 | 0 | 14/3/2013 | 0 | 14/3/2013 | 0 |
| 1 | 0 | 14/3/2013 | 0 | 14/3/2013 | 0 |
| 0 | | 14/3/2013 | | | |
| 1 | 0 | 14/3/2013 | 0 | 14/3/2013 | 0 |
| 1 | 0 | 14/3/2013 | 0 | 14/3/2013 | 0 |
| 1 | 0 | 14/3/2013 | 0 | 14/3/2013 | 1 |
| 1 | 0 | 14/3/2013 | 0 | 14/3/2013 | 0 |
| 1 | | 14/3/2013 | 0 | 14/3/2013 | 0 |
| 0 | | 14/3/2013 | | | |
| 1 | 0 | 14/3/2013 | 0 | 14/3/2013 | 0 |
| 1 | 2 | 14/3/2013 | 0 | 14/3/2013 | 1 |
| 1 | 0 | 14/3/2013 | 0 | 14/3/2013 | 0 |
| 1 | 0 | 14/3/2013 | 0 | 14/3/2013 | 0 |
| 1 | 0 | 14/3/2013 | 0 | 14/3/2013 | 0 |
| 1 | 0 | 14/3/2013 | 0 | 14/3/2013 | 0 |
| 1 | 0 | 14/3/2013 | 0 | 14/3/2013 | 0 |
| 1 | 1 | 14/3/2013 | 37 | 14/3/2013 | 30 |
| 1 | 0 | 14/3/2013 | 0 | 14/3/2013 | 1 |
| 1 | 0 | 14/3/2013 | 0 | 14/3/2013 | 0 |
| 1 | 0 | 14/3/2013 | 0 | 14/3/2013 | 0 |
| 1 | 0 | 14/3/2013 | 0 | 14/3/2013 | 0 |
| 1 | 0 | 14/3/2013 | 0 | 14/3/2013 | 0 |
| 1 | 0 | 14/3/2013 | 0 | 14/3/2013 | 0 |
| 1 | 0 | 14/3/2013 | 0 | 14/3/2013 | 0 |
| 0 | | 14/3/2013 | | | |
| 1 | 0 | 14/3/2013 | 0 | 14/3/2013 | 0 |
| 1 | 0 | 14/3/2013 | 0 | 14/3/2013 | 0 |
| 1 | 0 | 14/3/2013 | 0 | 14/3/2013 | 0 |
| 1 | 0 | 14/3/2013 | 0 | 14/3/2013 | 0 |
| 1 | 0 | 14/3/2013 | 0 | 14/3/2013 | 0 |
| 1 | 0 | 14/3/2013 | 0 | 14/3/2013 | 0 |
| 1 | 0 | 14/3/2013 | 0 | 14/3/2013 | 0 |
| 1 | 0 | 14/3/2013 | 0 | 14/3/2013 | 0 |
| 1 | 0 | 14/3/2013 | 0 | 14/3/2013 | 0 |
| 1 | 0 | 14/3/2013 | 0 | 14/3/2013 | 0 |
| 1 | 0 | 14/3/2013 | 0 | 14/3/2013 | 0 |

| | | | | | |
|---|---|---|---|---|---|
| 1 | 0 | 14/3/2013 | 0 | 14/3/2013 | 3 |
| 1 | 0 | 14/3/2013 | 0 | 14/3/2013 | 0 |
| 1 | 0 | 14/3/2013 | 0 | 14/3/2013 | 0 |
| 1 | 0 | 14/3/2013 | 0 | 14/3/2013 | 0 |
| 1 | 4 | 14/3/2013 | 25 | 14/3/2013 | 33 |
| 1 | 0 | 14/3/2013 | 0 | 14/3/2013 | 0 |
| 1 | 0 | 14/3/2013 | 0 | 14/3/2013 | 0 |
| 1 | 0 | 14/3/2013 | 0 | 14/3/2013 | 0 |
| 1 | 0 | 14/3/2013 | 0 | 14/3/2013 | |
| 1 | 0 | 14/3/2013 | 0 | 14/3/2013 | |
| 1 | 0 | 14/3/2013 | 0 | 14/3/2013 | |
| 1 | 0 | 14/3/2013 | 0 | 14/3/2013 | |
| 1 | 0 | 14/3/2013 | 0 | 14/3/2013 | |
| 1 | 0 | 14/3/2013 | 0 | 14/3/2013 | |
| 1 | 0 | 14/3/2013 | 0 | 14/3/2013 | |
| 1 | 0 | 14/3/2013 | 0 | 14/3/2013 | |
| 1 | 0 | 14/3/2013 | 0 | 14/3/2013 | |
| 1 | 2 | 14/3/2013 | 16 | 14/3/2013 | 14 |
| 1 | 4 | 14/3/2013 | 0 | 14/3/2013 | 1 |
| 1 | 1 | 14/3/2013 | 0 | 14/3/2013 | 7 |
| 1 | 0 | 14/3/2013 | 1 | 14/3/2013 | |
| 1 | 0 | 14/3/2013 | 0 | 14/3/2013 | |
| 1 | 0 | 14/3/2013 | 0 | 14/3/2013 | |
| 1 | 0 | 14/3/2013 | 0 | 14/3/2013 | |
| 1 | 0 | 14/3/2013 | 0 | 14/3/2013 | |
| 1 | 0 | 14/3/2013 | 0 | 14/3/2013 | |
| 1 | 0 | 14/3/2013 | 0 | 14/3/2013 | |
| 1 | 0 | 14/3/2013 | 0 | 14/3/2013 | |
| 1 | 0 | 14/3/2013 | 0 | 14/3/2013 | |
| 1 | 0 | 14/3/2013 | 0 | 14/3/2013 | |
| 1 | 0 | 14/3/2013 | 0 | 14/3/2013 | |
| 1 | 0 | 14/3/2013 | 0 | 14/3/2013 | |
| 1 | 0 | 14/3/2013 | 0 | 14/3/2013 | |
| 1 | 0 | 14/3/2013 | 0 | 14/3/2013 | |
| 1 | 0 | 14/3/2013 | 0 | 14/3/2013 | |
| 1 | 0 | 14/3/2013 | 2 | 14/3/2013 | 2 |
| 1 | 0 | 14/3/2013 | 0 | 14/3/2013 | |
| 1 | 0 | 14/3/2013 | 0 | 14/3/2013 | |
| 1 | 0 | 15/3/2013 | 0 | 18/3/2013 | 0 |
| 1 | 0 | 15/3/2013 | 0 | 18/3/2013 | 0 |
| 1 | 0 | 15/3/2013 | 0 | 18/3/2013 | 0 |
| 1 | 0 | 15/3/2013 | 0 | 18/3/2013 | 0 |
| 1 | 0 | 15/3/2013 | 0 | 18/3/2013 | 0 |
| 1 | 0 | 15/3/2013 | 0 | 18/3/2013 | 0 |
| 1 | 0 | 15/3/2013 | 0 | 18/3/2013 | |
| 1 | 0 | 15/3/2013 | 0 | 18/3/2013 | 0 |
| 1 | 0 | 15/3/2013 | 0 | 18/3/2013 | 0 |
| 1 | 0 | 15/3/2013 | 0 | 18/3/2013 | 0 |
| 1 | 0 | 15/3/2013 | 0 | 18/3/2013 | 0 |
| 1 | 0 | 15/3/2013 | 0 | 18/3/2013 | 0 |

| | | | | | |
|---|---|---|---|---|---|
| 1 | 0 | 15/3/2013 | 0 | 18/3/2013 | 0 |
| 1 | 0 | 15/3/2013 | 0 | 18/3/2013 | 0 |
| 1 | 0 | 15/3/2013 | 0 | 18/3/2013 | 0 |
| 1 | 0 | 15/3/2013 | 0 | 18/3/2013 | 0 |
| 1 | 0 | 15/3/2013 | 0 | 18/3/2013 | 0 |
| 1 | 0 | 15/3/2013 | 0 | 18/3/2013 | 0 |
| 0 | | 15/3/2013 | | | |
| 1 | 0 | 15/3/2013 | 0 | 18/3/2013 | 0 |
| 1 | 0 | 15/3/2013 | 0 | 18/3/2013 | 0 |
| 1 | 0 | 15/3/2013 | 0 | 18/3/2013 | 0 |
| 1 | 0 | 15/3/2013 | 0 | 18/3/2013 | 0 |
| 1 | 0 | 15/3/2013 | 0 | 18/3/2013 | 0 |
| 1 | 0 | 15/3/2013 | 0 | 18/3/2013 | |
| 1 | 0 | 15/3/2013 | 0 | 18/3/2013 | 0 |
| 1 | 0 | 15/3/2013 | 0 | 18/3/2013 | 0 |
| 0 | | 15/3/2013 | | | |
| 1 | 0 | 15/3/2013 | 0 | 18/3/2013 | 0 |
| 1 | 0 | 15/3/2013 | 0 | 18/3/2013 | 0 |
| 1 | 0 | 15/3/2013 | 0 | 18/3/2013 | 0 |
| 0 | | 15/3/2013 | | | |
| 1 | 0 | 15/3/2013 | 0 | 18/3/2013 | 0 |
| 1 | 0 | 15/3/2013 | 0 | 18/3/2013 | 0 |
| 1 | 0 | 15/3/2013 | 0 | 18/3/2013 | 0 |
| 1 | 0 | 15/3/2013 | 0 | 18/3/2013 | 0 |
| 1 | 0 | 15/3/2013 | 0 | 18/3/2013 | 0 |
| 0 | | 15/3/2013 | | | |
| 0 | | 15/3/2013 | | | |
| 1 | 0 | 15/3/2013 | 0 | 18/3/2013 | 0 |
| 1 | 0 | 15/3/2013 | 0 | 18/3/2013 | 0 |
| 1 | 0 | 15/3/2013 | 0 | 18/3/2013 | 0 |
| 1 | 0 | 15/3/2013 | 0 | 18/3/2013 | 0 |
| 1 | 0 | 15/3/2013 | 0 | 18/3/2013 | 0 |
| 0 | | 15/3/2013 | | | |
| 1 | 0 | 15/3/2013 | 0 | 18/3/2013 | 0 |
| 1 | 0 | 15/3/2013 | 0 | 18/3/2013 | 0 |
| 1 | 0 | 15/3/2013 | 0 | 18/3/2013 | 0 |
| 1 | 0 | 15/3/2013 | 0 | 18/3/2013 | 0 |
| 1 | 0 | 15/3/2013 | 0 | 18/3/2013 | 0 |
| 1 | 0 | 15/3/2013 | 0 | 18/3/2013 | 0 |
| 1 | 0 | 15/3/2013 | 0 | 18/3/2013 | 0 |
| 1 | 0 | 15/3/2013 | 0 | 18/3/2013 | 0 |
| 1 | 0 | 15/3/2013 | 0 | 18/3/2013 | 0 |
| 1 | 0 | 15/3/2013 | 0 | 18/3/2013 | 0 |
| 1 | 0 | 15/3/2013 | 0 | 18/3/2013 | 0 |
| 1 | 0 | 15/3/2013 | 0 | 18/3/2013 | 0 |
| 0 | | 15/3/2013 | | | |
| 1 | 0 | 15/3/2013 | 0 | 18/3/2013 | 0 |
| 1 | 0 | 15/3/2013 | 0 | 18/3/2013 | 0 |
| 1 | 0 | 15/3/2013 | 0 | 18/3/2013 | 0 |
| 1 | 0 | 15/3/2013 | 0 | 18/3/2013 | 0 |

| | | | | | |
|---|---|---|---|---|---|
| 0 | | 15/3/2013 | | | |
| 1 | 0 | 15/3/2013 | 6 | 18/3/2013 | 8 |
| 1 | 0 | 15/3/2013 | 0 | 18/3/2013 | 0 |
| 1 | 0 | 15/3/2013 | 1 | 18/3/2013 | 1 |
| 1 | 0 | 15/3/2013 | 7 | 18/3/2013 | 13 |
| 1 | 0 | 15/3/2013 | 0 | 18/3/2013 | 0 |
| 0 | | 15/3/2013 | | | |
| 1 | 0 | 15/3/2013 | 0 | 18/3/2013 | 0 |
| 1 | 0 | 15/3/2013 | 0 | 18/3/2013 | 0 |
| 1 | 0 | 15/3/2013 | 0 | 18/3/2013 | 0 |
| 1 | 0 | 15/3/2013 | 0 | 18/3/2013 | 0 |
| 1 | 0 | 15/3/2013 | 0 | 18/3/2013 | 0 |
| 1 | 0 | 15/3/2013 | 0 | 18/3/2013 | 0 |
| 1 | 0 | 15/3/2013 | 0 | 18/3/2013 | 0 |
| 1 | 0 | 15/3/2013 | 0 | 18/3/2013 | 0 |
| 1 | 0 | 15/3/2013 | 0 | 18/3/2013 | 0 |
| 1 | 0 | 15/3/2013 | 0 | 18/3/2013 | 0 |
| 1 | 0 | 15/3/2013 | 0 | 18/3/2013 | 0 |
| 0 | | 15/3/2013 | | | |
| 1 | 0 | 15/3/2013 | 0 | 18/3/2013 | 0 |
| 1 | 0 | 15/3/2013 | 0 | 18/3/2013 | 0 |
| 1 | 0 | 15/3/2013 | 0 | 18/3/2013 | 0 |
| 1 | 0 | 15/3/2013 | 0 | 18/3/2013 | 0 |
| 1 | 0 | 15/3/2013 | 0 | 18/3/2013 | 0 |
| 1 | 0 | 15/3/2013 | 0 | 18/3/2013 | 0 |
| 1 | 0 | 15/3/2013 | 0 | 18/3/2013 | 0 |
| 1 | 0 | 15/3/2013 | 0 | 18/3/2013 | 0 |
| 1 | 0 | 15/3/2013 | 4 | 18/3/2013 | 2 |
| 1 | 0 | 15/3/2013 | 0 | 18/3/2013 | 2 |
| 1 | 0 | 15/3/2013 | 0 | 18/3/2013 | 0 |
| 1 | 0 | 15/3/2013 | 0 | 18/3/2013 | 0 |
| 1 | 0 | 15/3/2013 | 0 | 18/3/2013 | 0 |
| 1 | 0 | 15/3/2013 | 0 | 18/3/2013 | 0 |
| 1 | 0 | 15/3/2013 | 0 | 18/3/2013 | 0 |
| 1 | 0 | 15/3/2013 | 0 | 18/3/2013 | 0 |
| 1 | 0 | 15/3/2013 | 0 | 18/3/2013 | 0 |
| 1 | 0 | 15/3/2013 | 0 | 18/3/2013 | 0 |
| 1 | 0 | 15/3/2013 | 0 | 18/3/2013 | 0 |
| 0 | | 15/3/2013 | | | |
| 1 | 0 | 15/3/2013 | 0 | 18/3/2013 | |
| 1 | 0 | 15/3/2013 | 0 | 18/3/2013 | |
| 1 | 0 | 15/3/2013 | 0 | 18/3/2013 | |
| 1 | 0 | 15/3/2013 | 0 | 18/3/2013 | |
| 1 | 0 | 15/3/2013 | 0 | 18/3/2013 | |
| 1 | 0 | 15/3/2013 | 0 | 18/3/2013 | |
| 1 | 3 | 15/3/2013 | 8 | 18/3/2013 | |
| 1 | 0 | 15/3/2013 | 0 | 18/3/2013 | |
| 1 | 0 | 15/3/2013 | 0 | 18/3/2013 | |
| 1 | 0 | 15/3/2013 | 0 | 18/3/2013 | |
| 1 | 0 | 15/3/2013 | 0 | 18/3/2013 | |

| | | | | | | |
|---|---|---|---|---|---|---|
| 1 | 0 | 15/3/2013 | 0 | 18/3/2013 | |
| 1 | 0 | 15/3/2013 | 0 | 18/3/2013 | |
| 1 | 0 | 15/3/2013 | 0 | 18/3/2013 | |
| 1 | 0 | 15/3/2013 | 0 | 18/3/2013 | |
| 0 | | 15/3/2013 | | | |
| 1 | 0 | 15/3/2013 | 0 | 18/3/2013 | |
| 1 | 0 | 15/3/2013 | 0 | 18/3/2013 | |
| 1 | 0 | 15/3/2013 | 0 | 18/3/2013 | |
| 1 | 0 | 15/3/2013 | 0 | 18/3/2013 | |
| 1 | 0 | 15/3/2013 | 0 | 18/3/2013 | |
| 1 | 0 | 15/3/2013 | 0 | 18/3/2013 | |
| 1 | 0 | 15/3/2013 | 0 | 18/3/2013 | |
| 1 | 0 | 15/3/2013 | 0 | 18/3/2013 | |
| 1 | 0 | 15/3/2013 | 0 | 18/3/2013 | |
| 1 | 0 | 15/3/2013 | 0 | 18/3/2013 | |
| 1 | 0 | 15/3/2013 | 0 | 18/3/2013 | |
| 1 | 0 | 15/3/2013 | 0 | 18/3/2013 | |
| 1 | 3 | 15/3/2013 | 630 | 18/3/2013 | 423 |

| Test date/series | CAA (pg/ml) | CAA pn | Ratio UCP | Assay |
|---|---|---|---|---|
| 18-11-2013 - series 1 | 0.0000000000000000 | neg | 0.016398819285012 | UCAA2000 |
| 18-11-2013 - series 1 | 0.0000000000000000 | neg | 0.022222222222222 | UCAA2000 |
| 18-11-2013 - series 1 | 0.0000000000000000 | neg | 0.019683908045977 | UCAA2000 |
| 18-11-2013 - series 1 | 0.0000000000000000 | neg | 0.019440042260961 | UCAA2000 |
| 18-11-2013 - series 1 | 0.0000000000000000 | neg | 0.011022927689594 | UCAA2000 |
| 18-11-2013 - series 1 | 0.0164703297673097 | neg | 0.039509954058193 | UCAA2000 |
| 18-11-2013 - series 1 | 0.0454156555959439 | neg | 0.056022808267997 | UCAA2000 |
| 18-11-2013 - series 1 | 0.0000000000000000 | neg | 0.019719976336028 | UCAA2000 |
| 18-11-2013 - series 1 | 0.0138200641057651 | neg | 0.037866069763104 | UCAA2000 |
| 18-11-2013 - series 1 | 0.1596178560387530 | neg | 0.111903526970954 | UCAA2000 |
| 18-11-2013 - series 1 | 16.6590661529444000 | pos | 2.774375743162900 | UCAA2000 |
| 18-11-2013 - series 1 | 0.0000000000000000 | neg | 0.010490977759127 | UCAA2000 |
| 18-11-2013 - series 1 | 0.0405717383726756 | neg | 0.053386341047849 | UCAA2000 |
| 18-11-2013 - series 1 | 0.0181565487380255 | neg | 0.040538625088590 | UCAA2000 |
| 18-11-2013 - series 1 | 0.0000000000000000 | neg | 0.011212019284673 | UCAA2000 |
| 18-11-2013 - series 1 | 0.0000000000000000 | neg | 0.025862068965517 | UCAA2000 |
| 18-11-2013 - series 1 | 0.0149055650623216 | neg | 0.038543689320388 | UCAA2000 |
| 18-11-2013 - series 1 | 0.0090601383753763 | neg | 0.034807641978540 | UCAA2000 |
| 18-11-2013 - series 1 | 0.0000000000000000 | neg | 0.014216661927779 | UCAA2000 |
| 18-11-2013 - series 1 | 0.0000000000000000 | neg | 0.010683760683761 | UCAA2000 |
| 18-11-2013 - series 1 | 0.0000000000000000 | neg | 0.014819205690575 | UCAA2000 |
| 18-11-2013 - series 1 | 0.0000000000000000 | neg | 0.011664528169836 | UCAA2000 |
| 18-11-2013 - series 1 | 0.0000000000000000 | neg | 0.013908205841447 | UCAA2000 |
| 18-11-2013 - series 1 | 0.0000000000000000 | neg | 0.015062509414068 | UCAA2000 |
| 18-11-2013 - series 1 | 12.8071235356559000 | pos | 2.435775734203500 | UCAA2000 |
| 18-11-2013 - series 1 | 0.0000000000000000 | neg | 0.016798252981690 | UCAA2000 |
| 18-11-2013 - series 1 | 0.0000000000000000 | neg | 0.014037057832678 | UCAA2000 |
| 18-11-2013 - series 1 | 0.0000000000000000 | neg | 0.012523481527865 | UCAA2000 |
| 18-11-2013 - series 1 | 0.0000000000000000 | neg | 0.015992323684631 | UCAA2000 |
| 18-11-2013 - series 1 | 0.0984417610459948 | neg | 0.083104284559418 | UCAA2000 |
| 18-11-2013 - series 1 | 0.0000000000000000 | neg | 0.012163970319912 | UCAA2000 |
| 18-11-2013 - series 1 | 0.0000000000000000 | neg | 0.014505366985785 | UCAA2000 |
| 18-11-2013 - series 1 | 0.0000000000000000 | neg | 0.014845605700713 | UCAA2000 |
| 18-11-2013 - series 1 | 0.0075458718780834 | neg | 0.033796634967081 | UCAA2000 |
| 18-11-2013 - series 2 | 15.5671392720495000 | pos | 2.686118479221930 | UCAA2000 |
| 18-11-2013 - series 2 | 0.0000000000000000 | neg | 0.006293832044596 | UCAA2000 |
| | | | | |
| 18-11-2013 - series 2 | 0.0000000000000000 | neg | 0.019967218000298 | UCAA2000 |
| 18-11-2013 - series 2 | 0.0477836016156388 | neg | 0.057298335467350 | UCAA2000 |
| 18-11-2013 - series 2 | 0.0619937546105507 | neg | 0.064795564795565 | UCAA2000 |
| 18-11-2013 - series 2 | 0.1320932960954840 | neg | 0.099183736331434 | UCAA2000 |
| 18-11-2013 - series 2 | 0.0000000000000000 | neg | 0.017959770114943 | UCAA2000 |
| 18-11-2013 - series 2 | 0.0164230011025499 | neg | 0.039480899287150 | UCAA2000 |
| 18-11-2013 - series 2 | 0.0000000000000000 | neg | 0.024553348637589 | UCAA2000 |
| 18-11-2013 - series 2 | 0.0000000000000000 | neg | 0.006915213469633 | UCAA2000 |
| 18-11-2013 - series 2 | 0.0000000000000000 | neg | 0.011448481831757 | UCAA2000 |
| | | | | |
| 18-11-2013 - series 2 | 0.0000000000000000 | neg | 0.013579576317219 | UCAA2000 |
| 18-11-2013 - series 2 | 4.1230298886774200 | pos | 1.219200000000000 | UCAA2000 |

| | | | | |
|---|---|---|---|---|
| 18-11-2013 - series 2 | 0.0000000000000000 | neg | 0.016000000000000 | UCAA2000 |
| 18-11-2013 - series 2 | 0.0000000000000000 | neg | 0.016509823344890 | UCAA2000 |
| 18-11-2013 - series 2 | 0.0000000000000000 | neg | 0.012280486307258 | UCAA2000 |
| 18-11-2013 - series 2 | 0.0000000000000000 | neg | 0.015969338869371 | UCAA2000 |
| 18-11-2013 - series 2 | 0.0117833965744434 | neg | 0.036576444769568 | UCAA2000 |
| 18-11-2013 - series 2 | 0.0000000000000000 | neg | 0.016431153466973 | UCAA2000 |
| 18-11-2013 - series 2 | 0.0000000000000000 | neg | 0.013175230566535 | UCAA2000 |
| 18-11-2013 - series 2 | 0.0000000000000000 | neg | 0.013588802826471 | UCAA2000 |
| 18-11-2013 - series 2 | 0.0000000000000000 | neg | 0.020495867768595 | UCAA2000 |
| 18-11-2013 - series 2 | 0.0105234753245234 | neg | 0.035765124555160 | UCAA2000 |
| 18-11-2013 - series 2 | 0.0000000000000000 | neg | 0.018135654697135 | UCAA2000 |
| 18-11-2013 - series 2 | 0.0000000000000000 | neg | 0.005242966751918 | UCAA2000 |
| 18-11-2013 - series 2 | 0.0960812828970658 | neg | 0.081950359817888 | UCAA2000 |
| 18-11-2013 - series 2 | 0.0708027356905505 | neg | 0.069327731092437 | UCAA2000 |
| 18-11-2013 - series 2 | 0.0217688171534628 | neg | 0.042704065553104 | UCAA2000 |
| 18-11-2013 - series 2 | 0.0292561332162755 | neg | 0.047058823529412 | UCAA2000 |
| 18-11-2013 - series 2 | 0.3575700388193560 | indecisive | 0.196176604460628 | UCAA2000 |
| 18-11-2013 - series 2 | 0.0802290836383550 | neg | 0.074095682613769 | UCAA2000 |
| 18-11-2013 - series 2 | 0.0048005597991952 | neg | 0.031894636623294 | UCAA2000 |
| 18-11-2013 - series 2 | 0.0000000000000000 | neg | 0.019290123456790 | UCAA2000 |
| 18-11-2013 - series 2 | 0.0000000000000000 | neg | 0.002576950608447 | UCAA2000 |
| 18-11-2013 - series 2 | 0.0000000000000000 | neg | 0.026223366892999 | UCAA2000 |
| 18-11-2013 - series 2 | 0.0271703788610555 | neg | 0.045861451590690 | UCAA2000 |
| 18-11-2013 - series 2 | 0.0000000000000000 | neg | 0.018487705675726 | UCAA2000 |
| 18-11-2013 - series 3 | 0.0000000000000000 | neg | 0.003398248594955 | UCAA2000 |
| 18-11-2013 - series 3 | 0.0000000000000000 | neg | 0.025297619047619 | UCAA2000 |
| 18-11-2013 - series 3 | 0.0000000000000000 | neg | 0.016313364055300 | UCAA2000 |
| 18-11-2013 - series 3 | 1.3454785083899100 | pos | 0.536162841056735 | UCAA2000 |
| 18-11-2013 - series 3 | 0.0000000000000000 | neg | 0.022033448367401 | UCAA2000 |
| 18-11-2013 - series 3 | 0.0158069568412421 | neg | 0.039101758793970 | UCAA2000 |
| 18-11-2013 - series 3 | 0.0361418482779056 | neg | 0.050939876596355 | UCAA2000 |
| 18-11-2013 - series 3 | 0.0000000000000000 | neg | 0.000739827373613 | UCAA2000 |
| 18-11-2013 - series 3 | 0.0000000000000000 | neg | 0.016448099577684 | UCAA2000 |
| 18-11-2013 - series 3 | 0.0036038024473638 | neg | 0.031026515568269 | UCAA2000 |
| 18-11-2013 - series 3 | 0.0000000000000000 | neg | 0.024857104878373 | UCAA2000 |
| 18-11-2013 - series 3 | 0.0096294614611825 | neg | 0.035182250396197 | UCAA2000 |
| 18-11-2013 - series 3 | 0.0000000000000000 | neg | 0.014332807797047 | UCAA2000 |
| 18-11-2013 - series 3 | 0.0000000000000000 | neg | 0.013972334777141 | UCAA2000 |
| 18-11-2013 - series 3 | 0.0000000000000000 | neg | 0.023792862141358 | UCAA2000 |
| 18-11-2013 - series 3 | 0.0000000000000000 | neg | 0.016268098259314 | UCAA2000 |
| 18-11-2013 - series 3 | 0.0000000000000000 | neg | 0.025571443481891 | UCAA2000 |

| | | | | |
|---|---|---|---|---|
| 18-11-2013 - series 3 | 0.0000000000000000 | neg | 0.012227928588897 | UCAA2000 |
| 18-11-2013 - series 3 | 0.0122851806268044 | neg | 0.036896551724138 | UCAA2000 |
| 18-11-2013 - series 3 | 0.0932149182576143 | neg | 0.080543933054393 | UCAA2000 |
| 18-11-2013 - series 3 | 0.0228061239779167 | neg | 0.043317340644277 | UCAA2000 |
| 18-11-2013 - series 3 | 0.0000000000000000 | neg | 0.007955355022560 | UCAA2000 |
| 18-11-2013 - series 3 | 0.0000000000000000 | neg | 0.012349490583513 | UCAA2000 |
| 18-11-2013 - series 3 | 0.0200968332759722 | neg | 0.041707798617966 | UCAA2000 |
| 18-11-2013 - series 3 | 0.0000000000000000 | neg | 0.002878464818763 | UCAA2000 |
| 18-11-2013 - series 3 | 0.0267401419713992 | neg | 0.045613056214256 | UCAA2000 |
| 18-11-2013 - series 3 | 0.0749368307230638 | neg | 0.071428571428571 | UCAA2000 |
| 18-11-2013 - series 3 | 0.0000000000000000 | neg | 0.013869625520111 | UCAA2000 |

| | | | | |
|---|---|---|---|---|
| 18-11-2013 - series 3 | 0.0001596633952680 | neg | 0.028194993412385 | UCAA2000 |
| 18-11-2013 - series 3 | 0.0000000000000000 | neg | 0.015851294332307 | UCAA2000 |
| 18-11-2013 - series 3 | 0.0231248149056935 | neg | 0.043505052994824 | UCAA2000 |
| 18-11-2013 - series 3 | 0.0900931976472062 | neg | 0.079005524861879 | UCAA2000 |
| 18-11-2013 - series 3 | 0.0488747990619470 | neg | 0.057883369330454 | UCAA2000 |
| 18-11-2013 - series 3 | 0.0134766049471707 | neg | 0.037650323774283 | UCAA2000 |
| 18-11-2013 - series 3 | 0.0000000000000000 | neg | 0.013185654008439 | UCAA2000 |
| 18-11-2013 - series 3 | 0.0000000000000000 | neg | 0.003635537377869 | UCAA2000 |
| 19-11-2013 - series 1 | 0.0389960498617119 | neg | 0.036311810633288 | UCAA2000 |
| 18-11-2013 - series 3 | 0.0000000000000000 | neg | 0.003361721201255 | UCAA2000 |
| 19-11-2013 - series 1 | 23.1491771171693000 | pos | 2.784892086330940 | UCAA2000 |
| 19-11-2013 - series 1 | 0.0000000000000000 | neg | 0.012136697540722 | UCAA2000 |
| 19-11-2013 - series 1 | 0.0000000000000000 | neg | 0.003201970443350 | UCAA2000 |
| 19-11-2013 - series 1 | 0.0000000000000000 | neg | 0.009479571523367 | UCAA2000 |
| 19-11-2013 - series 1 | 0.0227536494612969 | neg | 0.028321863419799 | UCAA2000 |
| 19-11-2013 - series 1 | 0.0000000000000000 | neg | 0.010167768174886 | UCAA2000 |
| 19-11-2013 - series 1 | 0.0000000000000000 | neg | 0.008315764816116 | UCAA2000 |
| 19-11-2013 - series 1 | 0.0010180650407817 | neg | 0.014359194170596 | UCAA2000 |
| 19-11-2013 - series 1 | 0.0025809498927468 | neg | 0.015807777426494 | UCAA2000 |
| 19-11-2013 - series 1 | 0.0005248849538947 | neg | 0.013810741687980 | UCAA2000 |
| 19-11-2013 - series 1 | 0.0321377724656109 | neg | 0.033051498847041 | UCAA2000 |
| 19-11-2013 - series 1 | 0.0021252757296732 | neg | 0.015413070283601 | UCAA2000 |
| 19-11-2013 - series 1 | 0.0000000000000000 | neg | 0.010009008107297 | UCAA2000 |
| 19-11-2013 - series 1 | 0.0000000000000000 | neg | 0.012706480304956 | UCAA2000 |
| 19-11-2013 - series 1 | 0.0000000000000000 | neg | 0.011253657438668 | UCAA2000 |
| 19-11-2013 - series 1 | 0.0203664981650276 | neg | 0.027054108216433 | UCAA2000 |
| 19-11-2013 - series 1 | 0.0145937215602753 | neg | 0.023839051949842 | UCAA2000 |
| 19-11-2013 - series 1 | 0.0024221213588089 | neg | 0.015672091621459 | UCAA2000 |
| 19-11-2013 - series 1 | 0.0439924884982221 | neg | 0.038606403013183 | UCAA2000 |
| 19-11-2013 - series 1 | 0.0299976221477041 | neg | 0.032003459833496 | UCAA2000 |
| 19-11-2013 - series 1 | 8.8715173241824200 | pos | 1.466423637759020 | UCAA2000 |
| 19-11-2013 - series 1 | 0.0000324331711504 | neg | 0.013092432573972 | UCAA2000 |
| 19-11-2013 - series 1 | 0.0000000000000000 | neg | 0.012601478250333 | UCAA2000 |
| 19-11-2013 - series 1 | 0.0122109748740575 | neg | 0.022432956051800 | UCAA2000 |
| 19-11-2013 - series 1 | 0.0000000423273883 | neg | 0.013000520020801 | UCAA2000 |
| 19-11-2013 - series 1 | 2.2238498367370000 | pos | 0.540751240255138 | UCAA2000 |
| 19-11-2013 - series 1 | 0.0139626238121237 | neg | 0.023471929488408 | UCAA2000 |

| | | | | |
|---|---|---|---|---|
| 19-11-2013 - series 1 | 0.0000000000000000 | neg | 0.010648493238207 | UCAA2000 |
| 19-11-2013 - series 2 | 0.0033557082952934 | neg | 0.016445623342175 | UCAA2000 |
| 19-11-2013 - series 2 | 0.0109462872830286 | neg | 0.021662296203398 | UCAA2000 |
| 19-11-2013 - series 2 | 0.0002240870042999 | neg | 0.013417415805716 | UCAA2000 |
| 19-11-2013 - series 2 | 1.9948708908449500 | pos | 0.499256136870816 | UCAA2000 |
| 19-11-2013 - series 2 | 0.0000000000000000 | neg | 0.012556504269212 | UCAA2000 |
| | | | | |
| 19-11-2013 - series 2 | 1.0426909257846300 | pos | 0.309978540772532 | UCAA2000 |
| | | | | |
| 19-11-2013 - series 2 | 0.0000000000000000 | neg | 0.012062726176116 | UCAA2000 |
| 19-11-2013 - series 2 | 0.0000000000000000 | neg | 0.011633317822243 | UCAA2000 |
| 19-11-2013 - series 2 | 0.0115423265602087 | neg | 0.022027824620573 | UCAA2000 |
| 19-11-2013 - series 2 | 0.0000000000000000 | neg | 0.011176930814798 | UCAA2000 |
| 19-11-2013 - series 2 | 0.0000000000000000 | neg | 0.009644064426327 | UCAA2000 |
| 19-11-2013 - series 2 | 0.0000000000000000 | neg | 0.010760787689659 | UCAA2000 |
| 19-11-2013 - series 2 | 0.0000000000000000 | neg | 0.009739943508328 | UCAA2000 |
| | | | | |
| 19-11-2013 - series 2 | 0.0419517630094191 | neg | 0.037676609105181 | UCAA2000 |
| 19-11-2013 - series 2 | 0.0118308269432805 | neg | 0.022203245089667 | UCAA2000 |
| 19-11-2013 - series 2 | 0.0160112899935510 | neg | 0.024651202602071 | UCAA2000 |
| 19-11-2013 - series 2 | 0.0000000000000000 | neg | 0.007419930496854 | UCAA2000 |
| 19-11-2013 - series 2 | 0.0000000000000000 | neg | 0.010780508840017 | UCAA2000 |
| 19-11-2013 - series 2 | 0.0000000000000000 | neg | 0.009192645883293 | UCAA2000 |
| | | | | |
| 19-11-2013 - series 2 | 0.0000000000000000 | neg | 0.004379149597401 | UCAA2000 |
| 19-11-2013 - series 2 | 0.0000000000000000 | neg | 0.002100375856732 | UCAA2000 |
| 19-11-2013 - series 2 | 0.0333001073957288 | neg | 0.033614167812930 | UCAA2000 |
| 19-11-2013 - series 2 | 0.0000000000000000 | neg | 0.011548677676406 | UCAA2000 |

| | | | | |
|---|---|---|---|---|
| 19-11-2013 - series 2 | 39.5512718162793000 | pos | 3.854007633587790 | UCAA2000 |
| 19-11-2013 - series 2 | 0.0416388799094477 | neg | 0.037533173259194 | UCAA2000 |
| 19-11-2013 - series 2 | 0.0000000000000000 | neg | 0.011442956860053 | UCAA2000 |
| 19-11-2013 - series 2 | 0.0000000000000000 | neg | 0.008167932696235 | UCAA2000 |
| 19-11-2013 - series 2 | 0.0098397586910540 | neg | 0.020971733750163 | UCAA2000 |
| 19-11-2013 - series 2 | 0.0223327194766444 | neg | 0.028100545411430 | UCAA2000 |
| 19-11-2013 - series 2 | 0.0041985613652705 | neg | 0.017103509551311 | UCAA2000 |
| 19-11-2013 - series 2 | 0.0000000000000000 | neg | 0.005526631958350 | UCAA2000 |
| 19-11-2013 - series 2 | 0.0086463152001041 | neg | 0.020207344930592 | UCAA2000 |
| 19-11-2013 - series 2 | 0.0000000000000000 | neg | 0.009479694064419 | UCAA2000 |
| 19-11-2013 - series 2 | 0.0237538112936708 | neg | 0.028844136773652 | UCAA2000 |
| 19-11-2013 - series 2 | 0.0263029049563650 | neg | 0.030153846153846 | UCAA2000 |
| 19-11-2013 - series 2 | 0.0000000000000000 | neg | 0.011403808872163 | UCAA2000 |
| 19-11-2013 - series 2 | 0.0041861103163570 | neg | 0.017094017094017 | UCAA2000 |
| 19-11-2013 - series 3 | 11.5777290945886000 | pos | 1.762711864406780 | UCAA2000 |
| 19-11-2013 - series 3 | 0.0461390792503246 | neg | 0.039574126155082 | UCAA2000 |
| 19-11-2013 - series 3 | 0.0027242677558681 | neg | 0.015928639694170 | UCAA2000 |
| 19-11-2013 - series 3 | 0.0356976208271387 | neg | 0.034761235955056 | UCAA2000 |
| 19-11-2013 - series 3 | 0.0291293354809407 | neg | 0.031573565128467 | UCAA2000 |
| 19-11-2013 - series 3 | 0.0000000000000000 | neg | 0.010730765103552 | UCAA2000 |
| 19-11-2013 - series 3 | 0.0395527939841109 | neg | 0.036570598207799 | UCAA2000 |
| 19-11-2013 - series 3 | 0.0000000000000000 | neg | 0.011095084877399 | UCAA2000 |
| 19-11-2013 - series 3 | 0.0007532668724484 | neg | 0.014074595355384 | UCAA2000 |
| 19-11-2013 - series 3 | 0.1663895172578620 | neg | 0.085051212239077 | UCAA2000 |
| 19-11-2013 - series 3 | 0.0000000000000000 | neg | 0.010223903486351 | UCAA2000 |
| 19-11-2013 - series 3 | 0.0052692840055884 | neg | 0.017898693395382 | UCAA2000 |
| 19-11-2013 - series 3 | 0.0000608365143680 | neg | 0.013150973172015 | UCAA2000 |
| 19-11-2013 - series 3 | 0.0000347653216274 | neg | 0.013097576948265 | UCAA2000 |
| 19-11-2013 - series 3 | 0.0000000000000000 | neg | 0.012242899118511 | UCAA2000 |
| 19-11-2013 - series 3 | 0.0763073194572477 | neg | 0.052311259237792 | UCAA2000 |

| | | | | |
|---|---|---|---|---|
| 19-11-2013 - series 3 | 0.2755098431047890 | indecisive | 0.119528283030182 | UCAA2000 |
| 19-11-2013 - series 3 | 0.0186499944475388 | neg | 0.026122148638705 | UCAA2000 |
| 19-11-2013 - series 3 | 0.0338228979626124 | neg | 0.033865814696486 | UCAA2000 |
| 19-11-2013 - series 3 | 0.0000000000000000 | neg | 0.012805736970163 | UCAA2000 |
| 19-11-2013 - series 3 | 0.0000000000000000 | neg | 0.012809017548354 | UCAA2000 |
| 19-11-2013 - series 3 | 2.0018079634326700 | pos | 0.500531672489746 | UCAA2000 |
| 19-11-2013 - series 3 | 0.0000000000000000 | neg | 0.011379153390988 | UCAA2000 |
| 19-11-2013 - series 3 | 0.0103467947734699 | neg | 0.021290185224612 | UCAA2000 |
| 19-11-2013 - series 3 | 0.0000000000000000 | neg | 0.010632642211590 | UCAA2000 |
| 19-11-2013 - series 3 | 0.0015394843508045 | neg | 0.014876524843797 | UCAA2000 |
| 19-11-2013 - series 3 | 0.0000000000000000 | neg | 0.005550660792952 | UCAA2000 |
| 19-11-2013 - series 3 | 0.0000000000000000 | neg | 0.010440593025684 | UCAA2000 |
| 19-11-2013 - series 3 | 0.5920148998665210 | pos | 0.205338299193048 | UCAA2000 |
| 19-11-2013 - series 3 | 0.0000000000000000 | neg | 0.012016342225427 | UCAA2000 |
| 19-11-2013 - series 3 | 0.0001996778453812 | neg | 0.013381506757661 | UCAA2000 |
| 19-11-2013 - series 3 | 44.0436386817185000 | pos | 4.098576700052710 | UCAA2000 |
| 19-11-2013 - series 3 | 0.0022558755816020 | neg | 0.015527950310559 | UCAA2000 |
| 19-11-2013 - series 3 | 0.0000000000000000 | neg | 0.008371470030137 | UCAA2000 |
| 19-11-2013 - series 3 | 0.0000000000000000 | neg | 0.012217470983506 | UCAA2000 |
| 19-11-2013 - series 3 | 0.0000000000000000 | neg | 0.011357183418512 | UCAA2000 |
| 19-11-2013 - series 3 | 0.0000000000000000 | neg | 0.011349449551697 | UCAA2000 |
| 20-11-2013 - series 1 | 0.0372197822756346 | neg | 0.030440479790344 | UCAA2000 |
| 20-11-2013 - series 1 | 0.0000000000000000 | neg | 0.005988621618924 | UCAA2000 |
| 20-11-2013 - series 1 | 0.0083550809008777 | neg | 0.018126148193509 | UCAA2000 |
| 20-11-2013 - series 1 | 0.0087834157197497 | neg | 0.018340611353712 | UCAA2000 |
| 20-11-2013 - series 1 | 0.0159902689597999 | neg | 0.021727202896960 | UCAA2000 |
| 20-11-2013 - series 1 | 0.0344741366750521 | neg | 0.029379157427938 | UCAA2000 |
| 20-11-2013 - series 1 | 0.0232400725440094 | neg | 0.024856596558317 | UCAA2000 |
| 20-11-2013 - series 1 | 0.0167845652511755 | neg | 0.022080963532954 | UCAA2000 |
| 20-11-2013 - series 1 | 0.0000000000000000 | neg | 0.010593220338983 | UCAA2000 |
| 20-11-2013 - series 1 | 0.1078873682890080 | neg | 0.054690018265821 | UCAA2000 |
| 20-11-2013 - series 1 | 0.0255998086319531 | neg | 0.025834476451760 | UCAA2000 |
| 20-11-2013 - series 1 | 0.0154907566920708 | neg | 0.021503109518288 | UCAA2000 |
| 20-11-2013 - series 1 | 0.0000000000000000 | neg | 0.002803738317757 | UCAA2000 |
| 20-11-2013 - series 1 | 0.0354846711018904 | neg | 0.029771499004456 | UCAA2000 |

| | | | | |
|---|---|---|---|---|
| 20-11-2013 - series 1 | 0.0392913166257806 | neg | 0.031231813773036 | UCAA2000 |
| 20-11-2013 - series 1 | 0.0939296027780694 | neg | 0.050221298888989 | UCAA2000 |
| 20-11-2013 - series 1 | 0.0667968976424379 | neg | 0.041157294213529 | UCAA2000 |
| 20-11-2013 - series 1 | 0.0056988112674396 | neg | 0.016745973645681 | UCAA2000 |
| 20-11-2013 - series 1 | 0.0000000000000000 | neg | 0.009140767824497 | UCAA2000 |
| 20-11-2013 - series 1 | 0.0000000000000000 | neg | 0.009711566475673 | UCAA2000 |
| 20-11-2013 - series 1 | 0.0119012976977321 | neg | 0.019850802882792 | UCAA2000 |
| 20-11-2013 - series 1 | 0.0127714733189169 | neg | 0.020258767449779 | UCAA2000 |
| 20-11-2013 - series 1 | 0.3613482873840780 | indecisive | 0.124934328044552 | UCAA2000 |
| 20-11-2013 - series 1 | 0.0000000000000000 | neg | 0.011178180192265 | UCAA2000 |
| 20-11-2013 - series 1 | 0.0337387976122274 | neg | 0.029092350376536 | UCAA2000 |
| 20-11-2013 - series 1 | 0.0782399719839854 | neg | 0.045049229054431 | UCAA2000 |
| 15-11-2013 - series 2 | 0.1022388358214630 | neg | 0.014120304998588 | UCAA2000 |
| 15-11-2013 - series 2 | 0.1139969344559710 | neg | 0.014898688915375 | UCAA2000 |
| 15-11-2013 - series 2 | 0.1890411334963520 | neg | 0.020907247845920 | UCAA2000 |
| 15-11-2013 - series 2 | 0.1911238230036300 | neg | 0.021097046413502 | UCAA2000 |
| 15-11-2013 - series 2 | 0.0000000000000000 | neg | 0.009888262632256 | UCAA2000 |

| | | | | |
|---|---|---|---|---|
| 16-11-2013 - series 1 | 0.3967493541926330 | indecisive | 0.123591421301345 | UCAA2000 |
| 16-11-2013 - series 1 | 0.0000000000000000 | neg | 0.012236503856041 | UCAA2000 |
| 16-11-2013 - series 1 | 0.0000000000000000 | neg | 0.001438848920863 | UCAA2000 |
| 16-11-2013 - series 1 | 0.0000000000000000 | neg | 0.010551463268963 | UCAA2000 |
| | | | | |
| 16-11-2013 - series 1 | 0.0092896590393694 | neg | 0.025998258489862 | UCAA2000 |
| 16-11-2013 - series 1 | 0.0000000000000000 | neg | 0.001047419539135 | UCAA2000 |
| 16-11-2013 - series 1 | 0.0050300291320118 | neg | 0.024345867267371 | UCAA2000 |
| 16-11-2013 - series 1 | 0.0000000000000000 | neg | 0.016129032258065 | UCAA2000 |
| 16-11-2013 - series 1 | 0.0000000000000000 | neg | 0.020940649496081 | UCAA2000 |
| 16-11-2013 - series 1 | 0.0040906739642143 | neg | 0.023959978936282 | UCAA2000 |
| 16-11-2013 - series 1 | 0.0000000000000000 | neg | 0.019733470015377 | UCAA2000 |
| 16-11-2013 - series 1 | 0.0000000000000000 | neg | 0.017130387343153 | UCAA2000 |
| | | | | |
| 16-11-2013 - series 1 | 0.0633323956411304 | neg | 0.043123991083096 | UCAA2000 |
| 16-11-2013 - series 1 | 0.0000000000000000 | neg | 0.001124567474048 | UCAA2000 |
| | | | | |
| 16-11-2013 - series 1 | 0.0000000000000000 | neg | 0.016518557239937 | UCAA2000 |
| 16-11-2013 - series 1 | 0.0932284018550548 | neg | 0.051492770207011 | UCAA2000 |
| 16-11-2013 - series 1 | 0.0000000000000000 | neg | 0.008833922261484 | UCAA2000 |
| 16-11-2013 - series 1 | 0.5104903062952080 | pos | 0.147518035964251 | UCAA2000 |
| | | | | |
| 16-11-2013 - series 1 | 0.0147173233134440 | neg | 0.027962841324951 | UCAA2000 |
| 16-11-2013 - series 1 | 0.0000000000000000 | neg | 0.004264825345248 | UCAA2000 |
| 16-11-2013 - series 1 | 0.0000000000000000 | neg | 0.014013150803061 | UCAA2000 |
| 16-11-2013 - series 1 | 0.7481991438721470 | pos | 0.194359756097561 | UCAA2000 |
| 16-11-2013 - series 1 | 0.0000000000000000 | neg | 0.000363901018923 | UCAA2000 |
| 16-11-2013 - series 1 | 0.0390845697997283 | neg | 0.035912240184758 | UCAA2000 |
| 16-11-2013 - series 1 | 0.2077128356853400 | indecisive | 0.080662341435073 | UCAA2000 |
| 16-11-2013 - series 1 | 0.0190528209195208 | neg | 0.029461077844311 | UCAA2000 |
| 16-11-2013 - series 1 | 0.0808907676584062 | neg | 0.048094512195122 | UCAA2000 |
| 16-11-2013 - series 1 | 0.0000000000000000 | neg | 0.016765053128690 | UCAA2000 |
| | | | | |
| 16-11-2013 - series 1 | 0.0000000000000000 | neg | 0.014722256587901 | UCAA2000 |
| 16-11-2013 - series 1 | 30.3243593343285000 | pos | 1.895593869731800 | UCAA2000 |
| 16-11-2013 - series 1 | 0.0799647340977021 | neg | 0.047836538461539 | UCAA2000 |
| 16-11-2013 - series 1 | 0.0594640073125939 | neg | 0.042003989205679 | UCAA2000 |
| 16-11-2013 - series 1 | 0.0856492716602525 | neg | 0.049413526328924 | UCAA2000 |
| 16-11-2013 - series 1 | 999.0000000000000000 | pos | 6.086185819070900 | UCAA2000 |
| 16-11-2013 - series 1 | 0.2607663319339040 | indecisive | 0.093212185996793 | UCAA2000 |
| 16-11-2013 - series 1 | 0.0219672322666441 | neg | 0.030442035029191 | UCAA2000 |
| 16-11-2013 - series 1 | 0.1391559957576140 | neg | 0.063627299918427 | UCAA2000 |
| 16-11-2013 - series 2 | 0.0000000000000000 | neg | 0.006193748185425 | UCAA2000 |
| 16-11-2013 - series 2 | 0.0000000000000000 | neg | 0.003715978708446 | UCAA2000 |
| | | | | |
| 16-11-2013 - series 2 | 999.0000000000000000 | pos | 4.115997450605480 | UCAA2000 |
| 16-11-2013 - series 2 | 0.0000000000000000 | neg | 0.021592574499267 | UCAA2000 |
| 16-11-2013 - series 2 | 0.0000000000000000 | neg | 0.018336483931947 | UCAA2000 |
| | | | | |
| 16-11-2013 - series 2 | 0.0000000000000000 | neg | 0.003226314132830 | UCAA2000 |

| | | | | |
|---|---|---|---|---|
| 16-11-2013 - series 2 | 0.0000000000000000 | neg | 0.011475566606101 | UCAA2000 |
| 16-11-2013 - series 2 | 0.0000000000000000 | neg | 0.016832440703902 | UCAA2000 |
| 16-11-2013 - series 2 | 0.0293687450090812 | neg | 0.032860040567951 | UCAA2000 |
| 16-11-2013 - series 2 | 0.0000000000000000 | neg | 0.020334695466044 | UCAA2000 |
| 16-11-2013 - series 2 | 0.0000000000000000 | neg | 0.015547106041403 | UCAA2000 |
| 16-11-2013 - series 2 | 0.9914039033183790 | pos | 0.238916506036319 | UCAA2000 |
| 16-11-2013 - series 2 | 0.5461321910039220 | pos | 0.154791419639970 | UCAA2000 |
| 16-11-2013 - series 2 | 999.0000000000000000 | pos | 2.663682277318640 | UCAA2000 |
| 16-11-2013 - series 2 | 999.0000000000000000 | pos | 4.688373951258490 | UCAA2000 |
| 16-11-2013 - series 2 | 0.0448788009454207 | neg | 0.037681420715767 | UCAA2000 |
| 16-11-2013 - series 2 | 6.0327039445648800 | pos | 0.850263492640378 | UCAA2000 |
| 16-11-2013 - series 2 | 0.0006889656244021 | neg | 0.022416325268609 | UCAA2000 |
| 16-11-2013 - series 2 | 0.0854643779855585 | neg | 0.049362477231330 | UCAA2000 |
| 16-11-2013 - series 2 | 0.0000000000000000 | neg | 0.010844370860927 | UCAA2000 |
| 16-11-2013 - series 2 | 0.0000000000000000 | neg | 0.004940267672685 | UCAA2000 |
| 16-11-2013 - series 2 | 0.0000000000000000 | neg | 0.008666597055445 | UCAA2000 |
| 16-11-2013 - series 2 | 0.0000000000000000 | neg | 0.006470537485981 | UCAA2000 |
| 16-11-2013 - series 2 | 999.0000000000000000 | pos | 5.124051593323220 | UCAA2000 |
| 16-11-2013 - series 2 | 0.0000000000000000 | neg | 0.004729288975864 | UCAA2000 |
| 16-11-2013 - series 2 | 0.0264390642020533 | neg | 0.031914137465349 | UCAA2000 |
| 16-11-2013 - series 2 | 0.0510956086563055 | neg | 0.039544753086420 | UCAA2000 |
| 16-11-2013 - series 2 | 0.0229513844016500 | neg | 0.030769230769231 | UCAA2000 |
| 16-11-2013 - series 2 | 0.0000000000000000 | neg | 0.004045492710480 | UCAA2000 |
| 16-11-2013 - series 2 | 0.0195326801318832 | neg | 0.029623905203503 | UCAA2000 |
| 16-11-2013 - series 2 | 0.0145134033856537 | neg | 0.027891030392884 | UCAA2000 |
| 16-11-2013 - series 2 | 0.0486063022786621 | neg | 0.038802660753880 | UCAA2000 |
| 16-11-2013 - series 2 | 0.0071632175718319 | neg | 0.025189816882537 | UCAA2000 |
| 16-11-2013 - series 2 | 0.0615006816522292 | neg | 0.042594902869364 | UCAA2000 |
| 16-11-2013 - series 2 | 0.0171341107071949 | neg | 0.028804347826087 | UCAA2000 |
| 16-11-2013 - series 2 | 0.0000000000000000 | neg | 0.008826041567809 | UCAA2000 |
| 16-11-2013 - series 2 | 0.0316659736323192 | neg | 0.033592749482810 | UCAA2000 |
| 18-11-2013 - series 1 | 0.0000000000000000 | neg | 0.023307436182020 | UCAA2000 |
| 18-11-2013 - series 1 | 0.0084653002532038 | neg | 0.034413159753759 | UCAA2000 |
| 18-11-2013 - series 1 | 25.2770596123226000 | pos | 3.320571254937710 | UCAA2000 |

| | | | | |
|---|---|---|---|---|
| 14-11-2013 - series 1 | 0.0457099508833964 | neg | 0.029010733971570 | UCAA2000 |
| | | | | |
| 14-11-2013 - series 1 | 0.0000000000000000 | neg | 0.018878610534265 | UCAA2000 |
| 14-11-2013 - series 1 | 0.0000000000000000 | neg | 0.005642279480910 | UCAA2000 |
| 14-11-2013 - series 1 | 0.0000000000000000 | neg | 0.005754685958566 | UCAA2000 |
| 14-11-2013 - series 1 | 0.2406862004400310 | indecisive | 0.071218939542164 | UCAA2000 |
| 14-11-2013 - series 1 | 0.0000000000000000 | neg | 0.014517639712769 | UCAA2000 |
| 14-11-2013 - series 1 | 0.0000000000000000 | neg | 0.013421017313112 | UCAA2000 |
| 14-11-2013 - series 1 | 0.1151483070106410 | neg | 0.043718166383701 | UCAA2000 |
| 14-11-2013 - series 1 | 0.0000000000000000 | neg | 0.006144971156258 | UCAA2000 |
| 14-11-2013 - series 1 | 0.0000000000000000 | neg | 0.016405828386400 | UCAA2000 |
| 14-11-2013 - series 1 | 0.0000000000000000 | neg | 0.008796927270475 | UCAA2000 |
| 14-11-2013 - series 1 | 0.0202104801800264 | neg | 0.023827824750192 | UCAA2000 |
| 14-11-2013 - series 1 | 0.0000000000000000 | neg | 0.016963528413910 | UCAA2000 |
| | | | | |
| 14-11-2013 - series 1 | 0.0480045441914888 | neg | 0.029485570890841 | UCAA2000 |
| 14-11-2013 - series 1 | 7.2536206745694100 | pos | 1.455952171619480 | UCAA2000 |
| 14-11-2013 - series 1 | 0.0713363019999853 | neg | 0.034366976101126 | UCAA2000 |
| 14-11-2013 - series 1 | 0.0679745476460086 | neg | 0.033658420551855 | UCAA2000 |
| 14-11-2013 - series 1 | 0.3050149642023980 | indecisive | 0.085535378835316 | UCAA2000 |
| 14-11-2013 - series 1 | 0.0000000000000000 | neg | 0.000104865771812 | UCAA2000 |
| 14-11-2013 - series 1 | 0.0000000000000000 | neg | 0.016733524355301 | UCAA2000 |
| 15-11-2013 - series 1 | 0.0000000000000000 | neg | 0.030704697986577 | UCAA2000 |
| 15-11-2013 - series 1 | 0.0000000000000000 | neg | 0.012545477355413 | UCAA2000 |
| 15-11-2013 - series 1 | 0.0000000000000000 | neg | 0.012020342117430 | UCAA2000 |
| 15-11-2013 - series 1 | 0.0000000000000000 | neg | 0.032486845115534 | UCAA2000 |
| | | | | |
| 15-11-2013 - series 1 | 1.7973089377137200 | pos | 0.366280174871697 | UCAA2000 |
| 15-11-2013 - series 1 | 0.4315974032894820 | pos | 0.130730659025788 | UCAA2000 |
| 15-11-2013 - series 1 | 0.0000000000000000 | neg | 0.018214936247723 | UCAA2000 |
| 15-11-2013 - series 1 | 0.0000000000000000 | neg | 0.001461835647906 | UCAA2000 |
| 15-11-2013 - series 1 | 0.0000000000000000 | neg | 0.001830065359477 | UCAA2000 |
| 15-11-2013 - series 1 | 0.0000000000000000 | neg | 0.020817732514602 | UCAA2000 |
| 15-11-2013 - series 1 | 0.0000000000000000 | neg | 0.019908466819222 | UCAA2000 |
| 15-11-2013 - series 1 | 0.0514693638134125 | neg | 0.047218155197657 | UCAA2000 |
| 15-11-2013 - series 1 | 0.0000000000000000 | neg | 0.006663314055821 | UCAA2000 |
| 15-11-2013 - series 1 | 0.0000000000000000 | neg | 0.001787924632100 | UCAA2000 |
| 15-11-2013 - series 1 | 0.0000000000000000 | neg | 0.027216748768473 | UCAA2000 |
| | | | | |
| 15-11-2013 - series 1 | 0.0000000000000000 | neg | 0.003453237410072 | UCAA2000 |

| | | | | |
|---|---|---|---|---|
| 15-11-2013 - series 1 | 0.0982123352953936 | neg | 0.058664002283757 | UCAA2000 |
| 15-11-2013 - series 1 | 0.0000000000000000 | neg | 0.032538303585532 | UCAA2000 |
| 15-11-2013 - series 1 | 0.0000000000000000 | neg | 0.027772634697278 | UCAA2000 |
| 15-11-2013 - series 1 | 0.0257351340148334 | neg | 0.040531561461794 | UCAA2000 |
| 15-11-2013 - series 1 | 0.0000000000000000 | neg | 0.012815583749840 | UCAA2000 |
| 15-11-2013 - series 1 | 0.0467612579130007 | neg | 0.046022353714661 | UCAA2000 |
| 15-11-2013 - series 1 | 0.0000000000000000 | neg | 0.025819777949910 | UCAA2000 |
| 15-11-2013 - series 1 | 0.0658754525861155 | neg | 0.050820256776034 | UCAA2000 |
| 15-11-2013 - series 1 | 0.0000000000000000 | neg | 0.001932633903920 | UCAA2000 |
| 15-11-2013 - series 1 | 0.0000000000000000 | neg | 0.004174045029092 | UCAA2000 |
| 15-11-2013 - series 1 | 3.2800552330354700 | pos | 0.568209500609013 | UCAA2000 |
| 15-11-2013 - series 1 | 0.0000000000000000 | neg | 0.000586510263930 | UCAA2000 |
| 15-11-2013 - series 1 | 999.0000000000000000 | pos | 2.650640024976580 | UCAA2000 |
| 15-11-2013 - series 1 | 2.1084158431223300 | pos | 0.412267890673899 | UCAA2000 |
| 15-11-2013 - series 2 | 0.0346823162694182 | neg | 0.010734269727076 | UCAA2000 |
| 15-11-2013 - series 2 | 0.0000000000000000 | neg | 0.001271006919927 | UCAA2000 |
| 15-11-2013 - series 2 | 0.7172759700631330 | pos | 0.092414133926365 | UCAA2000 |
| 15-11-2013 - series 2 | 0.3781911739321170 | indecisive | 0.042049934296978 | UCAA2000 |
| 15-11-2013 - series 2 | 0.4493819718223390 | pos | 0.051620770246926 | UCAA2000 |
| 15-11-2013 - series 2 | 0.3826652052363870 | indecisive | 0.042629939334317 | UCAA2000 |
| 15-11-2013 - series 2 | 0.2963756835668990 | indecisive | 0.032029339853301 | UCAA2000 |
| 15-11-2013 - series 2 | 1.7705066857184600 | pos | 0.262119967132293 | UCAA2000 |
| 15-11-2013 - series 2 | 0.2991679644616380 | indecisive | 0.032351566152408 | UCAA2000 |
| 15-11-2013 - series 2 | 0.1477519773723510 | neg | 0.017391304347826 | UCAA2000 |
| 15-11-2013 - series 2 | 0.0000000000000000 | neg | 0.002357182473655 | UCAA2000 |
| 15-11-2013 - series 2 | 0.0000000000000000 | neg | 0.004054836841089 | UCAA2000 |
| 15-11-2013 - series 2 | 0.1718440582826830 | neg | 0.019384540828689 | UCAA2000 |
| 15-11-2013 - series 2 | 0.2306321626234700 | indecisive | 0.024905023216547 | UCAA2000 |
| 15-11-2013 - series 2 | 0.1185265749429000 | neg | 0.015211439002130 | UCAA2000 |
| 15-11-2013 - series 2 | 0.1061853136444570 | neg | 0.014376078205865 | UCAA2000 |
| 15-11-2013 - series 2 | 0.3946009697788590 | indecisive | 0.044192091941442 | UCAA2000 |
| 15-11-2013 - series 2 | 0.0000000000000000 | neg | 0.008316008316008 | UCAA2000 |
| 15-11-2013 - series 2 | 0.0000000000000000 | neg | 0.006161007667032 | UCAA2000 |
| 15-11-2013 - series 2 | 999.0000000000000000 | pos | 2.368453105968330 | UCAA2000 |
| 15-11-2013 - series 2 | 0.2421888141324120 | indecisive | 0.026089579248982 | UCAA2000 |
| 15-11-2013 - series 2 | 999.0000000000000000 | pos | 2.124483775811210 | UCAA2000 |
| 15-11-2013 - series 2 | 0.0000000000000000 | neg | 0.005244308007163 | UCAA2000 |
| 15-11-2013 - series 2 | 0.3915709294094550 | indecisive | 0.043793503480278 | UCAA2000 |
| 15-11-2013 - series 2 | 0.0000000000000000 | neg | 0.009987016878059 | UCAA2000 |
| 15-11-2013 - series 2 | 0.0876053789846014 | neg | 0.013222266296443 | UCAA2000 |
| 14-11-2013 - series 1 | 0.5915010272883980 | pos | 0.150047483380817 | UCAA2000 |

| | | | | |
|---|---|---|---|---|
| 15-11-2013 - series 2 | 0.1071965682075850 | neg | 0.014442518775274 | UCAA2000 |
| 15-11-2013 - series 2 | 0.0000000000000000 | neg | 0.008460698689956 | UCAA2000 |
| | | | | |
| 15-11-2013 - series 2 | 2.6362782689059500 | pos | 0.369640576537128 | UCAA2000 |
| 15-11-2013 - series 2 | 999.0000000000000000 | pos | 3.617086453369640 | UCAA2000 |
| 15-11-2013 - series 2 | 0.5545989034291720 | pos | 0.066902698083262 | UCAA2000 |

| | | | | |
|---|---|---|---|---|
| 20-11-2013 - series 1 | 0.0000000000000000 | neg | 0.009357162908206 | UCAA2000 |
| 20-11-2013 - series 1 | 0.1324284142900730 | neg | 0.062300918118793 | UCAA2000 |
| | | | | |
| 20-11-2013 - series 1 | 0.0314893743883874 | neg | 0.028207819469955 | UCAA2000 |
| 20-11-2013 - series 1 | 0.1057362412777250 | neg | 0.054008567931457 | UCAA2000 |
| 20-11-2013 - series 1 | 0.0074409156842547 | neg | 0.017661603673614 | UCAA2000 |
| 20-11-2013 - series 1 | 0.0139495125984820 | neg | 0.020803183791606 | UCAA2000 |
| | | | | |
| 20-11-2013 - series 1 | 0.0568452950055024 | neg | 0.037672666387610 | UCAA2000 |
| 20-11-2013 - series 1 | 0.0147823735792190 | neg | 0.021183053557154 | UCAA2000 |

| | | | | |
|---|---|---|---|---|
| 20-11-2013 - series 1 | 0.0956368291056169 | neg | 0.050774096886965 | UCAA2000 |
| 20-11-2013 - series 1 | 0.0336920370398218 | neg | 0.029074074074074 | UCAA2000 |
| 20-11-2013 - series 1 | 17.8673784063627000 | pos | 2.436565364206460 | UCAA2000 |
| 20-11-2013 - series 2 | 0.1052627431089740 | neg | 0.053858229682406 | UCAA2000 |
| 20-11-2013 - series 2 | 0.1414129299025280 | neg | 0.065020231617134 | UCAA2000 |
| 20-11-2013 - series 2 | 0.8166854704320990 | pos | 0.230290456431535 | UCAA2000 |
| 20-11-2013 - series 2 | 0.0003019885698340 | neg | 0.013336889837290 | UCAA2000 |
| 20-11-2013 - series 2 | 0.0000000000000000 | neg | 0.011866143076660 | UCAA2000 |
| 20-11-2013 - series 2 | 0.0000000000000000 | neg | 0.012143290831815 | UCAA2000 |
| 20-11-2013 - series 2 | 0.0000000000000000 | neg | 0.006578947368421 | UCAA2000 |
| 20-11-2013 - series 2 | 0.0520588607928903 | neg | 0.035957759657800 | UCAA2000 |
| 20-11-2013 - series 2 | 0.0000000000000000 | neg | 0.002668523030514 | UCAA2000 |
| 20-11-2013 - series 2 | 0.1488707157564670 | neg | 0.067253299811439 | UCAA2000 |
| 20-11-2013 - series 2 | 0.1137122513202190 | neg | 0.056522940851299 | UCAA2000 |
| 20-11-2013 - series 2 | 0.0022771324405132 | neg | 0.014765724915708 | UCAA2000 |
| 20-11-2013 - series 2 | 0.0587435561589458 | neg | 0.038345410628019 | UCAA2000 |
| 20-11-2013 - series 2 | 0.0304408054871042 | neg | 0.027791612710429 | UCAA2000 |
| 20-11-2013 - series 2 | 0.0255707077956853 | neg | 0.025822519334120 | UCAA2000 |
| 20-11-2013 - series 2 | 0.0010016498443102 | neg | 0.013900472616069 | UCAA2000 |
| 20-11-2013 - series 2 | 0.0014516809110832 | neg | 0.014220705346985 | UCAA2000 |
| 20-11-2013 - series 2 | 0.0430989239284409 | neg | 0.032666991711360 | UCAA2000 |
| 20-11-2013 - series 2 | 0.0042521098380885 | neg | 0.015946420028704 | UCAA2000 |
| 20-11-2013 - series 2 | 0.1425789970612450 | neg | 0.065370789508721 | UCAA2000 |
| 20-11-2013 - series 2 | 0.0003737349594822 | neg | 0.013401232913428 | UCAA2000 |
| 20-11-2013 - series 2 | 0.0018804244972857 | neg | 0.014509246088194 | UCAA2000 |
| 20-11-2013 - series 2 | 0.2167746141288700 | indecisive | 0.086760563380282 | UCAA2000 |
| 20-11-2013 - series 2 | 0.0000000000000000 | neg | 0.012210012210012 | UCAA2000 |
| 20-11-2013 - series 2 | 0.0066237618228187 | neg | 0.017237569060774 | UCAA2000 |
| 20-11-2013 - series 2 | 0.0017078508326299 | neg | 0.014394702749388 | UCAA2000 |
| 20-11-2013 - series 2 | 0.0029545290459281 | neg | 0.015186028853455 | UCAA2000 |
| 20-11-2013 - series 2 | 0.0073756663231959 | neg | 0.017628065276202 | UCAA2000 |
| 20-11-2013 - series 2 | 0.0399078317191590 | neg | 0.031465848042978 | UCAA2000 |
| 20-11-2013 - series 2 | 0.2845494575522700 | indecisive | 0.105109900432087 | UCAA2000 |
| 20-11-2013 - series 2 | 0.0894913242612466 | neg | 0.048775468203334 | UCAA2000 |
| 20-11-2013 - series 2 | 0.0570158040452202 | neg | 0.037733260153677 | UCAA2000 |
| 20-11-2013 - series 2 | 0.1090003857413800 | neg | 0.055041628122109 | UCAA2000 |
| 20-11-2013 - series 2 | 0.0004192259293353 | neg | 0.013440860215054 | UCAA2000 |
| 20-11-2013 - series 2 | 0.0000000000000000 | neg | 0.008495478213209 | UCAA2000 |
| 20-11-2013 - series 2 | 0.1846405162900520 | neg | 0.077696843565730 | UCAA2000 |

| | | | | |
|---|---|---|---|---|
| 20-11-2013 - series 2 | 0.0000000000000000 | neg | 0.003071410289224 | UCAA2000 |
| 20-11-2013 - series 3 | 0.0525330951364209 | neg | 0.036128912417572 | UCAA2000 |
| 20-11-2013 - series 3 | 0.6726358613833640 | pos | 0.198622417031935 | UCAA2000 |
| 20-11-2013 - series 3 | 0.1097559585504700 | neg | 0.055279943302622 | UCAA2000 |
| 20-11-2013 - series 3 | 0.0001040385037443 | neg | 0.013140604467806 | UCAA2000 |
| | | | | |
| 20-11-2013 - series 3 | 0.0505719493005763 | neg | 0.035419274092616 | UCAA2000 |
| 20-11-2013 - series 3 | 0.0000000000000000 | neg | 0.010592098294672 | UCAA2000 |
| 20-11-2013 - series 3 | 0.3936163032437840 | indecisive | 0.133018230925051 | UCAA2000 |
| 20-11-2013 - series 3 | 0.0000000000000000 | neg | 0.009720062208398 | UCAA2000 |
| 20-11-2013 - series 3 | 0.0074166459992199 | neg | 0.017649135192376 | UCAA2000 |
| 20-11-2013 - series 3 | 0.0000000000000000 | neg | 0.010052271813430 | UCAA2000 |
| 20-11-2013 - series 3 | 7.5876848420759800 | pos | 1.292005053239490 | UCAA2000 |
| | | | | |
| 20-11-2013 - series 3 | 0.0000000000000000 | neg | 0.008677730773403 | UCAA2000 |
| 20-11-2013 - series 3 | 0.0000000000000000 | neg | 0.009195095948827 | UCAA2000 |
| | | | | |
| 20-11-2013 - series 3 | 0.0009264622786800 | neg | 0.013844662882459 | UCAA2000 |
| 20-11-2013 - series 3 | 0.1396950873473660 | neg | 0.064502819419612 | UCAA2000 |
| | | | | |
| 20-11-2013 - series 3 | 0.0356147228113241 | neg | 0.029821843532146 | UCAA2000 |
| | | | | |
| 20-11-2013 - series 3 | 0.0055630315999784 | neg | 0.016672642524202 | UCAA2000 |
| 20-11-2013 - series 3 | 0.0668159373306278 | neg | 0.041163866020074 | UCAA2000 |
| 20-11-2013 - series 3 | 1.0307523482143800 | pos | 0.275391849529781 | UCAA2000 |
| | | | | |
| 20-11-2013 - series 3 | 0.0000000000000000 | neg | 0.009575792396821 | UCAA2000 |
| 20-11-2013 - series 3 | 0.0000000000000000 | neg | 0.010680337498665 | UCAA2000 |
| 20-11-2013 - series 3 | 0.0724139784511375 | neg | 0.043081761006289 | UCAA2000 |
| 20-11-2013 - series 3 | 0.0652293693034280 | neg | 0.040615058324496 | UCAA2000 |
| 20-11-2013 - series 3 | 0.0017350462126397 | neg | 0.014412886816448 | UCAA2000 |
| 20-11-2013 - series 3 | 0.0650689709344724 | neg | 0.040559440559441 | UCAA2000 |
| 20-11-2013 - series 3 | 0.1517906363791250 | neg | 0.068121942039895 | UCAA2000 |
| 20-11-2013 - series 3 | 0.0203883781868247 | neg | 0.023650385604113 | UCAA2000 |
| | | | | |
| 20-11-2013 - series 3 | 0.0261727576650546 | neg | 0.026069395997797 | UCAA2000 |
| 20-11-2013 - series 3 | 0.0399071503921173 | neg | 0.031465589769477 | UCAA2000 |
| 20-11-2013 - series 3 | 0.0868251926251323 | neg | 0.047900650502661 | UCAA2000 |

| | | | | |
|---|---|---|---|---|
| 20-11-2013 - series 3 | 0.0000000000000000 | neg | 0.010930156301235 | UCAA2000 |
| 20-11-2013 - series 3 | 0.0000000000000000 | neg | 0.011309658448315 | UCAA2000 |
| 20-11-2013 - series 3 | 0.0433453734497530 | neg | 0.032759070738711 | UCAA2000 |
| 20-11-2013 - series 3 | 0.0000000000000000 | neg | 0.011726078799250 | UCAA2000 |
| 20-11-2013 - series 3 | 0.0000000000000000 | neg | 0.010793308148948 | UCAA2000 |
| 20-11-2013 - series 3 | 0.0000000000000000 | neg | 0.012000480019201 | UCAA2000 |
| 20-11-2013 - series 3 | 0.0242120807441436 | neg | 0.025261478461266 | UCAA2000 |
| | | | | |
| | | | | |
| 21-11-2013 - series 1 | 0.0000000000000000 | neg | 0.017292062943109 | UCAA2000 |
| | | | | |
| | | | | |
| 21-11-2013 - series 1 | 0.0000000000000000 | neg | 0.006251288040118 | UCAA2000 |
| 21-11-2013 - series 1 | 0.0146162631047263 | neg | 0.038363913264196 | UCAA2000 |
| 21-11-2013 - series 1 | 0.0593348016802237 | neg | 0.052048192771084 | UCAA2000 |
| 21-11-2013 - series 1 | 0.0267031596582485 | neg | 0.042427078458899 | UCAA2000 |
| 21-11-2013 - series 1 | 0.0000000000000000 | neg | 0.021075268817204 | UCAA2000 |
| 21-11-2013 - series 1 | 0.0000000000000000 | neg | 0.013713658804169 | UCAA2000 |
| 21-11-2013 - series 1 | 0.0000000000000000 | neg | 0.020019772614928 | UCAA2000 |
| | | | | |
| 21-11-2013 - series 1 | 0.0000000000000000 | neg | 0.012504689258472 | UCAA2000 |
| 21-11-2013 - series 1 | 0.0147656001123100 | neg | 0.038417147568013 | UCAA2000 |
| 21-11-2013 - series 1 | 0.0000000000000000 | neg | 0.018122508155129 | UCAA2000 |
| 21-11-2013 - series 1 | 0.0322485783676767 | neg | 0.044169813479803 | UCAA2000 |
| 21-11-2013 - series 1 | 0.0000000000000000 | neg | 0.030303030303030 | UCAA2000 |
| 21-11-2013 - series 1 | 0.0000000000000000 | neg | 0.013414149443561 | UCAA2000 |
| 21-11-2013 - series 1 | 0.0000000000000000 | neg | 0.027234107115764 | UCAA2000 |
| 21-11-2013 - series 1 | 0.0000000000000000 | neg | 0.018318373328448 | UCAA2000 |
| 21-11-2013 - series 1 | 0.0000000000000000 | neg | 0.010864841373316 | UCAA2000 |
| | | | | |
| 21-11-2013 - series 1 | 0.0000000000000000 | neg | 0.016793282686925 | UCAA2000 |

| | | | | |
|---|---|---|---|---|
| 21-11-2013 - series 1 | 2.0429875680343900 | pos | 0.384859096214774 | UCAA2000 |
| 21-11-2013 - series 1 | 0.0000000000000000 | neg | 0.010531858873091 | UCAA2000 |
| 21-11-2013 - series 1 | 0.0000000000000000 | neg | 0.013755158184319 | UCAA2000 |
| 21-11-2013 - series 1 | 0.0000000000000000 | neg | 0.004912194522903 | UCAA2000 |
| 21-11-2013 - series 1 | 0.0518877541898221 | neg | 0.049964106245513 | UCAA2000 |
| 21-11-2013 - series 1 | 0.0000000000000000 | neg | 0.011450818733539 | UCAA2000 |
| 21-11-2013 - series 1 | 0.0000000000000000 | neg | 0.011317338162064 | UCAA2000 |
| 21-11-2013 - series 1 | 0.0000000000000000 | neg | 0.012263919548688 | UCAA2000 |
| 21-11-2013 - series 1 | 0.0000000000000000 | neg | 0.000490316253984 | UCAA2000 |
| 21-11-2013 - series 1 | 0.0000000000000000 | neg | 0.013290802764487 | UCAA2000 |
| 21-11-2013 - series 1 | 0.0494398313949258 | neg | 0.049267360373752 | UCAA2000 |
| 21-11-2013 - series 1 | 0.0000000000000000 | neg | 0.013704262025490 | UCAA2000 |
| 21-11-2013 - series 1 | 0.0349211057237080 | neg | 0.044989775051125 | UCAA2000 |
| 21-11-2013 - series 1 | 0.0164512561262739 | neg | 0.039011540391370 | UCAA2000 |
| 21-11-2013 - series 1 | 0.0000000000000000 | neg | 0.013201320132013 | UCAA2000 |
| 21-11-2013 - series 1 | 0.0203348882660435 | neg | 0.040341237709659 | UCAA2000 |
| 21-11-2013 - series 1 | 0.0000000000000000 | neg | 0.029541230558545 | UCAA2000 |
| 21-11-2013 - series 1 | 0.0000000000000000 | neg | 0.019405897658283 | UCAA2000 |
| 21-11-2013 - series 1 | 0.0000000000000000 | neg | 0.022921593596507 | UCAA2000 |
| 21-11-2013 - series 2 | 0.1309554208414620 | neg | 0.070300560366786 | UCAA2000 |
| 21-11-2013 - series 2 | 0.0190000000000000 | neg | 0.040000000000000 | UCAA2000 |
| 21-11-2013 - series 2 | 0.0000000000000000 | neg | 0.009002520705798 | UCAA2000 |
| 21-11-2013 - series 2 | 0.0000000000000000 | neg | 0.005538600655589 | UCAA2000 |
| 21-11-2013 - series 2 | 0.0159090843999282 | neg | 0.038821620272126 | UCAA2000 |
| 21-11-2013 - series 2 | 0.1569063994681950 | neg | 0.076393831553974 | UCAA2000 |
| 21-11-2013 - series 2 | 0.2001109185289780 | indecisive | 0.086139298892989 | UCAA2000 |
| 21-11-2013 - series 2 | 0.0898995952940305 | neg | 0.060163611887750 | UCAA2000 |
| 21-11-2013 - series 2 | 0.0153606261939941 | neg | 0.038628301143082 | UCAA2000 |
| 21-11-2013 - series 2 | 0.1584148663750890 | neg | 0.076741931015376 | UCAA2000 |
| 21-11-2013 - series 2 | 0.1960889931154450 | neg | 0.085250190777281 | UCAA2000 |
| 21-11-2013 - series 2 | 0.0000000000000000 | neg | 0.012138868657441 | UCAA2000 |
| 21-11-2013 - series 2 | 0.0630475889904873 | neg | 0.053069053708440 | UCAA2000 |
| 21-11-2013 - series 2 | 0.0676712938598611 | neg | 0.054325114918512 | UCAA2000 |
| 21-11-2013 - series 2 | 0.0181630841737363 | neg | 0.039603960396040 | UCAA2000 |
| 21-11-2013 - series 2 | 0.0095011292595147 | neg | 0.036471123237835 | UCAA2000 |
| 21-11-2013 - series 2 | 0.0225170583217118 | neg | 0.041067761806982 | UCAA2000 |
| 21-11-2013 - series 2 | 0.0503820752637774 | neg | 0.049536279323513 | UCAA2000 |
| 21-11-2013 - series 2 | 0.0000000000000000 | neg | 0.027021657063382 | UCAA2000 |
| 21-11-2013 - series 2 | 0.0000000000000000 | neg | 0.027673649393605 | UCAA2000 |
| 21-11-2013 - series 2 | 0.1201901046631430 | neg | 0.067708333333333 | UCAA2000 |
| 21-11-2013 - series 2 | 0.0661879707568413 | neg | 0.053923928743380 | UCAA2000 |

| | | | | |
|---|---|---|---|---|
| 21-11-2013 - series 2 | 0.0000000000000000 | neg | 0.021932681867535 | UCAA2000 |
| 21-11-2013 - series 2 | 0.0069846146463764 | neg | 0.035474411124775 | UCAA2000 |
| 21-11-2013 - series 2 | 0.0381744598647843 | neg | 0.045972701364932 | UCAA2000 |
| 21-11-2013 - series 2 | 0.0659193654577579 | neg | 0.053851104913109 | UCAA2000 |
| 21-11-2013 - series 2 | 0.0836340985419195 | neg | 0.058548009367682 | UCAA2000 |
| 21-11-2013 - series 2 | 0.0000000000000000 | neg | 0.003867591855307 | UCAA2000 |
| 21-11-2013 - series 2 | 0.0000000000000000 | neg | 0.023371365476629 | UCAA2000 |
| 21-11-2013 - series 2 | 0.0109208908129674 | neg | 0.037011745424747 | UCAA2000 |
| 21-11-2013 - series 2 | 0.0000000000000000 | neg | 0.025029216984807 | UCAA2000 |
| 21-11-2013 - series 2 | 0.0492032724974669 | neg | 0.049199699215813 | UCAA2000 |
| 21-11-2013 - series 2 | 0.0000000000000000 | neg | 0.017035775127768 | UCAA2000 |
| 21-11-2013 - series 2 | 0.0198845226806749 | neg | 0.040189573459716 | UCAA2000 |
| 21-11-2013 - series 2 | 0.0000000000000000 | neg | 0.004772977603720 | UCAA2000 |
| 22-11-2013 - series 3 | 999.0000000000000000 | pos | 4.646628131021190 | UCAA2000 |
| 22-11-2013 - series 3 | 4.4290185564789300 | pos | 0.446371347785108 | UCAA2000 |

| | | | | |
|---|---|---|---|---|
| 22-11-2013 - series 3 | 0.0000000000000000 | neg | 0.012331976815884 | UCAA2000 |
| 22-11-2013 - series 3 | 0.0140377487984809 | neg | 0.017019959771004 | UCAA2000 |
| 22-11-2013 - series 3 | 0.0000000000000000 | neg | 0.004474898615578 | UCAA2000 |
| 22-11-2013 - series 3 | 0.0000000000000000 | neg | 0.008766662663624 | UCAA2000 |
| 25-11-2013 - series 1 | 0.0000000000000000 | neg | 0.016581473631717 | UCAA2000 |
| 25-11-2013 - series 1 | 0.0000000000000000 | neg | 0.011349449551697 | UCAA2000 |
| 25-11-2013 - series 1 | 0.0000000000000000 | neg | 0.016157760814249 | UCAA2000 |
| 25-11-2013 - series 1 | 0.0605459096352687 | neg | 0.039576531117048 | UCAA2000 |
| 25-11-2013 - series 1 | 0.2557970212370840 | indecisive | 0.070918492982024 | UCAA2000 |
| 25-11-2013 - series 1 | 0.0125927792897323 | neg | 0.030211153221440 | UCAA2000 |
| 25-11-2013 - series 1 | 0.0260936067525078 | neg | 0.033050847457627 | UCAA2000 |
| 25-11-2013 - series 1 | 0.6505258364577770 | pos | 0.125385162360749 | UCAA2000 |
| 25-11-2013 - series 1 | 0.0089467066167813 | neg | 0.029385273587030 | UCAA2000 |
| 25-11-2013 - series 1 | 0.0664741558259164 | neg | 0.040639821445178 | UCAA2000 |
| 25-11-2013 - series 1 | 0.0000000000000000 | neg | 0.017750826901874 | UCAA2000 |
| 25-11-2013 - series 1 | 0.0000000000000000 | neg | 0.026108767529358 | UCAA2000 |
| 25-11-2013 - series 1 | 0.1108342732621660 | neg | 0.048263492581119 | UCAA2000 |
| 25-11-2013 - series 1 | 0.0000000000000000 | neg | 0.019815927873779 | UCAA2000 |
| 25-11-2013 - series 1 | 0.0000000000000000 | neg | 0.000478659752692 | UCAA2000 |
| 25-11-2013 - series 1 | 0.0000000000000000 | neg | 0.015757957768673 | UCAA2000 |
| 25-11-2013 - series 1 | 0.0000000000000000 | neg | 0.026164824401847 | UCAA2000 |
| 25-11-2013 - series 1 | 0.0000000000000000 | neg | 0.012565971349585 | UCAA2000 |
| 25-11-2013 - series 1 | 0.0502397847392895 | neg | 0.037694013303769 | UCAA2000 |
| 25-11-2013 - series 1 | 0.0000000000000000 | neg | 0.017361111111111 | UCAA2000 |
| 25-11-2013 - series 1 | 6.8269297612780000 | pos | 0.740254411161264 | UCAA2000 |
| 25-11-2013 - series 1 | 0.0000000000000000 | neg | 0.016806722689076 | UCAA2000 |
| 25-11-2013 - series 1 | 0.0553842412512067 | neg | 0.038639489808678 | UCAA2000 |
| 25-11-2013 - series 1 | 9.8213274145657100 | pos | 0.980339403973510 | UCAA2000 |
| 25-11-2013 - series 1 | 0.2200852957346680 | indecisive | 0.065553795540392 | UCAA2000 |
| 25-11-2013 - series 1 | 0.1170641294260900 | neg | 0.049297274275980 | UCAA2000 |
| 25-11-2013 - series 1 | 0.0000000000000000 | neg | 0.010147804985661 | UCAA2000 |
| 25-11-2013 - series 1 | 0.3125198438909170 | indecisive | 0.079234564209897 | UCAA2000 |
| 25-11-2013 - series 1 | 63.8789874885217000 | pos | 3.519514967790830 | UCAA2000 |
| 25-11-2013 - series 1 | 0.0467316193676548 | neg | 0.037041972018654 | UCAA2000 |
| 25-11-2013 - series 1 | 0.0000000000000000 | neg | 0.025493421052632 | UCAA2000 |
| 25-11-2013 - series 1 | 0.0000000000000000 | neg | 0.001018675721562 | UCAA2000 |
| 25-11-2013 - series 1 | 0.0207482526952813 | neg | 0.031957390146471 | UCAA2000 |
| 25-11-2013 - series 1 | 0.0000000000000000 | neg | 0.012048192771084 | UCAA2000 |
| 25-11-2013 - series 1 | 0.0201252520637781 | neg | 0.031827694454133 | UCAA2000 |
| 25-11-2013 - series 1 | 15.7187591190263000 | pos | 1.396136314304320 | UCAA2000 |
| 25-11-2013 - series 1 | 0.0000000000000000 | neg | 0.025505257662764 | UCAA2000 |
| 25-11-2013 - series 2 | 0.0000000000000000 | neg | 0.024028584189370 | UCAA2000 |
| 25-11-2013 - series 2 | 0.0000000000000000 | neg | 0.012974479948531 | UCAA2000 |
| 25-11-2013 - series 2 | 0.0000000000000000 | neg | 0.001390374331551 | UCAA2000 |
| 25-11-2013 - series 2 | 0.0127921070558667 | neg | 0.030255314362839 | UCAA2000 |
| 25-11-2013 - series 2 | 0.0616660064039663 | neg | 0.039778449144008 | UCAA2000 |
| 25-11-2013 - series 2 | 0.0569078369400385 | neg | 0.038917236011416 | UCAA2000 |
| 25-11-2013 - series 2 | 0.1209552432799720 | neg | 0.049939212568970 | UCAA2000 |

| | | | | |
|---|---|---|---|---|
| 25-11-2013 - series 2 | 0.0370430658359166 | neg | 0.035205643551178 | UCAA2000 |
| 25-11-2013 - series 2 | 12.2662552432050000 | pos | 1.160627001516940 | UCAA2000 |
| 25-11-2013 - series 2 | 0.0000000000000000 | neg | 0.011389521640091 | UCAA2000 |
| 25-11-2013 - series 2 | 0.0000000000000000 | neg | 0.015195022732711 | UCAA2000 |
| 25-11-2013 - series 2 | 0.0000000000000000 | neg | 0.009462266328179 | UCAA2000 |
| 25-11-2013 - series 2 | 0.1043163057140960 | neg | 0.047173540650942 | UCAA2000 |
| 25-11-2013 - series 2 | 0.0138902144879195 | neg | 0.030497031777442 | UCAA2000 |
| 25-11-2013 - series 2 | 0.0472503831849754 | neg | 0.037138787138787 | UCAA2000 |
| 25-11-2013 - series 2 | 0.0333570938734575 | neg | 0.034491046749189 | UCAA2000 |
| 25-11-2013 - series 2 | 0.3626862076734090 | indecisive | 0.086414445399828 | UCAA2000 |
| 25-11-2013 - series 2 | 0.1154482564324420 | neg | 0.049029859416754 | UCAA2000 |
| 25-11-2013 - series 2 | 0.0755511046360428 | neg | 0.042244046818243 | UCAA2000 |
| 25-11-2013 - series 2 | 0.0282626190341820 | neg | 0.033485856905158 | UCAA2000 |
| 25-11-2013 - series 2 | 0.0876913863884398 | neg | 0.044350476939719 | UCAA2000 |
| 25-11-2013 - series 2 | 0.0628981136839739 | neg | 0.040000000000000 | UCAA2000 |
| | | | | |
| 25-11-2013 - series 2 | 0.0000000000000000 | neg | 0.003967521682967 | UCAA2000 |
| 25-11-2013 - series 2 | 65.5295029913139000 | pos | 3.570048309178740 | UCAA2000 |
| 25-11-2013 - series 2 | 0.0000000000000000 | neg | 0.003922887245012 | UCAA2000 |
| | | | | |
| 25-11-2013 - series 2 | 0.0000000000000000 | neg | 0.024856181482688 | UCAA2000 |
| | | | | |
| 25-11-2013 - series 2 | 0.1626587163974110 | neg | 0.056662840446624 | UCAA2000 |
| | | | | |
| 25-11-2013 - series 2 | 0.0000000000000000 | neg | 0.005976602238047 | UCAA2000 |
| 25-11-2013 - series 2 | 0.0053718627223170 | neg | 0.028530513328927 | UCAA2000 |
| | | | | |
| 25-11-2013 - series 2 | 0.1390565614153350 | neg | 0.052890638983354 | UCAA2000 |
| 25-11-2013 - series 2 | 0.0000000000000000 | neg | 0.013457139012246 | UCAA2000 |
| 25-11-2013 - series 2 | 0.0000000000000000 | neg | 0.022346368715084 | UCAA2000 |
| 25-11-2013 - series 2 | 0.0890999524754366 | neg | 0.044592264104459 | UCAA2000 |
| 25-11-2013 - series 2 | 0.4029445723467270 | pos | 0.092075184150368 | UCAA2000 |
| 25-11-2013 - series 2 | 0.0000000000000000 | neg | 0.016150740242261 | UCAA2000 |
| 25-11-2013 - series 2 | 0.0000000000000000 | neg | 0.013349700782569 | UCAA2000 |
| 25-11-2013 - series 2 | 0.0000000000000000 | neg | 0.005900816070308 | UCAA2000 |
| 25-11-2013 - series 3 | 0.0000000000000000 | neg | 0.023512123438648 | UCAA2000 |
| 25-11-2013 - series 3 | 0.0000000000000000 | neg | 0.016089362574298 | UCAA2000 |
| 25-11-2013 - series 3 | 0.0080299757185671 | neg | 0.029171210468920 | UCAA2000 |
| 25-11-2013 - series 3 | 0.0000000000000000 | neg | 0.023556821123479 | UCAA2000 |
| 25-11-2013 - series 3 | 0.0491176175447334 | neg | 0.037486116253240 | UCAA2000 |
| 25-11-2013 - series 3 | 0.0000000000000000 | neg | 0.013846579894766 | UCAA2000 |
| 25-11-2013 - series 3 | 0.0653386227984524 | neg | 0.040437158469945 | UCAA2000 |
| 25-11-2013 - series 3 | 0.0589797986656601 | neg | 0.039293379560839 | UCAA2000 |
| 25-11-2013 - series 3 | 0.0101473131087344 | neg | 0.029661366070693 | UCAA2000 |
| 25-11-2013 - series 3 | 0.0590528753156928 | neg | 0.039306613668985 | UCAA2000 |
| 25-11-2013 - series 3 | 0.0725435468223946 | neg | 0.041715490977267 | UCAA2000 |
| | | | | |
| 25-11-2013 - series 3 | 0.0000000000000000 | neg | 0.011132115101869 | UCAA2000 |

| | | | | |
|---|---|---|---|---|
| 27-11-2013 - series 1 | 0.3378977108951290 | indecisive | 0.062265566391598 | UCAA2000 |
| 27-11-2013 - series 1 | 0.0948031767898461 | neg | 0.040488351812632 | UCAA2000 |
| 27-11-2013 - series 1 | 0.0000000000000000 | neg | 0.027474694360458 | UCAA2000 |
| 27-11-2013 - series 1 | 0.0000000000000000 | neg | 0.026429926809434 | UCAA2000 |
| 27-11-2013 - series 1 | 0.0000000000000000 | neg | 0.025280304632960 | UCAA2000 |
| 27-11-2013 - series 1 | 0.0000000000000000 | neg | 0.027501733302519 | UCAA2000 |
| 27-11-2013 - series 1 | 0.0000000000000000 | neg | 0.011371389583807 | UCAA2000 |
| 27-11-2013 - series 1 | 0.0000000000000000 | neg | 0.010154292496374 | UCAA2000 |
| 27-11-2013 - series 1 | 0.0000000000000000 | neg | 0.007992327365729 | UCAA2000 |
| 27-11-2013 - series 1 | 0.0000000000000000 | neg | 0.007044090790504 | UCAA2000 |
| 27-11-2013 - series 1 | 0.0000000000000000 | neg | 0.022855001873361 | UCAA2000 |
| 27-11-2013 - series 1 | 0.0000000000000000 | neg | 0.011610356437943 | UCAA2000 |
| 27-11-2013 - series 1 | 1.3259482880363000 | pos | 0.132400993334205 | UCAA2000 |
| 27-11-2013 - series 1 | 0.3038692045931650 | indecisive | 0.059445290038897 | UCAA2000 |
| 27-11-2013 - series 2 | 12.6470620272964000 | pos | 0.644213023991563 | UCAA2000 |
| | | | | |
| 27-11-2013 - series 2 | 0.0000000000000000 | neg | 0.020218883324059 | UCAA2000 |
| 27-11-2013 - series 2 | 0.0576256046946204 | neg | 0.036568096626406 | UCAA2000 |
| 27-11-2013 - series 2 | 0.0000000000000000 | neg | 0.009263773768893 | UCAA2000 |
| 27-11-2013 - series 2 | 0.0597496908162663 | neg | 0.036801375752365 | UCAA2000 |
| 27-11-2013 - series 2 | 0.0000000000000000 | neg | 0.023497608650447 | UCAA2000 |

| | | | | |
|---|---|---|---|---|
| 27-11-2013 - series 2 | 0.0000000000000000 | neg | 0.014929658340511 | UCAA2000 |
| 27-11-2013 - series 2 | 0.0000000000000000 | neg | 0.025331871879187 | UCAA2000 |
| 27-11-2013 - series 2 | 0.0000000000000000 | neg | 0.019647559246506 | UCAA2000 |
| 27-11-2013 - series 2 | 999.0000000000000000 | pos | 3.923913043478260 | UCAA2000 |
| 27-11-2013 - series 2 | 0.0000000000000000 | neg | 0.025286448044251 | UCAA2000 |
| 27-11-2013 - series 2 | 3.9787435762087700 | pos | 0.281288584577394 | UCAA2000 |
| 27-11-2013 - series 2 | 0.0000000000000000 | neg | 0.024955132039997 | UCAA2000 |
| 27-11-2013 - series 2 | 0.0000000000000000 | neg | 0.013144329896907 | UCAA2000 |
| 27-11-2013 - series 2 | 0.1252190864998910 | neg | 0.043504592712224 | UCAA2000 |
| 27-11-2013 - series 2 | 39.8109190729367000 | pos | 1.371730769230770 | UCAA2000 |
| 27-11-2013 - series 2 | 1.4650929793696000 | pos | 0.141238012205754 | UCAA2000 |
| 27-11-2013 - series 2 | 0.4588179667433980 | pos | 0.071928188388902 | UCAA2000 |
| 27-11-2013 - series 2 | 999.0000000000000000 | pos | 4.656068069861170 | UCAA2000 |
| 27-11-2013 - series 2 | 0.0000000000000000 | neg | 0.026158634031458 | UCAA2000 |
| 27-11-2013 - series 2 | 0.0235394883632084 | neg | 0.032570497985772 | UCAA2000 |
| 27-11-2013 - series 2 | 0.0000000000000000 | neg | 0.020100502512563 | UCAA2000 |
| 27-11-2013 - series 2 | 0.3279262040810440 | indecisive | 0.061444379732091 | UCAA2000 |
| | | | | |
| 27-11-2013 - series 2 | 0.0000000000000000 | neg | 0.022932330827068 | UCAA2000 |
| 27-11-2013 - series 2 | 0.0000000000000000 | neg | 0.016572790294627 | UCAA2000 |
| 27-11-2013 - series 2 | 0.3441225928812550 | indecisive | 0.062776098581725 | UCAA2000 |
| 27-11-2013 - series 2 | 0.0677514189365976 | neg | 0.037668517049960 | UCAA2000 |
| 27-11-2013 - series 2 | 0.0000000000000000 | neg | 0.028543568832550 | UCAA2000 |
| 27-11-2013 - series 2 | 0.0000000000000000 | neg | 0.009154183688909 | UCAA2000 |
| 27-11-2013 - series 2 | 0.0000000000000000 | neg | 0.006964388282596 | UCAA2000 |
| 27-11-2013 - series 2 | 0.0000000000000000 | neg | 0.011329396167289 | UCAA2000 |
| 27-11-2013 - series 2 | 0.0000000000000000 | neg | 0.009162202034319 | UCAA2000 |
| 27-11-2013 - series 2 | 2.3625520079277400 | pos | 0.194820255121763 | UCAA2000 |
| 27-11-2013 - series 2 | 6.6542981416754000 | pos | 0.407239819004525 | UCAA2000 |
| 27-11-2013 - series 2 | 35.7662016761758000 | pos | 1.285375962107760 | UCAA2000 |
| 27-11-2013 - series 2 | 0.0575827063777092 | neg | 0.036563371044434 | UCAA2000 |
| 27-11-2013 - series 2 | 0.0000000000000000 | neg | 0.002005213555244 | UCAA2000 |
| 27-11-2013 - series 3 | 0.0000000000000000 | neg | 0.028693257528649 | UCAA2000 |
| 27-11-2013 - series 3 | 0.0000000000000000 | neg | 0.024640872391737 | UCAA2000 |
| 27-11-2013 - series 3 | 0.0891911296481804 | neg | 0.039915580840521 | UCAA2000 |
| 27-11-2013 - series 3 | 0.0529033996035386 | neg | 0.036044362292052 | UCAA2000 |
| 27-11-2013 - series 3 | 0.0396548123075104 | neg | 0.034531162268389 | UCAA2000 |
| | | | | |
| 27-11-2013 - series 3 | 0.0000000000000000 | neg | 0.015211422295442 | UCAA2000 |
| 27-11-2013 - series 3 | 0.0619582175847148 | neg | 0.037042502951594 | UCAA2000 |
| 27-11-2013 - series 3 | 0.0000000000000000 | neg | 0.012130033964095 | UCAA2000 |
| 27-11-2013 - series 3 | 0.9464595706054680 | pos | 0.107300025886617 | UCAA2000 |
| 27-11-2013 - series 3 | 0.0000000000000000 | neg | 0.023798832510103 | UCAA2000 |
| | | | | |
| 27-11-2013 - series 3 | 0.0000000000000000 | neg | 0.013367196898810 | UCAA2000 |
| 27-11-2013 - series 3 | 0.0000000000000000 | neg | 0.009167583425009 | UCAA2000 |
| 27-11-2013 - series 3 | 0.0000000000000000 | neg | 0.025801474369964 | UCAA2000 |

| | | | | |
|---|---|---|---|---|
| 27-11-2013 - series 3 | 0.0000000000000000 | neg | 0.009594166746618 | UCAA2000 |
| 27-11-2013 - series 3 | 0.0000000000000000 | neg | 0.024539363484087 | UCAA2000 |
| 27-11-2013 - series 3 | 0.0000000000000000 | neg | 0.000235294117647 | UCAA2000 |
| | | | | |
| 27-11-2013 - series 3 | 0.0668040506929129 | neg | 0.037566760206391 | UCAA2000 |
| | | | | |
| 27-11-2013 - series 3 | 0.0000000000000000 | neg | 0.009560229445507 | UCAA2000 |
| 27-11-2013 - series 3 | 0.0007171491154867 | neg | 0.029190294691829 | UCAA2000 |
| | | | | |
| 27-11-2013 - series 3 | 0.0000000000000000 | neg | 0.009807767752060 | UCAA2000 |
| | | | | |
| | | | | |
| | | | | |
| 27-11-2013 - series 3 | 0.0000000000000000 | neg | 0.015353021709368 | UCAA2000 |
| 27-11-2013 - series 3 | 0.3804986227729810 | indecisive | 0.065728838224594 | UCAA2000 |
| | | | | |
| 27-11-2013 - series 3 | 999.0000000000000000 | pos | 3.687695190505930 | UCAA2000 |
| | | | | |
| | | | | |
| 27-11-2013 - series 3 | 0.1383938615335250 | neg | 0.044772093859570 | UCAA2000 |
| 27-11-2013 - series 3 | 26.2551819189250000 | pos | 1.057491767288690 | UCAA2000 |
| 27-11-2013 - series 3 | 0.1207598584397860 | neg | 0.043070686597416 | UCAA2000 |
| 27-11-2013 - series 3 | 0.0000000000000000 | neg | 0.019094333012175 | UCAA2000 |
| 27-11-2013 - series 3 | 11.1923120199012000 | pos | 0.590981644054270 | UCAA2000 |
| 27-11-2013 - series 3 | 0.0000000000000000 | neg | 0.019647186690076 | UCAA2000 |
| 27-11-2013 - series 3 | 0.0000000000000000 | neg | 0.028995167472088 | UCAA2000 |
| 27-11-2013 - series 3 | 0.0500235741536324 | neg | 0.035721257075932 | UCAA2000 |
| 27-11-2013 - series 3 | 999.0000000000000000 | pos | 3.232091690544410 | UCAA2000 |

| | | | | |
|---|---|---|---|---|
| 27-11-2013 - series 3 | 0.0000000000000000 | neg | 0.011603620329543 | UCAA2000 |
| 27-11-2013 - series 3 | 0.0418276598637471 | neg | 0.034784357092591 | UCAA2000 |
| 27-11-2013 - series 3 | 0.0000000000000000 | neg | 0.010770059235326 | UCAA2000 |
| 27-11-2013 - series 3 | 0.0000000000000000 | neg | 0.010905125408942 | UCAA2000 |
| 27-11-2013 - series 3 | 0.3122529765798700 | indecisive | 0.060144927536232 | UCAA2000 |
| 27-11-2013 - series 4 | 0.1056688858612380 | neg | 0.041581902514444 | UCAA2000 |
| 27-11-2013 - series 4 | 0.0000000000000000 | neg | 0.009567546880980 | UCAA2000 |
| 27-11-2013 - series 4 | 0.0000000000000000 | neg | 0.025268636668905 | UCAA2000 |
| 27-11-2013 - series 4 | 1.3444211002089500 | pos | 0.133584291382643 | UCAA2000 |
| 27-11-2013 - series 4 | 0.1085412795757960 | neg | 0.041867833433917 | UCAA2000 |
| 27-11-2013 - series 4 | 0.0000000000000000 | neg | 0.009288500835965 | UCAA2000 |
| 27-11-2013 - series 4 | 0.0000000000000000 | neg | 0.013246787653994 | UCAA2000 |
| 27-11-2013 - series 4 | 1.7322719334778000 | pos | 0.157757234726688 | UCAA2000 |
| 27-11-2013 - series 4 | 0.1752192212449970 | neg | 0.048215777539086 | UCAA2000 |
| 27-11-2013 - series 4 | 0.0551107765392233 | neg | 0.036290086181626 | UCAA2000 |
| 27-11-2013 - series 4 | 0.0000000000000000 | neg | 0.004658385093168 | UCAA2000 |
| 27-11-2013 - series 4 | 0.0202040554413258 | neg | 0.032140691328078 | UCAA2000 |
| 27-11-2013 - series 4 | 0.0544919405482379 | neg | 0.036221361729590 | UCAA2000 |
| 27-11-2013 - series 4 | 0.1800161466107820 | neg | 0.048654970760234 | UCAA2000 |
| 27-11-2013 - series 4 | 0.0199528883461963 | neg | 0.032107882485150 | UCAA2000 |
| 27-11-2013 - series 4 | 0.0000000000000000 | neg | 0.013668671405139 | UCAA2000 |
| 27-11-2013 - series 4 | 0.0000000000000000 | neg | 0.010612331529237 | UCAA2000 |
| 27-11-2013 - series 4 | 0.1779151491527160 | neg | 0.048462852263023 | UCAA2000 |
| 27-11-2013 - series 4 | 0.1025996066356110 | neg | 0.041274957471573 | UCAA2000 |
| 27-11-2013 - series 4 | 0.0000000000000000 | neg | 0.017644511799091 | UCAA2000 |
| 27-11-2013 - series 4 | 0.0000000000000000 | neg | 0.019539249146758 | UCAA2000 |
| 27-11-2013 - series 4 | 2.4378913955811000 | pos | 0.199099239012269 | UCAA2000 |
| 27-11-2013 - series 4 | 0.0000000000000000 | neg | 0.012501562695337 | UCAA2000 |
| 27-11-2013 - series 4 | 0.0000000000000000 | neg | 0.011435105774728 | UCAA2000 |
| 27-11-2013 - series 4 | 4.3290815723146600 | pos | 0.298815753236023 | UCAA2000 |
| 27-11-2013 - series 4 | 0.3392017720091600 | indecisive | 0.062372651120670 | UCAA2000 |
| 27-11-2013 - series 4 | 0.0000000000000000 | neg | 0.010092854259185 | UCAA2000 |
| 27-11-2013 - series 4 | 0.2906596806660750 | indecisive | 0.058336209872282 | UCAA2000 |
| 27-11-2013 - series 4 | 0.0000000000000000 | neg | 0.013755158184319 | UCAA2000 |
| 27-11-2013 - series 4 | 0.0000000000000000 | neg | 0.013812500000000 | UCAA2000 |
| 27-11-2013 - series 4 | 0.7671475474061880 | pos | 0.094802494802495 | UCAA2000 |
| 27-11-2013 - series 4 | 0.0000000000000000 | neg | 0.026311263972485 | UCAA2000 |
| 27-11-2013 - series 4 | 0.1859235619544690 | neg | 0.049193169356102 | UCAA2000 |
| 27-11-2013 - series 4 | 999.0000000000000000 | pos | 4.595254237288140 | UCAA2000 |
| 27-11-2013 - series 4 | 0.2848810619974990 | indecisive | 0.057848355291859 | UCAA2000 |

| | | | | |
|---|---|---|---|---|
| 27-11-2013 - series 4 | 3.1814408223859100 | pos | 0.239914468995011 | UCAA2000 |
| 27-11-2013 - series 4 | 0.3051366446210390 | indecisive | 0.059551268609771 | UCAA2000 |
| 28-11-2013 - series 1 | 0.0000000000000000 | neg | 0.015390279823270 | UCAA2000 |
| 28-11-2013 - series 1 | 0.0162045620012742 | neg | 0.040936265072449 | UCAA2000 |
| 28-11-2013 - series 1 | 0.0000000000000000 | neg | 0.031818677127602 | UCAA2000 |
| 28-11-2013 - series 1 | 0.1018799455998310 | neg | 0.063538016151150 | UCAA2000 |
| 28-11-2013 - series 1 | 0.2278330037860170 | indecisive | 0.093947455936149 | UCAA2000 |
| 28-11-2013 - series 1 | 0.0000000000000000 | neg | 0.016460905349794 | UCAA2000 |
| 28-11-2013 - series 1 | 0.0000000000000000 | neg | 0.015598190609889 | UCAA2000 |
| 28-11-2013 - series 1 | 0.0000000000000000 | neg | 0.028399599064484 | UCAA2000 |
| 28-11-2013 - series 1 | 0.0000000000000000 | neg | 0.017094017094017 | UCAA2000 |
| 28-11-2013 - series 1 | 0.0014863782455640 | neg | 0.036523503550896 | UCAA2000 |
| 28-11-2013 - series 1 | 0.0135941558769363 | neg | 0.040186228865474 | UCAA2000 |
| 28-11-2013 - series 1 | 0.0000000000000000 | neg | 0.015979546180889 | UCAA2000 |
| 28-11-2013 - series 1 | 0.0000000000000000 | neg | 0.034492968171725 | UCAA2000 |
| 28-11-2013 - series 1 | 0.1835886622688860 | neg | 0.083508359237167 | UCAA2000 |
| 28-11-2013 - series 1 | 0.1001410820141720 | neg | 0.063100838617304 | UCAA2000 |
| 28-11-2013 - series 1 | 0.4356993130870020 | pos | 0.140525114155251 | UCAA2000 |
| 28-11-2013 - series 1 | 0.0000000000000000 | neg | 0.022841757859997 | UCAA2000 |
| 28-11-2013 - series 1 | 0.8851093846625350 | pos | 0.231640449438202 | UCAA2000 |
| 28-11-2013 - series 1 | 0.0000000000000000 | neg | 0.009768486861385 | UCAA2000 |
| 28-11-2013 - series 1 | 0.0000000000000000 | neg | 0.022636965683323 | UCAA2000 |
| 28-11-2013 - series 1 | 0.0417733486516849 | neg | 0.047987747809070 | UCAA2000 |
| 28-11-2013 - series 1 | 0.0122206989180490 | neg | 0.039788021534320 | UCAA2000 |
| 28-11-2013 - series 1 | 0.0000000000000000 | neg | 0.005166126418152 | UCAA2000 |
| 28-11-2013 - series 1 | 1.8391820901722200 | pos | 0.396751015307716 | UCAA2000 |
| 28-11-2013 - series 1 | 0.0000000000000000 | neg | 0.016386510201924 | UCAA2000 |
| 28-11-2013 - series 1 | 0.0446017357006959 | neg | 0.048745260877415 | UCAA2000 |
| 28-11-2013 - series 1 | 0.0000000000000000 | neg | 0.011624610150269 | UCAA2000 |
| 28-11-2013 - series 1 | 0.0000000000000000 | neg | 0.028460038986355 | UCAA2000 |
| 28-11-2013 - series 1 | 0.0000000000000000 | neg | 0.006954872362286 | UCAA2000 |
| 28-11-2013 - series 1 | 0.0237874016199171 | neg | 0.043075020610058 | UCAA2000 |
| 28-11-2013 - series 1 | 0.0000000000000000 | neg | 0.012225467921671 | UCAA2000 |
| 28-11-2013 - series 1 | 0.0034479803260351 | neg | 0.037154297420616 | UCAA2000 |
| 28-11-2013 - series 1 | 0.0000000000000000 | neg | 0.028526808953670 | UCAA2000 |
| 28-11-2013 - series 1 | 0.0039772505036761 | neg | 0.037320035389689 | UCAA2000 |
| 28-11-2013 - series 1 | 0.0257551496216195 | neg | 0.043622141997593 | UCAA2000 |
| 28-11-2013 - series 1 | 0.0039784057049045 | neg | 0.037320395596193 | UCAA2000 |
| | | | | |
| 28-11-2013 - series 1 | 0.0000000000000000 | neg | 0.013649586037145 | UCAA2000 |
| | | | | |
| 28-11-2013 - series 2 | 0.5238461757909300 | neg | 0.058635942804238 | UCAA250 |
| 28-11-2013 - series 2 | 0.0567621461440234 | neg | 0.017554858934169 | UCAA250 |
| 28-11-2013 - series 2 | 0.0000000000000000 | neg | 0.009961151509114 | UCAA250 |
| 28-11-2013 - series 2 | 0.0000000000000000 | neg | 0.008256039139741 | UCAA250 |
| | | | | |
| 28-11-2013 - series 2 | 0.6615590762739600 | neg | 0.069101052364651 | UCAA250 |
| 28-11-2013 - series 2 | 0.5855891591562780 | neg | 0.063379420041600 | UCAA250 |
| 28-11-2013 - series 2 | 0.1292388058837350 | neg | 0.025066891987044 | UCAA250 |
| 28-11-2013 - series 2 | 0.3984789263596680 | neg | 0.048694502588961 | UCAA250 |

| | | | | |
|---|---|---|---|---|
| 28-11-2013 - series 2 | 0.0000000000000000 | neg | 0.001093792726278 | UCAA250 |
| 28-11-2013 - series 2 | 0.0045142955652138 | neg | 0.010901558922926 | UCAA250 |
| 28-11-2013 - series 2 | 0.1873340671703680 | neg | 0.030567685589520 | UCAA250 |
| 28-11-2013 - series 2 | 0.0252307333031935 | neg | 0.013824884792627 | UCAA250 |
| 28-11-2013 - series 2 | 0.0324023189626355 | neg | 0.014718869590815 | UCAA250 |
| 28-11-2013 - series 2 | 0.0090962113780706 | neg | 0.011623852144601 | UCAA250 |
| 28-11-2013 - series 2 | 0.0429601313718332 | neg | 0.015979546180889 | UCAA250 |
| 28-11-2013 - series 2 | 0.1041893620376460 | neg | 0.022576093529530 | UCAA250 |

| | | | | |
|---|---|---|---|---|
| 28-11-2013 - series 2 | 0.2344819798690310 | neg | 0.034824079906416 | UCAA250 |
| 28-11-2013 - series 2 | 0.1315609565774350 | neg | 0.025293586269196 | UCAA250 |
| | | | | |
| 28-11-2013 - series 2 | 0.0764247312393451 | neg | 0.019696832168467 | UCAA250 |
| 28-11-2013 - series 2 | 0.0657983828480077 | neg | 0.018552036199095 | UCAA250 |
| 28-11-2013 - series 2 | 0.0000000000000000 | neg | 0.003008811519450 | UCAA250 |
| 28-11-2013 - series 2 | 0.0000000000000000 | neg | 0.001676797317124 | UCAA250 |
| 28-11-2013 - series 2 | 0.1307871557218390 | neg | 0.025218118800048 | UCAA250 |
| 28-11-2013 - series 2 | 0.3301319766134100 | neg | 0.043058239028430 | UCAA250 |
| | | | | |
| 28-11-2013 - series 2 | 0.0000000000000000 | neg | 0.004165753124315 | UCAA250 |
| 28-11-2013 - series 2 | 0.3156610192476820 | neg | 0.041841004184100 | UCAA250 |

| | | | | |
|---|---|---|---|---|
| 28-11-2013 - series 2 | 0.2002841127839270 | neg | 0.031752692684292 | UCAA250 |
| 28-11-2013 - series 2 | 0.1896671807021690 | neg | 0.030782146355114 | UCAA250 |
| 28-11-2013 - series 2 | 0.1595985631844490 | neg | 0.027982779827798 | UCAA250 |
| 28-11-2013 - series 2 | 0.0193558099028570 | neg | 0.013061650992686 | UCAA250 |
| 28-11-2013 - series 2 | 0.0169268554707626 | neg | 0.012735608762099 | UCAA250 |
| 28-11-2013 - series 2 | 0.3371450490392130 | neg | 0.043644942494839 | UCAA250 |
| 28-11-2013 - series 3 | 0.0576910210860942 | neg | 0.017658484901995 | UCAA250 |
| 28-11-2013 - series 3 | 1.2398169173746100 | indecisive | 0.109714529186195 | UCAA250 |
| 28-11-2013 - series 3 | 0.8435565672395520 | indecisive | 0.082378133266154 | UCAA250 |
| 28-11-2013 - series 3 | 10.6249102330567000 | pos | 0.583665338645418 | UCAA250 |
| 29-11-2013 - series 1 | 0.3196830843985880 | neg | 0.022773855613755 | UCAA250 |
| 29-11-2013 - series 1 | 33.3562413314074000 | pos | 1.698685269653880 | UCAA250 |
| 29-11-2013 - series 1 | 1.3536345553163300 | indecisive | 0.051306413301663 | UCAA250 |
| 29-11-2013 - series 1 | 25.6302849242012000 | pos | 1.327908587257620 | UCAA250 |
| 29-11-2013 - series 1 | 0.0000000000000000 | neg | 0.017088174982912 | UCAA250 |
| 29-11-2013 - series 1 | 0.0000000000000000 | neg | 0.010207206287639 | UCAA250 |
| 29-11-2013 - series 1 | 11.7217464493919000 | pos | 0.567872781746946 | UCAA250 |
| 29-11-2013 - series 1 | 0.1836101461572680 | neg | 0.020259319286872 | UCAA250 |
| 29-11-2013 - series 1 | 0.9824108016475180 | indecisive | 0.039657968450538 | UCAA250 |
| 29-11-2013 - series 1 | 0.6456272375770720 | neg | 0.030311614730878 | UCAA250 |
| 29-11-2013 - series 1 | 0.0552566917636746 | neg | 0.018446781036709 | UCAA250 |
| 29-11-2013 - series 1 | 0.0000000000000000 | neg | 0.016101131071191 | UCAA250 |
| 29-11-2013 - series 1 | 0.2203573746650590 | neg | 0.020889910173386 | UCAA250 |
| 29-11-2013 - series 1 | 1.2123476322710100 | indecisive | 0.046727594339623 | UCAA250 |
| 29-11-2013 - series 1 | 11.9163345450859000 | pos | 0.578840284842319 | UCAA250 |
| 29-11-2013 - series 1 | 0.6223414794004640 | neg | 0.029717373233583 | UCAA250 |
| 29-11-2013 - series 1 | 0.9839133712266570 | indecisive | 0.039702504648365 | UCAA250 |
| 29-11-2013 - series 1 | 0.0000000000000000 | neg | 0.016869918699187 | UCAA250 |
| 29-11-2013 - series 1 | 0.0000000000000000 | neg | 0.014249073810202 | UCAA250 |
| 29-11-2013 - series 1 | 0.4957678624033330 | neg | 0.026625560538117 | UCAA250 |
| 29-11-2013 - series 1 | 128.4615338217460000 | pos | 3.797510069571580 | UCAA250 |
| 29-11-2013 - series 1 | 0.0000000000000000 | neg | 0.013280212483400 | UCAA250 |
| 29-11-2013 - series 1 | 1.6866445489260900 | pos | 0.062717770034843 | UCAA250 |

| | | | | |
|---|---|---|---|---|
| 29-11-2013 - series 1 | 0.0000000000000000 | neg | 0.013041210224309 | UCAA250 |
| 29-11-2013 - series 1 | 0.9099579263293340 | indecisive | 0.037538279166255 | UCAA250 |
| | | | | |
| 29-11-2013 - series 1 | 39.8503066648025000 | pos | 1.975622968580710 | UCAA250 |
| 29-11-2013 - series 1 | 0.0000000000000000 | neg | 0.001675905633621 | UCAA250 |
| 29-11-2013 - series 1 | 0.0000000000000000 | neg | 0.015128593040847 | UCAA250 |
| 29-11-2013 - series 1 | 0.0000000000000000 | neg | 0.014664906877841 | UCAA250 |
| | | | | |
| 29-11-2013 - series 1 | 0.0000000000000000 | neg | 0.012235409274440 | UCAA250 |
| | | | | |
| | | | | |
| 29-11-2013 - series 1 | 0.4234913485572960 | neg | 0.024975024975025 | UCAA250 |
| 29-11-2013 - series 1 | 0.6833191451993600 | neg | 0.031289111389237 | UCAA250 |
| 29-11-2013 - series 1 | 0.0000000000000000 | neg | 0.014222727919215 | UCAA250 |
| | | | | |
| 29-11-2013 - series 1 | 0.1304903718215550 | neg | 0.019425019425019 | UCAA250 |
| 29-11-2013 - series 1 | 0.3959766758959730 | neg | 0.024371069182390 | UCAA250 |
| 29-11-2013 - series 1 | 2.6790903147314100 | pos | 0.100872800114466 | UCAA250 |
| 29-11-2013 - series 1 | 0.5616559057837360 | neg | 0.028204894914021 | UCAA250 |
| 29-11-2013 - series 1 | 0.5412665087920330 | neg | 0.027708943455733 | UCAA250 |
| 29-11-2013 - series 1 | 0.0000000000000000 | neg | 0.002457956015524 | UCAA250 |
| 29-11-2013 - series 1 | 8.1293850752054700 | pos | 0.368591473286562 | UCAA250 |
| 29-11-2013 - series 1 | 1.3923044861078500 | indecisive | 0.052588331963846 | UCAA250 |
| 29-11-2013 - series 1 | 0.9939305191356140 | indecisive | 0.040000000000000 | UCAA250 |
| 29-11-2013 - series 1 | 0.0000000000000000 | neg | 0.016460905349794 | UCAA250 |
| 29-11-2013 - series 1 | 1.2512192215494200 | indecisive | 0.047970479704797 | UCAA250 |
| 29-11-2013 - series 1 | 0.5640914025382860 | neg | 0.028264556246467 | UCAA250 |
| 29-11-2013 - series 1 | 0.0631248661751838 | neg | 0.018534734384121 | UCAA250 |
| | | | | |
| 29-11-2013 - series 1 | 0.0000000000000000 | neg | 0.009189065012635 | UCAA250 |
| 29-11-2013 - series 1 | 0.0000000000000000 | neg | 0.012233912405187 | UCAA250 |
| 29-11-2013 - series 1 | 4.5636500470715700 | pos | 0.185171724513902 | UCAA250 |
| | | | | |
| 29-11-2013 - series 1 | 0.9864430370084550 | indecisive | 0.039777535969049 | UCAA250 |

| | | | | |
|---|---|---|---|---|
| 25-11-2013 - series 3 | 0.5411471942608760 | pos | 0.110947616244850 | UCAA2000 |
| 25-11-2013 - series 3 | 0.1208481919672790 | neg | 0.049921589127026 | UCAA2000 |
| 25-11-2013 - series 3 | 0.0000000000000000 | neg | 0.020879248347060 | UCAA2000 |
| 25-11-2013 - series 3 | 0.4556727175563970 | pos | 0.099370277078086 | UCAA2000 |
| 25-11-2013 - series 3 | 0.0000000000000000 | neg | 0.016524967069812 | UCAA2000 |
| 25-11-2013 - series 3 | 0.1844753981806730 | neg | 0.060084033613445 | UCAA2000 |
| 25-11-2013 - series 3 | 0.0000000000000000 | neg | 0.017374517374517 | UCAA2000 |
| 25-11-2013 - series 3 | 0.3326838102935510 | indecisive | 0.082138490607980 | UCAA2000 |
| 25-11-2013 - series 3 | 0.0000000000000000 | neg | 0.010620220900595 | UCAA2000 |
| 25-11-2013 - series 3 | 0.0000000000000000 | neg | 0.025855365474339 | UCAA2000 |
| 25-11-2013 - series 3 | 0.0000000000000000 | neg | 0.013606888487297 | UCAA2000 |
| 25-11-2013 - series 3 | 0.0628981136839739 | neg | 0.040000000000000 | UCAA2000 |
| 25-11-2013 - series 3 | 0.0000000000000000 | neg | 0.007413221698936 | UCAA2000 |
| 25-11-2013 - series 3 | 21.1905115481148000 | pos | 1.732938939361930 | UCAA2000 |
| 25-11-2013 - series 3 | 0.3117895212678140 | indecisive | 0.079128905721629 | UCAA2000 |
| 25-11-2013 - series 3 | 0.1851507010859980 | neg | 0.060189023379207 | UCAA2000 |
| 25-11-2013 - series 3 | 1.1007632499941400 | pos | 0.181590967853141 | UCAA2000 |
| 25-11-2013 - series 3 | 0.1213245860291960 | neg | 0.050000000000000 | UCAA2000 |
| 25-11-2013 - series 3 | 0.0355069763989016 | neg | 0.034909034364796 | UCAA2000 |
| 25-11-2013 - series 3 | 15.8063875245983000 | pos | 1.401859189271560 | UCAA2000 |
| 25-11-2013 - series 3 | 3.2087386731794200 | pos | 0.410000000000000 | UCAA2000 |
| 25-11-2013 - series 3 | 54.4240959663710000 | pos | 3.210203512684690 | UCAA2000 |

| | | | | |
|---|---|---|---|---|
| 25-11-2013 - series 3 | 65.2557813579477000 | pos | 3.561733931240660 | UCAA2000 |
| 25-11-2013 - series 3 | 21.8881596936744000 | pos | 1.773161145423920 | UCAA2000 |
| 25-11-2013 - series 3 | 5.8600618904662400 | pos | 0.657122319396190 | UCAA2000 |
| 25-11-2013 - series 4 | 6.8555214229942900 | pos | 0.742666091458154 | UCAA2000 |
| 25-11-2013 - series 4 | 0.1018669370690740 | neg | 0.046761623219565 | UCAA2000 |
| 25-11-2013 - series 4 | 0.0000000000000000 | neg | 0.015948963317384 | UCAA2000 |
| 25-11-2013 - series 4 | 0.0000000000000000 | neg | 0.010986596352450 | UCAA2000 |
| 25-11-2013 - series 4 | 0.0000000000000000 | neg | 0.014417531718570 | UCAA2000 |
| 25-11-2013 - series 4 | 70.4238977515006000 | pos | 3.714465408805030 | UCAA2000 |
| 25-11-2013 - series 4 | 3.9246588207947800 | pos | 0.480015622965760 | UCAA2000 |
| 25-11-2013 - series 4 | 0.0000000000000000 | neg | 0.003759077317386 | UCAA2000 |
| 25-11-2013 - series 4 | 0.0783592591759513 | neg | 0.042735042735043 | UCAA2000 |
| 25-11-2013 - series 4 | 23.4942998167173000 | pos | 1.863684099979000 | UCAA2000 |
| 25-11-2013 - series 4 | 0.6852658717146210 | pos | 0.129893641095270 | UCAA2000 |
| 25-11-2013 - series 4 | 0.7811576742935460 | pos | 0.142168404529656 | UCAA2000 |
| 25-11-2013 - series 4 | 0.0000000000000000 | neg | 0.015064298836497 | UCAA2000 |
| 25-11-2013 - series 4 | 0.0000000000000000 | neg | 0.023566287439053 | UCAA2000 |
| 25-11-2013 - series 4 | 0.0000000000000000 | neg | 0.005195599022005 | UCAA2000 |
| 25-11-2013 - series 4 | 0.0000000000000000 | neg | 0.014149821640904 | UCAA2000 |
| 25-11-2013 - series 4 | 0.0256487119072755 | neg | 0.032961046036502 | UCAA2000 |
| 25-11-2013 - series 4 | 0.0000000000000000 | neg | 0.024593537764972 | UCAA2000 |
| 25-11-2013 - series 4 | 0.1358406785945910 | neg | 0.052370256531505 | UCAA2000 |
| 25-11-2013 - series 4 | 0.0000000000000000 | neg | 0.009317927692881 | UCAA2000 |
| 25-11-2013 - series 4 | 0.0787663825628500 | neg | 0.042806031178124 | UCAA2000 |
| 25-11-2013 - series 4 | 0.7562686546720710 | pos | 0.139005208865284 | UCAA2000 |
| 25-11-2013 - series 4 | 0.0648512039030491 | neg | 0.040350024307244 | UCAA2000 |
| 25-11-2013 - series 4 | 0.0000000000000000 | neg | 0.022716736207696 | UCAA2000 |
| 25-11-2013 - series 4 | 0.1088334175941790 | neg | 0.047929838874159 | UCAA2000 |
| 25-11-2013 - series 4 | 1.6475164459785500 | pos | 0.245138479670006 | UCAA2000 |
| 25-11-2013 - series 4 | 0.0941880544628451 | neg | 0.045461491442543 | UCAA2000 |
| 25-11-2013 - series 4 | 2.9877992776168200 | pos | 0.387785016286645 | UCAA2000 |
| 25-11-2013 - series 4 | 0.0011262769932977 | neg | 0.027393196202532 | UCAA2000 |
| 25-11-2013 - series 4 | 12.3590525611949000 | pos | 1.167231971685590 | UCAA2000 |
| 25-11-2013 - series 4 | 0.1963129609536600 | neg | 0.061917047033142 | UCAA2000 |
| 25-11-2013 - series 4 | 0.0397567661242063 | neg | 0.035725746069098 | UCAA2000 |
| 25-11-2013 - series 4 | 0.0000000000000000 | neg | 0.025393600812595 | UCAA2000 |
| 25-11-2013 - series 4 | 4.7928711325085100 | pos | 0.561433447098976 | UCAA2000 |

| | | | | |
|---|---|---|---|---|
| 25-11-2013 - series 4 | 0.0000000000000000 | neg | 0.019199676637025 | UCAA2000 |
| 25-11-2013 - series 4 | 0.0000000000000000 | neg | 0.019449116904962 | UCAA2000 |
| 25-11-2013 - series 4 | 0.0000000000000000 | neg | 0.007286245353160 | UCAA2000 |
| | | | | |
| 26-11-2013 - series 1 | 63.1619294362567000 | pos | 3.497259201252940 | UCAA2000 |
| 26-11-2013 - series 1 | 3.0233147449776600 | pos | 0.391377005347594 | UCAA2000 |
| | | | | |
| 26-11-2013 - series 1 | 0.0135942443905120 | neg | 0.030432136335971 | UCAA2000 |
| 26-11-2013 - series 1 | 3.4240200429075000 | pos | 0.431358115568642 | UCAA2000 |
| 26-11-2013 - series 1 | 0.0506974043708999 | neg | 0.037778617302607 | UCAA2000 |
| 26-11-2013 - series 1 | 0.0000000000000000 | neg | 0.024044241404184 | UCAA2000 |
| 26-11-2013 - series 1 | 37.2158662255257000 | pos | 2.539779681762550 | UCAA2000 |
| 26-11-2013 - series 1 | 0.8722727595473260 | pos | 0.153623188405797 | UCAA2000 |

| | | | | |
|---|---|---|---|---|
| 26-11-2013 - series 1 | 0.0000000000000000 | neg | 0.023084025854109 | UCAA2000 |
| 26-11-2013 - series 1 | 0.0046608883009928 | neg | 0.028352707683584 | UCAA2000 |

| | | | | |
|---|---|---|---|---|
| 26-11-2013 - series 1 | 0.0000000000000000 | neg | 0.025176233635448 | UCAA2000 |
| 26-11-2013 - series 1 | 0.0000000000000000 | neg | 0.019146084625694 | UCAA2000 |
| 26-11-2013 - series 1 | 0.0000000000000000 | neg | 0.021272069772389 | UCAA2000 |
| 26-11-2013 - series 1 | 0.1009467306683260 | neg | 0.046606529209622 | UCAA2000 |
| 26-11-2013 - series 1 | 0.0628981136839739 | neg | 0.040000000000000 | UCAA2000 |
| 26-11-2013 - series 1 | 999.0000000000000000 | pos | 5.560930232558140 | UCAA2000 |
| 26-11-2013 - series 1 | 0.0000000000000000 | neg | 0.021593608291946 | UCAA2000 |
| 26-11-2013 - series 1 | 0.0000000000000000 | neg | 0.014825796886583 | UCAA2000 |
| 26-11-2013 - series 1 | 0.3785400542622790 | indecisive | 0.088653712699514 | UCAA2000 |
| 26-11-2013 - series 1 | 0.0000000000000000 | neg | 0.017522340984756 | UCAA2000 |
| 26-11-2013 - series 1 | 0.0000000000000000 | neg | 0.021381227282446 | UCAA2000 |
| 26-11-2013 - series 1 | 0.0000000000000000 | neg | 0.016969285593077 | UCAA2000 |
| 26-11-2013 - series 1 | 0.6695520972282500 | pos | 0.127858627858628 | UCAA2000 |
| 26-11-2013 - series 1 | 0.0420276580780938 | neg | 0.036157390996101 | UCAA2000 |
| 26-11-2013 - series 1 | 0.0000000000000000 | neg | 0.015460729746444 | UCAA2000 |
| 26-11-2013 - series 1 | 0.0000000000000000 | neg | 0.017137960582691 | UCAA2000 |
| 26-11-2013 - series 1 | 0.0000000000000000 | neg | 0.015352407536636 | UCAA2000 |
| 26-11-2013 - series 1 | 0.0000000000000000 | neg | 0.007409694350108 | UCAA2000 |
| | | | | |
| 26-11-2013 - series 1 | 0.0000000000000000 | neg | 0.011869436201780 | UCAA2000 |
| 26-11-2013 - series 1 | 0.0318779002320556 | neg | 0.034201408293283 | UCAA2000 |
| 26-11-2013 - series 1 | 0.0000000000000000 | neg | 0.013439053890606 | UCAA2000 |
| 26-11-2013 - series 1 | 4.1383872982366600 | pos | 0.500389029371718 | UCAA2000 |
| 26-11-2013 - series 1 | 0.0513624254638341 | neg | 0.037901386208107 | UCAA2000 |
| 26-11-2013 - series 1 | 0.0000000000000000 | neg | 0.005717202654416 | UCAA2000 |
| 26-11-2013 - series 1 | 0.0000000000000000 | neg | 0.012564392511622 | UCAA2000 |
| 26-11-2013 - series 1 | 0.0000000000000000 | neg | 0.006434519303558 | UCAA2000 |
| 26-11-2013 - series 1 | 0.0000000000000000 | neg | 0.015053439710974 | UCAA2000 |
| 26-11-2013 - series 2 | 57.2225278325294000 | pos | 3.305477528089890 | UCAA2000 |
| 26-11-2013 - series 2 | 0.0000000000000000 | neg | 0.012109469605231 | UCAA2000 |
| | | | | |
| 26-11-2013 - series 2 | 74.6236506125373000 | pos | 3.832333438585410 | UCAA2000 |
| 26-11-2013 - series 2 | 1.1938721053469700 | pos | 0.192718583075626 | UCAA2000 |
| 26-11-2013 - series 2 | 0.2318826813595730 | indecisive | 0.067338371116709 | UCAA2000 |
| | | | | |
| 26-11-2013 - series 2 | 0.0000000000000000 | neg | 0.020907380305248 | UCAA2000 |
| 26-11-2013 - series 2 | 0.0000000000000000 | neg | 0.021381227282446 | UCAA2000 |
| 26-11-2013 - series 2 | 0.0752120795461734 | neg | 0.042184607246760 | UCAA2000 |
| 26-11-2013 - series 2 | 0.0000000000000000 | neg | 0.013625834582368 | UCAA2000 |
| 26-11-2013 - series 2 | 0.0000000000000000 | neg | 0.016786973308712 | UCAA2000 |
| 26-11-2013 - series 2 | 0.0079257187743276 | neg | 0.029146669788131 | UCAA2000 |
| 26-11-2013 - series 2 | 0.0000000000000000 | neg | 0.011726078799250 | UCAA2000 |
| 26-11-2013 - series 2 | 0.0000000000000000 | neg | 0.018867924528302 | UCAA2000 |
| 26-11-2013 - series 2 | 0.0257848056263887 | neg | 0.032988537880906 | UCAA2000 |
| 26-11-2013 - series 2 | 71.3576035789808000 | pos | 3.741136103698060 | UCAA2000 |
| | | | | |
| 26-11-2013 - series 2 | 0.0880474549883253 | neg | 0.044411646316919 | UCAA2000 |
| 26-11-2013 - series 2 | 0.0000000000000000 | neg | 0.014771048744461 | UCAA2000 |
| 26-11-2013 - series 2 | 0.0176888109524501 | neg | 0.031315240083507 | UCAA2000 |

| | | | | |
|---|---|---|---|---|
| 26-11-2013 - series 2 | 0.0000000000000000 | neg | 0.019704433497537 | UCAA2000 |
| 26-11-2013 - series 2 | 0.0000000000000000 | neg | 0.015432098765432 | UCAA2000 |
| 26-11-2013 - series 2 | 0.0372619261120704 | neg | 0.035247771096828 | UCAA2000 |
| 26-11-2013 - series 2 | 5.0780008051715000 | pos | 0.587440758293839 | UCAA2000 |
| 26-11-2013 - series 2 | 0.0000000000000000 | neg | 0.004363526933494 | UCAA2000 |
| 26-11-2013 - series 2 | 0.8564979132780510 | pos | 0.151653504442251 | UCAA2000 |
| 26-11-2013 - series 2 | 0.0000000000000000 | neg | 0.012809017548354 | UCAA2000 |
| 26-11-2013 - series 2 | 0.0000000000000000 | neg | 0.011457378551787 | UCAA2000 |
| 26-11-2013 - series 2 | 0.0000000000000000 | neg | 0.020694752402070 | UCAA2000 |
| 26-11-2013 - series 2 | 0.0000000000000000 | neg | 0.024215420379698 | UCAA2000 |
| 26-11-2013 - series 2 | 0.0017530501903621 | neg | 0.027577791336181 | UCAA2000 |
| 26-11-2013 - series 2 | 0.1452711477130610 | neg | 0.053891716025695 | UCAA2000 |
| 26-11-2013 - series 2 | 72.9432411470464000 | pos | 3.785812356979410 | UCAA2000 |
| 26-11-2013 - series 2 | 0.1437345584825110 | neg | 0.053644740443390 | UCAA2000 |
| 26-11-2013 - series 2 | 1.8496897902568700 | pos | 0.267691737680622 | UCAA2000 |
| 26-11-2013 - series 2 | 2.4296002336327500 | pos | 0.330181637223190 | UCAA2000 |
| | | | | |
| 26-11-2013 - series 2 | 0.0000000000000000 | neg | 0.002120740845469 | UCAA2000 |
| 26-11-2013 - series 2 | 0.0652099342663129 | neg | 0.040414161656647 | UCAA2000 |
| 26-11-2013 - series 2 | 0.0000000000000000 | neg | 0.015042117930205 | UCAA2000 |
| 26-11-2013 - series 3 | 0.3929427670693520 | indecisive | 0.090676586840270 | UCAA2000 |
| 26-11-2013 - series 3 | 0.1369757234501040 | neg | 0.052554112554113 | UCAA2000 |
| 26-11-2013 - series 3 | 0.0506094300051066 | neg | 0.037762360586634 | UCAA2000 |
| 26-11-2013 - series 3 | 0.0000000000000000 | neg | 0.011475122634898 | UCAA2000 |
| 26-11-2013 - series 3 | 1.2408972398547000 | pos | 0.198285846652768 | UCAA2000 |
| 26-11-2013 - series 3 | 999.0000000000000000 | pos | 4.767746156362450 | UCAA2000 |
| 26-11-2013 - series 3 | 0.0563845490225786 | neg | 0.038821954484605 | UCAA2000 |
| 26-11-2013 - series 3 | 1.1027252530335000 | pos | 0.181826941607246 | UCAA2000 |
| 26-11-2013 - series 3 | 0.0000000000000000 | neg | 0.010027073097363 | UCAA2000 |
| | | | | |
| 26-11-2013 - series 3 | 999.0000000000000000 | pos | 4.607517965726920 | UCAA2000 |
| 26-11-2013 - series 3 | 0.0744143553676604 | neg | 0.042044605698431 | UCAA2000 |
| 26-11-2013 - series 3 | 0.5517583348450580 | pos | 0.112365664535437 | UCAA2000 |
| 26-11-2013 - series 3 | 54.5604844995372000 | pos | 3.214924452667280 | UCAA2000 |
| 26-11-2013 - series 3 | 0.6034488269629320 | pos | 0.119218439932835 | UCAA2000 |
| 26-11-2013 - series 3 | 0.0632423291723365 | neg | 0.040061791967044 | UCAA2000 |
| 26-11-2013 - series 3 | 0.0000000000000000 | neg | 0.012565971349585 | UCAA2000 |
| 26-11-2013 - series 3 | 0.0000000000000000 | neg | 0.012121212121212 | UCAA2000 |
| 26-11-2013 - series 3 | 0.0000000000000000 | neg | 0.011014428901861 | UCAA2000 |
| 26-11-2013 - series 3 | 0.0000000000000000 | neg | 0.017040692801937 | UCAA2000 |
| 26-11-2013 - series 3 | 0.0103713724570439 | neg | 0.029712404712405 | UCAA2000 |
| 26-11-2013 - series 3 | 22.1115780908585000 | pos | 1.785924328563030 | UCAA2000 |
| 26-11-2013 - series 3 | 15.5630450788043000 | pos | 1.385937768609250 | UCAA2000 |
| 26-11-2013 - series 3 | 0.0000000000000000 | neg | 0.017912586577502 | UCAA2000 |
| 26-11-2013 - series 3 | 0.0000000000000000 | neg | 0.009664637092877 | UCAA2000 |
| | | | | |
| 26-11-2013 - series 3 | 0.0000000000000000 | neg | 0.008831581736289 | UCAA2000 |
| 26-11-2013 - series 3 | 0.0000000000000000 | neg | 0.017789909638554 | UCAA2000 |

| | | | | |
|---|---|---|---|---|
| 26-11-2013 - series 3 | 0.1853630441751220 | neg | 0.060222025739267 | UCAA2000 |
| 26-11-2013 - series 3 | 0.0000000000000000 | neg | 0.009384384384384 | UCAA2000 |
| 26-11-2013 - series 3 | 0.0000000000000000 | neg | 0.009484066767830 | UCAA2000 |
| 26-11-2013 - series 3 | 0.1573890824064650 | neg | 0.055827424348347 | UCAA2000 |
| 26-11-2013 - series 3 | 0.0000000000000000 | neg | 0.013711235851661 | UCAA2000 |
| 26-11-2013 - series 3 | 0.0835773549279847 | neg | 0.043641287365899 | UCAA2000 |
| 26-11-2013 - series 3 | 0.0000000000000000 | neg | 0.023614663256607 | UCAA2000 |
| 26-11-2013 - series 3 | 0.0143386425213120 | neg | 0.030595013410152 | UCAA2000 |
| 26-11-2013 - series 3 | 0.0255642169665845 | neg | 0.032943967877350 | UCAA2000 |
| 26-11-2013 - series 3 | 0.0000000000000000 | neg | 0.023545587350692 | UCAA2000 |
| 26-11-2013 - series 3 | 0.0327931090353937 | neg | 0.034380816818132 | UCAA2000 |
| 26-11-2013 - series 4 | 0.0000000000000000 | neg | 0.023427644620652 | UCAA2000 |
| 26-11-2013 - series 4 | 0.0379522059598748 | neg | 0.035380428351674 | UCAA2000 |
| 26-11-2013 - series 4 | 0.0030975578580975 | neg | 0.027948070681572 | UCAA2000 |
| 26-11-2013 - series 4 | 0.0156605511206327 | neg | 0.030881576632061 | UCAA2000 |
| 26-11-2013 - series 4 | 0.0738712948440309 | neg | 0.041949184114448 | UCAA2000 |

| | | | | |
|---|---|---|---|---|
| 26-11-2013 - series 4 | 0.0918932192886236 | neg | 0.045070247420117 | UCAA2000 |
| 26-11-2013 - series 4 | 0.0000000000000000 | neg | 0.011334013374136 | UCAA2000 |
| 26-11-2013 - series 4 | 0.0000000000000000 | neg | 0.011562030292519 | UCAA2000 |
| 26-11-2013 - series 4 | 0.0000000000000000 | neg | 0.024488619994238 | UCAA2000 |
| 26-11-2013 - series 4 | 0.0607435128992037 | neg | 0.039612188365651 | UCAA2000 |
| 26-11-2013 - series 4 | 0.2395908226047970 | indecisive | 0.068497629556972 | UCAA2000 |
| 26-11-2013 - series 4 | 0.0217579208244997 | neg | 0.032166508987701 | UCAA2000 |
| 26-11-2013 - series 4 | 0.1633693979908370 | neg | 0.056775225356208 | UCAA2000 |
| 26-11-2013 - series 4 | 0.0976562308269472 | neg | 0.046050381853414 | UCAA2000 |
| 26-11-2013 - series 4 | 5.1935884851182400 | pos | 0.597888675623800 | UCAA2000 |
| 26-11-2013 - series 4 | 0.0000000000000000 | neg | 0.022653721682848 | UCAA2000 |
| 26-11-2013 - series 4 | 0.0000000000000000 | neg | 0.015909674108288 | UCAA2000 |
| 26-11-2013 - series 4 | 0.0000000000000000 | neg | 0.022309027777778 | UCAA2000 |
| 26-11-2013 - series 4 | 0.0000000000000000 | neg | 0.018230088495575 | UCAA2000 |
| 26-11-2013 - series 4 | 0.2029242811215390 | indecisive | 0.062934205149162 | UCAA2000 |
| 26-11-2013 - series 4 | 0.0000000000000000 | neg | 0.010765295246216 | UCAA2000 |
| 26-11-2013 - series 4 | 0.0000000000000000 | neg | 0.013478905512872 | UCAA2000 |
| 26-11-2013 - series 4 | 0.0000000000000000 | neg | 0.014797277300977 | UCAA2000 |
| 26-11-2013 - series 4 | 0.0485253814657224 | neg | 0.037376144487646 | UCAA2000 |
| 26-11-2013 - series 4 | 0.0423747860115526 | neg | 0.036223097908457 | UCAA2000 |
| 26-11-2013 - series 4 | 0.0000000000000000 | neg | 0.015915963711603 | UCAA2000 |
| 26-11-2013 - series 4 | 0.0000000000000000 | neg | 0.012467273407306 | UCAA2000 |
| 26-11-2013 - series 4 | 0.2109889745437240 | indecisive | 0.064168880190130 | UCAA2000 |
| 26-11-2013 - series 4 | 0.1607024975167190 | neg | 0.056353146384006 | UCAA2000 |
| 26-11-2013 - series 4 | 0.0117049119219482 | neg | 0.030013312356287 | UCAA2000 |
| 26-11-2013 - series 4 | 0.0071970992778428 | neg | 0.028973950026582 | UCAA2000 |
| 26-11-2013 - series 4 | 0.0000000000000000 | neg | 0.015551151090105 | UCAA2000 |
| 26-11-2013 - series 4 | 0.0000000000000000 | neg | 0.003208777559259 | UCAA2000 |
| 26-11-2013 - series 4 | 0.0498968018085831 | neg | 0.037630536550234 | UCAA2000 |
| 26-11-2013 - series 4 | 0.2643987830163530 | indecisive | 0.072194777699365 | UCAA2000 |
| 26-11-2013 - series 4 | 0.1722858078658120 | neg | 0.058179723502304 | UCAA2000 |

| | | | | |
|---|---|---|---|---|
| 26-11-2013 - series 4 | 0.0301775237725649 | neg | 0.033866267622273 | UCAA2000 |
| 27-11-2013 - series 1 | 0.2100093730032100 | indecisive | 0.051359084406295 | UCAA2000 |
| 27-11-2013 - series 1 | 0.0000000000000000 | neg | 0.014160294534126 | UCAA2000 |
| 27-11-2013 - series 1 | 0.0000000000000000 | neg | 0.002276751834050 | UCAA2000 |
| 27-11-2013 - series 1 | 0.0000000000000000 | neg | 0.020810363836825 | UCAA2000 |
| 27-11-2013 - series 1 | 0.3421208304822320 | indecisive | 0.062612098483613 | UCAA2000 |
| 27-11-2013 - series 1 | 0.2326529361259060 | indecisive | 0.053357531760436 | UCAA2000 |
| | | | | |
| 27-11-2013 - series 1 | 0.0000000000000000 | neg | 0.013479957431713 | UCAA2000 |
| 27-11-2013 - series 1 | 0.0000000000000000 | neg | 0.012663036596176 | UCAA2000 |
| 27-11-2013 - series 1 | 0.0000000000000000 | neg | 0.011340440009072 | UCAA2000 |
| 27-11-2013 - series 1 | 0.0000000000000000 | neg | 0.010682288077188 | UCAA2000 |
| 27-11-2013 - series 1 | 0.1570135210594060 | neg | 0.046530093695411 | UCAA2000 |
| 27-11-2013 - series 1 | 0.6541511193326760 | pos | 0.086654667009514 | UCAA2000 |
| 27-11-2013 - series 1 | 0.0000000000000000 | neg | 0.014887598630341 | UCAA2000 |
| 27-11-2013 - series 1 | 0.0325406458348336 | neg | 0.033685541407026 | UCAA2000 |
| 27-11-2013 - series 1 | 0.0000000000000000 | neg | 0.014524328249818 | UCAA2000 |
| | | | | |
| 27-11-2013 - series 1 | 0.4298105248549690 | pos | 0.069655338635816 | UCAA2000 |
| 27-11-2013 - series 1 | 0.0303252754572492 | neg | 0.033416328135238 | UCAA2000 |
| 27-11-2013 - series 1 | 0.1349448835330950 | neg | 0.044442283380337 | UCAA2000 |
| 27-11-2013 - series 1 | 0.0000000000000000 | neg | 0.025198210220583 | UCAA2000 |
| 27-11-2013 - series 1 | 0.3636303560043940 | indecisive | 0.064365927143248 | UCAA2000 |
| 27-11-2013 - series 1 | 0.0000000000000000 | neg | 0.012100677637948 | UCAA2000 |
| 27-11-2013 - series 1 | 0.0000000000000000 | neg | 0.018843037497645 | UCAA2000 |
| 27-11-2013 - series 1 | 0.0000000000000000 | neg | 0.013685507048036 | UCAA2000 |

| | | | | |
|---|---|---|---|---|
| 22-11-2013 - series 1 | 0.1059715050453800 | neg | 0.034059493530908 | UCAA2000 |
| 22-11-2013 - series 1 | 0.0020952991149130 | neg | 0.013845239106430 | UCAA2000 |
| 22-11-2013 - series 1 | 0.1167744138421620 | neg | 0.035800900796859 | UCAA2000 |
| 22-11-2013 - series 1 | 0.0215572484649267 | neg | 0.018713737027049 | UCAA2000 |
| 22-11-2013 - series 1 | 0.0697908143571819 | neg | 0.027960234333393 | UCAA2000 |
| 22-11-2013 - series 1 | 0.2901839503494890 | indecisive | 0.060991435245263 | UCAA2000 |
| 22-11-2013 - series 1 | 0.0696857479583640 | neg | 0.027941787941788 | UCAA2000 |
| 22-11-2013 - series 1 | 0.0000000000000000 | neg | 0.010682143157541 | UCAA2000 |
| 22-11-2013 - series 1 | 16.5971252895444000 | pos | 1.205394190871370 | UCAA2000 |
| 22-11-2013 - series 1 | 0.1764379561538200 | neg | 0.044958419958420 | UCAA2000 |
| 22-11-2013 - series 1 | 0.1205730099454480 | neg | 0.036406099738975 | UCAA2000 |
| 22-11-2013 - series 1 | 10.6573523567599000 | pos | 0.869284561592254 | UCAA2000 |
| 22-11-2013 - series 1 | 0.5778692483877150 | pos | 0.097158218125960 | UCAA2000 |
| 22-11-2013 - series 1 | 0.0017155534588908 | neg | 0.013717421124829 | UCAA2000 |
| | | | | |
| 22-11-2013 - series 1 | 0.1153756333547640 | neg | 0.035577140275582 | UCAA2000 |
| 22-11-2013 - series 1 | 1.9645083797833200 | pos | 0.239674315321984 | UCAA2000 |
| 22-11-2013 - series 1 | 0.0000000000000000 | neg | 0.010841283607979 | UCAA2000 |
| 22-11-2013 - series 1 | 0.0718397347947843 | neg | 0.028318960928351 | UCAA2000 |
| | | | | |
| 22-11-2013 - series 1 | 0.0312568532555524 | neg | 0.020747327193818 | UCAA2000 |
| 22-11-2013 - series 1 | 0.0000000000000000 | neg | 0.007406101216717 | UCAA2000 |
| 22-11-2013 - series 1 | 0.1558253417431060 | neg | 0.041870886430472 | UCAA2000 |
| 22-11-2013 - series 1 | 0.0109797950870999 | neg | 0.016286644951140 | UCAA2000 |
| 22-11-2013 - series 1 | 0.0000000000000000 | neg | 0.002674446875760 | UCAA2000 |
| | | | | |
| 22-11-2013 - series 2 | 0.0591472774798046 | neg | 0.026064178127047 | UCAA2000 |
| | | | | |
| 22-11-2013 - series 2 | 9.0982048035108600 | pos | 0.772006275056650 | UCAA2000 |
| 22-11-2013 - series 2 | 0.1976029493455630 | neg | 0.048060075093867 | UCAA2000 |
| 22-11-2013 - series 2 | 0.1503276599613630 | neg | 0.041034993730765 | UCAA2000 |
| 22-11-2013 - series 2 | 0.0572541307442990 | neg | 0.025720675944334 | UCAA2000 |
| 22-11-2013 - series 2 | 0.0146303496210898 | neg | 0.017158544955388 | UCAA2000 |
| 22-11-2013 - series 2 | 0.0000000000000000 | neg | 0.005781799899447 | UCAA2000 |
| 22-11-2013 - series 2 | 0.7420099510804490 | pos | 0.116135662898253 | UCAA2000 |
| 22-11-2013 - series 2 | 0.0000000000000000 | neg | 0.005115612850419 | UCAA2000 |
| 22-11-2013 - series 2 | 0.7791344345724790 | pos | 0.120308917581755 | UCAA2000 |
| 22-11-2013 - series 2 | 0.1234088982644320 | neg | 0.036855638545366 | UCAA2000 |
| 22-11-2013 - series 2 | 0.1593446814358120 | neg | 0.042403127124405 | UCAA2000 |
| 22-11-2013 - series 2 | 0.0036240935377120 | neg | 0.014324595330182 | UCAA2000 |
| | | | | |
| 22-11-2013 - series 2 | 999.0000000000000000 | pos | 4.690084985835690 | UCAA2000 |

| | | | | |
|---|---|---|---|---|
| 22-11-2013 - series 2 | 0.0000000000000000 | neg | 0.006028131279303 | UCAA2000 |
| 22-11-2013 - series 2 | 0.1139386867520290 | neg | 0.035346756152125 | UCAA2000 |
| 22-11-2013 - series 2 | 0.1271200736866040 | neg | 0.037441077441077 | UCAA2000 |
| 22-11-2013 - series 2 | 0.2584640168618780 | indecisive | 0.056662034892697 | UCAA2000 |
| | | | | |
| 22-11-2013 - series 2 | 0.1149884522056720 | neg | 0.035515115937775 | UCAA2000 |
| 22-11-2013 - series 2 | 0.0001346046015888 | neg | 0.013089005235602 | UCAA2000 |
| 22-11-2013 - series 2 | 0.6998028439209040 | pos | 0.111341273951321 | UCAA2000 |
| 22-11-2013 - series 2 | 0.6127973773014530 | pos | 0.101275318829707 | UCAA2000 |
| 22-11-2013 - series 2 | 0.0369677153962123 | neg | 0.021889311495770 | UCAA2000 |
| 22-11-2013 - series 2 | 0.0000000000000000 | neg | 0.010889687465970 | UCAA2000 |
| 22-11-2013 - series 2 | 0.0000000000000000 | neg | 0.011246063877643 | UCAA2000 |
| 22-11-2013 - series 2 | 0.1469426894157490 | neg | 0.040517534899557 | UCAA2000 |
| 22-11-2013 - series 2 | 0.0000000000000000 | neg | 0.011768859597505 | UCAA2000 |
| 22-11-2013 - series 2 | 0.0030629831249683 | neg | 0.014153944020356 | UCAA2000 |
| 22-11-2013 - series 2 | 0.4403979985867160 | pos | 0.080450522928399 | UCAA2000 |
| | | | | |
| 22-11-2013 - series 2 | 0.4224219208485500 | pos | 0.078197345405906 | UCAA2000 |
| 22-11-2013 - series 2 | 0.0000000000000000 | neg | 0.011229646266143 | UCAA2000 |
| 22-11-2013 - series 2 | 40.7288476939673000 | pos | 2.249911504424780 | UCAA2000 |
| 22-11-2013 - series 2 | 0.0000000000000000 | neg | 0.001923539312335 | UCAA2000 |
| 22-11-2013 - series 2 | 0.0000000000000000 | neg | 0.011144544745347 | UCAA2000 |
| | | | | |
| 22-11-2013 - series 2 | 0.0707651791532020 | neg | 0.028131062729036 | UCAA2000 |
| 22-11-2013 - series 2 | 51.0197665758323000 | pos | 2.601487778958550 | UCAA2000 |
| 22-11-2013 - series 2 | 0.2519182608659560 | indecisive | 0.055756600991824 | UCAA2000 |
| 22-11-2013 - series 3 | 0.2763007443698140 | indecisive | 0.059108006448146 | UCAA2000 |
| 22-11-2013 - series 3 | 0.1455630682084760 | neg | 0.040306005937429 | UCAA2000 |
| 22-11-2013 - series 3 | 0.0794261764385878 | neg | 0.029631474103586 | UCAA2000 |
| 22-11-2013 - series 3 | 0.6314227827452900 | pos | 0.103452191687486 | UCAA2000 |
| 22-11-2013 - series 3 | 999.0000000000000000 | pos | 3.978841484564690 | UCAA2000 |
| 22-11-2013 - series 3 | 0.0000000000000000 | neg | 0.011325864828265 | UCAA2000 |
| 22-11-2013 - series 3 | 0.2438723115146340 | indecisive | 0.054637626736763 | UCAA2000 |
| 22-11-2013 - series 3 | 0.0978611893028821 | neg | 0.032730758696248 | UCAA2000 |
| 22-11-2013 - series 3 | 0.0151616351739893 | neg | 0.017281930667757 | UCAA2000 |
| 22-11-2013 - series 3 | 0.0013792427315351 | neg | 0.013599891200870 | UCAA2000 |
| 22-11-2013 - series 3 | 0.6356868308177410 | pos | 0.103948805993445 | UCAA2000 |
| 22-11-2013 - series 3 | 0.0337240232207622 | neg | 0.021244954323348 | UCAA2000 |
| | | | | |
| 22-11-2013 - series 3 | 0.2220498170208640 | indecisive | 0.051567239635996 | UCAA2000 |
| 22-11-2013 - series 3 | 0.0468557623773548 | neg | 0.023794614902943 | UCAA2000 |
| 22-11-2013 - series 3 | 0.0088262330213164 | neg | 0.015748031496063 | UCAA2000 |
| 22-11-2013 - series 3 | 0.2191723374776900 | indecisive | 0.051158301158301 | UCAA2000 |
| 22-11-2013 - series 3 | 0.1548892589322320 | neg | 0.041728948679621 | UCAA2000 |
| 22-11-2013 - series 3 | 0.0061945665992284 | neg | 0.015055706112617 | UCAA2000 |
| 22-11-2013 - series 3 | 0.1394987763827710 | neg | 0.039371772805508 | UCAA2000 |
| 22-11-2013 - series 3 | 0.0070417736927095 | neg | 0.015283509093688 | UCAA2000 |
| 22-11-2013 - series 3 | 0.0551161956190788 | neg | 0.025330262998424 | UCAA2000 |
| 22-11-2013 - series 3 | 11.5434164972869000 | pos | 0.922649818112196 | UCAA2000 |
| 22-11-2013 - series 3 | 0.0126858854048030 | neg | 0.016699754758846 | UCAA2000 |

| | | | | |
|---|---|---|---|---|
| 22-11-2013 - series 3 | 3.0356055013033600 | pos | 0.334155900711279 | UCAA2000 |
| 22-11-2013 - series 3 | 0.8453673829320020 | pos | 0.127659574468085 | UCAA2000 |
| 22-11-2013 - series 3 | 0.3906611724366790 | indecisive | 0.074171212757029 | UCAA2000 |
| 22-11-2013 - series 3 | 0.2847167736443430 | indecisive | 0.060251798561151 | UCAA2000 |
| 22-11-2013 - series 3 | 2.0094600292285200 | pos | 0.243833464257659 | UCAA2000 |
| 22-11-2013 - series 3 | 0.1734714894384900 | neg | 0.044518357426123 | UCAA2000 |
| 22-11-2013 - series 3 | 0.0871437637202325 | neg | 0.030943396226415 | UCAA2000 |
| 22-11-2013 - series 3 | 0.0000000000000000 | neg | 0.002376237623762 | UCAA2000 |

| | | | | |
|---|---|---|---|---|
| 21-11-2013 - series 2 | 0.0000000000000000 | neg | 0.007577092511013 | UCAA2000 |
| 21-11-2013 - series 3 | 0.0422995482407119 | neg | 0.047197370823444 | UCAA2000 |
| 21-11-2013 - series 3 | 0.0000000000000000 | neg | 0.020139707883516 | UCAA2000 |
| 21-11-2013 - series 3 | 0.0225222049993976 | neg | 0.041069459757442 | UCAA2000 |
| 21-11-2013 - series 3 | 0.0000000000000000 | neg | 0.027087430716968 | UCAA2000 |
| 21-11-2013 - series 3 | 0.0359397237806928 | neg | 0.045299253448058 | UCAA2000 |

| | | | | |
|---|---|---|---|---|
| 21-11-2013 - series 3 | 0.0000000000000000 | neg | 0.005920913512370 | UCAA2000 |
| 21-11-2013 - series 3 | 0.0618326154905655 | neg | 0.052736232385234 | UCAA2000 |
| 21-11-2013 - series 3 | 0.0000000000000000 | neg | 0.010995052226498 | UCAA2000 |
| 21-11-2013 - series 3 | 0.0000000000000000 | neg | 0.010900832702498 | UCAA2000 |
| 21-11-2013 - series 3 | 0.0494533151233646 | neg | 0.049271215237414 | UCAA2000 |

| | | | | |
|---|---|---|---|---|
| 21-11-2013 - series 3 | 0.0000000000000000 | neg | 0.020535216502509 | UCAA2000 |
| 21-11-2013 - series 3 | 0.0000000000000000 | neg | 0.025783895990386 | UCAA2000 |
| 21-11-2013 - series 3 | 0.0000000000000000 | neg | 0.010464629552114 | UCAA2000 |
| 21-11-2013 - series 3 | 0.0073510013485671 | neg | 0.035623114411697 | UCAA2000 |
| 21-11-2013 - series 3 | 0.0000000000000000 | neg | 0.031452548270451 | UCAA2000 |
| 21-11-2013 - series 3 | 0.1469450632798280 | neg | 0.074079026542756 | UCAA2000 |

| | | | | |
|---|---|---|---|---|
| 21-11-2013 - series 3 | 0.0021287167057308 | neg | 0.033311702357776 | UCAA2000 |
| 21-11-2013 - series 3 | 0.0000000000000000 | neg | 0.018748197288722 | UCAA2000 |
| 21-11-2013 - series 3 | 0.0000000000000000 | neg | 0.030013226167464 | UCAA2000 |
| 21-11-2013 - series 3 | 0.0000000000000000 | neg | 0.024876181900045 | UCAA2000 |
| 21-11-2013 - series 3 | 0.0000000000000000 | neg | 0.012420817289778 | UCAA2000 |

| | | | | |
|---|---|---|---|---|
| 21-11-2013 - series 3 | 0.0000000000000000 | neg | 0.008631106507854 | UCAA2000 |
| 21-11-2013 - series 3 | 0.0000000000000000 | neg | 0.027865673165255 | UCAA2000 |

| | | | | |
|---|---|---|---|---|
| 21-11-2013 - series 3 | 0.0000000000000000 | neg | 0.024863553668890 | UCAA2000 |
| 21-11-2013 - series 3 | 0.0000000000000000 | neg | 0.020295020295020 | UCAA2000 |
| 21-11-2013 - series 3 | 0.0000000000000000 | neg | 0.001644601595264 | UCAA2000 |

| | | | | |
|---|---|---|---|---|
| 21-11-2013 - series 3 | 0.0000000000000000 | neg | 0.012727504136439 | UCAA2000 |
| 21-11-2013 - series 3 | 0.0271259336684349 | neg | 0.042562113429081 | UCAA2000 |
| 21-11-2013 - series 3 | 0.0000000000000000 | neg | 0.027631044290939 | UCAA2000 |
| 21-11-2013 - series 3 | 0.0000000000000000 | neg | 0.029203653672971 | UCAA2000 |
| 21-11-2013 - series 3 | 0.0000000000000000 | neg | 0.014452955629426 | UCAA2000 |

| | | | | |
|---|---|---|---|---|
| 21-11-2013 - series 3 | 0.0000000000000000 | neg | 0.018733273862623 | UCAA2000 |
| 21-11-2013 - series 3 | 0.0000000000000000 | neg | 0.012023566189732 | UCAA2000 |
| 21-11-2013 - series 3 | 0.0000000000000000 | neg | 0.012436264146251 | UCAA2000 |
| 21-11-2013 - series 3 | 0.0291704778688397 | neg | 0.043209876543210 | UCAA2000 |
| 21-11-2013 - series 3 | 0.0032468020050396 | neg | 0.033854166666667 | UCAA2000 |
| 21-11-2013 - series 3 | 0.0000000000000000 | neg | 0.024544179523142 | UCAA2000 |

| | | | | |
|---|---|---|---|---|
| 21-11-2013 - series 4 | 9.0470400042392200 | pos | 1.134246575342470 | UCAA2000 |
| 21-11-2013 - series 4 | 0.0988622752184635 | neg | 0.062439160061188 | UCAA2000 |
| 21-11-2013 - series 4 | 0.0000000000000000 | neg | 0.029488291413703 | UCAA2000 |
| 21-11-2013 - series 4 | 0.0000000000000000 | neg | 0.016228594185583 | UCAA2000 |
| 21-11-2013 - series 4 | 0.0168276174810632 | neg | 0.039142710472279 | UCAA2000 |
| 21-11-2013 - series 4 | 0.0000000000000000 | neg | 0.010172939979654 | UCAA2000 |
| 21-11-2013 - series 4 | 0.0000000000000000 | neg | 0.026204637531247 | UCAA2000 |
| 21-11-2013 - series 4 | 0.9059610264904660 | pos | 0.216314731020613 | UCAA2000 |
| 21-11-2013 - series 4 | 0.0460251976284794 | neg | 0.048284789644013 | UCAA2000 |
| 21-11-2013 - series 4 | 0.0041707411214997 | neg | 0.034276729559748 | UCAA2000 |
| 21-11-2013 - series 4 | 0.0000000000000000 | neg | 0.012187690432663 | UCAA2000 |
| 21-11-2013 - series 4 | 0.0000000000000000 | neg | 0.012072920439454 | UCAA2000 |

| | | | | |
|---|---|---|---|---|
| 21-11-2013 - series 4 | 0.0000000000000000 | neg | 0.014551083591331 | UCAA2000 |
| 21-11-2013 - series 4 | 0.0000000000000000 | neg | 0.009722222222222 | UCAA2000 |
| 21-11-2013 - series 4 | 0.0000000000000000 | neg | 0.013005063039809 | UCAA2000 |
| 21-11-2013 - series 4 | 0.0000000000000000 | neg | 0.010399838449112 | UCAA2000 |

| | | | | |
|---|---|---|---|---|
| 21-11-2013 - series 4 | 0.0000000000000000 | neg | 0.012152144853567 | UCAA2000 |
| 21-11-2013 - series 4 | 0.0000000000000000 | neg | 0.014074870491643 | UCAA2000 |
| 21-11-2013 - series 4 | 0.6862027680812870 | pos | 0.179319546364243 | UCAA2000 |
| 21-11-2013 - series 4 | 45.3305291849048000 | pos | 3.200955305356530 | UCAA2000 |
| 21-11-2013 - series 4 | 0.0000000000000000 | neg | 0.011709601873536 | UCAA2000 |
| | | | | |
| 21-11-2013 - series 4 | 0.1030934111458340 | neg | 0.063500000000000 | UCAA2000 |
| 21-11-2013 - series 4 | 0.0000000000000000 | neg | 0.015951143924893 | UCAA2000 |
| 21-11-2013 - series 4 | 0.0010073982049449 | neg | 0.032710280373832 | UCAA2000 |
| 21-11-2013 - series 4 | 0.0000000000000000 | neg | 0.017203628401627 | UCAA2000 |
| 21-11-2013 - series 4 | 0.0000000000000000 | neg | 0.009179364787957 | UCAA2000 |
| 21-11-2013 - series 4 | 0.0000000000000000 | neg | 0.009963136395337 | UCAA2000 |
| 21-11-2013 - series 4 | 0.0000000000000000 | neg | 0.031959878060773 | UCAA2000 |
| 21-11-2013 - series 4 | 0.0000000000000000 | neg | 0.011064394777606 | UCAA2000 |
| 21-11-2013 - series 4 | 0.0000000000000000 | neg | 0.024220952931820 | UCAA2000 |
| 21-11-2013 - series 4 | 0.0000000000000000 | neg | 0.026853339453753 | UCAA2000 |
| 21-11-2013 - series 4 | 0.0000000000000000 | neg | 0.018608183803285 | UCAA2000 |
| | | | | |
| 21-11-2013 - series 4 | 0.0000000000000000 | neg | 0.003209739900387 | UCAA2000 |
| 21-11-2013 - series 4 | 0.0703156424428663 | neg | 0.055036344755971 | UCAA2000 |
| 21-11-2013 - series 4 | 0.0000000000000000 | neg | 0.023753638156042 | UCAA2000 |
| 21-11-2013 - series 4 | 0.0000000000000000 | neg | 0.021822149481724 | UCAA2000 |
| 21-11-2013 - series 4 | 0.0000000000000000 | neg | 0.005307855626327 | UCAA2000 |
| 22-11-2013 - series 1 | 0.1970244783542870 | neg | 0.047976147452114 | UCAA2000 |
| 22-11-2013 - series 1 | 0.1420613476861920 | neg | 0.039767441860465 | UCAA2000 |
| 22-11-2013 - series 1 | 2.2749009142239900 | pos | 0.268005354752343 | UCAA2000 |
| 22-11-2013 - series 1 | 0.1805597767782060 | neg | 0.045567616463139 | UCAA2000 |
| 22-11-2013 - series 1 | 0.0533193205506260 | neg | 0.025000000000000 | UCAA2000 |
| 22-11-2013 - series 1 | 0.0200207861618639 | neg | 0.018377754257673 | UCAA2000 |
| 22-11-2013 - series 1 | 0.0703474002246319 | neg | 0.028057869355546 | UCAA2000 |
| 22-11-2013 - series 1 | 0.2154740559858010 | indecisive | 0.050631246712257 | UCAA2000 |
| 22-11-2013 - series 1 | 0.0430139546023408 | neg | 0.023063958130045 | UCAA2000 |
| 22-11-2013 - series 1 | 0.2662084089927290 | indecisive | 0.057727775728514 | UCAA2000 |
| 22-11-2013 - series 1 | 2.7193117178742200 | pos | 0.307157510827445 | UCAA2000 |
| 22-11-2013 - series 1 | 0.0629089680100265 | neg | 0.026740871453780 | UCAA2000 |
| 22-11-2013 - series 1 | 0.0986477297632351 | neg | 0.032860477644101 | UCAA2000 |
| 22-11-2013 - series 1 | 0.0000000000000000 | neg | 0.011107408641564 | UCAA2000 |
