# Supplementary material for: Sensitivity and Specificity of a Urine Circulating Anodic Antigen Test for the Diagnosis of Schistosoma haematobium in Low Endemic Settings
Source: PLoS Negl Trop Dis. 2015 May 14;9(5):e0003752. doi: 10.1371/journal.pntd.0003752 (PMC4431728; doi:10.1371/journal.pntd.0003752)
Supplement: S1 Models — (PDF) [file pntd.0003752.s003.pdf]

Supplementary file 1: Latent class analysis modeling approach

In absence of a true 'gold' standard, we used latent class analysis (LCA) to estimate the sensitivity, specificity, and *S. haematobium* prevalence for reagent strips, quality control urine filtration readings (QCUF), and an up-converting phosphor-lateral flow assay detecting circulating anodic antigen (CAA) in 1.5 ml urine (UCAA2000) [1-3]. LCA is a statistical modeling technique, which examines associations between observed variables (in our case the three diagnostic tests; reagent strip for microhematuria assessment, QCUF for egg identification, and UCAA2000 for antigen detection) that imperfectly measure a non-observable (latent) variable, in our case the *S. haematobium* infection status (i.e., 'infected' or 'non-infected'). LCA is an attractive choice for assessing error in the absence of a 'gold' standard because it does not rely on error-free measures.

Technical details of the LCA model for the evaluation of three diagnostic tests for the detection of *S. haematobium* infection have been described in Ibironke et al. (2012) [3]. Here, we fitted four LCA models with MPlus version 7 [4] with full information maximum likelihood estimation by assuming that indecisive UCAA2000 results data were missing at random. In all four LCA models we included the indecisive results of the UCAA2000 by treating them as 'missing' and not forcing them to be categorized as positive or negative as we wanted to avoid any strong *a priori* assumptions in the modeling approaches [5]. We evaluated the four LCA models on the basis of the lowest Bayesian information criterion (BIC) and Akaike information criterion (AIC) as indications of the best model fit and parsimony in combination with different biological plausible scenarios and tests of assumptions. More technical information about these information criteria are provided in Koukounari et al. (2013) [6]. The assumptions and tested scenarios are discussed in more detail below:

In model 2 (see Table S1), we tested whether the ‘true’ *S. haematobium* prevalence derived by LCA varied significantly between the schools. For this purpose, we allowed for the probability that an individual belongs to the ‘infected’ latent class to vary across different schools by using the parametric multilevel LCA approach as described in Henry et al (2010) [7].

In model 3 (see Table S1), we tested whether sensitivity and specificity varied at different school prevalence levels, and whether the prevalences varied between schools. For this purpose, we fitted a 2-latent class model in which (i) the probability that an individual belongs to the ‘infected’ latent class and (ii) the conditional item response probabilities of all three examined diagnostic tests had random intercepts that varied between schools.

In model 1 (see Table S1), we relied on the assumption of conditional or local independence, which affirms that the results from the three diagnostic tests in the same individual were independent within the real condition of illness. We also tested assumption of conditional or local independence by speculating the standardized residuals for each response pattern from the three diagnostic tests as estimated from this model. No random effects were incorporated. Considering that model 1 showed the lowest BIC and AIC values, this model seemed to provide the best fit for our data.

In Model 4, Table S1, we aimed to further validate the decision of selecting Model 1 as our final model. Here, we followed the approach of Qu et al. (1996) [8] and, in order to ensure unbiased estimates of the model estimated prevalence and diagnostic test accuracies, added another unobserved (latent) continuous variable with a Gaussian distribution of zero mean and unit variance influencing the QCUF and reagent strip results in the ‘infected’ latent class. With this approach, we allowed partial conditional dependence between microhematuria detected with reagent strips and egg counts derived by quality controlled microscopy (considering that microhematuria can be caused by egg-induced damage in the bladder wall).

In the manuscript, we present results from the LCA model 1, which contains the lowest BIC and AIC.

Table S1: Information criteria for 4 different LCA models

Model	*R*	*BIC*	*AIC*
*1**	7	**1537.10**	**1501.47**
2†	10	2022.63	1971.73
3‡	13	2159.76	2093.59
4•	6	1617.45	1586.91

Abbreviations: *r,* number of free parameters; *BIC,* Bayesian information criterion; *AIC,* Akaike information criterion

**Model 1:* conditional independence between the 3 diagnostic tests is assumed. No random effects are incorporated.

†Model 2: a multilevel LCA model is fitted where conditional independence is assumed and the probability that an individual would belong to the 'infected' latent class is allowed to vary across different schools.

‡Model 3: same as model 2 with the additional scenario that the diagnostic test operating characteristics varied at different school prevalence levels of *S. haematobium* infection but without making any *a priori* assumptions for those school prevalences in the fitted model. In technical terms, the conditional item response probabilities of all three examined diagnostic tests had random intercepts that varied between schools.

Model 4: we allowed for partial conditional dependence between reagent strips and microscopy. In technical terms, another unobserved (latent) continuous variable with a Gaussian distribution of zero mean and unit variance influencing the QCUF and reagent strips in the 'infected' latent class was added.

References

1. Rindskopf D, Rindskopf W (1986) The value of latent class analysis in medical diagnosis. Stat Med 5: 21-27.
2. van Smeden M, Naaktgeboren CA, Reitsma JB, Moons KG, de Groot JA (2014) Latent class models in diagnostic studies when there is no reference standard - a systematic review. Am J Epidemiol 179: 423-431.
3. Ibironke O, Koukounari A, Asaolu S, Moustaki I, Shiff C (2012) Validation of a new test for *Schistosoma haematobium* based on detection of Dra1 DNA fragments in urine: evaluation through latent class analysis. PLoS Negl Trop Dis 6: e1464.
4. Muthén LK, Muthén BO (2012) Mplus user's guide; Muthén M. Los Angeles; 856 p.
5. Simel DL, Rennie D, Bossuyt PM (2008) The STARD statement for reporting diagnostic accuracy studies: application to the history and physical examination. J Gen Intern Med 23: 768-774.
6. Koukounari A, Donnelly CA, Moustaki I, Tukahebwa EM, Kabatereine NB, et al. (2013) A latent Markov modelling approach to the evaluation of circulating cathodic antigen strips for schistosomiasis diagnosis pre- and post-praziquantel treatment in Uganda. PLoS Comput Biol 9: e1003402.
7. Henry KL, Muthén BO (2010) Multilevel latent class analysis: an application of adolescent smoking typologies with individual and contextual predictors. Struct Equ Modeling 17: 193-215.

8. Qu T, Tan M, Kutner MH (1996) Random effects models in latent class analysis for evaluating accuracy of diagnostic tests. Biometrics 52: 797-810.
