# Supplementary material for: Sensitivity and Specificity of a Urine Circulating Anodic Antigen Test for the Diagnosis of Schistosoma haematobium in Low Endemic Settings
Source: PLoS Negl Trop Dis. 2015 May 14;9(5):e0003752. doi: 10.1371/journal.pntd.0003752 (PMC4431728; doi:10.1371/journal.pntd.0003752)
Supplement: S1 Translation — (PDF) [file pntd.0003752.s004.pdf]

Nederlands:

Sensitiviteit en specificiteit van een "Circulating Anodic Antigen" test op urine voor de diagnose van *Schistosoma haematobium* in laag-endemische settings.

Samenvatting

Achtergrond: Eliminatie van schistosomiasis als een volksgezondheidsprobleem en de onderbreking van de transmissie (verspreiding) in bepaalde gebieden, zijn belangrijke doelen van de Wereld Gezondheids Organisatie voor 2025. Conventionele parasitologische methoden zijn echter niet voldoende voor het detecteren van lage infectie graden. Voor nauwkeurige diagnose in laag-transmissie gebieden en voor het verifiëren van eliminatie zijn technieken nodig met een hogere gevoeligheid en specificiteit. We hebben de nauwkeurigheid bepaald van de "Up-Converting Phosphor Lateral Flow Circulating Anodic Antigen" (UCP-LF CAA) urine test voor diagnose van *Schistosoma haematobium* infectie in een laag prevalent gebied in Zanzibar, Tanzania.

Methodologie: In 2013 werden er in totaal 1,740 urine monsters verzameld van kinderen van verschillende scholen op Pemba waar de *S. haematobium* prevalentie, gebaseerd op één urine filtratie, <2%, 2-5% en 5-10% bedroeg. Alle urine monsters werden op de dag van afname getest op microhematurie (sporen van bloed in de urine) met een strip test, en op de aanwezigheid van *S. haematobium* eieren d.m.v. microscopie. Van 1,200 urine monsters werd er 1,5 ml ingevroren (-20 °C) en acht maanden later geanalyseerd met de UCP-LF CAA test. Tegelijk werden ook de ei-tellingen (urine filtratie objectglaasjes) onderworpen aan microscopische kwaliteitscontrole (QCUF). Vanwege het ontbreken van een echte 'gouden' standaard, zijn de diagnostische resultaten berekend met behulp van "Latent Class Analysis" (LCA).

Belangrijkste bevindingen: De empirische *S. haematobium* prevalentie gemeten met de UCP-LF CAA test, de QCUF en de microhematurie strip test was respectievelijk 14%, 5% en 4%. LCA toonde een gevoeligheid van de UCP-LF CAA, QCUF en microhematurie strip test van respectievelijk 97% (95% betrouwbaarheidsinterval (CI): 91-100%), 86% (95% CI: 72-99%), en 67% (95% CI: 52-81%). Alle testen hadden een specificiteit van meer dan 90%.

Conclusies/Significantie: De UCP-LF CAA test laat een hoge gevoeligheid zien voor de diagnose van *S. haematobium* in laag-endemische gebieden. De test detecteert een aanzienlijk hoger aantal infecties dan microscopie. De UCP-LF CAA toegepast in combinatie met QCUF, is daarom een veelbelovend instrument is voor het monitoren en bewaken van urogenitale schistosomiasis in lage-transmissie gebieden, die gericht zijn op eliminatie.

Vertaling: Govert van Dam

Français:

Sensitivité et spécificité d'un dosage «circulating anodic antigen» dans les urines pour le diagnostic de *Schistosoma haematobium* en milieux à faible transmission

Résumé

Contexte: L'élimination de la schistosomiase en tant que problème de santé publique et l'interruption de la transmission en zones sélectionnées sont des objectifs clés de l'Organisation Mondiale de la Santé pour 2025. Les méthodes parasitologiques classiques sont insensibles pour la détection des infections de faible intensité. Des techniques à haute sensibilité et haute spécificité sont nécessaires pour un diagnostic précis en milieux à faible transmission et pour la vérification de l'élimination. On a déterminé la précision d'un dosage UCP-LF CAA («urine-based up-converting phosphor-lateral flow circulating anodic antigen») pour le diagnostic de *Schistosoma haematobium* en milieux à faible prévalence, à Zanzibar en Tanzanie.

Méthode: Un total de 1,740 échantillons d'urine ont été recueillis en 2013 auprès d'enfants sur l'île de Pemba, dans des écoles où la prévalence de *S. haematobium* est <2%, de 2-5% et de 5-10%, ce basé sur une unique filtration d'urine. Pour tous les échantillons, le jour même de la collecte, la présence de micro-hématurie a été déterminée à l'aide de bandelettes réactives et la présence d'œufs de *S. haematobium* par microscopie. Huit mois plus tard, 1,5 ml d'urine de chacun des 1,200 échantillons stockés à -20°C ont été analysés par dosage CAA UCP-LF, tandis que des lames de filtration d'urine ont été soumises à un contrôle de qualité (QCUF). En l'absence d'un étalon-or, la performance du diagnostic a été déterminée à l'aide d'une analyse en classes latentes (LCA).

Résultats principaux: La prévalence empirique de *S. haematobium*, révélée par UCP-LF CAA, QCUF et bandelettes réactives était de 14%, 5% et 4%, respectivement. D'après

l’analyse LCA, la sensibilité de la CAA UCP-LF, QCUF et des bandelettes réactives est de 97% (intervalle de confiance à 95% (95% IC): 91-100%), 86% (95% IC: 72-99%) et 67% (95% IC: 52-81%), respectivement. La spécificité des tests est quant à elle cohérente supérieure à 90%.

Conclusions/Importance: Le dosage UCP-LF CAA montre une haute sensibilité pour le diagnostic de *S. haematobium* en milieux à faible endémicité. Empiriquement, il détecte un nombre considérablement plus élevé d'infections que la microscopie. Par conséquent, le dosage CAA UCP-LF combiné avec QCUF est un outil prometteur pour le suivi et la surveillance de la schistosomiase urogénitale en milieux de faible transmission ciblés pour l'élimination.

Translation: Artemis Koukounari

Deutsch:

Sensitivität und Spezifität eines Urin Antigen Tests für die Diagnose von *Schistosoma haematobium* in schwach endemischen Gebieten

Zusammenfassung

Hintergrund: Die Weltgesundheitsorganisation hat sich zum Ziel gesetzt, dass die Bilharziose bis zum Jahr 2025 kein öffentliches Gesundheitsproblem mehr darstellt und dass die Übertragung des Parasiten der Gattung *Schistosoma* in bestimmten Gebieten komplett unterbunden wird. Konventionelle parasitologische Methoden sind nicht sensitiv genug, um leichte Infektionen zu diagnostizieren. Für eine akkurate Diagnose in Gebieten mit niedriger Übertragungsrate, oder zum Nachweis, dass die Übertragung der Krankheit tatsächlich unterbunden werden konnte, werden Techniken benötigt, die eine hohe Sensitivität und Spezifität besitzen. Wir untersuchten die Genauigkeit eines Urin-basierten „up-converting phosphor-lateral flow circulating anodic antigen" (UCP-LF CAA) Tests für die Diagnose von *Schistosoma haematobium* Infektionen in einem schwach endemischen Gebiet – der Insel Pemba, die zu Tansania gehört.

Methoden: Insgesamt erhielten wir 1'740 Urinproben von Kindern, die im Jahr 2013 Schulen in Pemba besuchten, wo die *S. haematobium* Prävalenz basierend auf einer einzelnen Urinfiltration <2%, 2-5% und 5-10% betrug. Alle Urinproben wurden am Tag des Einsammelns mit Teststreifen auf Hämaturie und unter einem Mikroskop auf *S. haematobium* Eier untersucht. Von 1'200 Urinproben wurden 1.5 ml bei -20°C tiefgefroren und acht Monate später mit einem UCP-LF CAA Test untersucht. Zudem wurden die Urinfilter nochmals einer Qualitätskontrolle (QK) unterzogen. Da kein wirklicher „Goldstandard" zur Verfügung steht, berechneten wir die diagnostische Leistung mit „Latent Class Analysen" (LCA).

Wichtigste Ergebnisse: Die empirische *S. haematobium* Prävalenz, die von UCP-LF CAA, QK und Teststreifen festgestellt wurde, war 14%, 5% und 4%. Die LCA bestimmte eine Sensitivität von 97% (95% Vertrauensintervall (VI): 91-100%), 86% (95% VI: 72-99%) und 67% (95% VI: 52-81%) für UCP-LF CAA, QK und Teststreifen. Alle Tests hatten eine Spezifität von über 90%.

Schlussfolgerung/Bedeutung: Die Sensitivität des UCP-LF CAA Tests für die Diagnose von *S. haematobium* Infektionen in schwach endemischen Gebieten ist hoch. Die Anzahl der von dem Test identifizieren Infektionen ist empirisch höher als bei der klassischen Mikroskopie. Aus diesem Grund ist der UCP-LF CAA, wenn er in Kombination mit qualitätskontrollierter Urinfiltration durchgeführt wird, ein vielversprechender Test für die kontinuierliche Überwachung der urogenitalen Bilharziose in schwach endemischen Gebieten, in denen die Unterbindung der Übertragung dieser Krankheit angestrebt wird.

Übersetzung: Stefanie Knopp
